# Ru-catalyzed sequence for the synthesis of cyclic amido-ethers†
†Electronic supplementary information (ESI) available. See DOI: 10.1039/c6sc02849g


**DOI:** 10.1039/c6sc02849g

**Published:** 2016-09-12

**Authors:** Barry M. Trost, Ehesan U. Sharif, James J. Cregg

**Affiliations:** a Department of Chemistry , Stanford University , 333 Campus Dr. , Stanford , CA 94035 , USA . Email: bmtrost@stanford.edu

## Abstract

Efficient synthesis of versatile building blocks for enabling medicinal chemistry research has always challenged synthetic chemists to develop innovative methods. Of particular interest are the methods that are amenable to the synthesis of chemically distinct and diverse classes of pharmaceutically relevant motifs. Herein we report a general method for the one-pot synthesis of cyclic α-amido-ethers containing different amide functionalities including lactams, tetramic acids and amino acids. For the incorporation of the nucleotide bases, a chemo and regioselective palladium-catalyzed transformation has been developed, providing rapid access to nucleoside analogs.

## Abstract 2

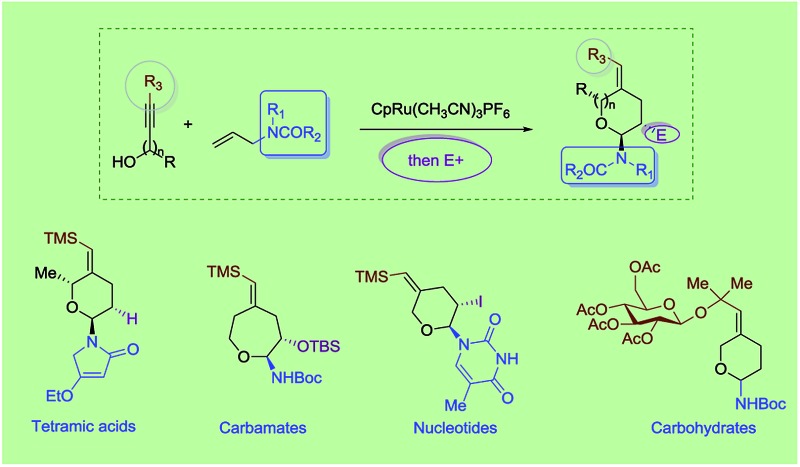
A general method for the synthesis of cyclic α-amido-ethers containing different amide functionalities including lactams, tetramic acids, amino acids and nucleoside bases.

The cyclic amido-ether scaffold is contained in a plethora of biologically relevant structures, including DNA, RNA, glycopeptides, nucleotide analogues, antisense oligonucleotides, and bioactive natural products (Fig. 1).^1–3^ However there remain few general disconnection strategies for the synthesis of such motifs. Synthetic sequences to cyclic amido-ethers almost exclusively rely on enol ethers or acetals containing a leaving group as oxonium ion precursors.^2a,3b,4^ While these strategies remain practical for the synthesis of structures with natural sugar moieties, the methods are not well suited for the rapid formation of unnatural analogs.^1a,3a–d^

**Fig. 1:**
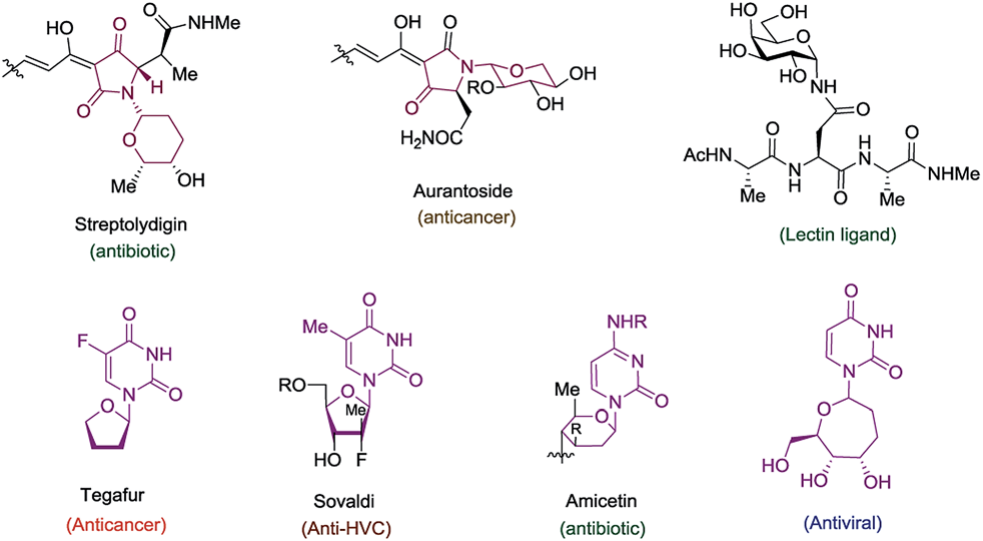
Bioactive molecules containing cyclic amido-ethers.

For unnatural carbohydrate moieties multiple steps are required to form the suitable oxonium precursor and then additional steps are needed to subsequently transform those structures into the desired amido-ethers.^4^ Given these limitations, we became interested in developing an alternative route to cyclic amido-ethers. The process would rely on the Ru-catalyzed alkene–alkyne coupling reaction developed in our lab to form both the desired C–C bond^5^ as well as a properly functionalized enamide required for cyclization (intermediate C, Scheme 1). The enamides would then be reacted with appropriate electrophiles to concomitantly form the new carbon-heteroatom bond and integration of the electrophile in the cyclized product (Scheme 1).^5*c*^

**Scheme 1:**
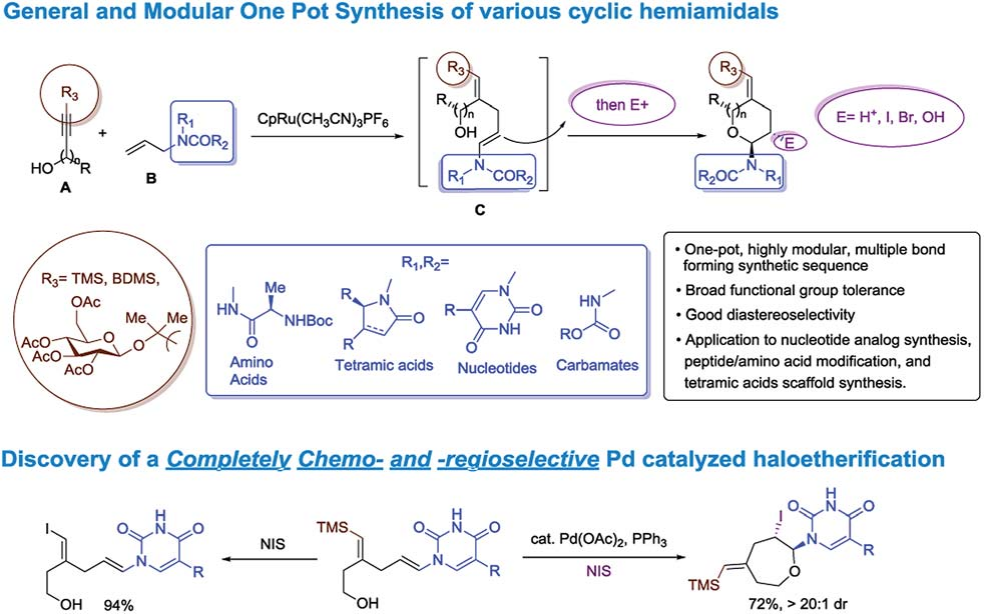
Synthetic approach toward cyclic amido-ethers.

Herein we wish to report the development and utilization of a successful reaction sequence that allows for the synthesis of highly substituted amido-ethers with high levels of diversity in one pot,^6^ while also giving a geometrically defined vinyl silane as a versatile functional group handle for further modification. A chemo- and diastereoselective Pd-catalyzed amido-etherification was also developed for the synthesis of pyrimidine nucleoside analogs. We believe that this reaction could provide a novel disconnection for future nucleotide analogue synthesis. Initial optimization studies were carried out using a simple system involving 3-(trimethylsilyl)propargyl alcohol, *tert*-butyl *N*-allylcarbamate and [CpRu(MeCN)_3_]PF_6_ in acetone. Under Ru-catalyzed alkene–alkyne coupling conditions, using 3 mol% Ru-catalyst, a small amount of the desired cyclized product **1** was observed along with 1,4-diene **D**. To facilitate the isomerization and a successive cyclization, addition of an exogenous acid was investigated (see ESI†). We found that 10 mol% of diphenylphosphate (PhO)_2_PO_2_H is ideal to carry out the reaction to yield 72% of the desired product **1**.^7^ Carrying out the reaction by incorporating a simple filtration through a plug of florisil to remove the Ru-catalyst before adding diphenylphosphate increased the yield of the product **1** to (81%). Using this modified protocol, a substrate scope involving allyl carbamates was explored (Scheme 2).

**Scheme 2:**
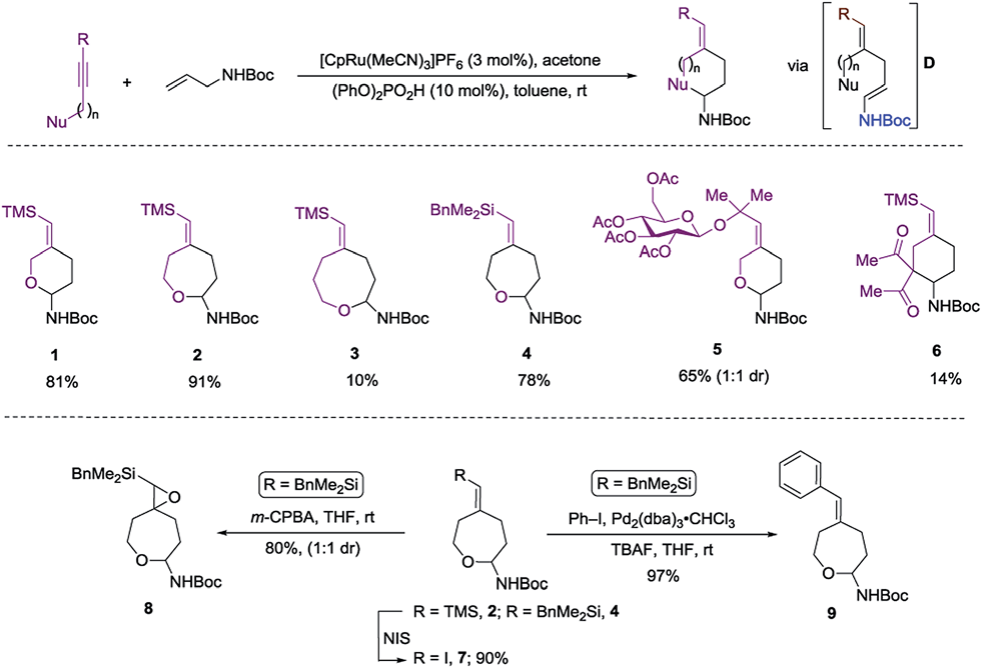
Initial investigation and substrate scope.

Both propargylic and homopropargylic alcohols coupled efficiently with *tert*-butyl *N*-allylcarbamate to form six- and seven-membered ring products (products **1** and **2**, 81% and 91%, respectively). Benzyldimethylsilyl (BDMS) could be used as a directing group in place of TMS, giving rise to the corresponding product **4** in 78% yield without changing the regioselectivity in alkene–alkyne coupling.^5a,b^ The versatility of the vinyl–BDMS was illustrated by employing it directly as a Hiyama–Denmark coupling partner to give **9** in an excellent yield of 97%.^8^ The vinylsilane moiety could also be transformed to the epoxide **8** in 80% yield or the vinyl-iodide **7** in 90% yield (Scheme 2). Interested in peptidoglycan-mimetic^9^ type structures, we incorporated glucose on the alkyne portion, which upon cyclization gave compound **5** (65%, 1 : 1 dr). Here the α-tertiary ether was used to link the carbohydrate moiety to the alkyne as well as to dictate the regioselectivity in alkene–alkyne coupling. Instead of an acid catalyzed isomerization/cyclization of enecarbamates, addition of alternate electrophiles to the enecarbamates was also studied. We found that, using *m*CPBA (3-chloroperbenzoic acid), DMDO (dimethyldioxirane) or NIS (*N*-iodosuccinimide), the cyclization could be accomplished in a chemo- and diastereoselective fashion (Scheme 3).

**Scheme 3:**
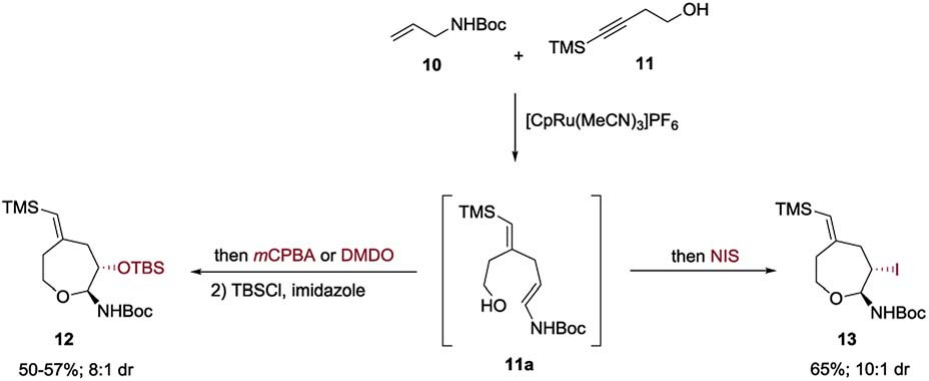
Oxidative cyclization.

Exploring the scope of the reaction led us to investigate incorporation of lactams and tetramic acid scaffolds. Tetramic acid forms the core of numerous biologically active natural products including, streptolydigin,^1*a*^ aurantoside^1*b*^ and kibdelomycin^1*c*^ (Fig. 1). Both primary and secondary alcohols as well as sterically hindered *N*-allyl partners resulted in good yields of products **14–19**. Interestingly, high diastereoselectivity (20 : 1, diastereomers assigned by NOE, see ESI for details†) was achieved in the case of secondary alcohols (product **15** and **18**), presumably because of allylic-1,3-interaction of the vinyl–TMS and the allylic methyl group, which forces the methyl group to be in an axial position in the six-membered transition state to release the steric strain (Scheme 4). Especially noteworthy is the high diastereoselectivity observed in case of a 5-substituted tetramic acid, which gave a 7 : 1 ratio of **19**.

**Scheme 4:**
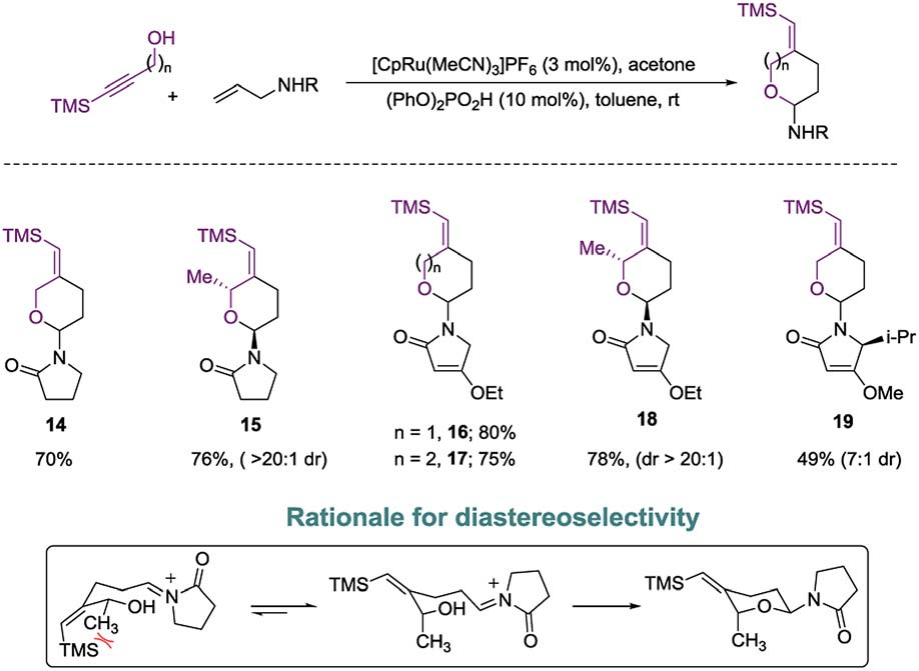
Introduction of lactams and tetramic acids.

We also examined the above amido-etherification using *N*-allylated amino acid derivatives and a dipeptide. Protein modification can impart many beneficial effects including protecting against proteolysis and influencing uptake, distribution, and excretion. Some recent investigations have revealed that attachment of carbohydrate residues to peptides, which are not glycosylated in nature, can influence the biological functions of the peptides.^2,10^ These studies show that glycosylation can be used as a tool to modify the biological activities of peptides. Thus, we initiated examination of *N*-allylated amino acids, which would readily yield the substituted pyran or oxepane analogs using our method. *N*-Allylated amino acids are readily available from commercial sources or could be prepared in one step from the corresponding ester of amino acids (see ESI†). Simple amino acids such as alanine and β-alanine were introduced in good yields both for the six- and seven-membered ring products (**20–23**, 70–84% yields). A diamino acid was also successfully introduced to form the corresponding product **24** in 82% yield (Scheme 5).

**Scheme 5:**
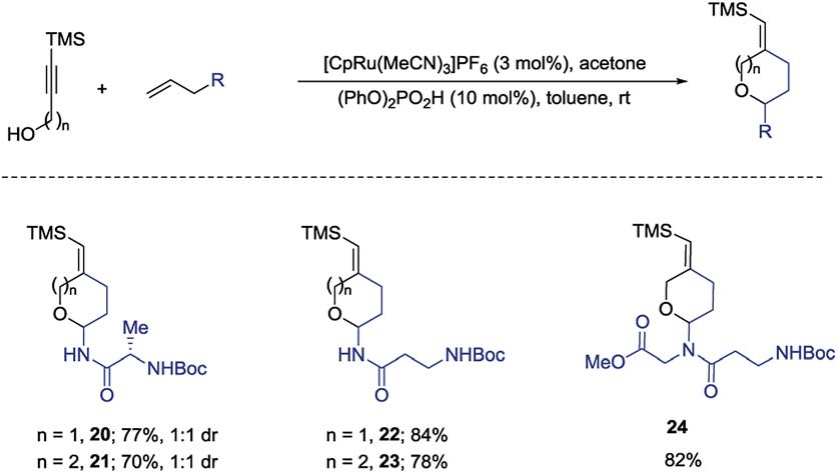
Introduction of amino acids.

A most important direction was to focus attention on the synthesis of cyclic α-amido-ethers containing nucleotide bases. Nucleoside and nucleotide analogs are important pharmacophores and have found applications in the treatment of a variety of illnesses including cancer and viral, and bacterial diseases (Fig. 1).^3^ The typical methods involved in the synthesis of nucleoside analogs largely utilize the C–N bond formation between the nucleotide base and the carbohydrate analog *via* oxo-carbenium intermediates.^11^ In contrast, construction of the carbohydrate portion on an existing nucleotide-base scaffold is rare. The later approach represents a different paradigm for analog synthesis. Surprisingly the intramolecular cyclization onto a vinyl nucleotide to form the amido-ether linkage is unknown. Thus, we aimed to utilize *N*-allyl pyrimidine bases in the alkene–alkyne coupling, wherein the newly formed enamide will be trapped by the pendant alcohol in presence of an electrophile to obtain nucleoside analogs (Scheme 6). Under our standard reaction conditions, both *N*-allyl thymine and uracil could be effectively coupled. However, no cyclization of the pendant alcohol was achieved (Scheme 6).

**Scheme 6:**
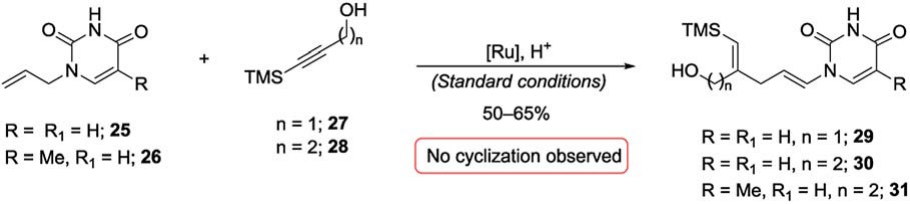
Attempted incorporation of nucleoside base.

Encouraged by our previous success in oxidative cyclization (Scheme 3), the alkene–alkyne coupled product **31** was subjected to the oxidation conditions (Scheme 7). Unfortunately, no desired cyclization was observed. For the reaction with *m*CPBA, epoxidation of the vinyl silane motif occurred to form **33**. Similarly, treatment with NIS resulted in the product of *ipso*-substitution **32** and additional equivalents of NIS did not form any cyclization product.^12^ Single-electron oxidative conditions were also explored using CAN (ceric ammonium nitrate). Although the desired product was not obtained, under these condition a six-membered cycle was formed with trapping of the nitrate anion **34** in 7 : 1 dr and 92% yield. The nitrate could be displaced in quantitative yield using NaOMe (**34** to **35**). The preferential reactivity of vinyl silane in **31** to electrophilic reagents suggests that it is a more electron rich olefin, and thus additional electrophilic oxidants would give poor selectivity for the enamide. To alleviate this problem we envisioned the thiamine or uracil acting as a ligand for a transition metal, which would direct the reactivity toward the proximal olefin rather than the more nucleophilic but distal vinyl silane. Use of Sc(OTf)_3_ in bromoamination of allyl *N*-tosylcarbamates has been reported.^13^ However, in our system, decomposition of **31** was observed. We attempted using palladium as the metal in an intramolecular Wacker-type oxidation and activation of the allylic C–H bond.^14,15^ The catalyst system proved to be inefficient and no reactivity was observed even at elevated temperature. However, when we used NIS in combination with Pd(OAc)_2_ and a phosphine ligand the desired product **36** was obtained in 72% yield (>20 : 1 dr), thus resulting in a complete switch of chemoselectivity (Scheme 6). None of the products resulting from *ipso* substitution of either the starting material or product were observed. Transition metal catalyzed haloetherifications have been reported,^16^ including a Pd(ii) catalyzed intramolecular iodoetherification of hydroxyalkenes to form tertrahydrofurans *via* an exocyclic cyclization onto the olefin.^16*a*^ Interestingly all reported intramolecular metal catalyzed haloetherifications also describe an exocyclic cyclization and only for the synthesis of 5-membered rings.^16^ Effects of chemo- and regioselectivity in such processes have not been described.

**Scheme 7:**
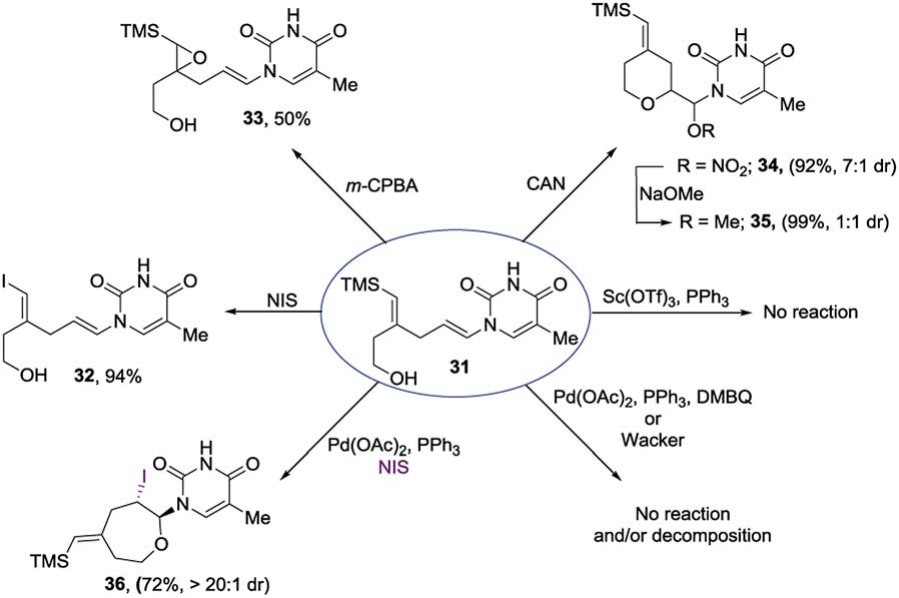
Oxidative cyclization conditions.

However, in our system the reaction proceeded by an endocyclic transition state thereby generating a 7-membered ring rather than the normally more favourable 6-membered ring. An alternative mechanism involving a cyclization to the iminium ion formed by ring opening of the intermediate iodonium ion cannot be ruled out. Additionally these conditions completely switch the chemoselectivity. The source of this marked difference in reactivity presumably derives from the presence of the nucleoside base. Controlled reactions were carried out to ascertain the role of each component. It was found that both palladium and phosphine are crucial for the cyclization event to occur. Absence of either one led to *ipso*-substitution as the sole product. Pd(0) precatalyst, Cp(allyl)Pd, was found to be the Pd species of choice and THF as the optimal solvent (see ESI†). Under the optimized conditions of amido-etherification, both six and seven membered rings could be formed in good yields and with excellent diastereoselectivity (**36–39**, 80–96% yield). The C-2 iodide of the nucleoside analog **36** can be eliminated using base to form dehydro analog **40**. NBS could also be used as the oxidant with high efficiency forming **39** in 84% yield (Scheme 8). It is worth mentioning that while furanose based nucleotides represent the most commonly derivatized structures, both six- and seven-membered nucleotides have been made and found to have very interesting properties (Fig. 1).^3b,c^ This method provides an efficient route for the rapid synthesis of such analogues.

**Scheme 8:**
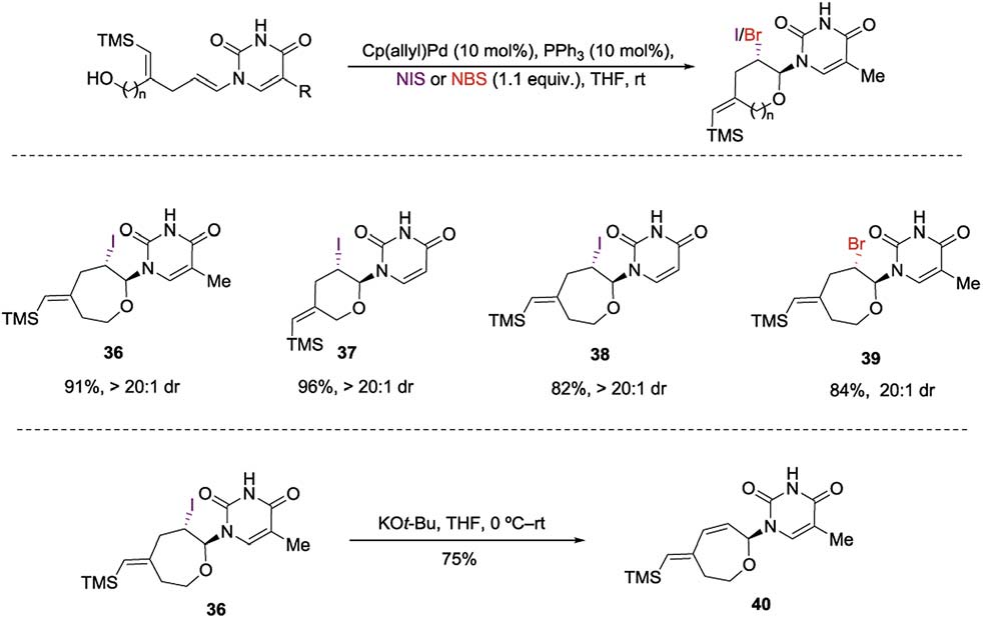
Incorporation of nucleoside bases.

## Conclusions

In summary, we have developed an efficient catalytic sequence for the synthesis of cyclic amido-ethers. The method readily allows incorporation of lactams, tetramic acids and amino acids. Cyclic ethers of varying ring size have been constructed. For the incorporation of nucleoside bases, a palladium-catalyzed chemo- and regioselective process was developed. To the best of our knowledge, this is the first example of using an intramolecular electrophile induced etherification for the synthesis of nucleoside analogs. These results stimulate many activities in furthering the chemistry and may have potential biological ramifications.

## References

[cit1] Temiakov D., Zenkin N., Vassylyeva M. N., Perederina A., Tahirov T. H., Kashkina E., Savkina M., Zorov S., Nikiforov V., Igarashi N., Matsugaki N., Wakatsuki S., Severinov K., Vassylyev D. G. (2005). Mol. Cell.

[cit2] Danishefsky S. J., Allen J. R. (2000). Angew. Chem., Int. Ed..

[cit3] (b) Storer R., Gosselin G., Dukhan D., Leroy F., Meillon J.-C., Converd T., PCT Int. Appl., WO 2007025043 A2 20070301, 2007.

[cit4] (f) Axt S., Chun B.-K., Jin Q., Rachakonda S., Ross B., Sarma K., Vitale J., Zhu J., EP20070839369, 2007; Int. Pat. Appl., WO 2008/045419, 2008.

[cit5] (c) Isomerization of allyl amides followed by trapping of the enamides with carbon nucleophiles has been reported: Sorimachi K. Terada M., J. Am. Chem. Soc., 2008, 130 , 14452 . 18850704 10.1021/ja807591m

[cit6] For improved yields, a simple filtration to remove Ru-catalyst and solvent exchange is desirable. For additional examples of Ru catalyzed multicomponent one-pot processes utilizing CpRu(MeCN)_3_PF_6_, see: Trost B. M. Koester D. C. Sharif E. U., Chem.–Eur. J., 2016, 22 , 2634 . 26669265 10.1002/chem.201504981 PMC4866645

[cit7] Diphenylphosphate causes deactivation of the [CpRu(MeCN)_3_]PF_6_ catalyst if used together presumably by formation of sandwich complex with phenyl rings. Thus, diphenylphosphate was introduced after initial alkene–alkyne coupling

[cit8] Trost B. M., Machacek M. R., Ball Z. T. (2003). Org. Lett..

[cit9] Horiya S., MacPherson I. S., Krauss I. J. (2014). Nat. Chem. Biol..

[cit10] Fisher J. F., Harrison A. W., Bundy G. L., Wilkinson K. F., Rush B. D., Ruwart M. J. (1991). J. Med. Chem..

[cit11] Qiu X., Qing F.-L. (2005). J. Org. Chem..

[cit12] Jokic M., Skaric V. (1990). J. Chem. Soc., Perkin Trans. 1.

[cit13] Huang D., Liu X., Li L., Cai Y., Liu W., Shi Y. (2013). J. Am. Chem. Soc..

[cit14] Perlmutter P., Selajerern W., Vounatsos F. (2004). Org. Biomol. Chem..

[cit15] Trost B. M., Hansmann M. M., Thaisrivongs D. A. (2012). Angew. Chem., Int. Ed..

[cit16] Doroski T. A., Cox M. R., Morgan J. B. (2009). Tetrahedron Lett..

