# Supplementary material for: Ru-catalyzed sequence for the synthesis of cyclic amido-ethers
Source: Chem Sci. 2016 Sep 12;8(1):770–4. doi: 10.1039/c6sc02849g (PMC5299796; doi:10.1039/c6sc02849g)
Supplement: Supplementary file 1 [file SC-008-C6SC02849G-s001.pdf]

Electronic Supplementary Material (ESI) for Chemical Science.
This journal is © The Royal Society of Chemistry 2016

Ru-Catalyzed Sequence for the Synthesis of Cyclic Amido-Ethers.

Barry M. Trost, Ehesan U. Sharif and James J. Cregg

Department of Chemistry, Stanford University, Stanford, California, 94305-5080, United States

Corresponding author e-mail: bmtrost@stanford.edu

Supporting Information

Optimization tables...S2
Experimental Section...S3
1H, 13C NMR Spectra S21

Optimization of Ruthenium catalyzed alkene-alkyne coupling/cyclization

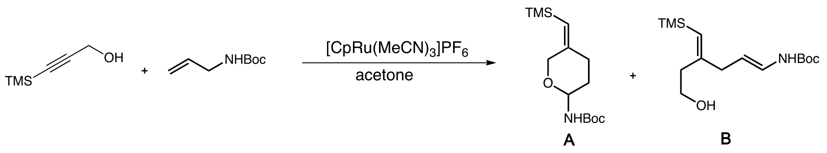

Entry	Ru-cat. (mol%)	Additive[a]	**A** : **B**	Yield of **A** (%)
1)	3	–	1:8	~ 10
2)	5	–	1:2.2	32
3)	10	–	1:1	54
4)	3	Malonic acid	only **A**	60
5)	3	Acetic acid	~1:7.5	~10
6)	3	Formic acid	~1:7	~15
7)	3	$(PhO)_2PO_2H$	only **A**	72[b]
8)	3	Dichloroacetic acid	–	trace
9)	3	Trichloroacetic acid	–	0[c]
10)	3	TFA	–	0[c]
11)	3	TsOH	–	0[c]
12)	3	Amberlyst-15	–	0[c]

[a] 10 mol% of additive was used; [b] diphenyl phosphate added after alkene alkyne coupling
[c] decomposition was observed

Optimization of Pd-catalyzed iodo-aminal formation

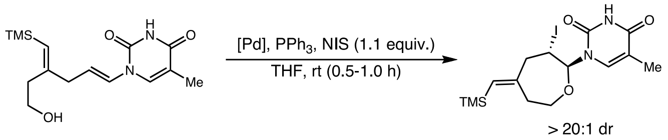

Entry	Catalyst	PPh_3	% Yield
1)	$Pd(OAc)_2$ 10 mol%	20 mol%	72
2)	$Pd(TFA)_2$ 10 mol%	20 mol%	60
3)	$Pd(OAc)_2$ 10 mol%	–	0[a]
4)	–	20 mol%	0[a]
5)	$Pd_2(dba)_3{\cdot}CHCl_3$ 5 mol%	20 mol%	86
6)	$Pd_2(dba)_3{\cdot}CHCl_3$ 5 mol%	–	trace[a]
7)	Cp(allyl)Pd 10 mol%	20 mol%	91
8)	Cp(allyl)Pd 5 mol%	10 mol%	85

[a] Ipso-substitution of vinyl-TMS by NIS was observed

Experimental section:

^{1}H and ^{13}C NMR spectra were recorded on a 400, 500 or 600 MHz spectrometer. Chemical shifts were reported relative to internal tetramethylsilane (δ 0.00 ppm) or $CDCl_3$ (δ 7.26 ppm) or CD_3OD (δ 3.30 ppm) or benzene-d_6 (δ 7.16 ppm) or pyridine-d_5 (δ 7.22, 7.58, 8.74 ppm) for 1-H NMR and CDCl3 (δ 77.23 ppm) or CD_3OD (δ 49.05 ppm) or benzene-d_6 (δ 128.39 ppm) or pyridine-d_5 (δ 150.35, 135.91, 123.87 ppm) for 13-C NMR. In the case of 19-F NMR, trifluoroacetic acid (δ –76.55 ppm) was used as an external reference for Mosher ester analyses. Infrared (IR) spectra were obtained on a FT-IR spectrometer. Optical rotations were measured with a digital polarimeter in the solvent specified. Melting points were determined with a standard melting point apparatus. Enantiomeric excess was determined using chiral Separation Products Spectra Series P-100 or 200 and UV100 (254 nm) using Chiralcel® columns (OD-H, OB-H, AD-H, OJ- H OD, OB, OJ, AD, As, OC, IA, IB or IC) eluting with heptane/iso-propanol mixtures indicated. Flash column chromatography was performed on 60-200 or 230-400 mesh silica gel. Analytical thin-layer chromatography was performed with precoated glassbacked plates and visualized by quenching of fluorescence and by charring after treatment with panisaldehyde or potassium permanganate stain. Rf values were obtained by elution in the stated solvent ratios. Diethyl ether, tetrahydrofuran, methylene dichloride and triethylamine were dried by passing through activated alumina column with argon gas pressure. Commercial reagents were used without purification unless otherwise noted. Air- and/or moisture-sensitive reactions were carried out under an atmosphere of argon/nitrogen using oven- or flame-dried glassware and standard syringe/septa techniques.

General procedures for alkene-alkyne coupling/cyclization:

Procedure A: An oven dried microwave vial was charged with alkynol (1 eq.), allyl amine (1 eq.), acid catalyst (10-mol%) and $[CpRu(MeCN)_3]PF_6$ (3-mmol%). The vial was sealed and flushed with argon for 5 min. Freshly distilled acetone (0.25 M) was added and the reaction was stirred under argon until all starting material was consumed. Solvent was evaporated under reduced pressure and the crude material was purified by silica gel flash chromatography.

Procedure B: An oven dried microwave vial was charged with alkynol (1 equiv.), allyl amine (1 equiv.) and $[CpRu(MeCN)_3]PF_6$ (3-mmol%). The vial was sealed and flushed with argon for 5 min. Freshly distilled acetone (0.25 M) was added and the reaction was stirred under argon until all starting material was consumed. The crude reaction was passed through a short plug of florisil to remove Ru-catalyst. After removing the solvent under reduced pressure, residue was redissolved in toluene (0.1M). Acid catalyst (5-10-mol%) was added and stirred at room temperature until all starting material was consumed. The reaction mixture was directly loaded onto a silica gel column and purified with EtOAc/Hexane mixture.

***tert*-butyl (*Z*)-(5-((trimethylsilyl)methylene)tetrahydro-2*H*-pyran-2-yl)carbamate (1)**

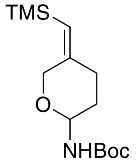

Procedure A: 3-(trimethylsilyl)prop-2-yn-1-ol (25.64 mg, 0.2 mmol), *N*-Boc allylamine (31.42 mg, 0.2 mmol), $[CpRu(MeCN)_3]PF_6$ (2.6 mg, 0.006 mmol), malonic acid (2.1 mg, 0.02 mmol) and acetone (0.8 mL, 0.25 M). Product **1** was purified on silica gel using 5% EtOAc/Hexane. Yield 29.7 mg, 52%.

Procedure B: 3-(trimethylsilyl)prop-2-yn-1-ol (25.64 mg, 0.2 mmol), *N*-Boc allylamine (31.42 mg, 0.2 mmol), $[CpRu(MeCN)_3]PF_6$ (2.6 mg, 0.006 mmol), and acetone (0.8 mL, 0.25 M), diphenylphosphate (5 mg, 0.02 mmol) and toluene (2 mL, 0.1 M). Yield of **1** was found to be 46.2 mg, 81%, waxy solid. IR 3293, 2912, 1685, 1503, 1371, 1347, 1231, 1149, 1033, 828 cm^{-1}; ^1H NMR (300 MHz; $CDCl_3$): δ 5.33 (s, 1H), 5.15-5.12 (m, 1H), 5.03-4.97 (m, 1H), 4.44 (d, J = 13.1, 1H), 4.09 (d, J = 13.1, 1H), 2.54-2.36 (m, 2H), 1.93 (ddt, J = 12.4, 5.3, 2.9, 1H), 1.67-1.54 (m, 1H), 1.46 (s, 9H), 0.11 (s, 9H) ^{13}C NMR (75 MHz; $CDCl_3$): δ 154.6, 150.5, 125.5, 80.4, 79.2, 69.0, 35.5, 32.9, 28.6, 0.5. HRMS-ESI $[C_{14}H_{27}NO_3Si+Na]^+$ calcd. 308.1652, found: 308.1658.

(*Z*)-*tert*-butyl 5-((trimethylsilyl)methylene)oxepan-2-ylcarbamate (2)

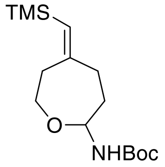

Procedure B: 4-(trimethylsilyl)but-3-yn-1-ol (28.42 mg, 0.2 mmol), *N*-Boc allylamine (31.42 mg, 0.2 mmol), $[CpRu(MeCN)_3]PF_6$ (2.6 mg, 0.006 mmol), and acetone (0.8 mL, 0.25 M), diphenylphosphate (5 mg, 0.02 mmol) and toluene (2 mL, 0.1 M). Yield 54.2 mg, 90.4%, waxy solid. IR 3292, 2910, 1686, 1589, 1498, 1347, 1231, 1154, 1085, 851, 827 cm^{-1}; ^1H NMR (400 MHz; $CDCl_3$): δ 5.32 (s, 1H), 5.12-5.11 (m, 1H), 4.97-4.95 (m, 1H), 3.93 (dt, J = 12.5, 4.8, 1H), 3.65-3.63 (m, 1H), 2.54-2.29 (m, 4H), 2.01-1.94 (m, 1H), 1.57 (dd, J = 9.4, 4.6, 1H), 1.44 (s, 9H), 0.08 (s, 9H); ^{13}C NMR (100 MHz; $CDCl_3$): δ 154.6, 154.9, 127.1, 83.3, 83.2, 66.7, 38.4, 37.4, 34.9, 28.5, 0.3. HRMS-ESI $[C_{15}H_{29}NO_3Si+Na]^+$ calcd. 322.1809, found: 322.1801.

(*E*)-*tert*-butyl 5-((trimethylsilyl)methylene)oxocan-2-ylcarbamate (3)

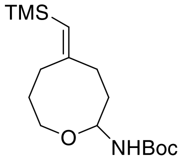

5-(trimethylsilyl)pent-4-yn-1-ol (31.3 mg, 0.2 mmol), *N*-Boc allylamine (31.42 mg, 0.2 mmol), $[CpRu(MeCN)_3]PF_6$ (2.6 mg, 0.006 mmol) was place in an oven dried microwave vial. The content was flushed with argon for 5 min and freshly distilled acetone (2 mL, 0.1 M) was added. After stirring for 1h at rt, crude reaction was passed through a short plug of florisil to remove Ru-catalyst. Solvent was removed under reduced pressure; residue was redissolved in toluene (2 ml, 0.1M) and dropwise added (using syringe pump for 12h) to a toluene solution (20 mL) containing diphenylphosphate (5 mg, 0.02 mmol). After complete addition, the reaction was stirred for an additional 12h. Solvent was removed under reduced pressure and the crude material was purified by preparative TLC (20% EtOAc/Hexane to obtain **3** as waxy solid, 6.3 mg, 10%. IR 3296, 2912, 1685, 1504, 1347, 1231, 1149, 1033, 852, 828, 681 cm^{-1}; ^1H NMR (500 MHz; $CDCl_3$): δ 5.20 (brs, 1H), 4.87-4.82 (m, 2H), 3.61-3.57 (m, 1H), 3.49-3.46 (m, 1H), 2.27- 2.17 (m, 4H), 1.78-1.62 (m, 4H), 1.44 (s, 9H), 0.07 (s, 9H); ^{13}C NMR (125 MHz; $CDCl_3$): δ 158.4, 155.4, 124.7, 81.0, 79.7, 67.4, 35.2, 34.9, 32.5, 29.1, 28.5, 0.5. HRMS-ESI $[C_{16}H_{31}NO_3Si+H]^+$ calcd. 314.2151, found: 314.2147.

tert-butyl (Z)-(5-((dimethyl(phenyl)silyl)methylene)oxepan-2-yl)carbamate (4)

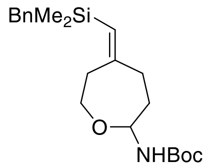

4-(benzyldimethylsilyl)but-3-yn-1-ol (100 mg, 0.46 mmol), *N*-Boc-allylamine (72 mg, 0.46 mmol) was dissolved in acetone (1.8 mL). After formation of the homogenous solution, $[RuCp(MeCN)_3]PF_6$ (6 mg, 0.014 mmol) was added and the resulted mixture was stirred for 45 min. Then the reaction was passed through a plug of florisil and concentrated. Dry toluene 1.8 mL was added to the crude reaction and subsequently added diphenyl phosphate (10 mg, 0.04 mmol). The reaction was stirred at room temperature for 2h and directly loaded into column, which was then purified by using 2–4 % EtOAc/Hexane to obtain **4** (129 mg, 78%). Colorless solid; MP: 56 ºC; IR 3145, 3074, 1770, 1681, 1266, 1238, 1183, 847, 731, 637 cm^{-1}; ^{1}H NMR (500 MHz; $CDCl_3$): δ 7.23 (t, J = 7.6, 2H), 7.09 (t, J = 7.4, 1H), 7.04-7.02 (m, 2H), 5.32 (s, 1H), 5.14-5.12 (m, 1H), 5.01-4.98 (m, 1H), 3.92-3.88 (m, 1H), 3.64-3.59 (m, 1H), 2.53-2.41 (m, 2H), 2.39-2.32 (m, 2H), 2.16 (s, 2H), 2.00 (ddt, J = 13.8, 7.4, 3.8, 1H), 1.61-1.56 (m, 1H), 1.48 (s, 9H), 0.10 (s, 3H), 0.10 (s, 3H); ^{13}C NMR (126 MHz; $CDCl_3$): δ 157.9, 154.8, 140.2, 128.3, 128.2, 124.7, 124.1, 83.2, 80.1, 66.2, 38.4, 37.4, 34.9, 28.4, 26.7, –1.6; MRMS-ESI $[C_{21}H_{33}NNaO_3Si]^+$ calcd. 398.2127, found: 398.2123.

(2*R*,3*R*,4*S*,5*R*,6*S*)-2-(acetoxymethyl)-6-((5-((*tert*-butyldimethylsilyl)oxy)-2-methylpent-3-yn-2-yl)oxy)tetrahydro-2*H*-pyran-3,4,5-triyl triacetate (5a)[1]

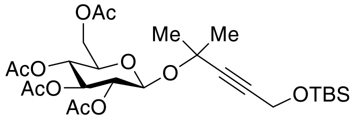

(2*R*,3*R*,4*S*,5*R*,6*S*)-2-(acetoxymethyl)-6-(2,2,2-trichloro-1-iminoethoxy)tetrahydro-2*H*-pyran-3,4,5-triyl triacetate (1.7 g, 4.4 mmol), 5-((*tert*-butyldimethylsilyl)oxy)-2-methylpent-3-yn-2-ol (500 mg, 2.2 mmol) was dissolved in dry CH_2Cl_2 and cooled to –40 ºC. To this cold stirred solution was slowly added $BF3•OEt_2$ (101 mL, 0.88 mmol). The reaction was warmed to 0 ºC and stirred until complete consumption of starting material. The reaction was then diluted with 50 mL diethyl ether and added 30 mL $NaHCO_3$. Aqueous layer was extracted with diethyl ether (3x50 mL) and the crude material was purified by silica gel column chromatography to yield **5a** (800 mg, 65%) as an oil. $[\alpha]_D^{25}$ +4.6 (c 1.0 in CH_2Cl_2). IR 1726, 1350, 1207, 1059, 824 cm^{-1}; ^{1}H NMR (400 MHz; $CDCl_3$): δ 5.38 (dd, J = 3.4, 1.0, 1H), 5.18 (dd, J = 10.5, 7.9, 1H), 5.05 (dd, J = 10.5, 3.5, 1H), 4.92 (d, J = 8.0, 1H), 4.35 (s, 2H), 4.13 (qd, J = 12.5, 6.7, 2H), 3.91 (td, J = 6.7, 1.0, 1H), 2.13 (s, 3H), 2.05 (s, 3H), 2.02 (s, 3H), 1.98 (s, 3H), 1.49 (s, 3H), 1.45 (s, 3H), 0.91 (s, 9H), 0.12 (s, 6H); ^{13}C NMR (101 MHz; $CDCl_3$): δ 170.62, 170.45, 169.82, 169.80, 97.9, 85.9, 84.4, 73.7, 71.2, 70.9, 69.0, 67.3, 61.7, 51.8, 30.5, 29.8, 26.0, 21.06, 20.89, 20.86, 18.5, –4.9; HRMS-ESI $[C_{26}H_{42}NaO_{11}Si]^+$ calcd . 581.2394, found: 581.2379.

[1] Compound **5a** is an intermediate for synthesis of **5** and is not included in the manuscript

(2*R*,3*R*,4*S*,5*R*,6*S*)-2-(acetoxymethyl)-6-((5-hydroxy-2-methylpent-3-yn-2-yl)oxy)tetrahydro-2*H*-pyran-3,4,5-triyl triacetate (5b)[2]

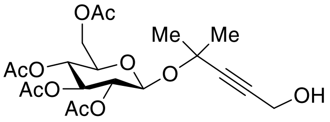

TBS-ether **5a** (666 mg, 1.2 mmol) was dissolved in dry THF (5 mL) and cooled to 0 ºC. To this cold stirred solution was added 0.5 mL HF•Pyr (70%). The reaction slowly warmed to rt over 4h. Upon completion of the starting material, was added NH_4Cl (30 mL) and the aqueous phase was extracted with ethyl acetate (3x50 mL). The crude was purified by silica gel chromatography using 30% ethyl acetate/hexane to yield **5b** (485 mg, 91%) as a clear oil. $[\alpha]_D^{25}$ +6.8 (c 1.0 in CH_2Cl_2). IR 3479, 1743, 1432, 1366, 1214, 1154, 1034, 733, 701 cm^{-1}; ^{1}H NMR (500 MHz; $CDCl_3$): δ 5.35 (d, J = 3.3, 1H), 5.13 (dd, J = 10.4, 7.9, 1H), 5.06 (dd, J = 10.4, 3.4, 1H), 4.92 (d, J = 7.9, 1H), 4.28 (s, 2H), 4.17 (dd, J = 11.3, 6.8, 1H), 4.05 (dd, J = 11.3, 6.5, 1H), 3.93 (t, J = 6.6, 1H), 2.42-2.38 (m, 1H), 2.12 (s, 3H), 2.03 (s, 3H), 2.02 (s, 3H), 1.96 (s, 3H), 1.48 (s, 3H), 1.43 (s, 3H); 13-C NMR (126 MHz; $CDCl_3$): δ 170.7, 170.47, 170.32, 169.7, 97.6, 86.5, 83.6, 73.3, 71.0, 70.6, 69.0, 67.2, 61.6, 50.8, 30.1, 29.7, 20.90, 20.76, 20.69; HRMS-ESI $[C_{20}H_{28}NaO_{11}]^+$ calcd. 467.1529, found: 467.1521.

(2*R*,3*R*,4*S*,5*R*,6*S*)-2-(acetoxymethyl)-6-(((*Z*)-1-(6-((*tert*-butoxycarbonyl)amino)dihydro-2*H*-pyran-3(4*H*)-ylidene)-2-methylpropan-2-yl)oxy)tetrahydro-2*H*-pyran-3,4,5-triyl triacetate (5)

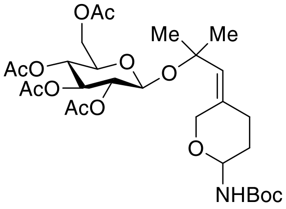

Compound **5** was prepared by following general procedure B: 100 mg, 0.23 mmol of **5b**, 71 mg, 0.45 mmol of *N*-Boc allylamine, 5 mg, 0.01 mmol of Ru-catalyst; 5 mg, 0.02 mmol diphenyl phosphate; 0.8 mL acetone and 1.0 mL toluene). Yield of **5** was 88 mg, 65%. Colorless oil mixture of 1:1 dr; IR 3460, 2976, 1750, 1455, 1365, 1216, 1033, 734 cm^{-1}; ^{1}H NMR (500 MHz; $CDCl_3$): δ 5.30 (s, 1H), 5.28-5.27 (m, 1H), 5.19 (td, J = 9.4, 1.5, 1H), 5.05-5.01 (m, 1H), 4.96-4.93 (m, 1H), 4.83-4.74 (m, 1H), 4.65 (t, J = 7.6, 1H), 4.18 (ddd, J = 12.1, 9.7, 5.4, 1H), 4.10 (ddd, J = 12.2, 9.8, 2.4, 1H), 3.96 (d, J = 13.5, 1H), 3.64 (ddd, J = 10.0, 5.5, 2.7, 1H), 2.40-2.32 (m, 1H), 2.27-2.24 (m, 1H), 2.18-2.13 (m, 1H), 2.06 (s, 3H), 2.03 (s, 3H), 2.02 (s, 3H), 2.00 (s, 3H), 1.94-1.90 (m, 1H), 1.43 (s, 9H), 1.35 (s, 3H), 1.33 (s, 3H); ^{13}C NMR (126 MHz; $CDCl_3$): δ 170.9, 170.6, 169.58, 169.42, 169.31, 154.7, 136.0, 129.8, 96.10, 95.98, 78.2, 73.3, 71.71, 71.65, 68.9, 62.5, 53.6, 33.2, 32.91, 32.76, 29.6, 29.3, 28.4, 20.91, 20.84, 20.77; HRMS-ESI $[C_{28}H_{43}NNaO_{13}]^+$ calcd 624.2632, found: 624.2616.

---

[2] Compound **5b** is an intermediate for synthesis of **5** and is not included in the manuscript

(*E*)-*tert*-butyl 2,2-diacetyl-4-((trimethylsilyl)methylene)cyclohexylcarbamate (6)

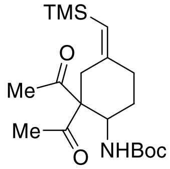

The cyclohexyl derivative **6** was prepared by general procedure B: (42.1 mg, 0.2 mmol of 3-(3-(trimethylsilyl)prop-2-yn-1-yl)pentane-2,4-dione; 31 mg, 0.2 mmol of *N*-Boc allylamine; Ru-catalyst 2.6 mg, 0.006 mmol; 5 mg, 0.02 mmol diphenylphosphate; 0.8 mL acetone and 1.0 mL toluene). Yield of **6** was 9.2 mg, 14%. Colorless solid, MP: 107 ºC; IR 3374, 2914, 2887, 1674, 1471, 1346, 1231, 1150 cm^{-1}; ^{1}H NMR (500 MHz; $CDCl_3$): δ 5.51 (d, J = 10.6, 1H), 5.29 (s, 1H), 4.34-4.29 (m, 1H), 2.92 (d, J = 14.8, 1H), 2.75 (d, J = 14.7, 1H), 2.36-2.26 (m, 2H), 2.24 (s, 3H), 2.15 (s, 3H), 1.87-1.82 (m, 1H), 1.79-1.71 (m, 1H), 1.40 (s, 9H), 0.12 (s, 9H); ^{13}C NMR (126 MHz; $CDCl_3$): δ 208.3, 207.3, 155.4, 149.5, 127.1, 79.8, 71.0, 51.6, 37.5, 36.4, 30.2, 29.2, 26.9, 0.6; HRMS-ESI $[C_{19}H_{33}NO_4SiNa]^+$ calcd. 390.2077, found: 390.2071.

***tert*-butyl (*Z*)-(5-(iodomethylene)oxepan-2-yl)carbamate (7)**

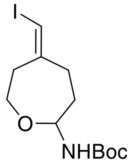

Vinyl-TMS **2** (30 mg, 0.1 mmol) was dissolved in 0.5 mL dry CH_2Cl_2. To this was added solid NIS (25 mg, 0.11 mmol) and the reaction was stirred at rt for 30 min. Solvent was removed under pressure and flash chromatography over silica gel gave vinyl iodide **7** (32 mg, 90%). Light yellow solid; MP: 101 ºC; IR 3360, 1689, 1514, 1456, 1217, 1154, 1043, 925, 884 cm^{-1}; ^{1}H NMR (500 MHz; $CDCl_3$): δ 6.09 (s, 1H), 5.10-5.07 (m, 1H), 4.99-4.95 (m, 1H), 3.99-3.96 (m, 1H), 3.71-3.66 (m, 1H), 2.66-2.60 (m, 1H), 2.57-2.46 (m, 2H), 2.44-2.39 (m, 1H), 1.98 (ddt, J = 13.9, 6.6, 3.4, 1H), 1.58-1.51 (m, 1H), 1.44 (s, 9H); ^{13}C NMR (126 MHz; $CDCl_3$): δ 154.7, 149.1, 83.3, 80.3, 77.5, 65.0, 40.5, 35.1, 34.2, 28.4; HRMS-ESI $[C_{12}H_{20}INNaO_3]^+$ calcd 376.0386, found: 376.0382.

***tert*-butyl (2-(dimethyl(phenyl)silyl)-1,6-dioxaspiro[2.6]nonan-7-yl)carbamate (8)**

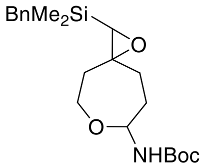

An oven-dried vial was charged with vinyl silane **4** (10 mg, 0.03 mmol). Dry CH_2Cl_2 was the added to the vial and was cooled to 0 ºC. To this mixture, was added a CH_2Cl_2 solution of *m*CPBA (9.2 mg, 0.056 mmol) and stirred over night slowly warming to rt. Saturated Na_2SO_3 (2 mL) was used to quench the reaction and aqueous phase was extracted with Et_2O and subsequently washed with $NaHCO_3$ (5 mL). Concentration under reduced pressure and silica gel chromatography gave product **8** (9 mg, 80%) as an

inseparable 2.3:1 diastereomeric mixture. Colorless wax; IR 3305, 2911, 1696, 1492, 1472, 1346, 1233, 1150, 1189, 1026, 979, 812 cm^{-1}; ^{1}H NMR (500 MHz; $CDCl_3$): δ 7.25-7.21 (m, 2H), 7.10 (d, J = 7.4, 1H), 7.02 (d, J = 7.4, 2H), 5.21-5.15 (m, 2H), 3.84-3.79 (m, 1H), 3.66-3.63 (m, 1H), 3.55-3.51 (m, 1H), 2.20 (t, J = 11.9, 2H), 2.09-2.07 (m, 2H), 1.94-1.90 (m, 2H), 1.46 (m, 11H), 0.11 (s, 3H), 0.07 (s, 3H); ^{13}C NMR (126 MHz; $CDCl_3$): δ 154.8, 139.0, 128.57, 128.21, 124.6, 81.1, 80.2, 59.8, 59.1, 37.7, 34.6, 30.2, 28.5, 24.91, –3.2, –3.6; HRMS-ESI $[C_{21}H_{33}NNaO_4Si]^+$ calcd. 414.2077, found: 414.2058.

tert-butyl (*Z*)-(5-benzylideneoxepan-2-yl)carbamate (9)

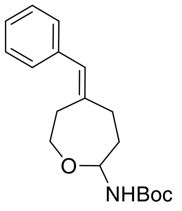

The vinyl BDMS **4** (20 mg, 0.053 mmol), and phenyl iodide (12 mg, 0.059 mmol) was dissolved in dry THF (0.3 mL). To this solution was added $Pd_2(dba)_3$•$CHCl_3$ (2.7 mg, 0.003 mmol) and TBAF (0.1 mL, 0.11 mmol). The mixture was stirred at rt for 5 min and quenched with saturated 10 mL $NaHCO_3$. Aqueous layer was extracted with Et_2O (3x10 mL), dried over $MgSO_4$ and filtering through a short plug of silica gave pure produce **9** (10 mg, 97%). Colorless solid; MP: 60 ºC; IR 3315, 2930, 1689, 1519, 1390, 1307, 1168, 1043, 1018, 786 cm^{-1}; ^{1}H NMR (500 MHz; $CDCl_3$): δ 7.31 (t, J = 7.6, 2H), 7.20 (t, J = 6.7, 3H), 6.40 (s, 1H), 5.15-5.13 (m, 1H), 5.07-5.03 (m, 1H), 3.99-3.97 (m, 1H), 3.75-3.71 (m, 1H), 2.73-2.67 (m, 1H), 2.60-2.57 (m, 1H), 2.54-2.49 (m, 1H), 2.45-2.40 (m, 1H), 2.11-2.04 (m, 1H), 1.69-1.66 (m, 1H), 1.45 (s, 9H); ^{13}C NMR (126 MHz; $CDCl_3$): δ 154.8, 140.6, 137.9, 128.8, 128.2, 127.4, 126.4, 83.3, 80.1, 66.2, 35.5, 34.60, 34.51, 28.5; HRMS-ESI [$C_{18}H_{25}NNaO_3]^+$ calcd 326.1732, found: 326.1728.

tert-butyl((2*S*,3*S*,*E*)-3-((*tert*-butyldimethylsilyl)oxy)-5-((trimethylsilyl)methylene)oxepan-2-yl)carbamate (12)

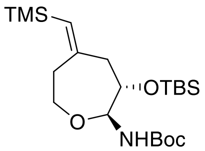

4-(trimethylsilyl)but-3-yn-1-ol (14.2 mg, 0.1 mmol) and *N*-Boc allylamine (15.7 mg, 0.1 mmol) were dissolved in acetone and added $[RuCp(MeCN)_3]PF_6$ (1.3 mg, 0.003 mmol). After 30 min, *m*CPBA (34 mg, 0.2 mmol) or excess DMDO was added. The reaction mixture was stirred at rt for additional 8 h in case of *m*CPBA, and 2h at –78 ºC for DMDO. The reaction was quenched by saturated Na_2SO_3 (4 mL) and aqueous phase was extracted with Et_2O (3x10 mL). The organic phase was washed with $NaHCO_3$ (10 mL). Solvent was removed under pressure followed by filtration through a silica pad gave crude product, which was dissolved in 1.0 mL dry CH_2Cl_2 and added TBSCl (15 mg, 0.1 mmol), and imidazole (7 mg, 0.1 mmol). The reaction was stirred at room temperature over night. Solvent was removed under pressure and the crude material was subjected to silica gel chromatography to obtain **12** (21 mg, 50% with *m*CPBA and 24.5 mg, 57% for DMDO as 8:1 dr). Colorless oil; IR 2952, 2930, 1726, 1483, 1167, 1071, 861, 834, 776 cm^{-1}; ^{1}H NMR (500 MHz; $CDCl_3$): δ 5.73 (d, J = 9.2, 1H), 5.35 (s, 1H), 4.82-4.79 (m, 1H), 4.16-4.12 (m, 1H), 3.82 (ddd, J = 8.3, 6.2, 3.3, 1H), 3.56-3.51 (m, 1H), 2.64 (ddd, J = 12.4, 6.2, 0.9, 1H), 2.46-2.44 (m, 2H), 2.29

(dd, J = 12.2, 8.5, 1H), 1.43 (s, 9H), 0.91 (s, 9H), 0.09 (s, 6H), 0.08 (s, 9H); ^{13}C NMR (126 MHz; $CDCl_3$): δ 155.0, 151.8, 129.9, 84.8, 79.8, 72.3, 70.2, 47.1, 39.6, 28.4, 25.9, 18.3, 0.3, -4.1, -5.1; HRMS-ESI $[C_{21}H_{43}NNaO_4Si_2]^+$ calcd. 452.2628, found: 452.261.

tert-butyl (_Z_)-(5-((trimethylsilyl)methylene)oxepan-2-yl)carbamate (13)

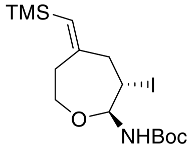

4-(trimethylsilyl)but-3-yn-1-ol (14.2 mg, 0.1 mmol) and *N*-Boc allylamine (15.7 mg, 0.1 mmol) were dissolved in acetone and added $[RuCp(MeCN)_3]PF_6$ (1.3 mg, 0.003 mmol). After 30 min, NIS (25 mg, 0.11 mmol) was added. The reaction mixture was stirred at rt for additional 30 min. Removal of solvent under pressure followed by flash chromatography gave product **13** (28 mg, 65%). Colorless solid; MP: 87 ºC; IR 3333, 2953, 2925, 1718, 1367, 1159, 1081, 804 cm^{-1}; ^{1}H NMR (500 MHz; $CDCl_3$): δ 5.57 (s, 1H), 5.13-5.09 (m, 1H), 5.06-4.99 (m, 1H), 4.06-4.02 (m, 1H), 3.96 (ddd, J = 9.8, 8.0, 4.8, 1H), 3.72-3.66 (m, 1H), 3.02-2.93 (m, 2H), 2.61 (ddd, J = 15.5, 10.2, 5.9, 1H), 2.40 (dddd, J = 15.0, 4.3, 2.8, 1.4, 1H), 1.45 (s, 9H), 0.11 (s, 9H); ^{13}C NMR (126 MHz; $CDCl_3$): δ 154.6, 152.3, 131.8, 89.8, 80.5, 68.2, 49.7, 37.5, 32.7, 28.4, 0.1; HRMS-ESI $[C_{15}H_{28}INNaO_3Si]^+$ calcd 448.0781, found: 448.0768.

(_Z_)-1-(5-((trimethylsilyl)methylene)tetrahydro-2H-pyran-2-yl)pyrrolidin-2-one (14)

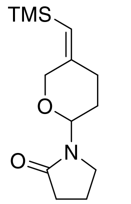

Compound **14** was prepared by following general procedure B: using 3-(trimethylsilyl)prop-2-yn-1-ol (25.64 mg, 0.2 mmol), 1-allylpyrrolidin-2-one (25 mg, 0.2 mmol), $[CpRu(MeCN)_3]PF_6$ (8.7 mg, 0.02 mmol), acetone (0.4 mL, 0.5 M), diphenylphosphate (5 mg, 0.02 mmol) and toluene (1 mL, 0.2 M). Flash chromatography using 66% EtOAc:Hexane gave the product as a white solid (36 mg, 70% yield). MP: 70-71 ºC; IR (thin film): 2912.6, 1679.8, 1400.4, 1231.0, 1051.7, 847.0; ^{1}H-NMR (500 MHz; $CDCl_3$): δ 5.33-5.30 (m, 2H), 4.43 (dd, *J* = 13.0, 1.9 Hz, 1H), 4.11 (d, *J* = 13.0 Hz, 1H), 3.53 (td, *J* = 8.8, 5.9 Hz, 1H), 3.37 (ddd, *J* = 9.3, 8.3, 6.3 Hz, 1H), 2.56 (d, *J* = 0.3 Hz, 1H), 2.40 (t, *J* = 8.2 Hz, 3H), 2.05-1.97 (m, 2H), 1.86-1.78 (m, 1H), 1.72 (ddt, *J* = 9.9, 5.0, 2.5 Hz, 1H), 0.09 (s, 9H). ^{13}C NMR (126 MHz; $CDCl_3$): δ 175.5, 150.3, 125.5, 79.1. 70.1, 42.7, 35.7, 31.6, 30.4, 18.2, 0.4; HRMS-ESI $[C_{13}H_{24}NO_2Si]^+$ calcd: 254.1578, found: 254.1571.

1-((2*S*,6*R*,*Z*)-6-methyl-5-((trimethylsilyl)methylene)tetrahydro-2*H*-pyran-2-yl)pyrrolidin-2-one (15)

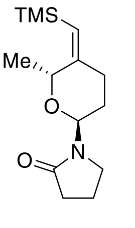

Compound **14** was prepared by following general procedure B: using 4-(trimethylsilyl)but-3-yn-2-ol (28.4mg, 0.2 mmol), 1-allylpyrrolidin-2-one (50 mg, 0.4 mmol), $[CpRu(MeCN)_3]PF_6$ (8.7 mg, 0.02 mmol), acetone (0.4 mL, 0.5 M), diphenylphosphate (5 mg, 0.02 mmol) and toluene (1 mL, 0.2 M). Flash chromatography using 40%t:PE gave the product as a white solid (41 mg, 76% yield). MP: 130-131 ºC; IR (thin film): 2915.7, 2892.5, 1668.3, 1232.5, 827.5; ^{1}H NMR (500 MHz; $CDCl_3$): δ 5.64 (d, J = 10.7, 1H), 5.21 (s, 1H), 4.70 (q, J = 6.8, 1H), 3.50 (q, J = 7.3, 1H), 3.33 (q, J = 7.7, 1H), 2.76-2.69 (m, 1H), 2.38 (t, J = 8.1, 2H), 2.24 (d, J = 14.3, 1H), 2.02-1.95 (m, 2H), 1.78-1.69 (m, 2H), 1.44 (d, J = 6.9, 3H), 0.09 (s, 9H); ^{13}C NMR (126 MHz; $CDCl_3$): δ 175.3, 154.0, 124.0, 74.4, 72.6, 42.3, 32.2, 31.7, 30.9, 18.6, 18.2, 0.27. HRMS-ESI $[C_{13}H_{24}NO_2Si]^+$ calcd: 268.1733, found: 268.1727.

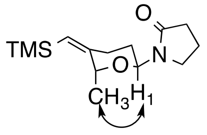

NOE on methyl
gives 3.6%
NOE on H_1

1-allyl-4-ethoxy-1,5-dihydro-2*H*-pyrrol-2-one (16a)[3]

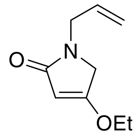

Ethyl allylglycinate (500 mg, 3.5 mmol) was dissolved in dry toluene (15 mL, 0.3 M) and cooled to 0 ºC. To this cold solution was added 3.5 mL of 1M HCl in Et_2O (dry). The mixture was stirred at rt for 30 min upon which the amine salt precipitates out. At this point, methyl 2-(triphenyl-λ^5-phosphanylidene)acetate (1.52 g, 4.54 mmol) was added and the content was refluxed for 6h. The reaction mixture was then cooled to 0 ºC, and quenched with 10 mL $NaHCO_3$. The aqueous phase was extracted with EtOAc (3x50 mL), dried over $MgSO_4$. The product and Ph_3P=O has similar R_f. Recrystallization with 1:1 Et_2O/Hexane removed most of Ph_3P=O. Finally, column chromatography with 35-40% EtOAc/Hexane gave the desired product **16a** (421 mg, 72%) as a mixture with 10% Ph_3P=O, which was directly use in the alkene-alkyne coupling reaction. Colorless oil; IR 3406, 2941, 2864, 1656, 1598, 1435, 1355, 1323, 1203, 1101, 1015 cm^{-1}; ^{1}H NMR (500 MHz; $CDCl_3$): δ 5.74 (ddt, J = 16.9, 10.3, 5.9, 1H), 5.15-5.10 (m, 2H), 5.00 (s, 1H), 3.99-3.94 (m, 4H), 3.78 (s, 2H), 1.36 (t, J = 7.1, 3H); ^{13}C NMR (126 MHz; $CDCl_3$): δ 172.41, 172.21, 133.6, 117.4, 94.4, 67.0, 50.3, 44.1, 14.2. HRMS-ESI $[C_9H_{14}NO_2]^+$ calcd. 168.1024, found: 168.1019.

[3] Compound **16a** is an intermediate for synthesis of **16**,**17** and **18** and is not included in the manuscript

(*Z*)-4-ethoxy-1-(5-((trimethylsilyl)methylene)tetrahydro-2*H*-pyran-2-yl)-1H-pyrrol-2(5*H*)-one (16)

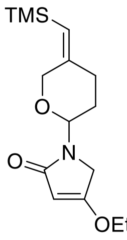

3-(trimethylsilyl)prop-2-yn-1-ol (25.64 mg, 0.2 mmol), *N*-allyl lactic **16a** (67 mg, 0.4 mmol) was dissolved in acetone (0.8 mL, 0.25 M). The content was flushed with argon and $[CpRu(MeCN)_3]PF_6$ (9.0 mg, 0.02 mmol) was added. The reaction mixture was sealed and stirred for 12 h. Analysis of the crude reaction mixture showed formation of cyclized product **16** without any added acid. The crude reaction was concentrated to remove solvent and product **16** was purified by flash chromatography using 35% EtOAc/Hexane, 47 mg, 80%. Colorless solid, MP: 98 ºC; IR 3436, 2910, 1674, 1599, 1428, 1354, 1319, 1231, 1208, 1089, 1048 cm^{-1}; ^{1}H NMR (500 MHz; $CDCl_3$): δ 5.41-5.38 (m, 1H), 5.31 (d, J = 1.9, 1H), 5.01 (s, 1H), 4.39 (dd, J = 13.0, 2.2, 1H), 4.11 (d, J = 13.0, 1H), 4.02 (d, J = 17.1, 1H), 3.97 (q, J = 7.1, 2H), 3.80-3.77 (m, 1H), 2.56-2.49 (m, 1H), 2.42-2.38 (m, 1H), 1.80-1.75 (m, 2H), 1.35 (t, J = 7.1, 3H), 0.07 (s, 9H); ^{13}C NMR (126 MHz; $CDCl_3$): δ 174.0, 172.9, 150.5, 125.4, 94.4, 77.8, 69.8, 67.2, 46.6, 35.9, 31.2, 14.2, 0.3. MRMS-ESI $[C_{15}H_{26}NO_3Si]^+$ calcd. 296.1682, found: 296.1676.

(*Z*)-4-ethoxy-1-(5-((trimethylsilyl)methylene)oxepan-2-yl)-1*H*-pyrrol-2(5*H*)-one (17)

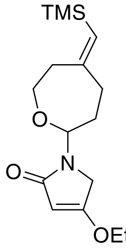

Compound **17** was prepared by using general procedure B: (28.42 mg, 0.2 mmol of 4-(trimethylsilyl)but-3-yn-1-ol; 67 mg, 0.4 mmol of **16a**; $[CpRu(MeCN)_3]PF_6$ (8.7 mg, 0.02 mmol); 5 mg, 0.02 mmol diphenylphosphate; 0.8 mL acetone and 1.0 mL toluene). Yield of **17** was 46.4 mg, 75%. Colorless solid, MP: 92 ºC; IR 3368, 2908, 1673, 1599, 1428, 1354, 1319, 1230, 1073, 1011 cm^{-1}; ^{1}H NMR (500 MHz; $CDCl_3$): δ 5.39 (dd, J = 9.7, 3.6, 1H), 5.36 (s, 1H), 5.01 (s, 1H), 4.03-3.92 (m, 4H), 3.81 (d, J = 17.1, 1H), 3.70 (ddd, J = 12.5, 10.1, 3.5, 1H), 2.58 (ddd, J = 15.5, 10.2, 5.1, 1H), 2.52-2.45 (m, 2H), 2.42-2.37 (m, 1H), 1.86-1.82 (m, 2H), 1.37 (t, J = 7.1, 3H), 0.09 (s, 9H); ^{13}C NMR (126 MHz; $CDCl_3$): δ 173.9, 172.7, 156.5, 127.1, 94.4, 82.0, 67.8, 67.2, 46.7, 37.91, 37.75, 33.6, 14.2, 0.2; MRMS-ESI $[C_{16}H_{28}NO_3Si]^+$ calcd. 310.1838, found: 310.1828.

4-ethoxy-1-((2*S*,6*R*,*Z*)-6-methyl-5-((trimethylsilyl)methylene)tetrahydro-2*H*-pyran-2-yl)-1,5-dihydro-2*H*-pyrrol-2-one (18)

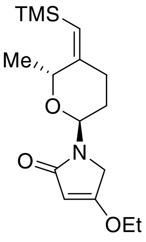

Compound **18** was prepared by following general procedure B : using 4-(trimethylsilyl)but-3-yn-2-ol (28.4mg, 0.2 mmol, 1 equiv.) 1-allyl-4-ethoxy-1,5-dihydro-2H-pyrrol-2-one **16a** (66.8 mg, 0.4 mmol, 2 equiv), $[CpRu(MeCN)_3]PF_6$ (8.7 mg, 0.02 mmol), acetone (0.8 mL, 0.25 M), diphenylphosphate (added in 2 portions. Second portion added after 24h) (10 mg, 0.04 mmol) and toluene (1 mL, 0.2 M). Let stir at RT for 70 h. Flash chromatography eluting with 60% EtOAc:Hexane gave the product as a white solid (47 mg, 76% yield). MP 79-80 °C; IR: 2911, 1678, 1601, 1319, 832 cm^{-1}; ^1H NMR (400 MHz; $CDCl_3$): δ 5.73 (dd, J = 11.0, 2.6, 1H), 5.21 (d, J = 1.8, 1H), 4.99 (s, 1H), 4.67 (q, J = 6.9, 1H), 4.03-3.94 (m, 3H), 3.75 (d, J = 17.2, 1H), 2.79-2.70 (m, 1H), 2.24 (dd, J = 14.3, 2.9, 1H), 1.81-1.69 (m, 2H), 1.44 (d, J = 6.9, 3H), 1.34 (t, J = 7.1, 3H), 0.08 (s, 9H); ^{13}C NMR (126 MHz; $CDCl_3$): δ 174.0, 172.9, 154.2, 123.9, 94.4, 74.3, 71.3, 67.2, 46.3, 32.5, 31.9, 18.6, 14.2, 0.3 LRMS-ESI $[C_{16}H_{28}NO_3Si]^+$ calcd. 310.2 , found: 310.6

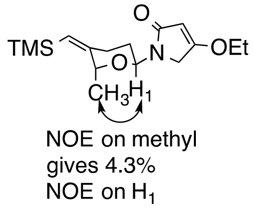

(*S*)-1-allyl-5-isopropyl-4-methoxy-1*H*-pyrrol-2(5*H*)-one (19a)[4]

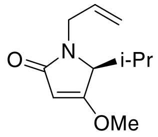

N-allyl L-valine methylester (560 mg, 3.27 mmol) was dissolved in dry toluene (11 mL, 0.3 M) and cooled to 0 ºC. To this cold solution was added 1.5 mL of 2M HCl in dry Et_2O (2.9 mmol). The mixture was stirred at rt for 30 min upon which the amine salt precipitates out. At this point, Wittig salt methyl 2-(triphenyl-λ^5-phosphanylidene)acetate (1.6 g, 4.9 mmol) was added and the content was refluxed for 12h. The reaction mixture was then cooled to 0 ºC, and quenched with 10 mL $NaHCO_3$. The aqueous phase was extracted with EtOAc (3x50 mL), dried over $MgSO_4$. The product was purified by column chromatography eluting with 30-35% EtOAc/Hexane gave the desired product **19a** (350 mg, 54%). Colorless oil; $[\alpha]_D^{25}$ +35.2 (c 1.0 in CH_2Cl_2); IR 1680, 1620, 1408, 1322, 1231, 996, 929, 802 cm^{-1}; ^1H NMR (500 MHz; $CDCl_3$): δ 5.72 (dddd, J = 16.9, 10.4, 7.4, 4.6, 1H), 5.14 (sextet, J = 1.4, 1H), 5.12-5.10 (m, 1H), 5.05 (s, 1H), 4.49-4.44 (m, 1H), 3.87 (d, J = 2.8, 1H), 3.75 (s, 3H), 3.47 (ddt, J = 15.8, 7.4, 1.1, 1H), 2.14 (dseptet, J = 7.0, 2.5, 1H), 1.02 (d, J = 7.1, 3H), 0.77 (d, J = 6.9, 3H); ^{13}C NMR (126 MHz; $CDCl_3$): δ 176.1, 172.0, 133.9, 117.3, 94.6, 64.5, 58.0, 42.4, 28.0, 18.1, 15.8 HRMS-ESI $[C_{11}H_{17}NNaO_2]^+$ calcd. 218.1157, found: 218.1149.

[4] Compound **19a** is an intermediate for synthesis of **19** and is not included in the manuscript

(5*S*)-5-isopropyl-4-methoxy-1-((*Z*)-5-((trimethylsilyl)methylene)tetrahydro-2*H*-pyran-2-yl)-1,5-dihydro-2*H*-pyrrol-2-one (19)

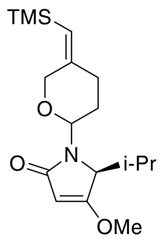

3-(trimethylsilyl)prop-2-yn-1-ol (19 mg, 0.15 mmol), *N*-allyl lactic **19a** (30 mg, 0.15 mmol) was dissolved in acetone (0.6 mL, 0.25 M). The content was flushed with argon and [CpRu(MeCN)$_3$]PF$_6$ (6.5 mg, 0.015 mmol) was added. The reaction mixture was sealed and stirred for 12 h. Analysis of the crude reaction mixture showed formation of cyclized product **19** along with a minor diastereomer in ~7:1 ratio without any added acid. Solvent was removed under reduced pressure and the product **19** was purified by flash chromatography using 25-30% EtOAc/Hexane, 23 mg, 49%. Colorless oil, IR 3292, 1917, 2884, 2813, 1671, 1601, 1440,1329, 1229, 1180, 1048, 1020 cm^{-1}; ^{1}H NMR (500 MHz; CDCl$_3$): δ 5.42 (dd, J = 11.3, 1.9, 1H), 5.34 (s, 1H), 5.06 (s, 1H), 4.43 (dd, J = 12.9, 1.8, 1H), 4.10 (d, J = 12.9, 1H), 4.01 (d, J = 2.4, 1H), 3.78 (s, 3H), 2.55-2.43 (m, 3H), 2.00-1.97 (m, 1H), 1.79-1.74 (m, 1H), 1.08 (d, J = 7.3, 3H), 0.82 (d, J = 6.7, 3H), 0.12 (s, 9H); ^{13}C NMR (126 MHz; CDCl$_3$): δ 177.1, 172.0, 151.3, 125.4, 94.3, 79.0, 70.0, 64.2, 58.2, 36.4, 31.0, 29.6, 18.7, 15.6, 0.5; MRMS-ESI [$C_{17}H_{29}NNaO_3Si$]$^+$ calcd. 346.1814, found: 346.1813.

***tert*-butyl (2*S*)-1-oxo-1-((*Z*)-5-((trimethylsilyl)methylene)tetrahydro-2*H*-pyran-2-ylamino)propan-2-ylcarbamate (20)**

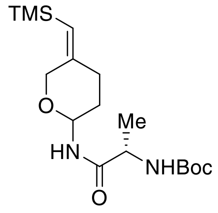

The alanine derivative **20** was prepared by general procedure B: (46 mg, 0.2 mmol of (*S*)-*tert*-butyl-1-(allylamino)-1-oxopropan-2-ylcarbamate; 25.6 mg, 0.2 mmol of 3-(trimethylsilyl)prop-2-yn-1-ol; [CpRu(MeCN)$_3$]PF$_6$ (2.6 mg, 0.006 mmol); 5 mg, 0.02 mmol diphenylphosphate; 0.8 mL acetone and 1.0 mL toluene). Yield of **20** was 55 mg, 77% (1:1 dr). Colorless oil; IR 3266, 2935, 2912, 1651, 1511, 1431, 1347, 1231, 1152, 1052, 1014 cm^{-1}; Spectral data for one diastereomer is reported. ^{1}H NMR (500 MHz; CDCl$_3$): δ 7.09-7.07 (m, 1H), 5.30 (s, 1H), 5.25-5.12 (m, 2H), 4.37 (t, J = 12.2, 1H), 4.18-4.16 (m, 1H), 4.07 (d, J = 13.2, 1H), 2.48-2.43 (m, 1H), 2.39-2.35 (m, 1H), 1.92-1.86 (m, 1H), 1.65-1.56 (m, 1H), 1.41 (s, 9H), 1.33 (t, J = 6.6, 3H), 0.07 (s, 9H); ^{13}C NMR (126 MHz; CDCl$_3$): δ 172.7, 155.7, 150.2, 125.5, 80.3, 77.2, 68.9, 50.0, 35.0, 32.5, 28.4, 18.2, 0.3. HRMS-ESI [$C_{17}H_{33}N_2O_4Si$]$^+$ calcd. 357.2209, found: 357.2204.

tert-butyl-(2_S_)-1-oxo-1-((_Z_)-5-((trimethylsilyl)methylene)oxepan-2-ylamino)propan-2-ylcarbamate (21)

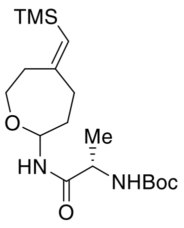

The D-alanine derivative **21** was prepared by general procedure B: (46 mg, 0.2 mmol of (_S_)-_tert_-butyl-1-(allylamino)-1-oxopropan-2-ylcarbamate; 28.42 mg, 0.2 mmol of 4-(trimethylsilyl)but-3-yn-1-ol; [CpRu(MeCN)$_3$]PF$_6$ (2.6 mg, 0.006 mmol); 5 mg, 0.02 mmol diphenylphosphate; 0.8 mL acetone and 1.0 mL toluene). Yield of **21** was 52 mg, 70%. Colorless oil as a mixture of diastereomers (1:1). IR 3264, 2909, 1649, 1589, 1515, 1432, 1374, 1230, 1153, 1010 cm^{-1}; ^{1}H NMR for one diastereomer (500 MHz; CDCl$_3$): δ 6.91 (dt, J = 0.9, 0.4, 1H), 5.33 (s, 1H), 5.23-5.18 (m, 1H), 5.06-5.06 (m, 1H), 4.18-4.11 (m, 1H), 3.90 (ddt, J = 13.6, 9.3, 4.7, 1H), 3.65 (dddd, J = 12.6, 9.1, 3.6, 1.8, 1H), 2.55 (ddt, J = 14.7, 9.6, 4.8, 1H), 2.47-2.39 (m, 2H), 2.34 (ddd, J = 13.7, 7.4, 4.3, 1H), 1.96 (dtd, J = 14.7, 7.5, 4.2, 1H), 1.64 (dtd, J = 14.0, 9.6, 4.4, 1H), 1.43 (s, 9H), 1.33 (dd, J = 7.1, 4.5, 3H), 0.08 (s, 9H); ^{13}C NMR for one diastereomer (126 MHz; CDCl$_3$): δ 172.3, 156.5, 155.8, 127.1, 81.3, 66.6, 50.2, 38.1, 37.28, 37.24, 34.6, 28.4, 18.1, 0.2. HRMS-ESI [$C_{18}H_{34}N_2NaO_4Si$]$^+$ calcd. 393.2186, found: 393.2164.

To eliminate the possibility of any rotamers, high temperature NMR studies were carried out. No coalescence of peak (notably for TMS and the methyl group of alanine) was observed ^{1}H NMR at 23 ºC (400 MHz; toluene): δ 5.59-5.47 (m, 1H), 5.41-5.33 (m, 2H), 4.29-4.27 (m, 1H), 3.87 (dt, J = 12.4, 4.8, 1H), 3.63-3.56 (m, 1H), 2.53 (ddd, J = 15.0, 9.6, 5.1, 1H), 2.35-2.24 (m, 3H), 2.16 (dt, J = 4.4, 2.2, 1H), 1.83-1.77 (m, 1H), 1.64-1.56 (m, 1H), 1.47 (s, 9H), 1.27 (dd, J = 7.1, 3H), 0.18 (d, J = 2.4, 9H); ^{1}H NMR at 80 ºC (400 MHz; toluene): δ 6.50 (d, J = 39.7, 1H), 5.38 (s, 1H), 5.32-5.31 (m, 1H), 5.06-4.95 (m, 1H), 4.11-4.05 (m, 1H), 3.87-3.82 (m, 1H), 3.60-3.54 (m, 1H), 2.56-2.49 (m, 1H), 2.39-2.34 (m, 1H), 2.30-2.26 (m, 2H), 2.17 (d, J = 2.0, 1H), 1.84-1.81 (m, 1H), 1.47 (s, 9H), 1.20 (d, J = 4.6, 3H), 0.17 (s, 9H).

(_Z_)-_tert_-butyl-3-oxo-3-(5-((trimethylsilyl)methylene)tetrahydro-2_H_-pyran-2-ylamino) propylcarbamate (22)

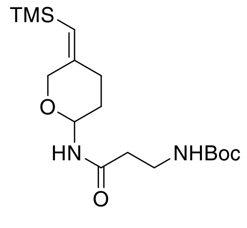

The β-alanine derivative **22** was prepared by general procedure B: (46 mg, 0.2 mmol of _tert_-butyl (3-(allylamino)-3-oxopropyl)carbamate; 25.6 mg, 0.2 mmol of 3-(trimethylsilyl)prop-2-yn-1-ol; [CpRu(MeCN)$_3$]PF$_6$ (2.6 mg, 0.006 mmol); 5 mg, 0.02 mmol diphenylphosphate; 0.8 mL acetone and 1.0 mL toluene). Yield of **22** was 60 mg, 84%. Colorless solid, MP: 117 ºC; IR 3275, 2919, 1669, 1516, 1346, 1231, 1156, 1051, 1019 cm^{-1}; ^{1}H NMR (500 MHz; CDCl$_3$): δ 6.53-6.51 (m, 1H), 5.32 (d, J = 1.2, 1H), 5.25-5.21 (m, 2H), 4.39 (dd, J = 13.0, 1.6, 1H), 4.08 (d, J = 13.0, 1H), 3.43-3.33 (m, 2H), 2.50-2.36 (m, 4H), 1.88 (ddt, J = 12.6, 5.1, 2.7, 1H), 1.63-1.55 (m, 1H), 1.40 (s, 9H), 0.07 (s, 9H)); ^{13}C NMR (126 MHz;

$CDCl_3$): δ 171.5, 156.2, 150.1, 125.7, 79.5, 69.0, 36.39, 36.28, 35.2, 32.6, 28.5, 0.3. HRMS-ESI $[C_{17}H_{33}N_2O_4Si]^+$ calcd 357.2209, found: 357.2204.

(*Z*)-tert-butyl-3-oxo-3-(5-((trimethylsilyl)methylene)oxepan-2-ylamino)propyl carbamate (23)

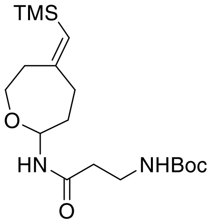

The β-alanine derivative **23** was prepared by general procedure B: (46 mg, 0.2 mmol of *tert*-butyl (3-(allylamino)-3-oxopropyl)carbamate; 28.42 mg, 0.2 mmol of 4-(trimethylsilyl)but-3-yn-1-ol; $[CpRu(MeCN)_3]PF_6$ (2.6 mg, 0.006 mmol); 5 mg, 0.02 mmol diphenylphosphate; 0.8 mL acetone and 1.0 mL toluene). Yield of **23** was 58 mg, 78%. Colorless oil; IR 3267, 2909, 1670, 1515, 1346, 1230, 1155, 1060 cm^{-1}; ^1H NMR (500 MHz; $CDCl_3$): δ 6.43 (s, 1H), 5.33 (s, 1H), 5.21 (m, 2H), 3.93 (dt, J = 12.5, 4.7, 1H), 3.65 (ddd, J = 12.7, 9.3, 3.5, 1H), 3.40-3.36 (m, 2H), 2.55 (ddd, J = 15.0, 9.7, 5.0, 1H), 2.39 (m, 5H), 1.95 (dq, J = 10.3, 3.6, 1H), 1.63 (m, 1H), 1.41 (s, 9H), 0.08 (s, 9H); ^{13}C NMR (126 MHz; $CDCl_3$): δ 171.3, 156.37, 156.26, 127.16, 81.3, 79.5, 66.8, 38.2, 37.3, 36.40, 36.35, 34.6, 28.5, 0.2. MRMS-ESI $[C_{18}H_{35}N_2O_4Si]^+$ calcd. 371.2366, found: 371.2361.

Methyl 2-(*N*-allyl-3-(*tert*-butoxycarbonylamino)propanamido)acetate (24a)[5]

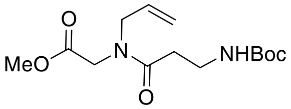

In an oven dried round-bottom flask, Et_3N (1.20 mL, 8.69 mmol) was dissolved in CH_2Cl_2 (5 mL) and cooled to 0 ºC. To this cold mixture, 3-((*tert*-butoxycarbonyl)amino)propanoic acid (822 mg, 4.34 mmol) was added and stirred for 5 min followed by addition of CH_2Cl_2 (*N,N'*-dicyclohexylcarbosiimide) (895.3 mg, 4.34 mmol), HOBt (Hydroxybenzotriazole) (587 mg, 4.34 mmol) and methyl allylglycinate (510 mg, 3.95 mmol). The reaction mixture was stirred for 48 h at room temperature. Solid DCU (Dicyclohexylurea) was filtered off and the eluent was washed successively with 0.5 M HCl (10 mL) and saturated 20 mL $NaHCO_3$. The organic fraction was dried over $MgSO_4$, concentrated and purified by silica gel chromatography. The dipeptide **24a** (1.03 g, 87%) was eluted with 40% EtOAc/Hexane. IR 3300, 2936, 1727, 1686, 1626, 1483, 1418, 1347, 1223, 1158 cm^{-1}; ^1H NMR (500 MHz; DMSO-d_6): Exists as a mixture of rotamers which coalesces at high temperature. 500 MHz; DMSO-d_6, rt: δ 6.68 (m 1H), 5.88-5.64 (m, 1H), 5.20-5.09 (m, 2H), 4.15-3.92 (m, 4H), 3.65 (m, 3H), 3.13 (dt, J = 6.5, 6.2, 2H), 2.51-2.36 (m, 2H), 1.37 (s, 9H); ^{13}C NMR (126 MHz; DMSO-d_6, 90 ºC): δ 170.7, 169.0, 133.3, 132.4, 116.3, 94.5, 77.2, 50.3, 47.0, 36.2, 32.2, 27.6. HRMS-ESI $[C_{14}H_{24}N_2NaO_5]^+$ calcd. 323.1583, found: 323.1577.

[5] Compound **24a** is an intermediate for synthesis of **24** and is not included in the manuscript

(*Z*)-methyl-2-(3-(*tert*-butoxycarbonylamino)-*N*-(5-((trimethylsilyl)methylene)tetrahydro-2*H*-pyran-2-yl)propanamido)acetate (24)

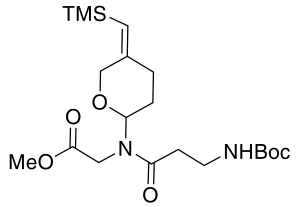

The dipeptide derivative **24** was prepared by general procedure B: (25.64 mg, 0.2 mmol of 3-(trimethylsilyl)prop-2-yn-1-ol; 120 mg, 0.4 mmol of **24a**; [CpRu(MeCN)$_3$]PF$_6$ (8.7 mg, 0.02 mmol); 5 mg, 0.02 mmol diphenylphosphate; 0.8 mL acetone and 1.0 mL toluene). Yield of **24a** was 70 mg, 82%. Colorless solid, MP: 105 ºC; IR 3312, 2913, 2888, 1730, 1689, 1639, 1482, 1417, 1346, 1232, 1156, 1020 cm^{-1}; ^{1}H NMR of **24** at room temperature exists as a mixture of rotamer but at higher temperature the rotamer peaks coalesces; at 80 ºC: ^{1}H NMR (500 MHz; toluene-d$_8$): δ 5.23 (s, 1H), 4.98-4.96 (m, 1H), 4.39 (dd, J = 13.0, 1.8, 1H), 3.84 (d, J = 13.0, 1H), 3.79-3.75 (m, 1H), 3.40-3.33 (m, 6H), 2.36 (s, 2H), 2.25-2.11 (m, 2H), 1.74-1.69 (m, 1H), 1.54-1.46 (m, 1H), 1.39 (s, 9H), 0.05 (s, 9H); ^{13}C NMR (126 MHz; CDCl$_3$), mixture of rotamers: δ 172.6, 170.3, 156.1, 150.1, 149.1, 126.6, 125.9, 84.5, 80.8, 79.3, 70.34, 70.25, 52.6, 52.3, 43.8, 42.9, 36.2, 36.0, 35.72, 35.6, 34.0, 33.5, 31.3, 30.5, 28.53, 28.51, 0.3. HRMS-ESI [$C_{20}H_{36}N_2NaO_6Si$]$^+$ calcd 451.2241, found: 451.2235.

1-((1*E*,4*Z*)-6-hydroxy-4-((trimethylsilyl)methylene)hex-1-en-1-yl)-5-methylpyrimidine- 2,4(1*H*,3*H*)-dione (31)

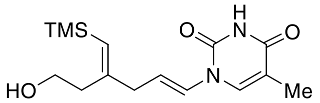

In an oven dried flask, 4-(trimethylsilyl)but-3-yn-1-ol (171 mg, 1.2 mmol), *N*-allyl thymine (200 mg, 1.2 mmol) was dissolved in acetone (3.0 mL, 0.4 M). The content was flushed with argon and [CpRu(MeCN)$_3$]PF$_6$ (26 mg, 0.06 mmol) was added. The reaction mixture was sealed and stirred for 12 h. Solvent was removed under reduced pressure and the product **31** was purified by flash chromatography using 50-70% EtOAc/Hexane, 215 mg, 58%. Colorless solid; MP: 136 ºC; IR 3426, 2913, 1656, 1357, 1229, 825 cm^{-1}; ^{1}H NMR (400 MHz; CDCl$_3$): δ 9.54 (s, 1H), 7.30 (d, J = 1.1, 1H), 6.94-6.90 (m, 1H), 5.59 (dt, J = 14.4, 7.3, 1H), 5.41 (s, 1H), 3.72 (t, J = 6.9, 2H), 2.94 (d, J = 7.4, 2H), 2.44 (t, J = 6.9, 2H), 1.96 (d, J = 1.2, 3H), 0.12 (s, 9H); ^{13}C NMR (101 MHz; CDCl$_3$): δ 163.9, 152.3, 149.6, 136.2, 129.2, 125.3, 117.5, 111.8, 61.3, 39.8, 38.7, 12.6, 0.4; MRMS-ESI [$C_{15}H_{24}N_2NaO_3Si$]$^+$ calcd. 331.1454, found: 331.1462.

1-((1*E*,4*Z*)-6-hydroxy-4-((trimethylsilyl)methylene)hex-1-en-1-yl)pyrimidine-2,4(1*H*,3*H*)-dione (30)

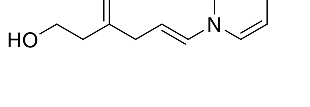

1,4-diene **30** was prepared following the same procedure as for the thiamine derivative **31** using: 4-(trimethylsilyl)but-3-yn-1-ol (95 mg, 0.67 mmol), *N*-allyl uracil (102 mg, 0.67 mmol) and $[CpRu(MeCN)_3]PF_6$ (29 mg, 0.06 mmol) in 2.7 mL acetone. The yield of **30** was found to be 156 mg, 54%. Colorless solid; MP: 152 ºC; IR 3465, 2953, 2888, 1681, 1247, 834, 766, 711 cm^{-1}; ^1H NMR (500 MHz; $CDCl_3$): δ 9.66 (s, 1H), 7.47 (d, J = 8.1, 1H), 6.91 (d, J = 14.3, 1H), 5.81-5.80 (m, 1H), 5.64 (dt, J = 14.4, 7.3, 1H), 5.40 (s, 1H), 3.73 (t, J = 6.8, 2H), 2.95 (d, J = 7.4, 2H), 2.44 (t, J = 6.8, 2H), 1.96-1.91 (m, 1H), 0.12 (s, 9H); ^{13}C NMR (126 MHz; $CDCl_3$): δ 163.4, 152.1, 149.6, 140.5, 129.4, 125.5, 118.8, 103.3, 61.4, 39.7, 38.8, 0.5; MRMS-ESI $[C_{14}H_{22}N_2NaO_3Si]^+$ calcd. 317.1297, found: 317.1300.

(*E*)-(5-methyl-2,4-dioxo-3,4-dihydropyrimidin-1(2*H*)-yl)(4-((trimethylsilyl) methylene)tetrahydro-2 *H*-pyran-2-yl)methyl nitrate (34)

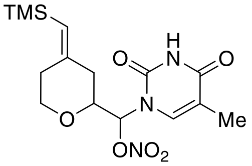

An oven dried microwave vial was charged with ceric ammonium nitrate (CAN), 89 mg, 0.16 mmol. The vial was then flushed with argon and sealed. Then dry CH_3CN (0.65 mL) and EtOH (57 μL) was added. When all CAN was dissolved, the mixture was placed in a –15 ºC bath. After stirring for 5 min at this temperature, **31** (20 mg, 0.065 mmol) was added as a solution in 0.65 mL CH_3CN. After complete addition, –15 ºC bath was replaced with an ice bath was the reaction was allowed to warm to rt over 1h. After consumption of starting material, the reaction was poured in a cold saturated solution of $NaHCO_3$ (10 mL). The product was extracted with EtOAc (3x20 mL), dried over Na_2SO_4, concentrated and purified by flash chromatography using 30% EtOAc/Hex. The product **34** was obtained as white solid, 22 mg, 92% yield as a mixture of diastereomers (7:1). MP: 140 ºC, IR 3161, 1035, 2914, 2815, 1673, 1638; 1442, 1352, 1272, 1233, 1118, 1083, 1041 cm^{-1}; ^1H NMR for major diastereomer: (400 MHz; $CDCl_3$): δ 8.85 (s, 1H), 7.27 (d, J = 1.0, 1H), 6.95 (d, J = 4.9, 1H), 5.36 (s, 1H), 4.16-4.12 (m, 1H), 3.87-3.83 (m, 1H), 3.41-3.34 (m, 1H), 2.37 (dd, J = 13.5, 0.3, 2H), 2.30-2.25 (m, 1H), 2.20-2.17 (m, 1H), 1.94 (s, 3H), 0.09 (s, 9H); ^{13}C NMR for major diastereomer: (101 MHz; $CDCl_3$): δ 163.3, 150.4, 149.3, 135.7, 126.5, 112.1, 83.4, 76.8, 68.9, 41.2, 33.9, 12.8, 0.3; MRMS-ESI $[C_{15}H_{24}N_3O_6Si]^+$ calcd. 370.1434, found: 370.1426.

(*E*)-1-(methoxy(4-((trimethylsilyl)methylene)tetrahydro-2*H*-pyran-2-yl)methyl)-5-methylpyrimidine-2,4(1*H*,3*H*)-dione (35)

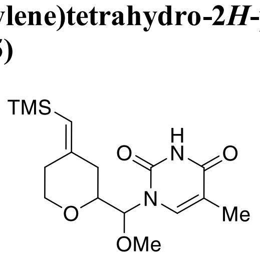

Nitrate **34** (5 mg, 0.02 mmol) was dissolved in 0.2 mL CH_3OH. To this mixture was added 30 μL of 1N NaOH(aq) at rt. The reaction mixture was then stirred for 15 min. Saturated $NaHSO_4$ (1 mL) was used to quench the reaction. The product was extracted with EtOAc (5 mL), dried over Na_2SO_4 and passed through a plug of silica gel to obtain pure **35** in quantitative yield (1:1 mixture of diastereomers). Colorless oil, IR 3154, 2911, 1672, 1443, 1231, 1077, 828 cm^{-1}; ^1H NMR (400 MHz; $CDCl_3$): δ 8.31 (s, 1H), 8.25 (s, 1H), 7.32-7.31 (m, 1H), 7.21-7.20 (m, 1H), 5.52 (d, J = 6.0, 1H), 5.46 (d, J = 3.6, 1H), 5.32 (s, 1H), 5.29 (s, 1H),

4.19-4.15 (m, 1H), 4.12-4.07 (m, 1H), 3.55-3.46 (m, 2H), 3.40 (s, 3H), 3.36 (d, J = 4.7, 3H), 3.35-3.29 (m, 2H), 2.49-2.42 (m, 1H), 2.39-2.31 (m, 3H), 2.30-2.25 (m, 2H), 2.19-2.09 (m, 2H), 1.94 (d, J = 1.2, 3H), 1.90 (d, J = 1.2, 3H), 0.091 (s, 9H), 0.088 (s, 9H); ^{13}C NMR (101 MHz; $CDCl_3$): δ 163.5, 163.2, 151.16, 151.05, 136.3, 135.8, 125.16, 125.08, 111.30, 111.21, 86.91, 86.81, 79.4, 78.7, 69.2, 68.9, 57.7, 57.1, 41.5, 41.0, 34.26, 34.20, 12.80, 12.77, 0.3; MRMS-ESI $[C_{16}H_{27}N_2O_4Si]^+$ calcd. 339.17401, found: 339.17397.

General procedure for Pd-catalyzed iodo-hemi-aminal formation:

1, 4-diene (0.1 mmol) was dissolved in Dry THF (0.75 mL). In a separate flask, Cp(allyl)Pd (0.01 mmol) and PPh_3 (0.02 mmol) was taken and flushed with Ar. Dry THF (0.25 mL) was then added and stirred for 15-30 min. This catalyst mixture was the added to the substrate solution followed by addition of NIS or NBS (0.11 mmol) in appropriate solvent. The reaction mixture was then stirred at room temperature for 0.5–1h. Solvent was removed under pressure and the crude reaction mixture was subjected to silica gel chromatography.

1-((2*S*,3*S*,*E*)-3-iodo-5-((trimethylsilyl)methylene)oxepan-2-yl)-5-methylpyrimidine-2,4(1*H*,3*H*)-dione (36)

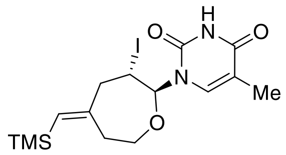

The cyclized product **36** was synthesized following general procedure for Pd-catalyzed iodo-hemi-aminal formation using: 31 mg, 0.1 mmol of **31** , Cp(allyl)Pd (2.2 mg, 0.01 mmol), PPh_3 (5.3 mg, 0.02 mmol) and NIS (25 mg, 0.11 mmol) in 1 mL THF. After purification, 40 mg, 91% of the desired product **36** was obtained as waxy solid: IR 2952, 1713, 1661, 1468, 1243, 1133, 1085, 834, 750 cm^{-1}; ^{1}H NMR (400 MHz; $CDCl_3$): δ 8.26 (s, 1H), 6.96 (d, J = 1.2, 1H), 5.86 (d, J = 10.3, 1H), 5.63 (s, 1H), 4.16 (td, J = 10.0, 4.4, 1H), 4.11-4.05 (m, 1H), 3.82 (ddd, J = 12.5, 10.1, 4.9, 1H), 3.15-3.10 (m, 1H), 3.03 (dd, J = 13.6, 9.7, 1H), 2.77-2.69 (m, 1H), 2.49-2.43 (m, 1H), 1.93 (d, J = 1.2, 3H), 0.12 (s, 9H); ^{13}C NMR (101 MHz; $CDCl_3$): δ 163.2, 151.5, 150.2, 134.4, 132.9, 112.0, 89.5, 69.5, 50.4, 36.4, 29.9, 12.8, 0.04; MRMS-ESI $[C_{15}H_{23}IN_2NaO_3Si]^+$ calcd 457.0420, found: 457.0400.

1-((2*S*,3*S*,*Z*)-3-iodo-5-((trimethylsilyl)methylene)tetrahydro-2H-pyran-2-yl)pyrimidine- 2,4(1*H*,3*H*)-dione (37)

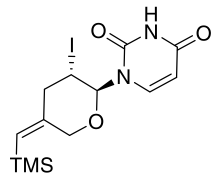

The product **37** was synthesized following general procedure for Pd-catalyzed iodo-hemi-aminal formation using: 28 mg, 0.1 mmol of **29**, Cp(allyl)Pd (2.2 mg, 0.01 mmol), PPh_3 (5.3 mg, 0.02 mmol) and NIS (25 mg, 0.11 mmol) in 1 mL THF. After purification, 39 mg, 96% of the desired product **37** was obtained as a waxy solid: IR 3308, 2953, 1743, 1507, 1392, 1366, 1248, 1159, 1081, 839, 720 cm^{-1}; ^{1}H NMR (500 MHz;

$CDCl_3$): δ 8.27 (s, 1H), 7.21 (d, J = 8.1, 1H), 5.89 (d, J = 10.4, 1H), 5.79 (dd, J = 8.1, 2.3, 1H), 5.49 (s, 1H), 4.60 (dd, J = 12.9, 0.9, 1H), 4.23 (d, J = 12.9, 1H), 4.13-4.06 (m, 1H), 3.14-3.12 (m, 2H), 0.13 (s, 9H); ^{13}C NMR (126 MHz; $CDCl_3$): δ 162.3, 150.0, 148.0, 138.7, 129.1, 103.7, 84.8, 70.9, 49.0, 24.6, 0.2; MRMS-ESI $[C_{13}H_{19}IN_2NaO_3Si]^+$ calcd. 429.0107, found: 429.0105.

1-((2*S*,3*S*,*E*)-3-iodo-5-((trimethylsilyl)methylene)oxepan-2-yl)pyrimidine-2,4(1*H*,3*H*)-dione (38)

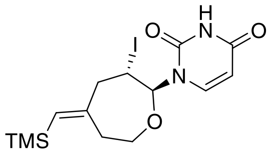

The cyclized product **38** was synthesized following general procedure for Pd-catalyzed iodo-hemi-aminal formation using: 29.5 mg, 0.1 mmol of **30**, Cp(allyl)Pd (2.2 mg, 0.01 mmol), PPh_3 (5.3 mg, 0.02 mmol) and NIS (25 mg, 0.11 mmol) in 1 mL THF. After purification, 34 mg, 82% of the desired product **38** was obtained as a waxy solid: IR 33030, 2953, 1716, 1668, 1256, 1084, 833, 749, 596 cm^{-1}; ^1H NMR (500 MHz; $CDCl_3$): δ 8.39 (s, 1H), 7.18 (d, J = 8.2, 1H), 5.89-5.87 (m, 1H), 5.78 (d, J = 8.2, 1H), 5.65 (s, 1H), 4.17-4.09 (m, 2H), 3.87-3.82 (m, 1H), 3.16-3.12 (m, 1H), 3.07-3.02 (m, 1H), 2.78-2.72 (m, 1H), 2.50-2.45 (m, 1H), 0.14 (s, 9H), ^{13}C NMR (126 MHz; $CDCl_3$): δ 162.5, 151.5, 150.0, 138.9, 133.0, 103.5, 89.6, 69.5, 50.2, 36.4, 29.6, 0.02; MRMS-ESI $[C_{14}H_{21}IN_2NaO_3Si]^+$ calcd. 443.0263, found: 443.0259.

1-((2*S*,3*S*,*E*)-3-bromo-5-((trimethylsilyl)methylene)oxepan-2-yl)-5-methylpyrimidine- 2,4(1*H*,3*H*)-dione (39)

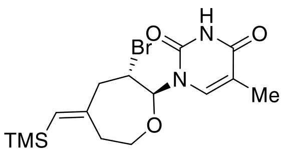

The product **39** was synthesized following general procedure for Pd-catalyzed iodo-hemi-aminal formation using: 31 mg, 0.1 mmol of **31**, Cp(allyl)Pd (2.2 mg, 0.01 mmol), PPh_3 (5.3 mg, 0.02 mmol) and NBS (20 mg, 0.11 mmol) in 1 mL THF. After purification, 33 mg, 84% of the desired product **39** was obtained as waxy solid: IR 2952, 1714, 1666, 1467, 1244, 1085, 838, 763, 596 cm^{-1}; ^1H NMR (500 MHz; $CDCl_3$): δ 8.30 (s, 1H), 7.02 (d, J = 1.2, 1H), 5.82 (d, J = 9.9, 1H), 5.68 (s, 1H), 4.15-4.05 (m, 2H), 3.86 (ddd, J = 12.4, 10.0, 4.9, 1H), 3.06 (dd, J = 13.8, 4.3, 1H), 2.94 (dd, J = 13.6, 9.4, 1H), 2.73 (ddd, J = 15.9, 9.7, 6.5, 1H), 2.48 (dddd, J = 15.5, 4.8, 3.5, 1.3, 1H), 1.94 (d, J = 1.2, 3H), 0.14 (s, 9H); ^{13}C NMR (126 MHz; $CDCl_3$): δ 163.2, 150.3, 149.7, 134.7, 133.5, 111.9, 88.5, 69.9, 50.6, 48.6, 36.3, 12.7, 0.1; MRMS-ESI $[C_{15}H_{23}BrN_2NaO_3Si]^+$ calcd. 409.0559, found: 409.0555.

(*S*,*E*)-5-methyl-1-(5-((trimethylsilyl)methylene)-2,5,6,7-tetrahydrooxepin-2-yl)pyrimidine-2,4(1*H*,3*H*)-dione (40)

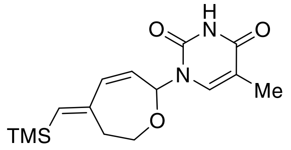

Iodide **36** (10 mg, 0.023 mmol) was dissolved in dry THF. To this was added KO^{t}Bu (5.2 mg, 0.46 mmol) and stirred at rt for 10 min. The reaction mixture was then quenched with 0.5M cold $NaHSO_4$ (1 mL) and the aqueous layer was extracted with Et_2O (10 mL). Pure **40** (5.2 mg, 75%) was obtained by preparative TLC. Colorless solid; MP: 140 ºC; IR 3359, 1686, 1442, 1231, 1095, 844 cm^{-1}; ^{1}H NMR (500 MHz; $CDCl_3$): δ 8.29 (s, 1H), 7.18 (s, 1H), 6.48 (s, 1H), 6.38 (ddt, J = 12.3, 0.6, 0.4, 1H), 5.81 (s, 1H), 5.32-5.29 (m, 1H), 4.00-3.95 (m, 1H), 3.87-3.82 (m, 1H), 2.85-2.75 (m, 2H), 1.92 (s, 3H), 0.16 (s, 9H); ^{13}C NMR (126 MHz; $CDCl_3$): δ 163.6, 150.24, 140.6, 138.5, 136.8, 125.8, 111.5, 95.7, 82.2, 66.5, 36.5, 12.6, -0.1; HRMS-ESI $[C_{15}H_{22}N_2NaO_3Si]^+$ calcd. 329.1297, found: 329.1296.

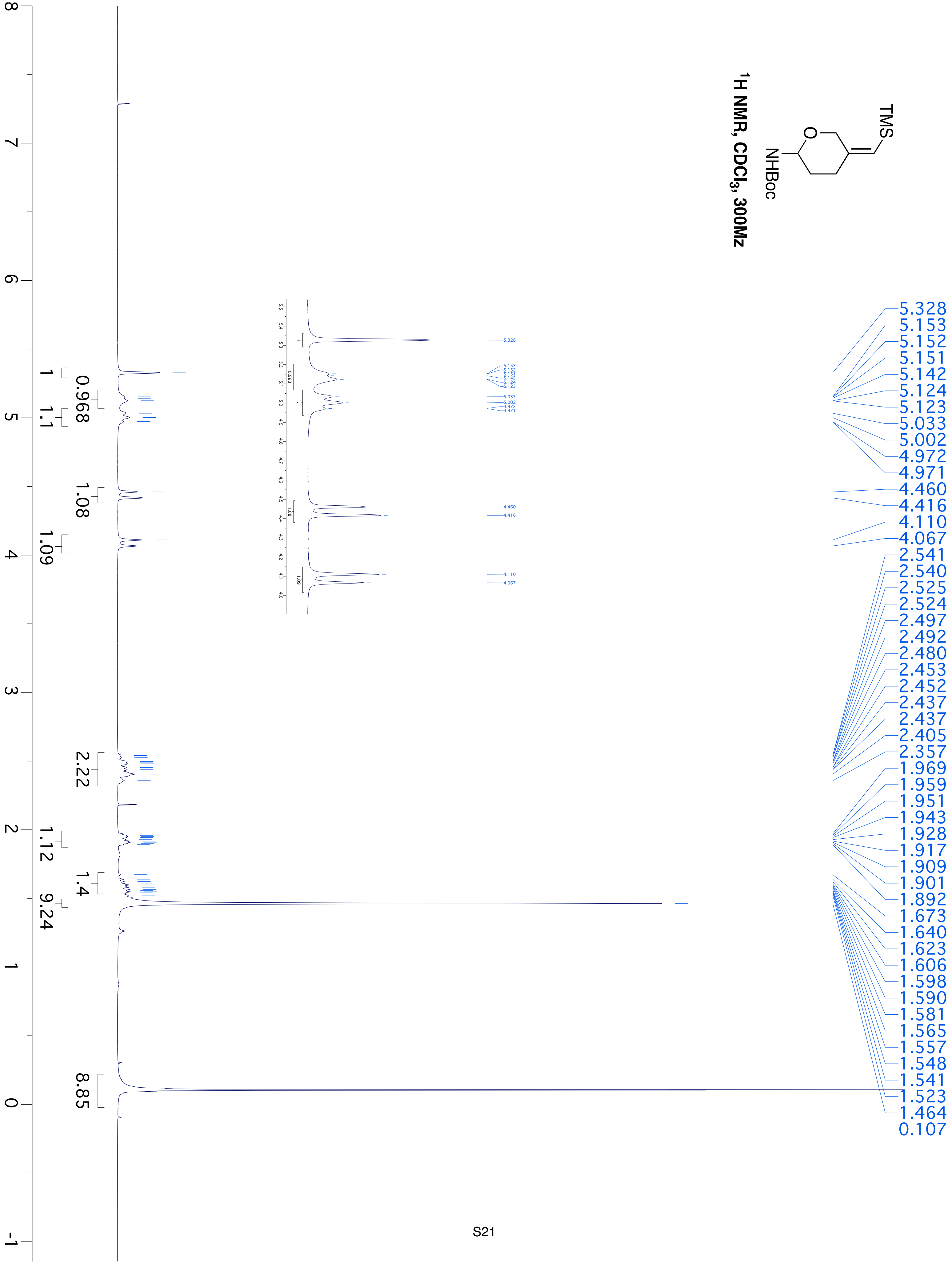
TMS
O
NHBoc
1H NMR, CDCl3, 300Mz
5.328
5.153
5.152
5.151
5.142
5.124
5.123
5.033
5.002
4.972
4.971
4.460
4.416
4.110
4.067
2.541
2.540
2.525
2.524
2.497
2.492
2.480
2.453
2.452
2.437
2.437
2.405
2.357
1.969
1.959
1.951
1.943
1.928
1.917
1.909
1.901
1.892
1.673
1.640
1.623
1.606
1.598
1.590
1.581
1.565
1.557
1.548
1.541
1.523
1.464
0.107
1
0.968
1.1
1.08
1.09
2.22
1.12
1.4
9.24
8.85

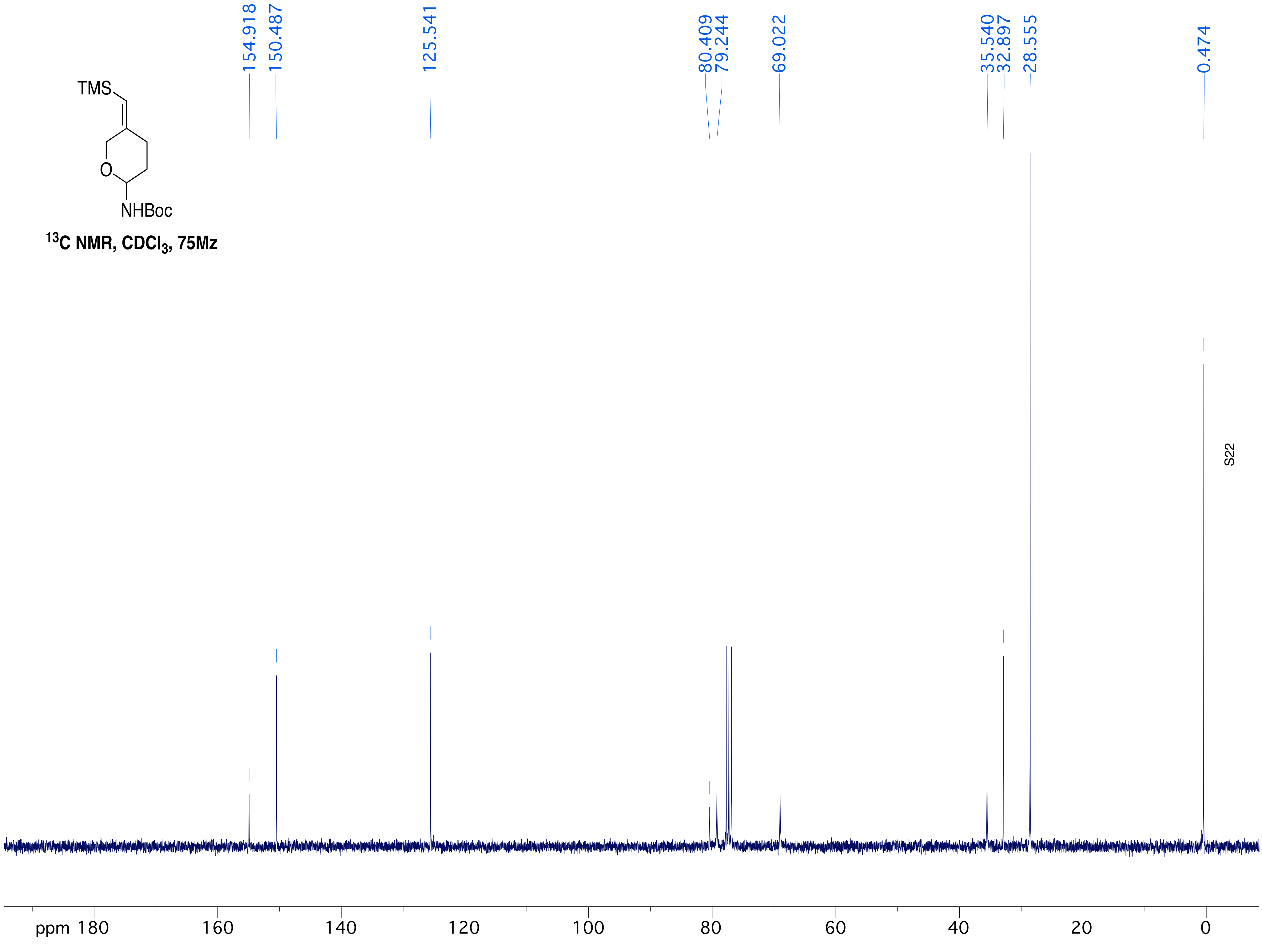

^{13}C NMR, $CDCl_3$, 75Mz

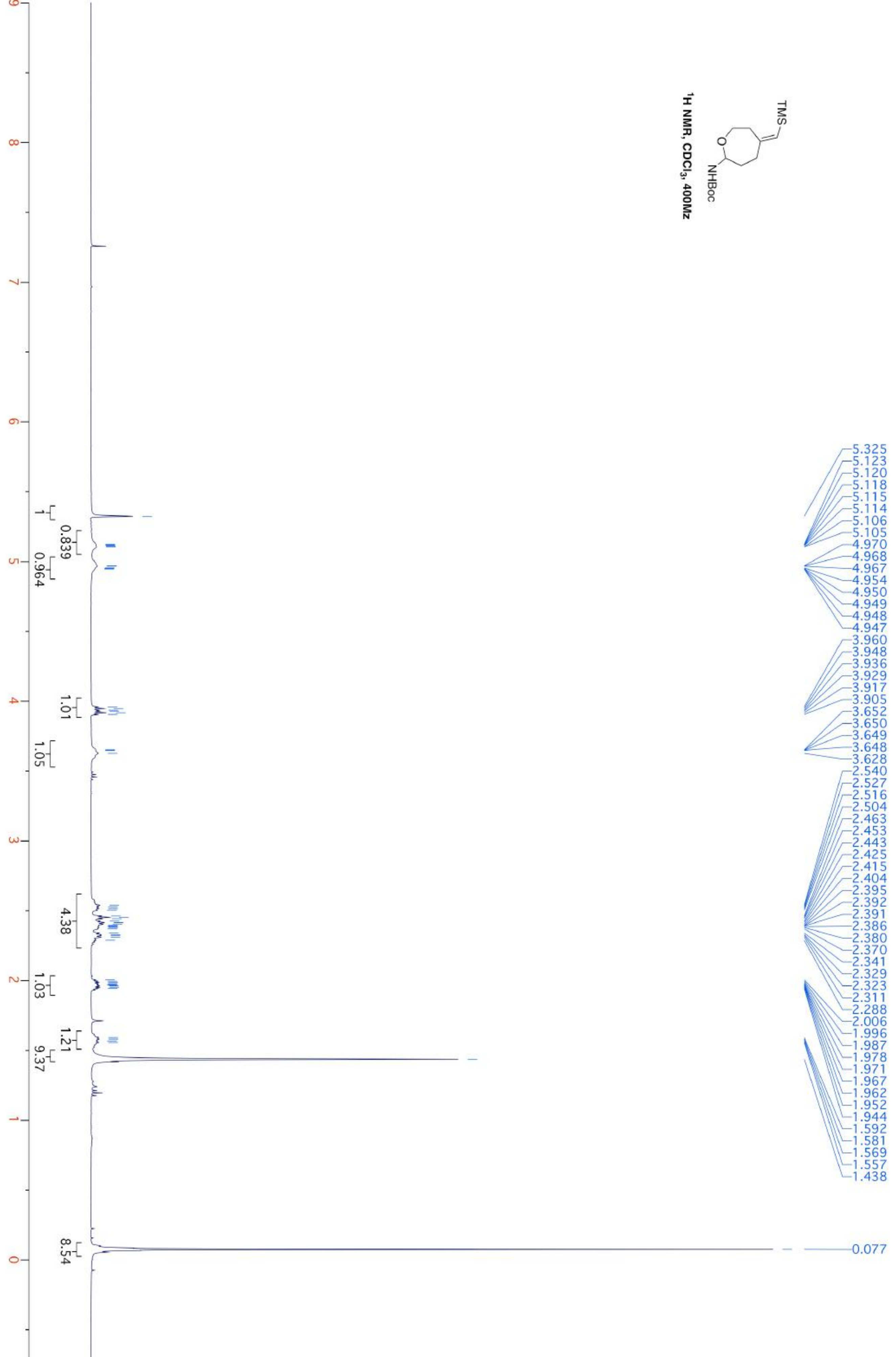
TMS
O
NHBoc
1H NMR, CDCl3, 400Mz
5.325
5.123
5.120
5.118
5.115
5.114
5.106
5.105
4.970
4.968
4.967
4.954
4.950
4.949
4.948
4.947
3.960
3.948
3.936
3.929
3.917
3.905
3.652
3.650
3.649
3.648
3.628
2.540
2.527
2.516
2.504
2.463
2.453
2.443
2.425
2.415
2.404
2.395
2.392
2.391
2.386
2.380
2.370
2.341
2.329
2.323
2.311
2.288
2.006
1.996
1.987
1.978
1.971
1.967
1.962
1.952
1.944
1.592
1.581
1.569
1.557
1.438
0.077
1
0.839
0.964
1.01
1.05
4.38
1.03
1.21
9.37
8.54
9
8
7
6
5
4
3
2
1
0
-1

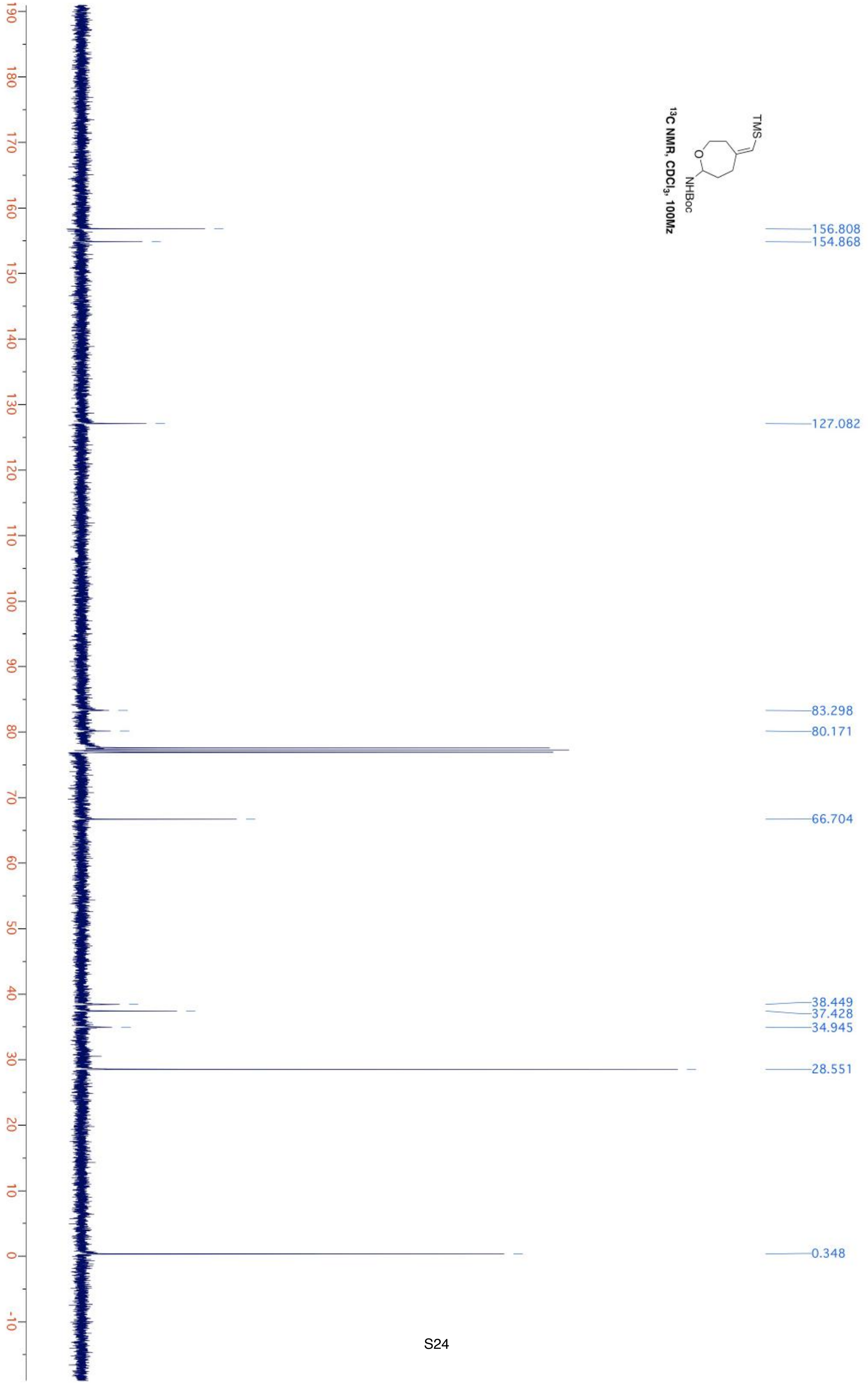
TMS
O
NHBoc
^{13}C NMR, CDCl$_3$, 100Mz
156.808
154.868
127.082
83.298
80.171
66.704
38.449
37.428
34.945
28.551
0.348
190
180
170
160
150
140
130
120
110
100
90
80
70
60
50
40
30
20
10
0
-10

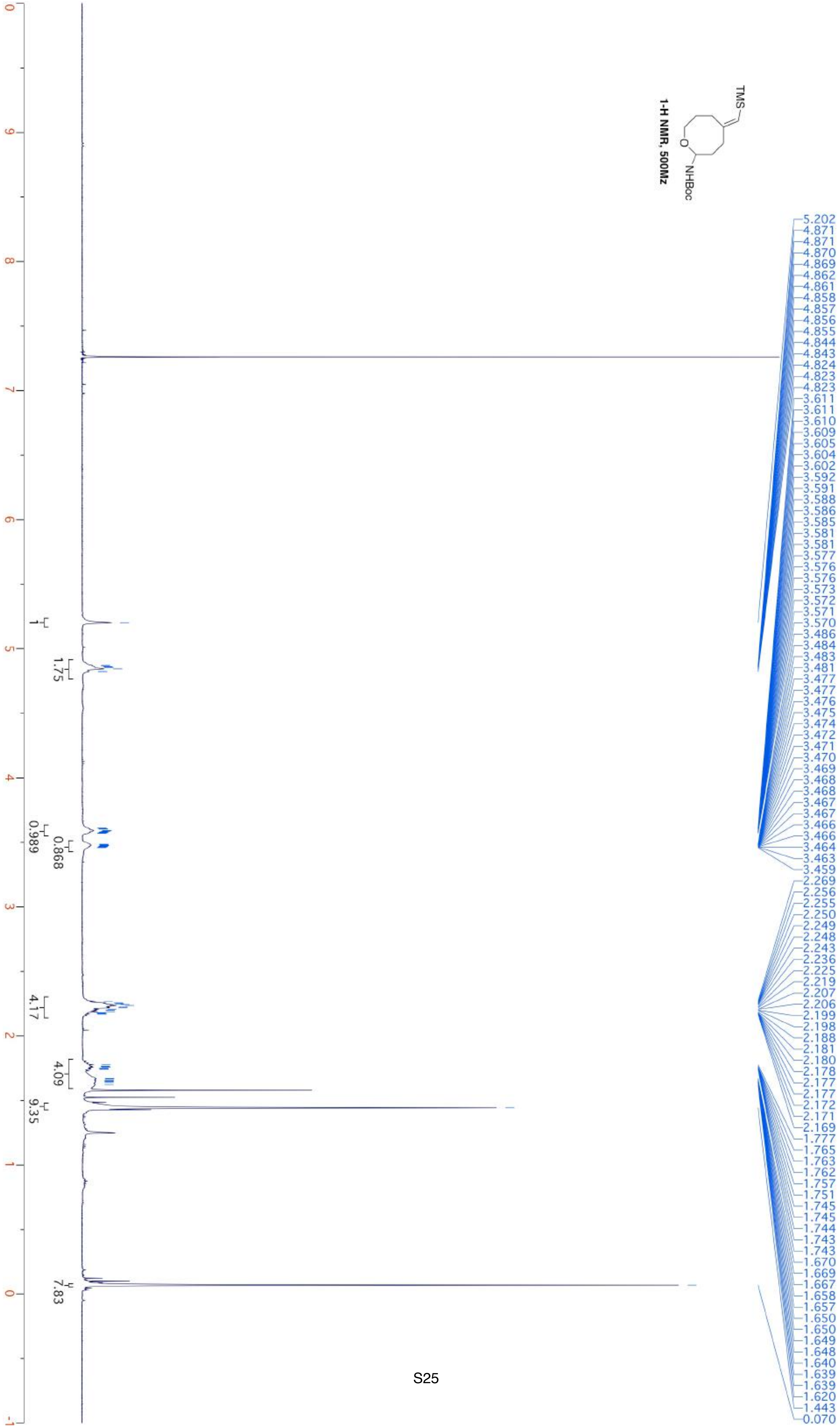
TMS
O
NHBoc
1-H NMR, 500Mz
5.202
4.871
4.871
4.870
4.869
4.862
4.861
4.858
4.857
4.856
4.855
4.844
4.843
4.824
4.823
4.823
3.611
3.611
3.610
3.609
3.605
3.604
3.602
3.592
3.591
3.588
3.586
3.585
3.581
3.581
3.577
3.576
3.576
3.573
3.572
3.571
3.570
3.486
3.484
3.483
3.481
3.477
3.477
3.476
3.475
3.474
3.472
3.471
3.470
3.469
3.468
3.468
3.467
3.467
3.466
3.466
3.464
3.463
3.459
2.269
2.256
2.255
2.250
2.249
2.248
2.243
2.236
2.225
2.219
2.207
2.206
2.199
2.198
2.188
2.181
2.180
2.178
2.177
2.177
2.172
2.171
2.169
1.777
1.765
1.763
1.762
1.757
1.751
1.745
1.745
1.744
1.743
1.743
1.670
1.669
1.667
1.658
1.657
1.650
1.650
1.649
1.648
1.640
1.639
1.639
1.620
1.443
0.070
1
1.75
0.989
0.868
4.17
4.09
9.35
7.83

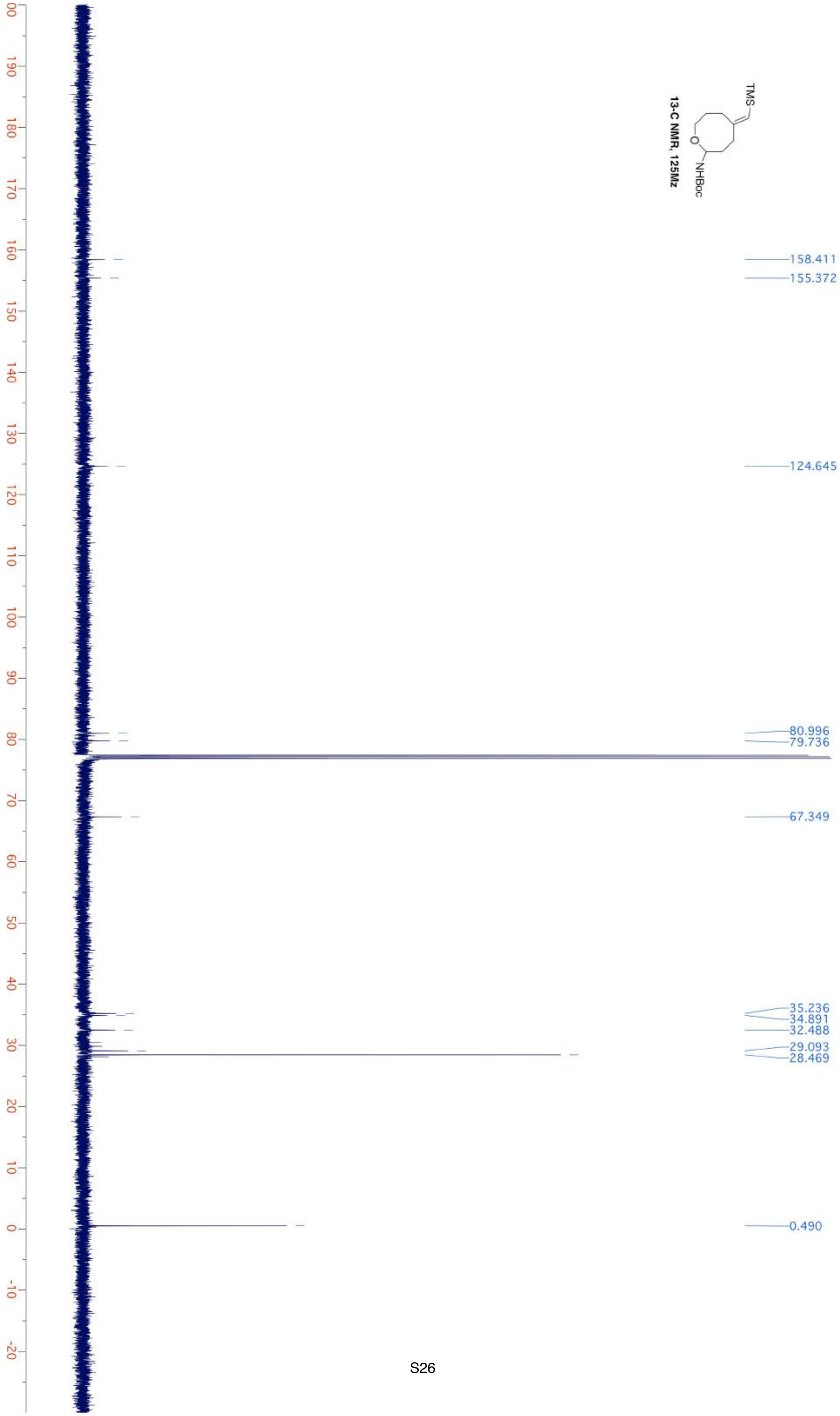
TMS
NHBoc
O
13-C NMR, 125Mz
158.411
155.372
124.645
80.996
79.736
67.349
35.236
34.891
32.488
29.093
28.469
0.490
200
190
180
170
160
150
140
130
120
110
100
90
80
70
60
50
40
30
20
10
0
-10
-20

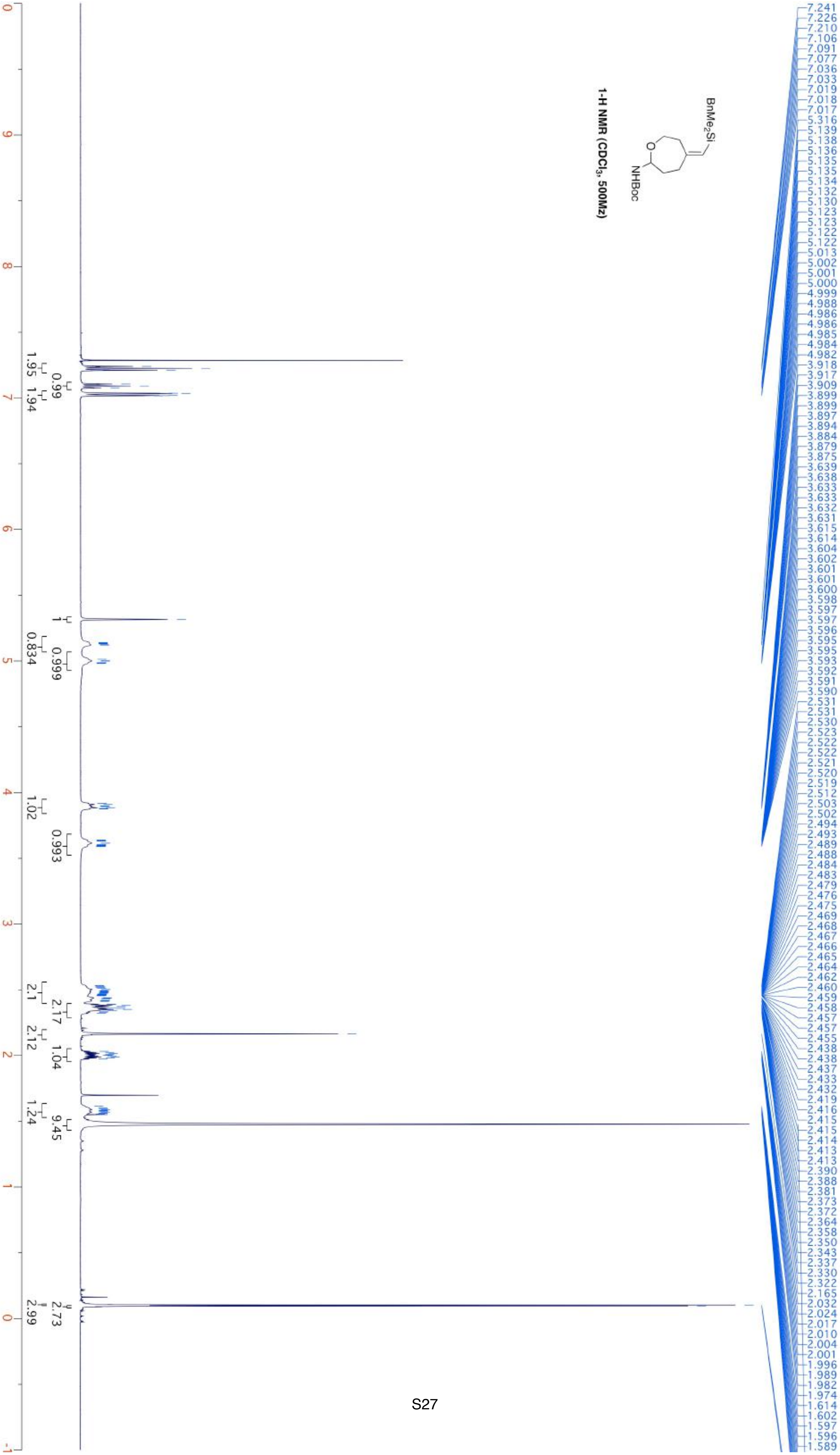
1-H NMR (CDCl3, 500Mz)
BnMe2Si
O
NHBoc
7.241
7.226
7.210
7.106
7.091
7.077
7.036
7.033
7.019
7.018
7.017
5.316
5.139
5.138
5.136
5.135
5.135
5.134
5.132
5.130
5.123
5.123
5.122
5.122
5.013
5.002
5.001
5.000
4.999
4.988
4.986
4.986
4.985
4.984
4.982
3.918
3.917
3.909
3.899
3.899
3.897
3.894
3.884
3.879
3.875
3.639
3.638
3.633
3.633
3.632
3.631
3.615
3.614
3.604
3.602
3.601
3.601
3.600
3.598
3.597
3.597
3.596
3.595
3.595
3.593
3.592
3.591
3.590
2.531
2.531
2.530
2.523
2.522
2.522
2.521
2.520
2.519
2.512
2.503
2.502
2.494
2.493
2.489
2.488
2.484
2.483
2.479
2.476
2.475
2.469
2.468
2.467
2.466
2.465
2.464
2.462
2.460
2.459
2.458
2.457
2.457
2.455
2.438
2.438
2.437
2.433
2.432
2.419
2.416
2.415
2.415
2.414
2.413
2.413
2.390
2.388
2.381
2.373
2.372
2.364
2.358
2.350
2.343
2.337
2.330
2.322
2.165
2.032
2.024
2.017
2.010
2.004
2.001
1.996
1.989
1.982
1.974
1.614
1.602
1.597
1.596
1.589

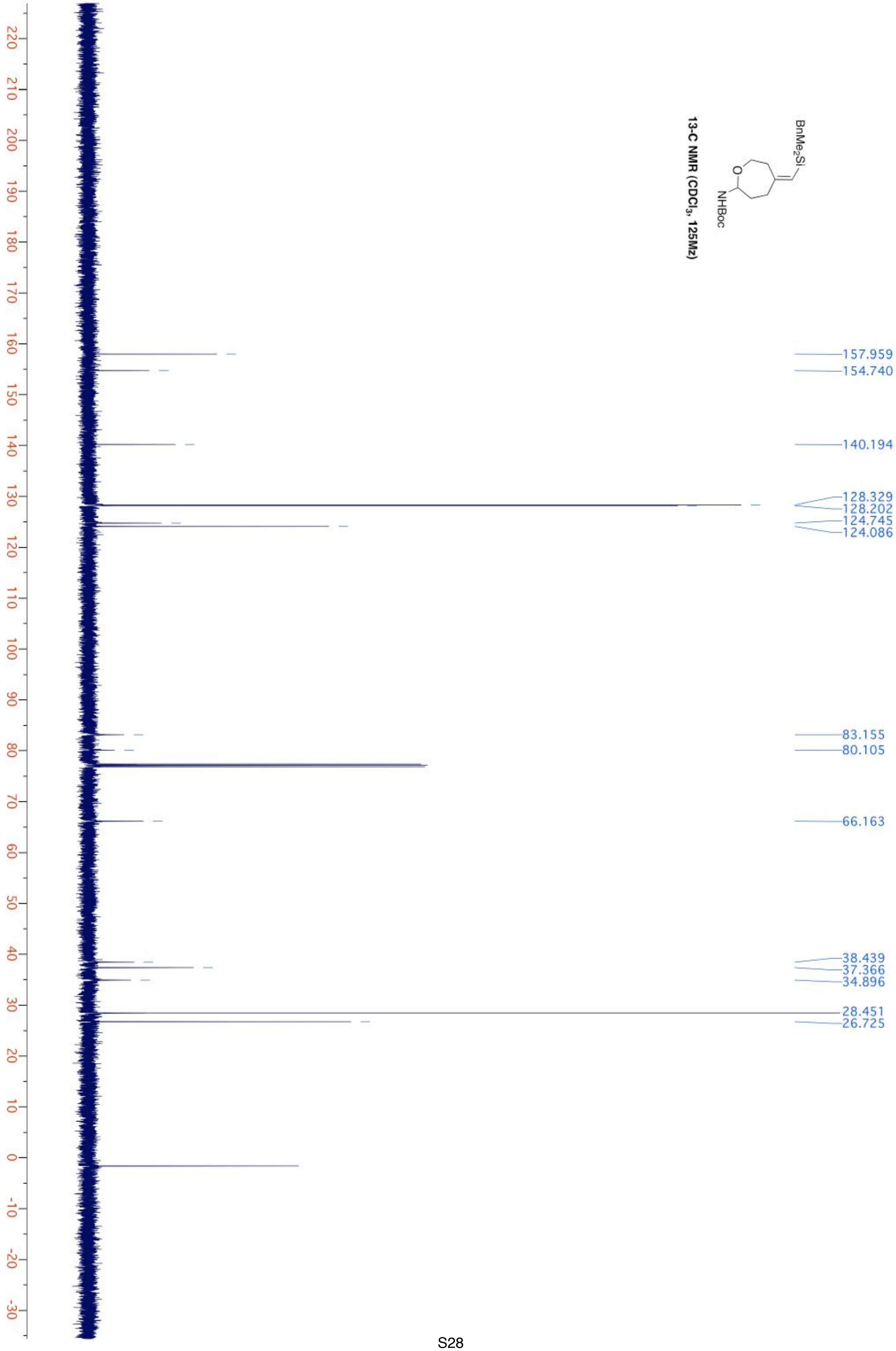
BnMe2Si
O
NHBoc
13-C NMR (CDCl3, 125Mz)
157.959
154.740
140.194
128.329
128.202
124.745
124.086
83.155
80.105
66.163
38.439
37.366
34.896
28.451
26.725
220
210
200
190
180
170
160
150
140
130
120
110
100
90
80
70
60
50
40
30
20
10
0
-10
-20
-30

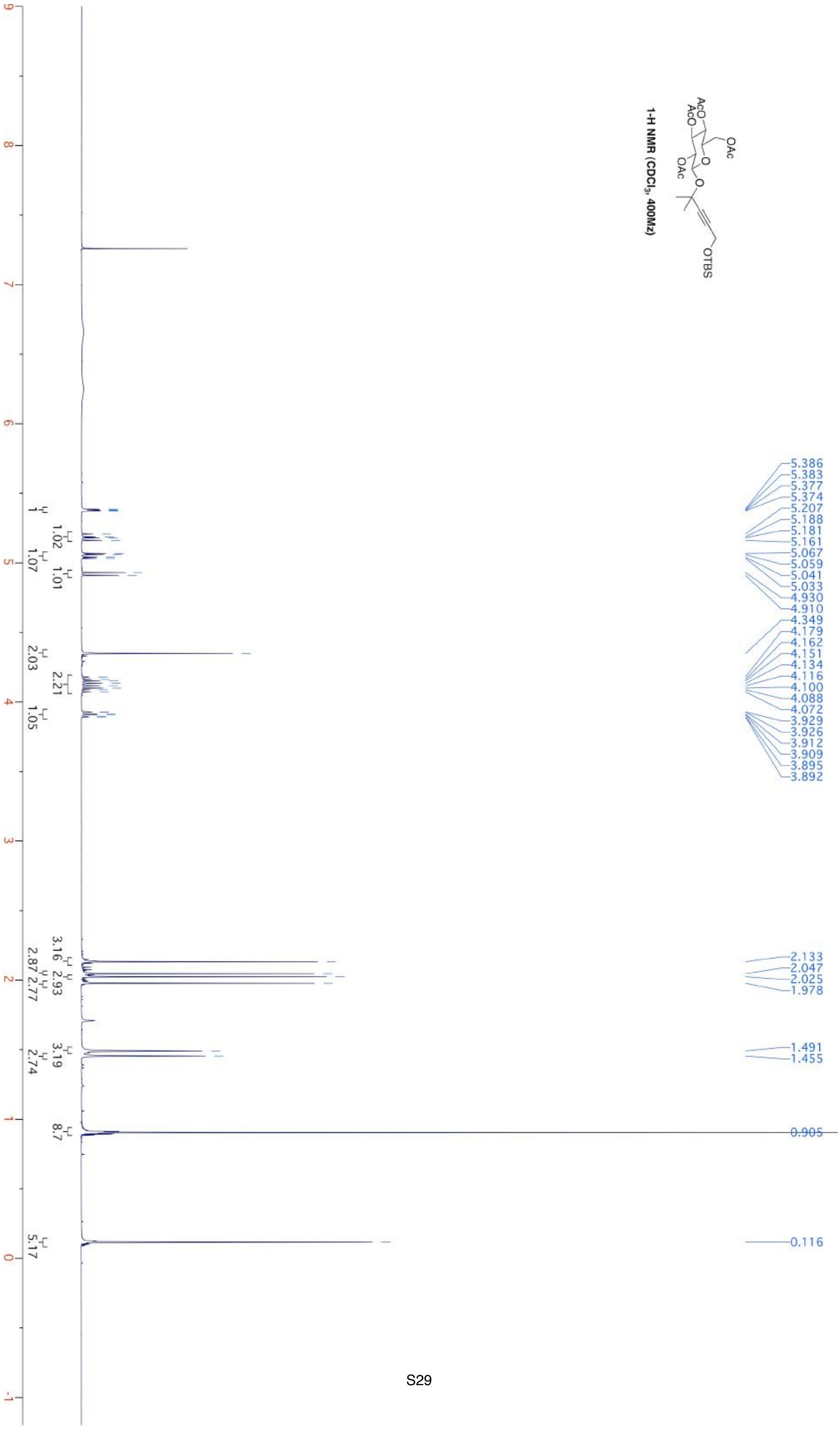
1-H NMR (CDCl3, 400Mz)
AcO
AcO
OAc
OAc
O
O
OTBS
5.386
5.383
5.377
5.374
5.207
5.188
5.181
5.161
5.067
5.059
5.041
5.033
4.930
4.910
4.349
4.179
4.162
4.151
4.134
4.116
4.100
4.088
4.072
3.929
3.926
3.912
3.909
3.895
3.892
2.133
2.047
2.025
1.978
1.491
1.455
0.905
0.116
1
1.02
1.07
1.01
2.03
2.21
1.05
3.16
2.87
2.93
2.77
3.19
2.74
8.7
5.17
9
8
7
6
5
4
3
2
1
0
-1

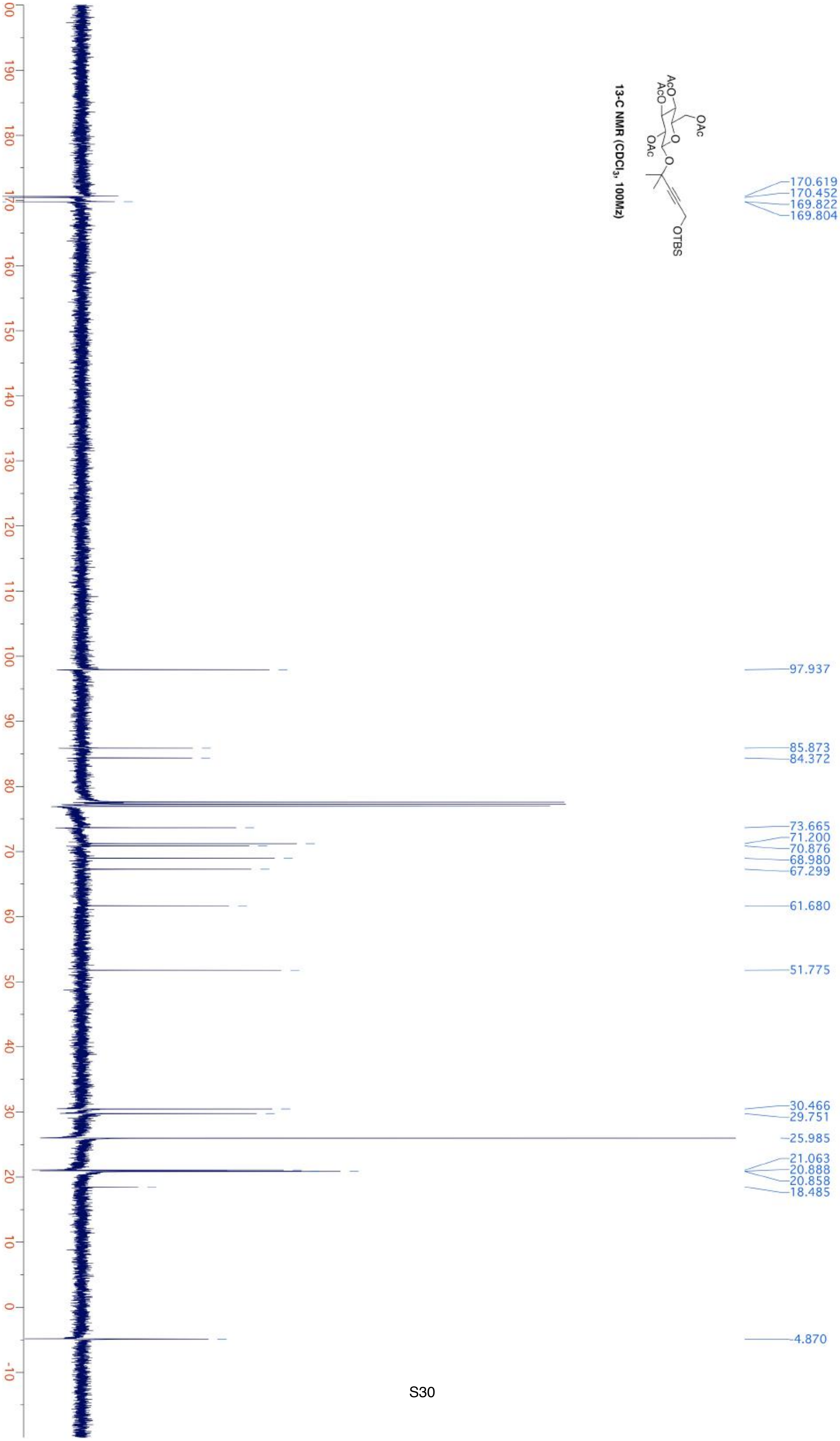
13-C NMR (CDCl3, 100Mz)
AcO
AcO
AcO
OAc
OAc
O
O
OTBS
170.619
170.452
169.822
169.804
97.937
85.873
84.372
73.665
71.200
70.876
68.980
67.299
61.680
51.775
30.466
29.751
25.985
21.063
20.888
20.858
18.485
-4.870

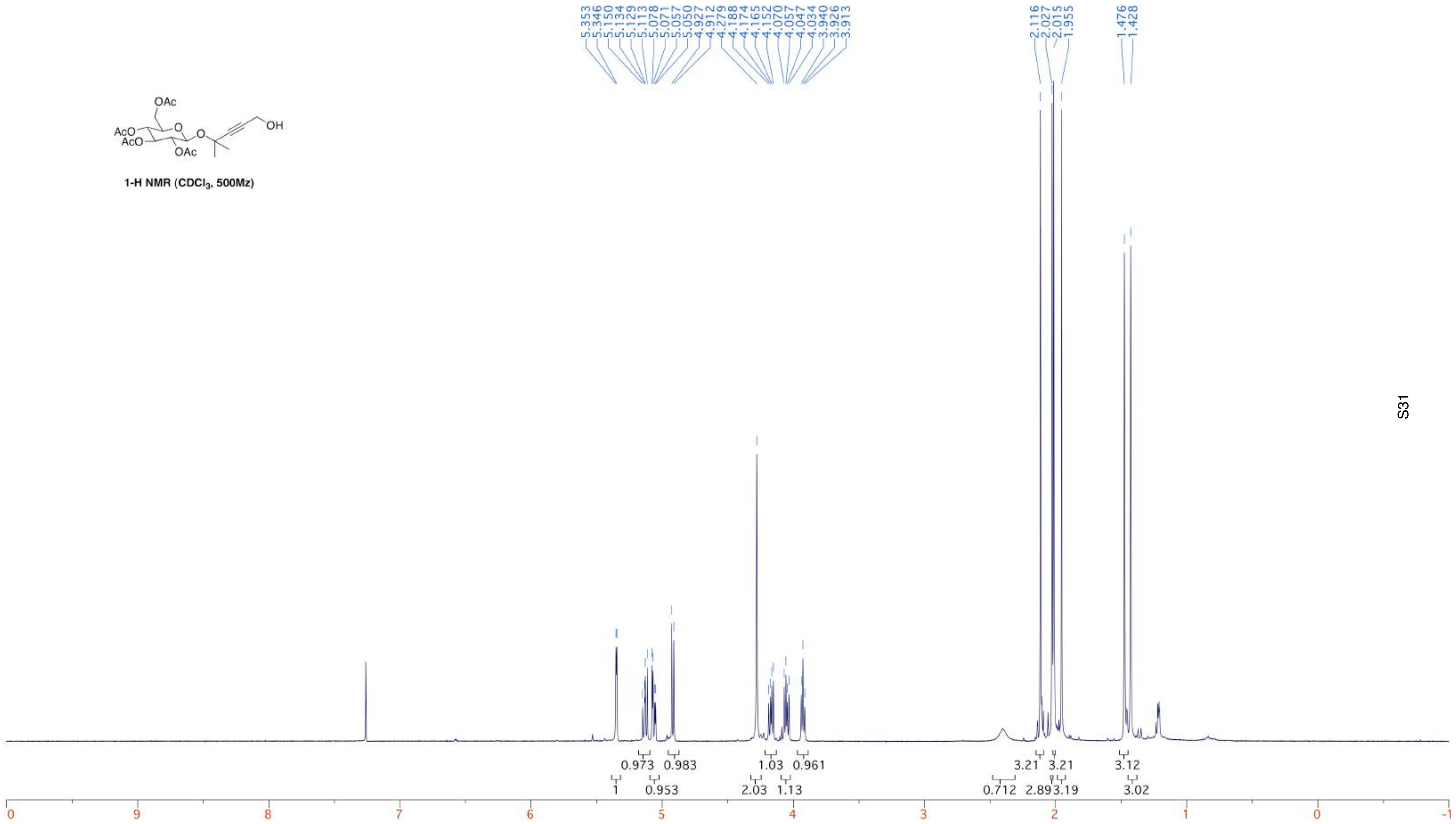

1-H NMR (CDCl$_3$, 500Mz)

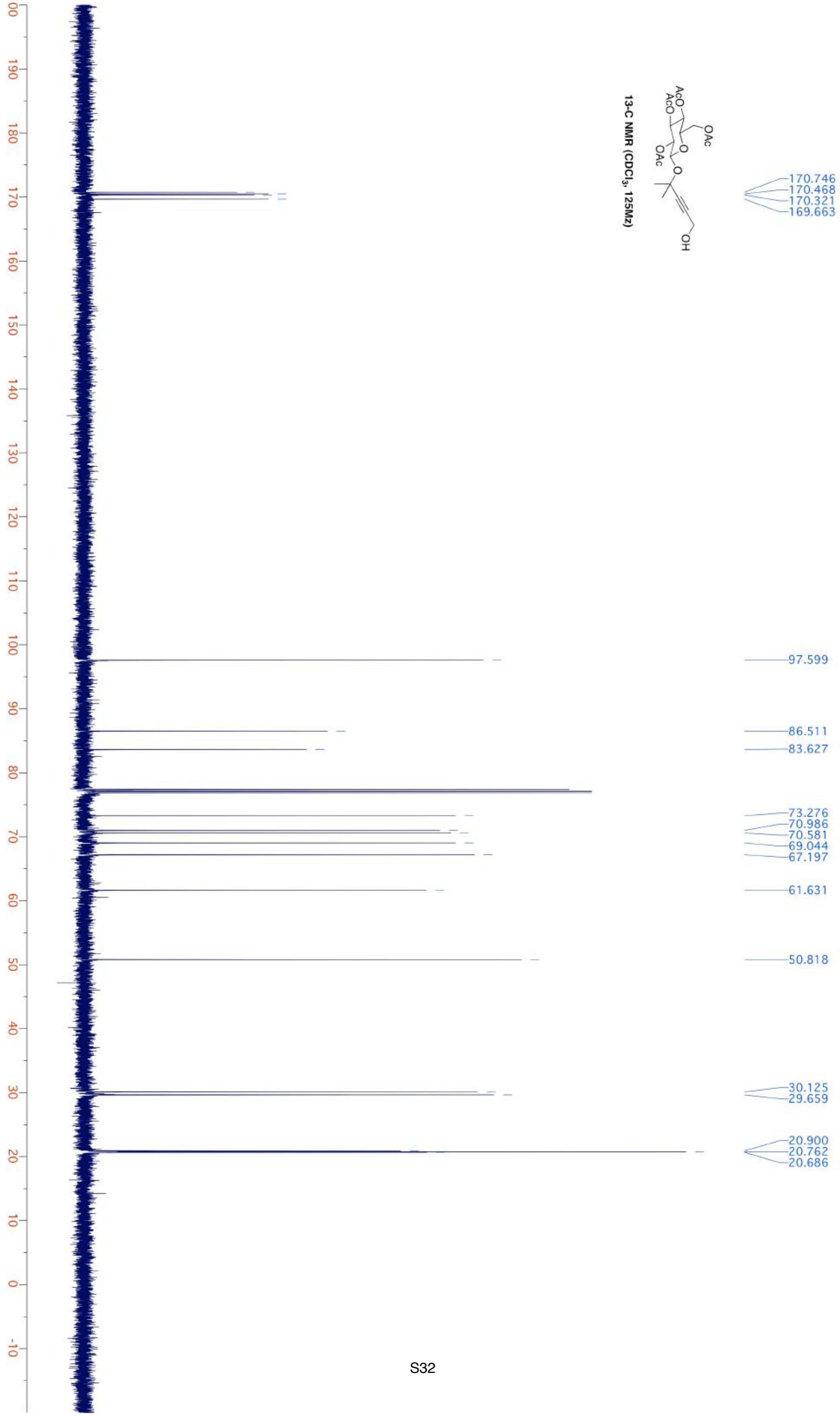
13-C NMR (CDCl3, 125Mz)
AcO
AcO
OAc
OAc
O
O
OH
170.746
170.468
170.321
169.663
97.599
86.511
83.627
73.276
70.986
70.581
69.044
67.197
61.631
50.818
30.125
29.659
20.900
20.762
20.686

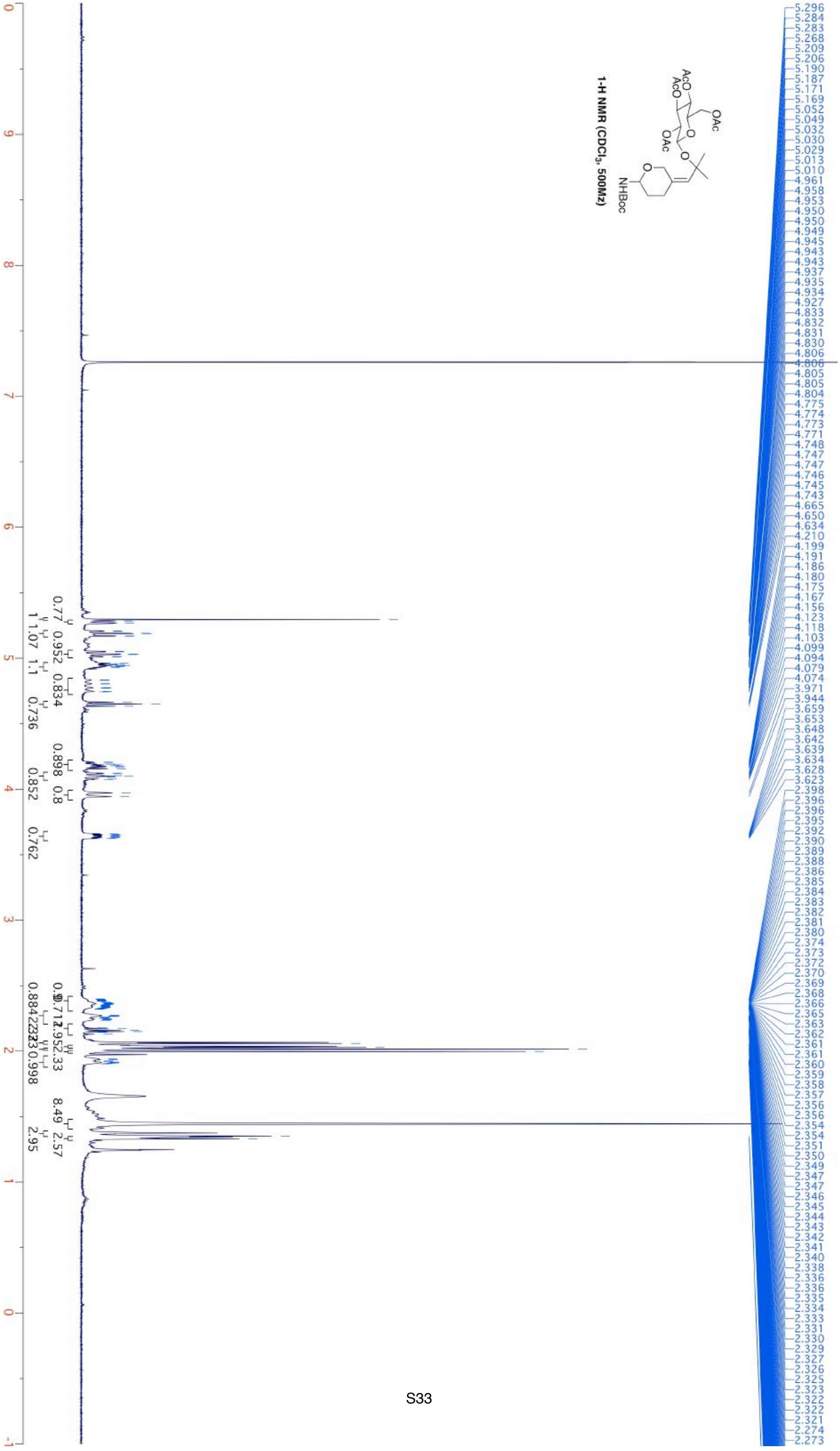
1-H NMR (CDCl3, 500Mz)
NHBoc

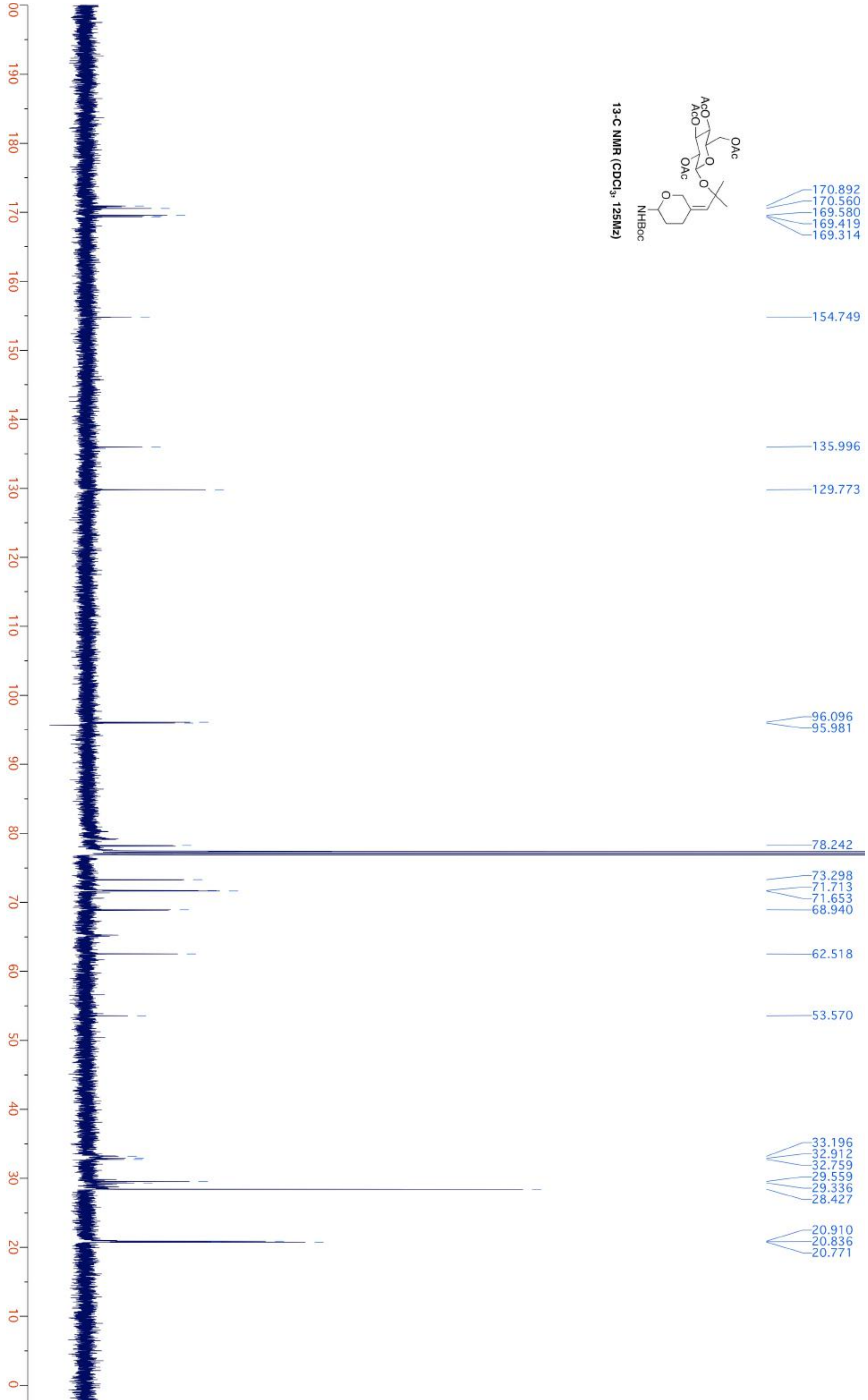
13-C NMR (CDCl3, 125Mz)
AcO
AcO
OAc
OAc
NHBoc
170.892
170.560
169.580
169.419
169.314
154.749
135.996
129.773
96.096
95.981
78.242
73.298
71.713
71.653
68.940
62.518
53.570
33.196
32.912
32.759
29.559
29.336
28.427
20.910
20.836
20.771
200
190
180
170
160
150
140
130
120
110
100
90
80
70
60
50
40
30
20
10
0

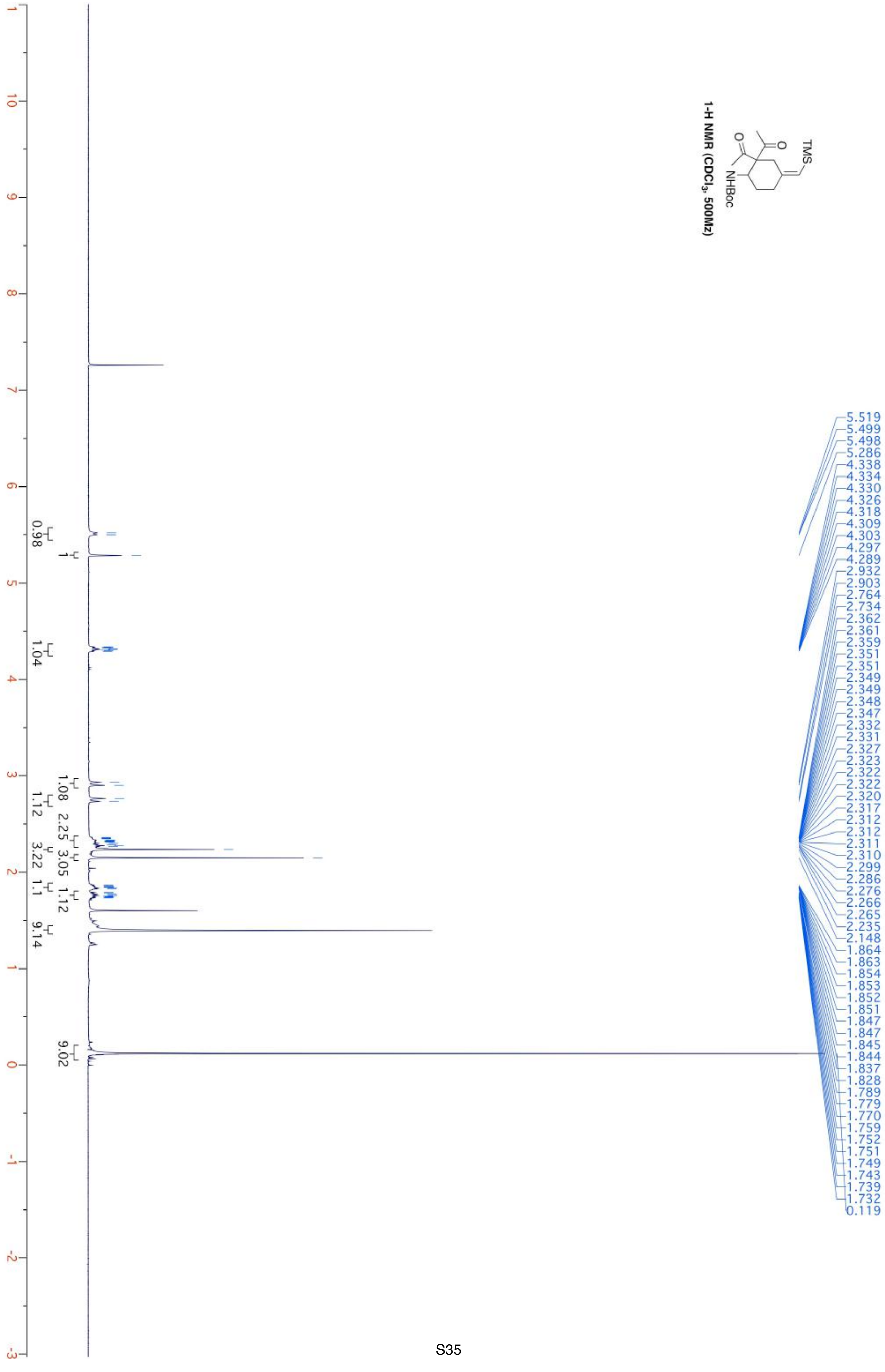

TMS
O
O
NHBoc
1-H NMR (CDCl3, 500Mz)
5.519
5.499
5.498
5.286
4.338
4.334
4.330
4.326
4.318
4.309
4.303
4.297
4.289
2.932
2.903
2.764
2.734
2.362
2.361
2.359
2.351
2.351
2.349
2.349
2.348
2.347
2.332
2.331
2.327
2.323
2.322
2.322
2.320
2.317
2.312
2.312
2.311
2.310
2.299
2.286
2.276
2.266
2.265
2.235
2.148
1.864
1.863
1.854
1.853
1.852
1.851
1.847
1.847
1.845
1.844
1.837
1.828
1.789
1.779
1.770
1.759
1.752
1.751
1.749
1.743
1.739
1.732
0.119
0.98
1
1.04
1.08
1.12
2.25
3.22
3.05
1.1
1.12
9.14
9.02
11
10
9
8
7
6
5
4
3
2
1
0
-1
-2
-3

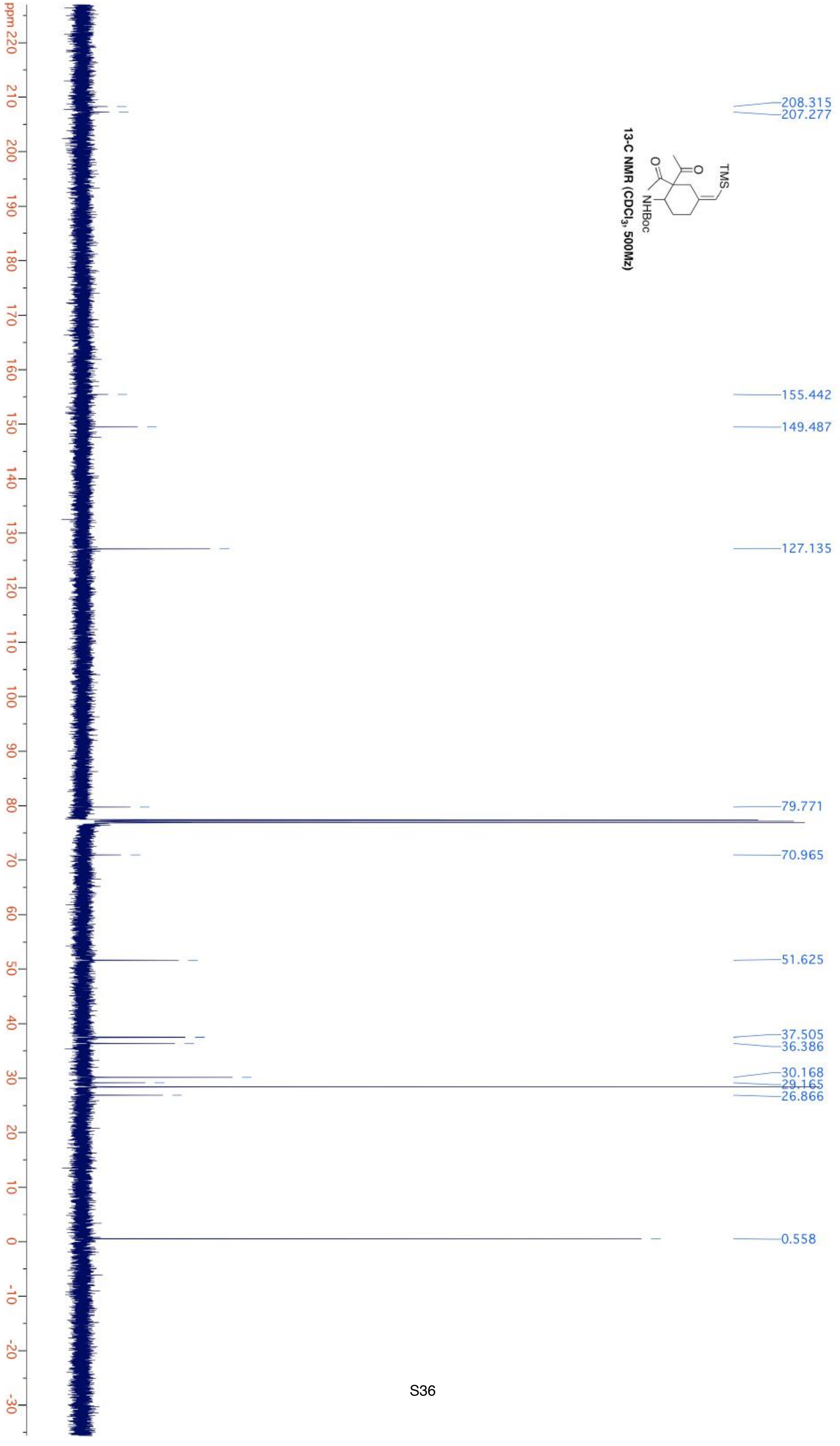
TMS
O
NHBoc
13-C NMR (CDCl3, 500Mz)
208.315
207.277
155.442
149.487
127.135
79.771
70.965
51.625
37.505
36.386
30.168
29.165
26.866
0.558
ppm 220
210
200
190
180
170
160
150
140
130
120
110
100
90
80
70
60
50
40
30
20
10
0
-10
-20
-30

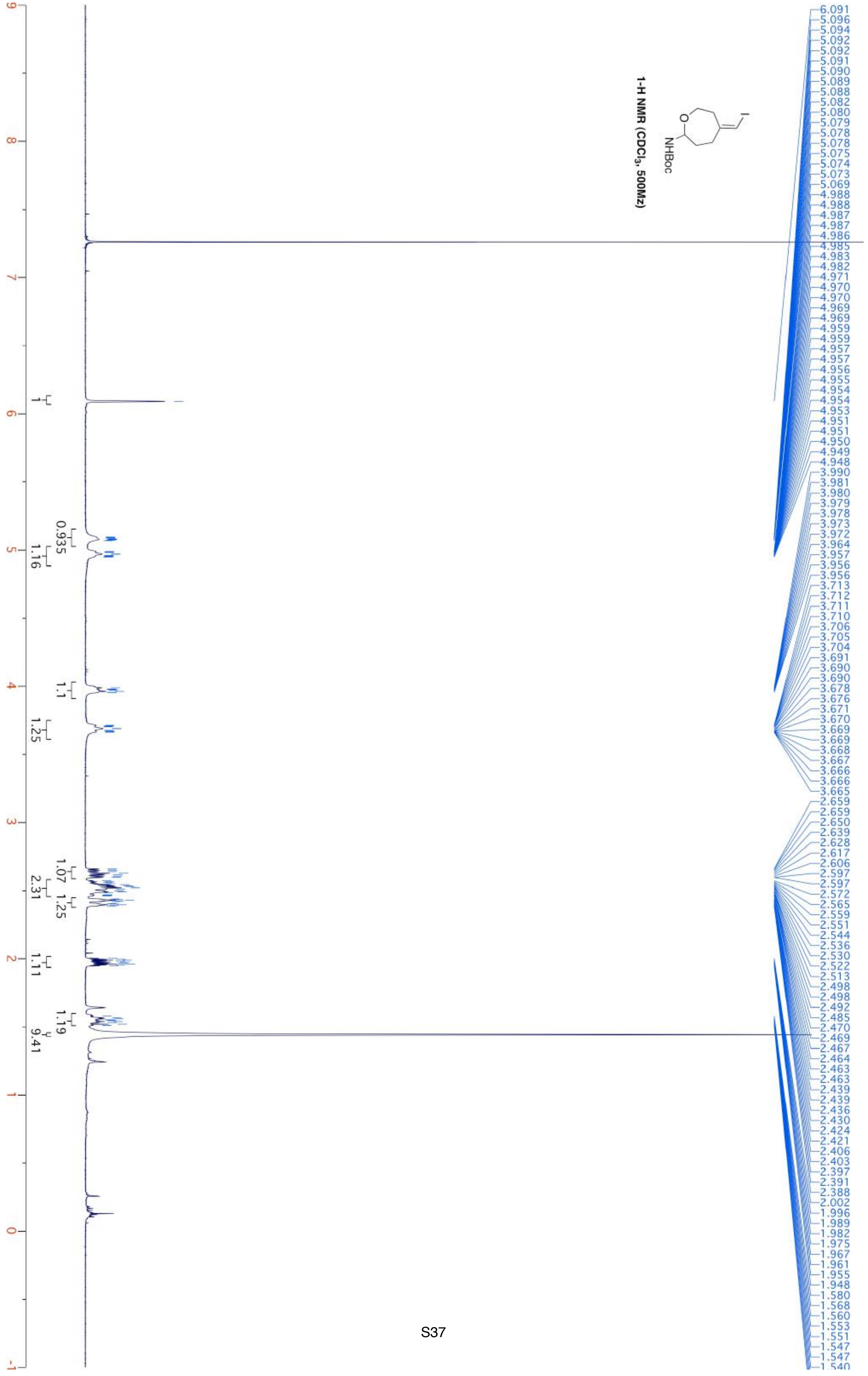
1-H NMR (CDCl$_3$, 500Mz)
I
O
NHBoc
6.091
5.096
5.094
5.092
5.092
5.091
5.090
5.089
5.088
5.082
5.080
5.079
5.078
5.078
5.075
5.074
5.073
5.069
4.988
4.988
4.987
4.987
4.986
4.985
4.983
4.982
4.971
4.970
4.970
4.969
4.969
4.959
4.959
4.957
4.957
4.956
4.955
4.954
4.954
4.953
4.951
4.951
4.950
4.949
4.948
3.990
3.981
3.980
3.979
3.978
3.973
3.972
3.964
3.957
3.956
3.956
3.713
3.712
3.711
3.710
3.706
3.705
3.704
3.691
3.690
3.690
3.678
3.676
3.671
3.670
3.669
3.669
3.668
3.667
3.666
3.666
3.665
2.659
2.659
2.650
2.639
2.628
2.617
2.606
2.597
2.597
2.572
2.565
2.559
2.551
2.544
2.536
2.530
2.522
2.513
2.498
2.498
2.492
2.485
2.470
2.469
2.467
2.464
2.463
2.463
2.439
2.439
2.436
2.430
2.424
2.421
2.406
2.403
2.397
2.391
2.388
2.002
1.996
1.989
1.982
1.975
1.967
1.961
1.955
1.948
1.580
1.568
1.560
1.553
1.551
1.547
1.547
1.540
1
0.935
1.16
1.1
1.25
1.07
2.31
1.25
1.11
1.19
9.41
9
8
7
6
5
4
3
2
1
0
-1

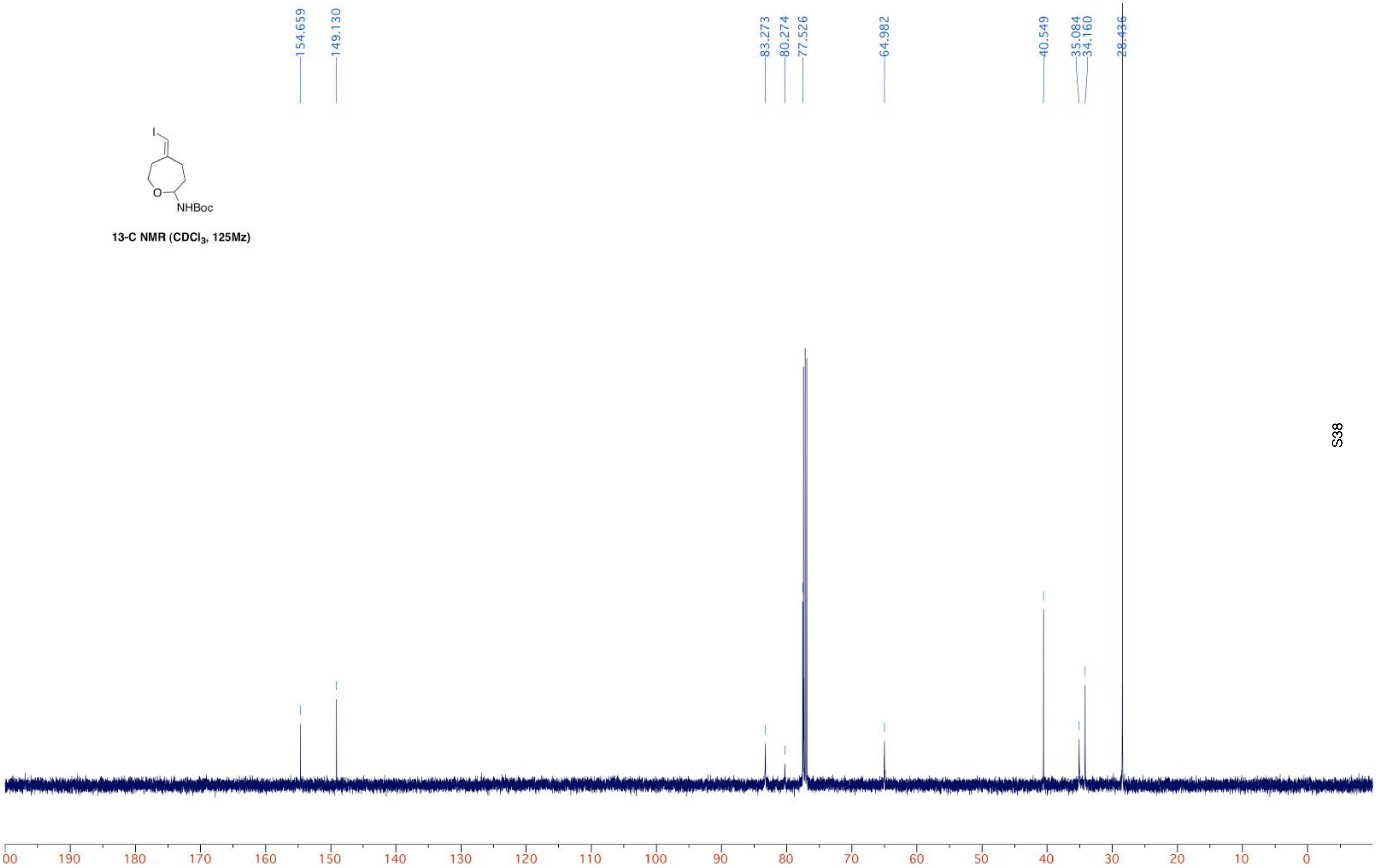

13-C NMR ($CDCl_3$, 125Mz)

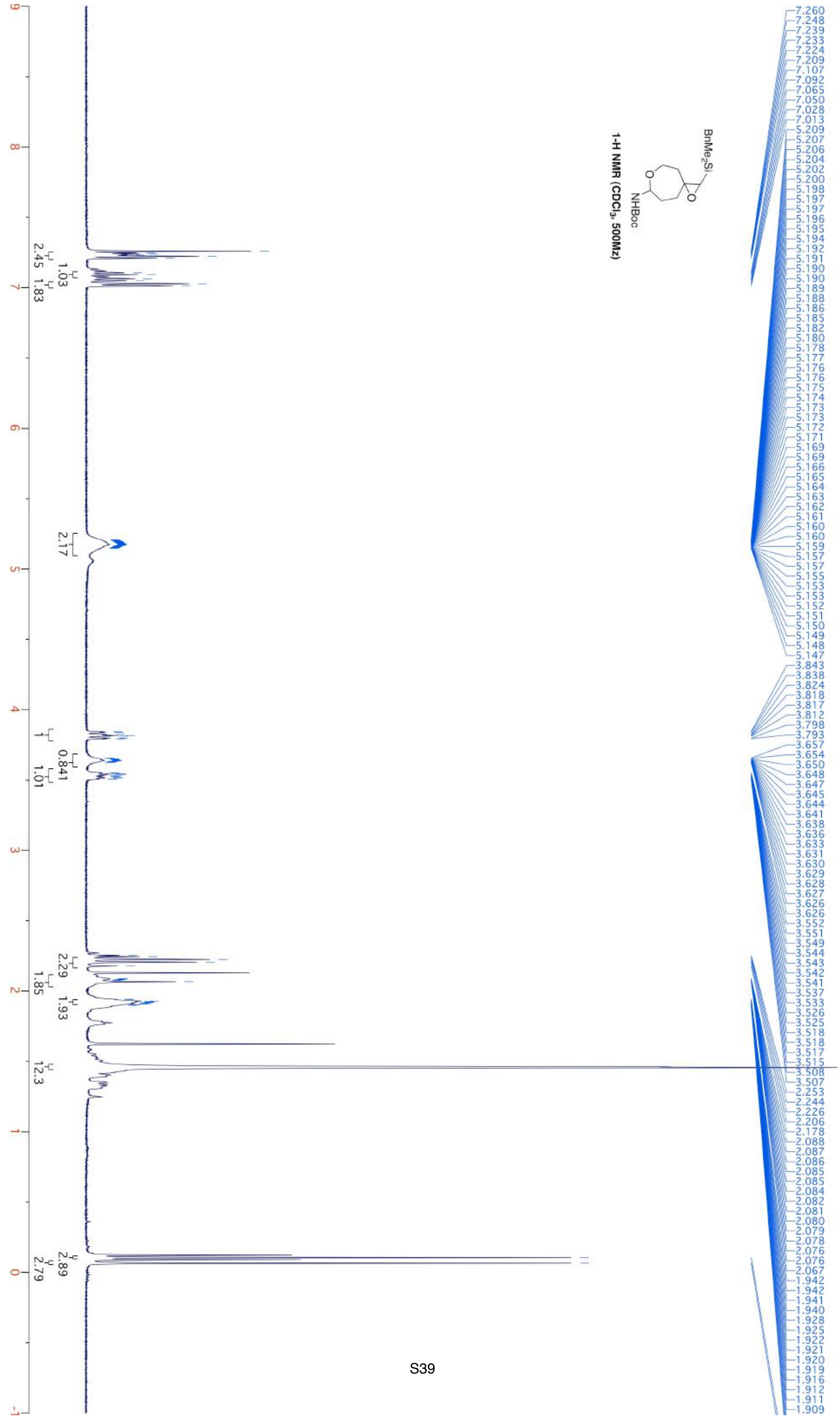
BnMe2Si
O
NHBoc
1-H NMR (CDCl3, 500Mz)

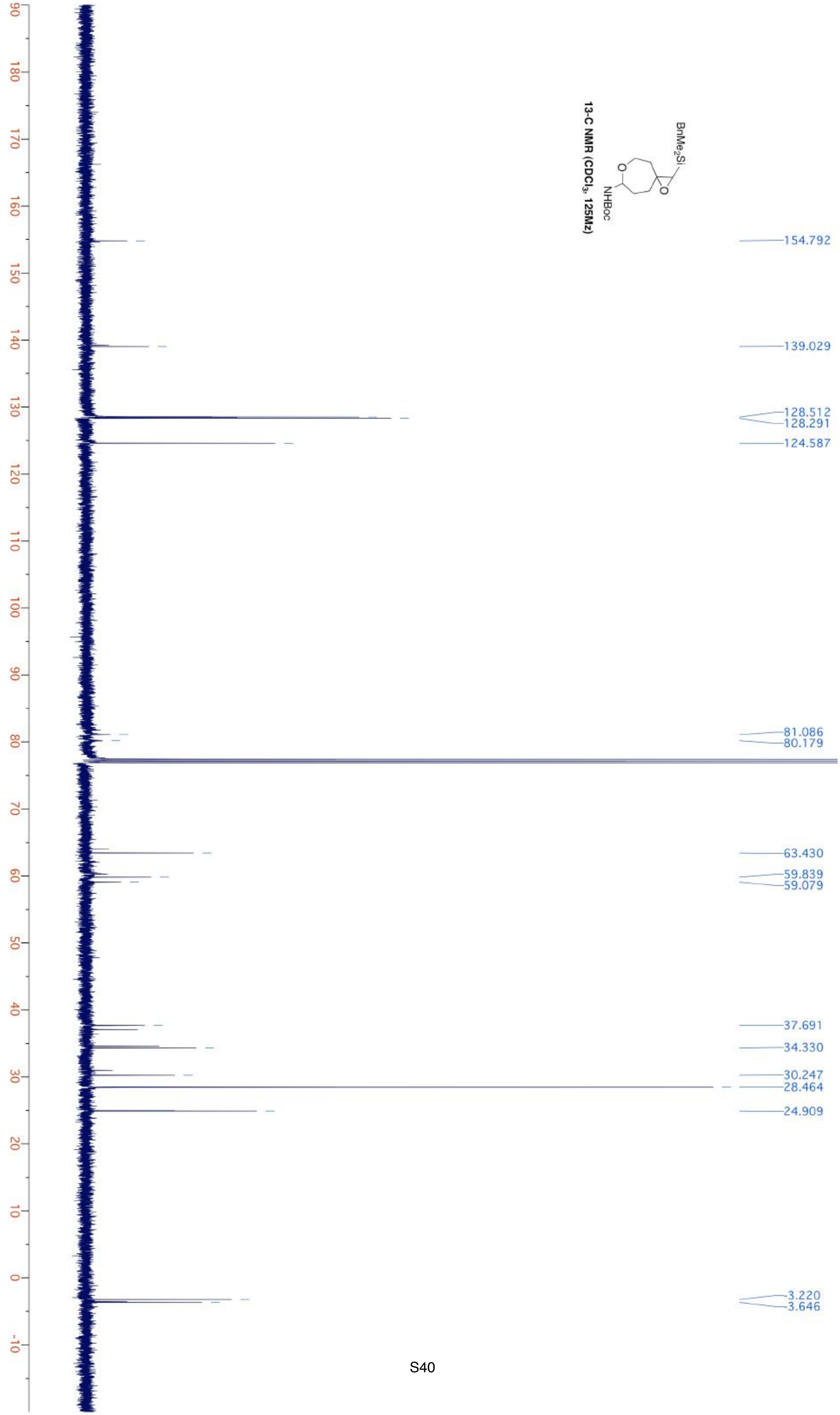
BnMe2Si
O
O
NHBoc
13-C NMR (CDCl3, 125Mz)
154.792
139.029
128.512
128.291
124.587
81.086
80.179
63.430
59.839
59.079
37.691
34.330
30.247
28.464
24.909
-3.220
-3.646

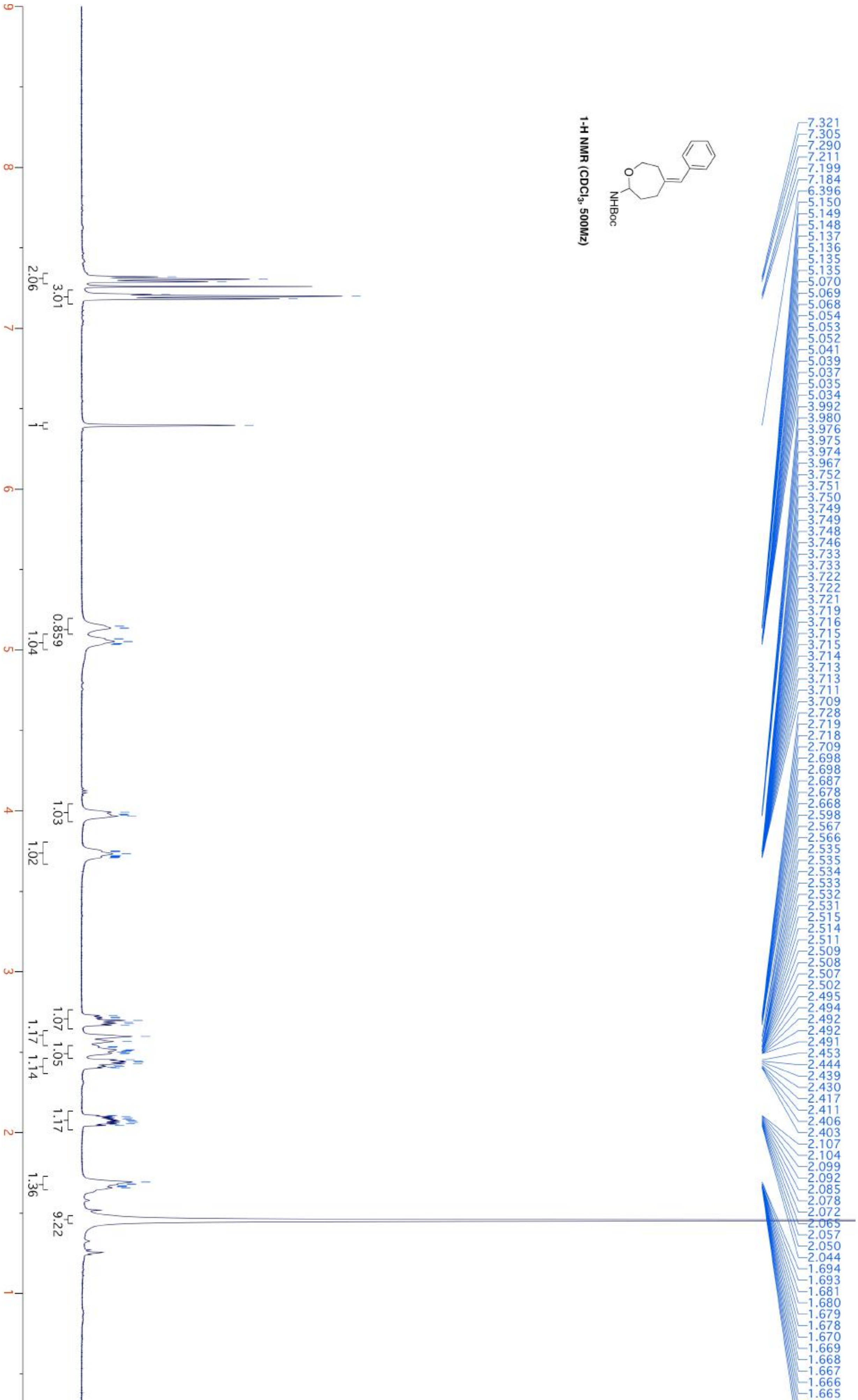
1-H NMR (CDCl3, 500Mz)
O
NHBoc

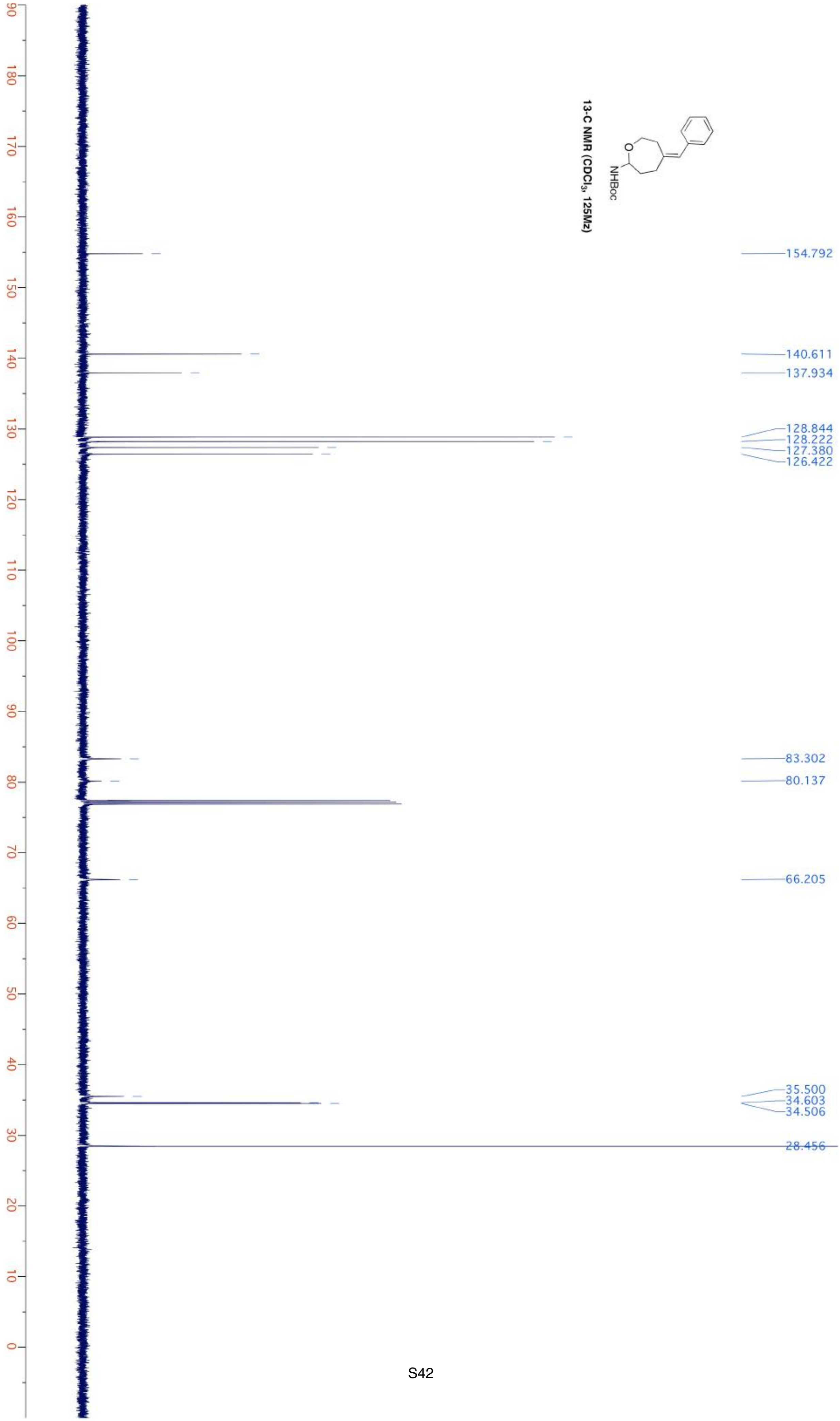

13-C NMR (CDCl3, 125Mz)
O
NHBoc
154.792
140.611
137.934
128.844
128.222
127.380
126.422
83.302
80.137
66.205
35.500
34.603
34.506
28.456
190
180
170
160
150
140
130
120
110
100
90
80
70
60
50
40
30
20
10
0

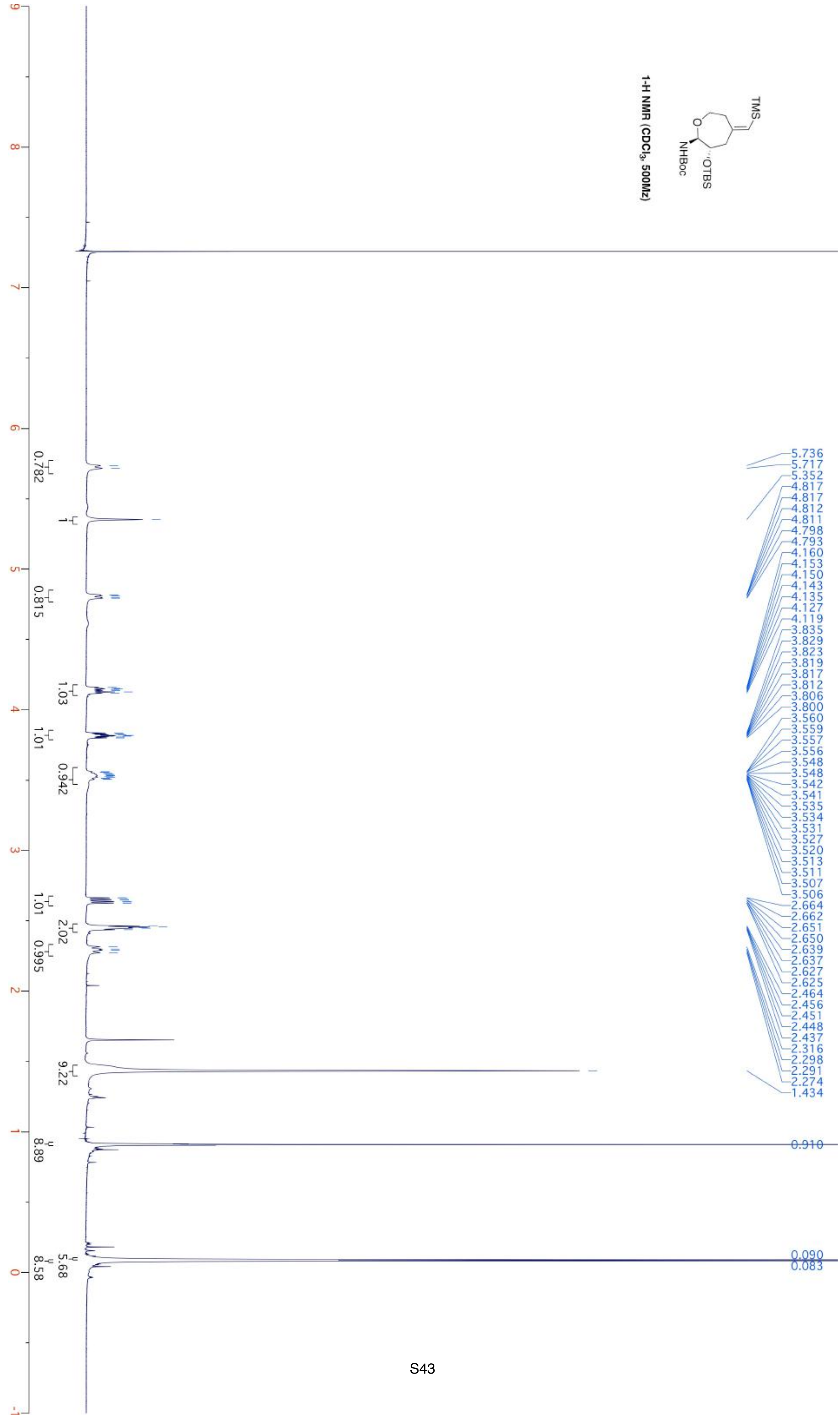
1-H NMR (CDCl$_3$, 500Mz)
TMS
O
OTBS
NHBoc
5.736
5.717
5.352
4.817
4.817
4.812
4.811
4.798
4.793
4.160
4.153
4.150
4.143
4.135
4.127
4.119
3.835
3.829
3.823
3.819
3.817
3.812
3.806
3.800
3.560
3.559
3.557
3.556
3.548
3.548
3.542
3.541
3.535
3.534
3.531
3.527
3.520
3.513
3.511
3.507
3.506
2.664
2.662
2.651
2.650
2.639
2.637
2.627
2.625
2.464
2.456
2.451
2.448
2.437
2.316
2.298
2.291
2.274
1.434
0.910
0.090
0.083
0.782
1
0.815
1.03
1.01
0.942
1.01
2.02
0.995
9.22
8.89
5.68
8.58
9
8
7
6
5
4
3
2
1
0
-1

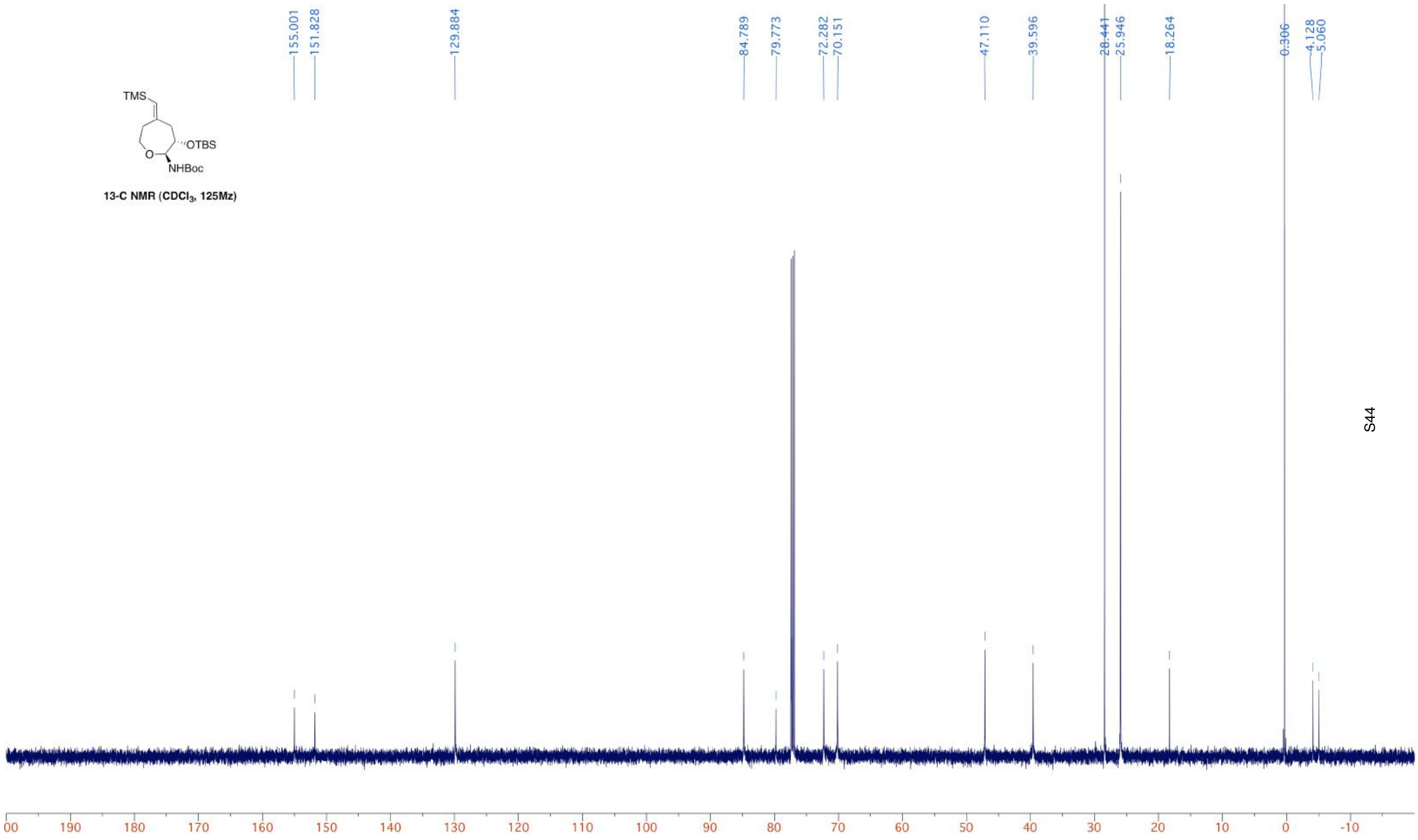

13-C NMR ($CDCl_3$, 125Mz)

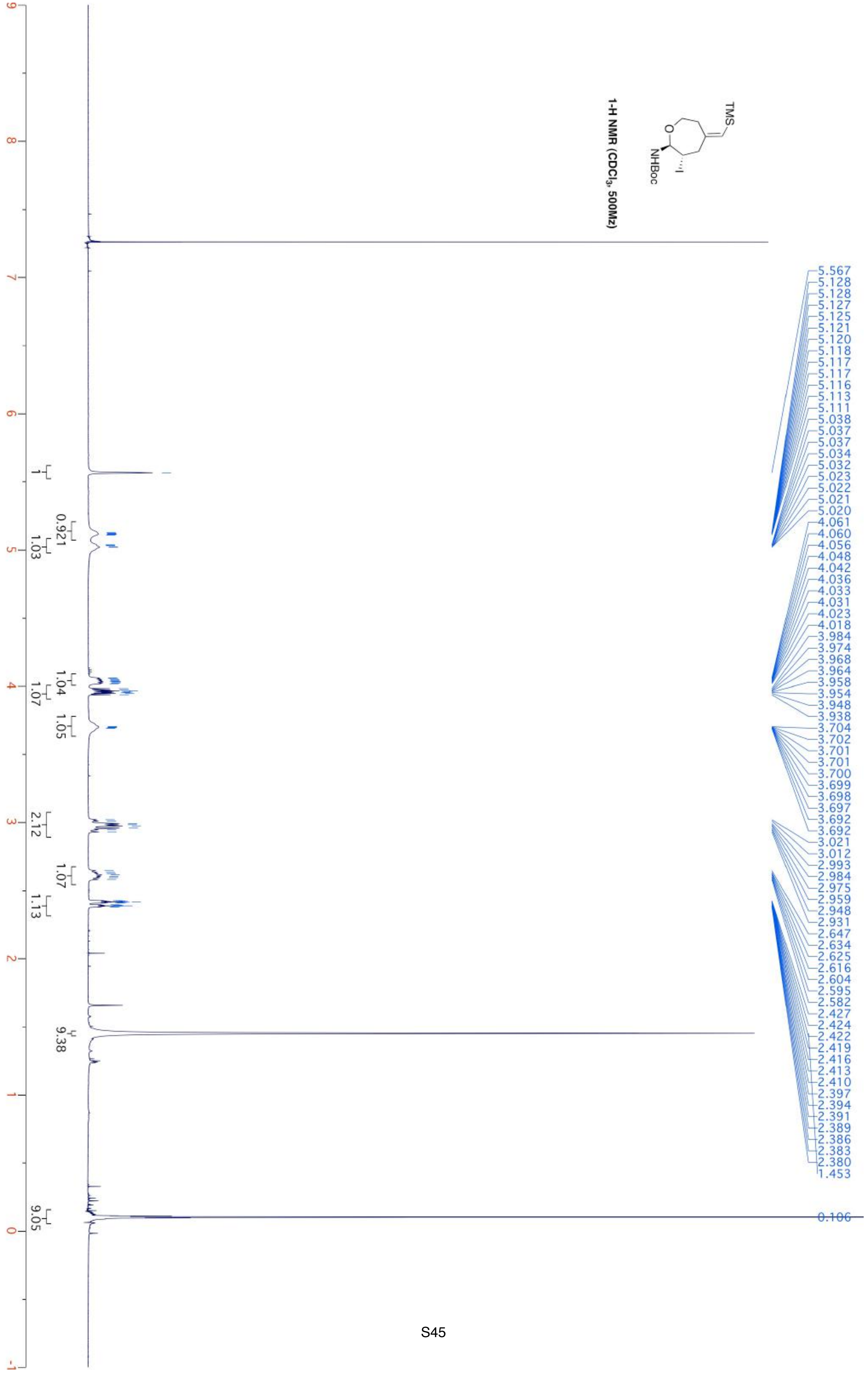

TMS
O
NHBoc
I
1-H NMR (CDCl3, 500Mz)
5.567
5.128
5.128
5.127
5.125
5.121
5.120
5.118
5.117
5.117
5.116
5.113
5.111
5.038
5.037
5.037
5.034
5.032
5.023
5.022
5.021
5.020
4.061
4.060
4.056
4.048
4.042
4.036
4.033
4.031
4.023
4.018
3.984
3.974
3.968
3.964
3.958
3.954
3.948
3.938
3.704
3.702
3.701
3.701
3.700
3.699
3.698
3.697
3.692
3.692
3.021
3.012
2.993
2.984
2.975
2.959
2.948
2.931
2.647
2.634
2.625
2.616
2.604
2.595
2.582
2.427
2.424
2.422
2.419
2.416
2.413
2.410
2.397
2.394
2.391
2.389
2.386
2.383
2.380
1.453
0.106
1
0.921
1.03
1.04
1.07
1.05
2.12
1.07
1.13
9.38
9.05
9
8
7
6
5
4
3
2
1
0
-1

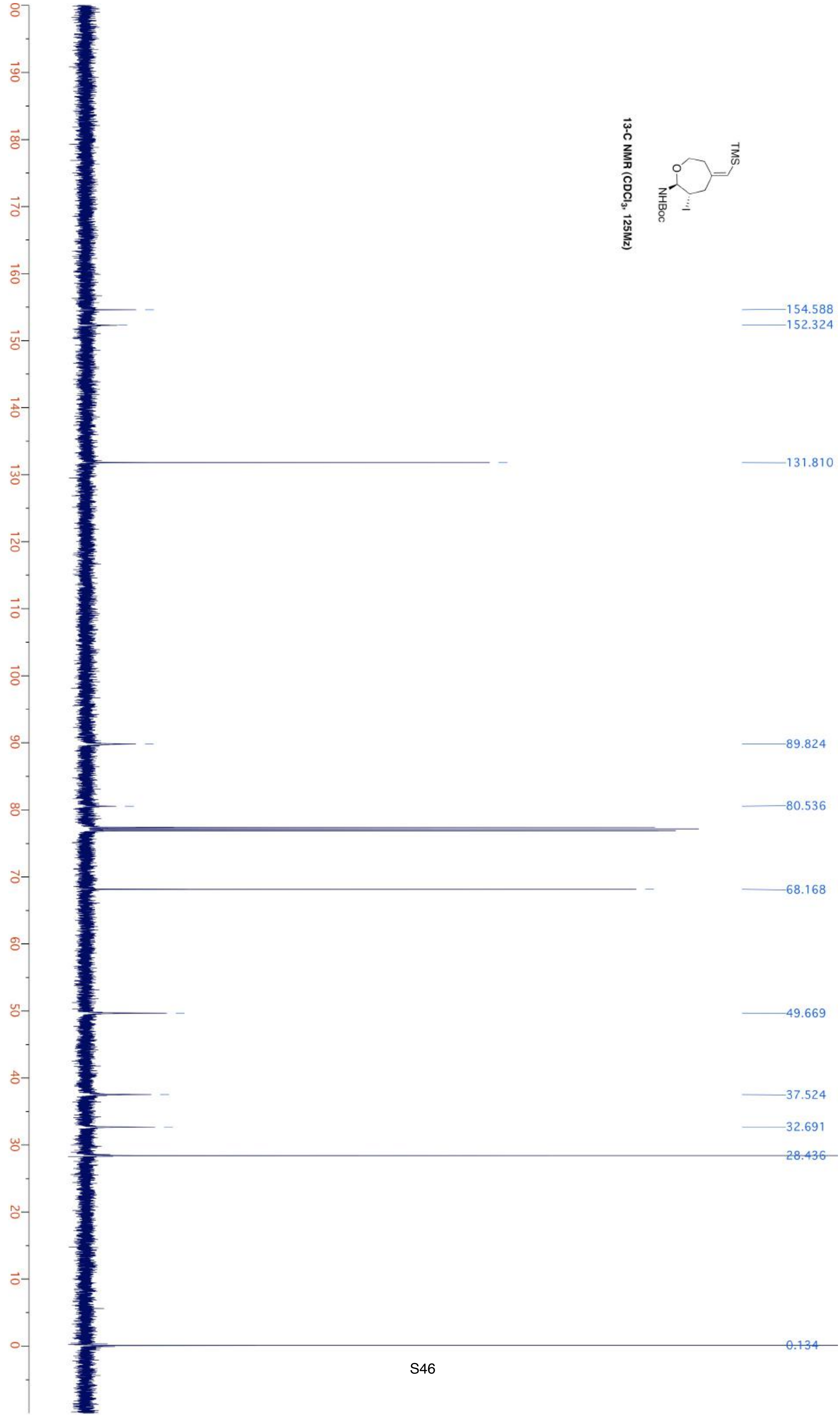

13-C NMR ($CDCl_3$, 125Mz)

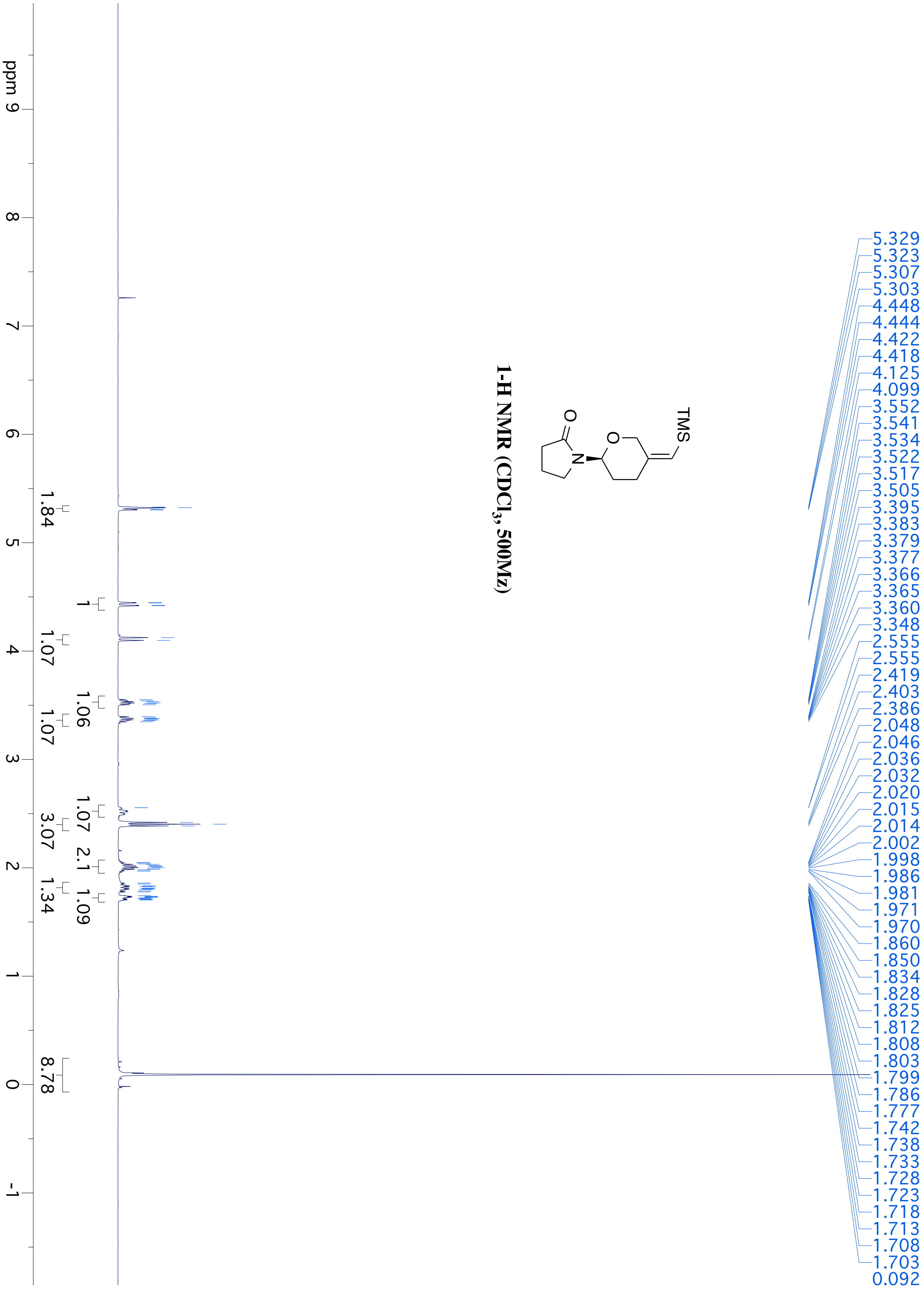
1-H NMR (CDCl3, 500Mz)
TMS
O
N
O
5.329
5.323
5.307
5.303
4.448
4.444
4.422
4.418
4.125
4.099
3.552
3.541
3.534
3.522
3.517
3.505
3.395
3.383
3.379
3.377
3.366
3.365
3.360
3.348
2.555
2.555
2.419
2.403
2.386
2.048
2.046
2.036
2.032
2.020
2.015
2.014
2.002
1.998
1.986
1.981
1.971
1.970
1.860
1.850
1.834
1.828
1.825
1.812
1.808
1.803
1.799
1.786
1.777
1.742
1.738
1.733
1.728
1.723
1.718
1.713
1.708
1.703
0.092
1.84
1
1.07
1.06
1.07
1.07
3.07
2.1
1.34
1.09
8.78
ppm 9
8
7
6
5
4
3
2
1
0
-1

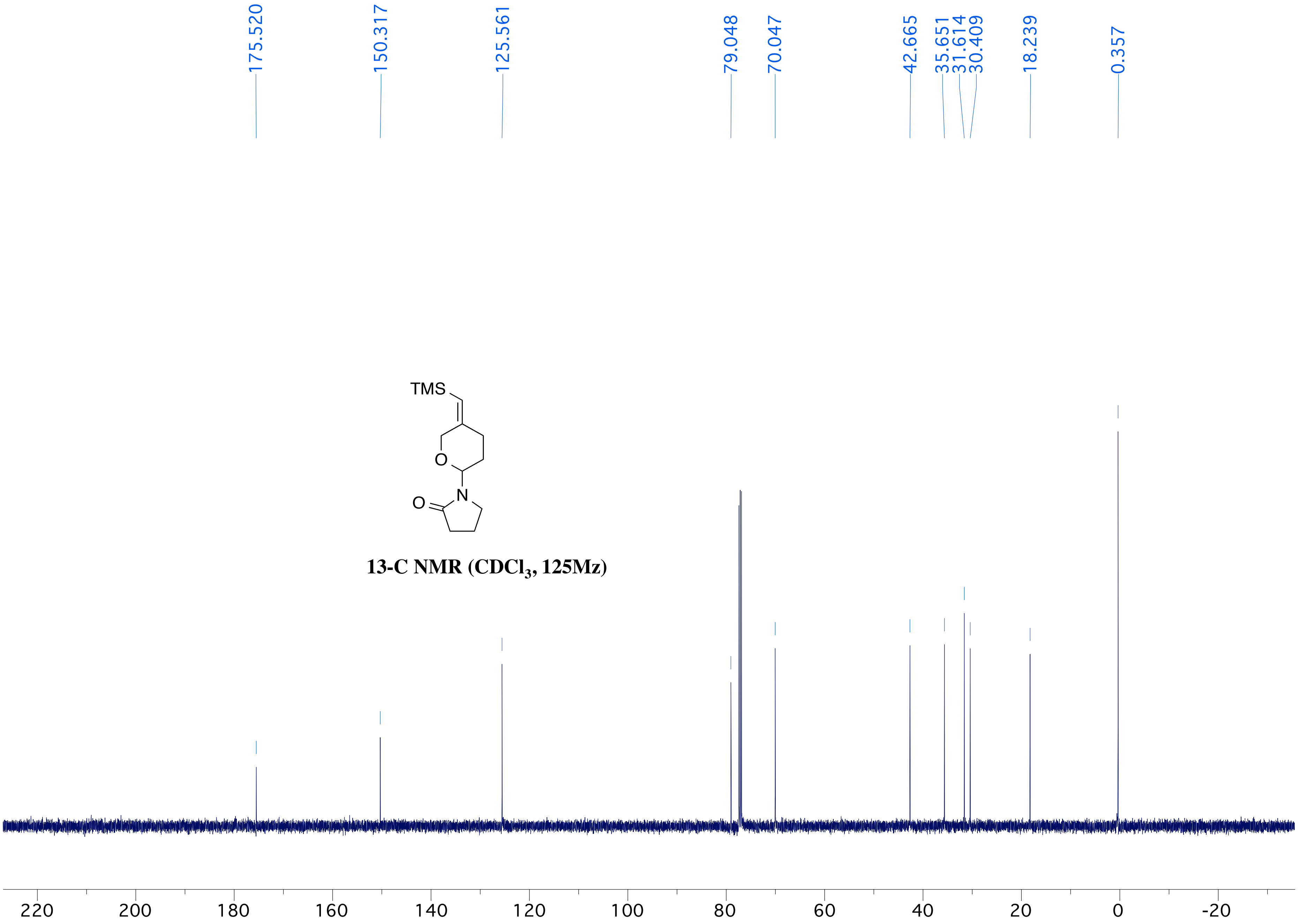
175.520
150.317
125.561
79.048
70.047
42.665
35.651
31.614
30.409
18.239
0.357
TMS
O
N
O
13-C NMR (CDCl$_3$, 125Mz)
220
200
180
160
140
120
100
80
60
40
20
0
-20

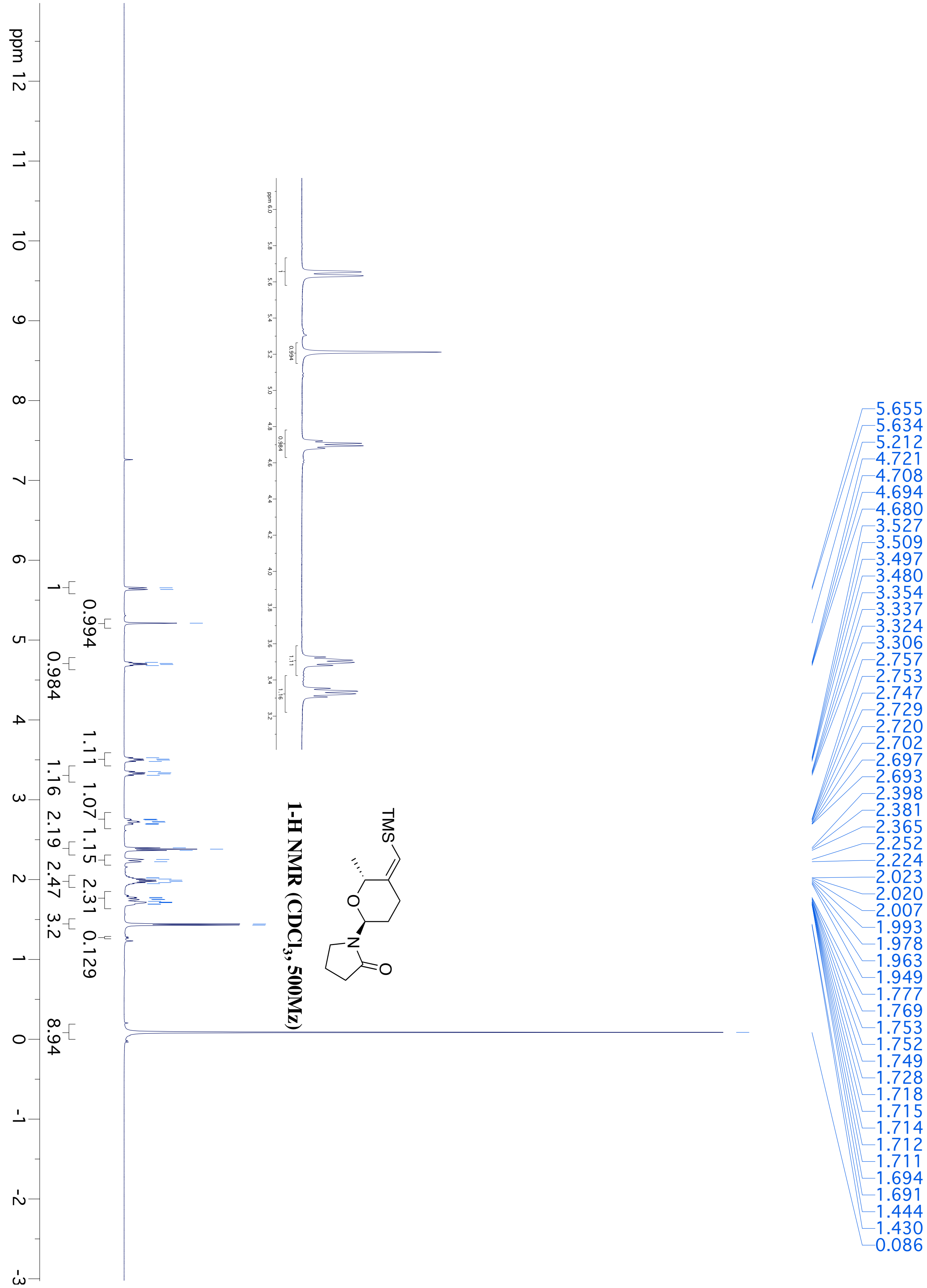
1-H NMR (CDCl3, 500Mz)
TMS
5.655
5.634
5.212
4.721
4.708
4.694
4.680
3.527
3.509
3.497
3.480
3.354
3.337
3.324
3.306
2.757
2.753
2.747
2.729
2.720
2.702
2.697
2.693
2.398
2.381
2.365
2.252
2.224
2.023
2.020
2.007
1.993
1.978
1.963
1.949
1.777
1.769
1.753
1.752
1.749
1.728
1.718
1.715
1.714
1.712
1.711
1.694
1.691
1.444
1.430
0.086
ppm

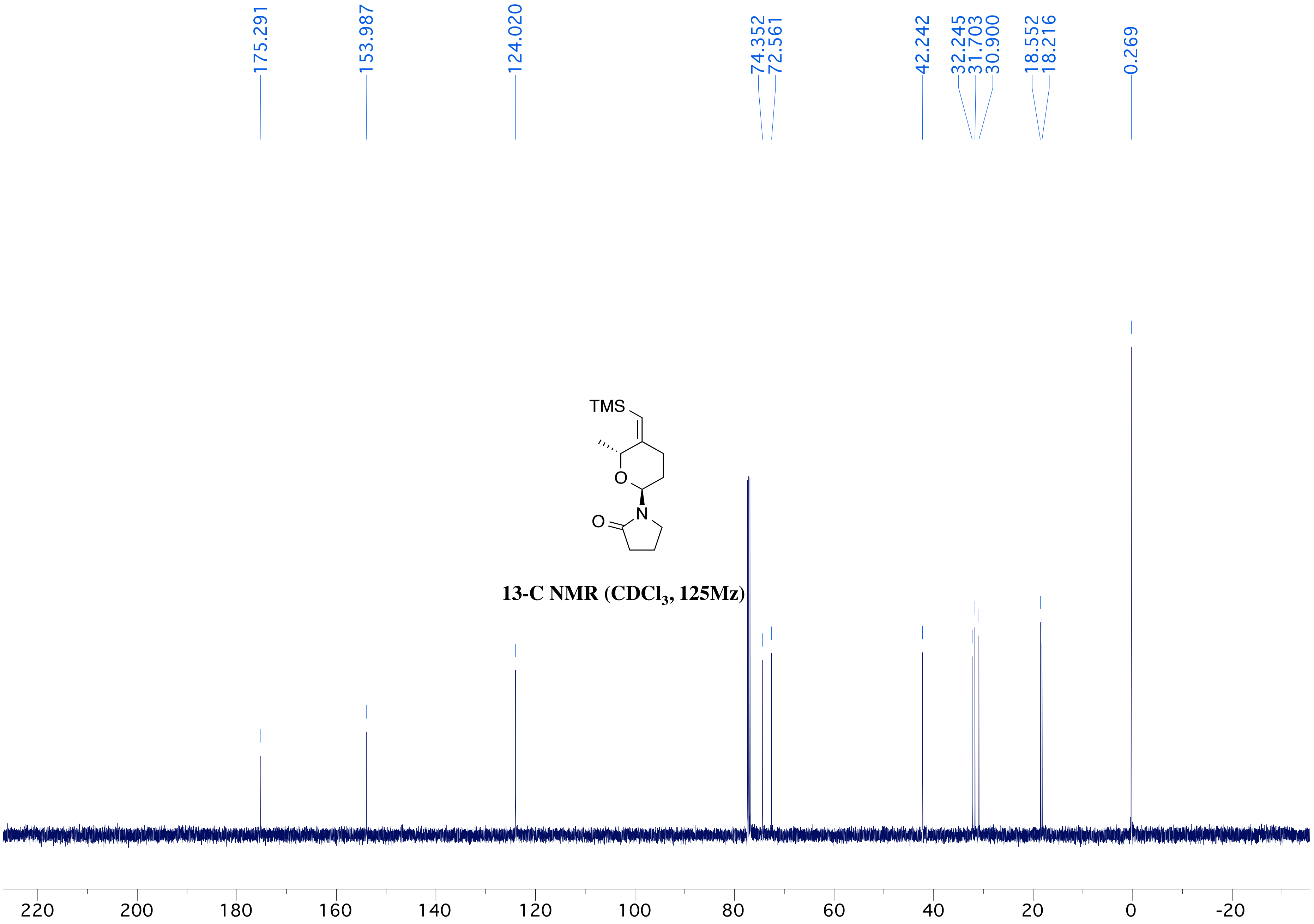

13-C NMR ($CDCl_3$, 125Mz)

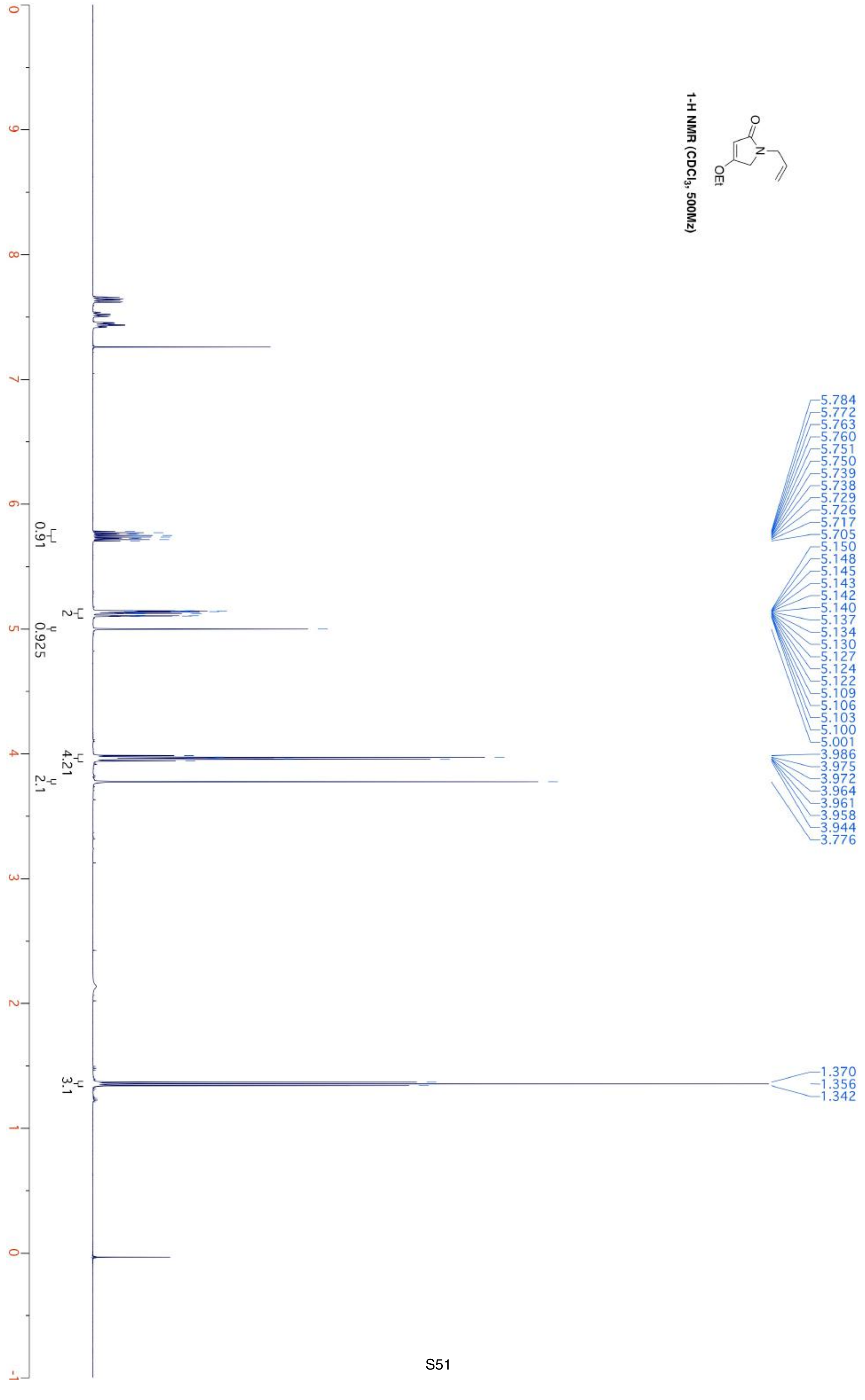
OEt
1-H NMR (CDCl$_3$, 500Mz)
5.784
5.772
5.763
5.760
5.751
5.750
5.739
5.738
5.729
5.726
5.717
5.705
5.150
5.148
5.145
5.143
5.142
5.140
5.137
5.134
5.130
5.127
5.124
5.122
5.109
5.106
5.103
5.100
5.001
3.986
3.975
3.972
3.964
3.961
3.958
3.944
3.776
1.370
1.356
1.342
0.91
2
0.925
4.21
2.1
3.1

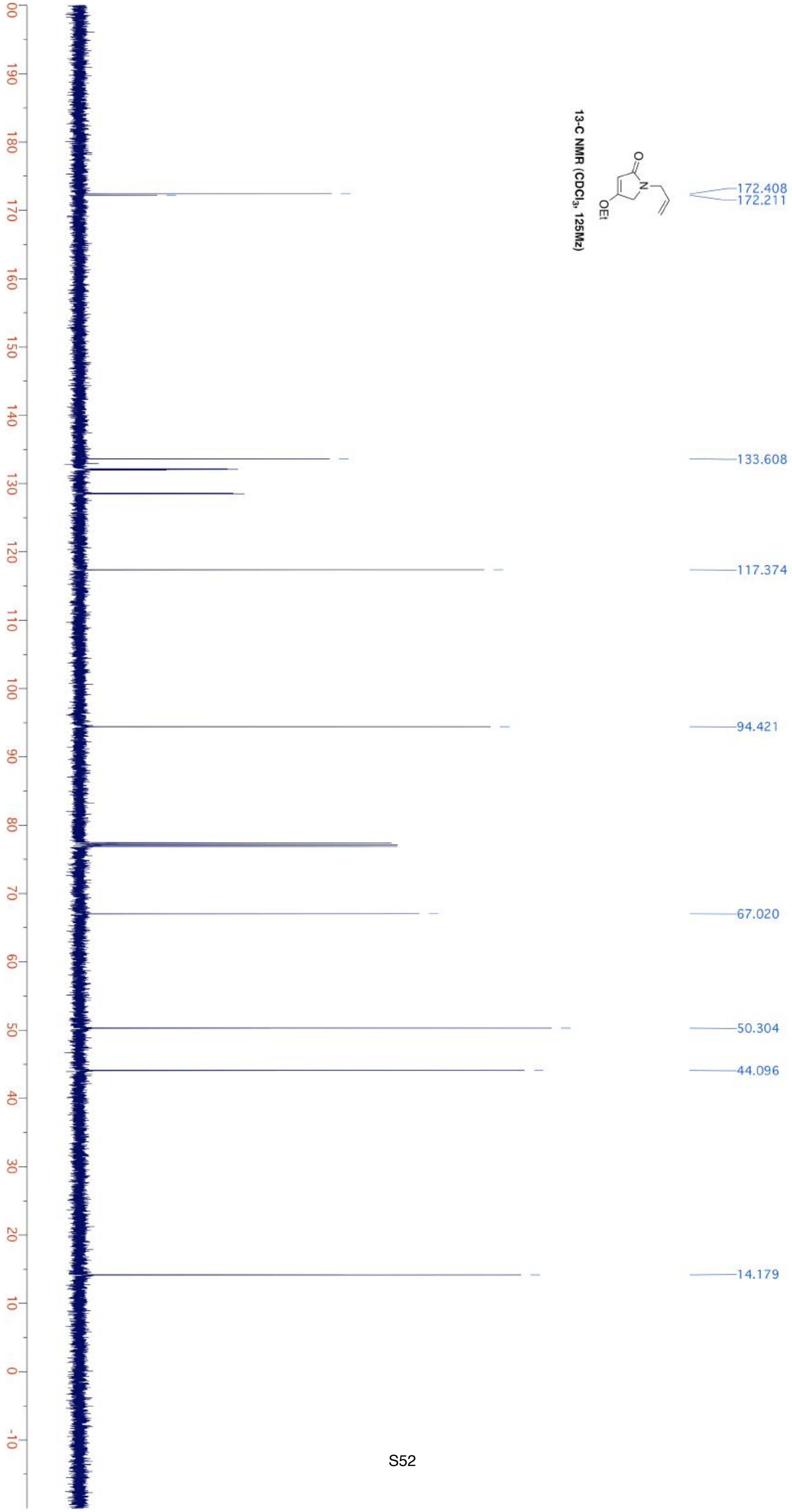
13-C NMR (CDCl3, 125Mz)
OEt
O
N
172.408
172.211
133.608
117.374
94.421
67.020
50.304
44.096
14.179
190
180
170
160
150
140
130
120
110
100
90
80
70
60
50
40
30
20
10
0
-10

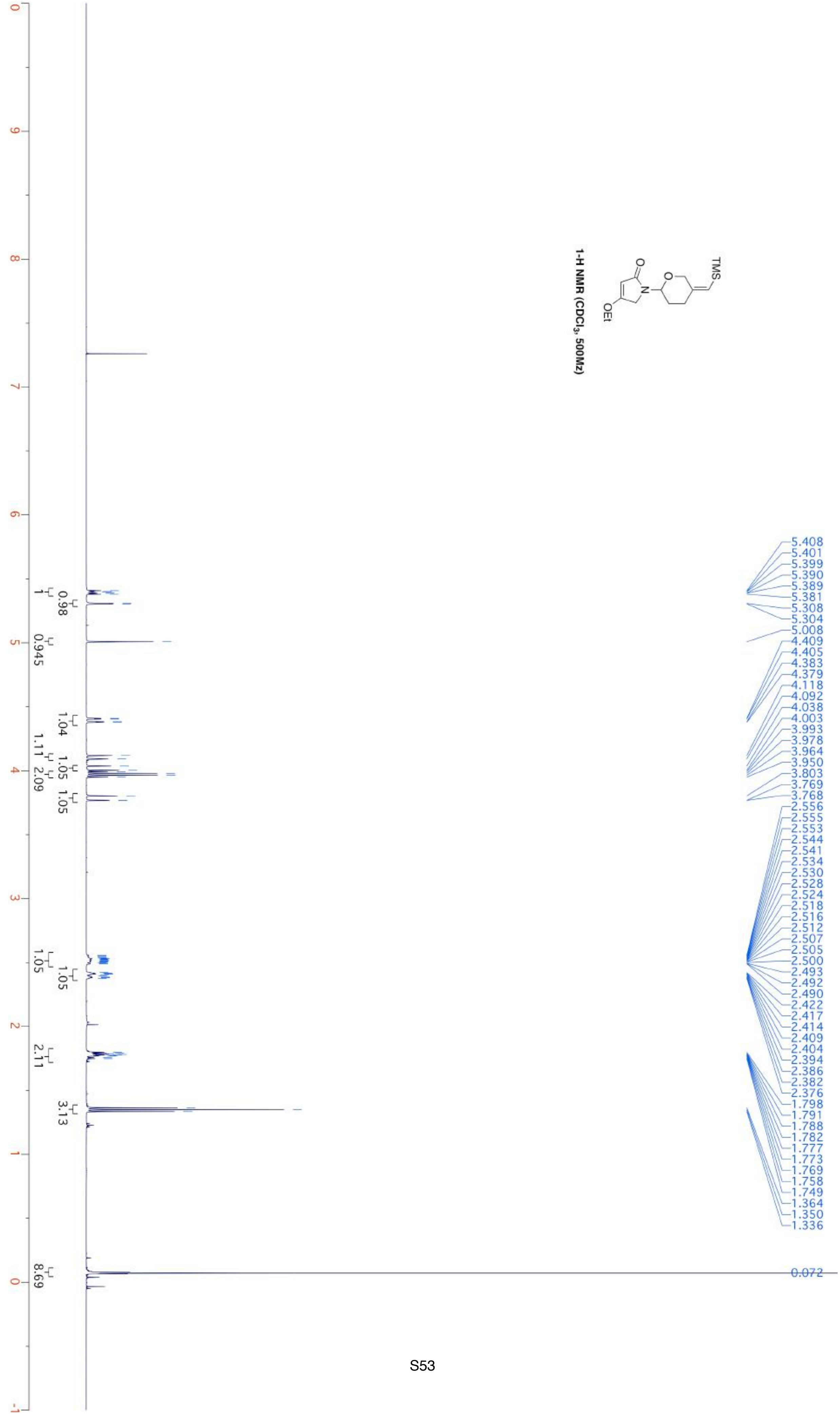

1-H NMR (CDCl$_3$, 500Mz)
TMS
O
N
O
OEt
5.408
5.401
5.399
5.390
5.389
5.381
5.308
5.304
5.008
4.409
4.405
4.383
4.379
4.118
4.092
4.038
4.003
3.993
3.978
3.964
3.950
3.803
3.769
3.768
2.556
2.555
2.553
2.544
2.541
2.534
2.530
2.528
2.524
2.518
2.516
2.512
2.507
2.505
2.500
2.493
2.492
2.490
2.422
2.417
2.414
2.409
2.404
2.394
2.386
2.382
2.376
1.798
1.791
1.788
1.782
1.777
1.773
1.769
1.758
1.749
1.364
1.350
1.336
0.072

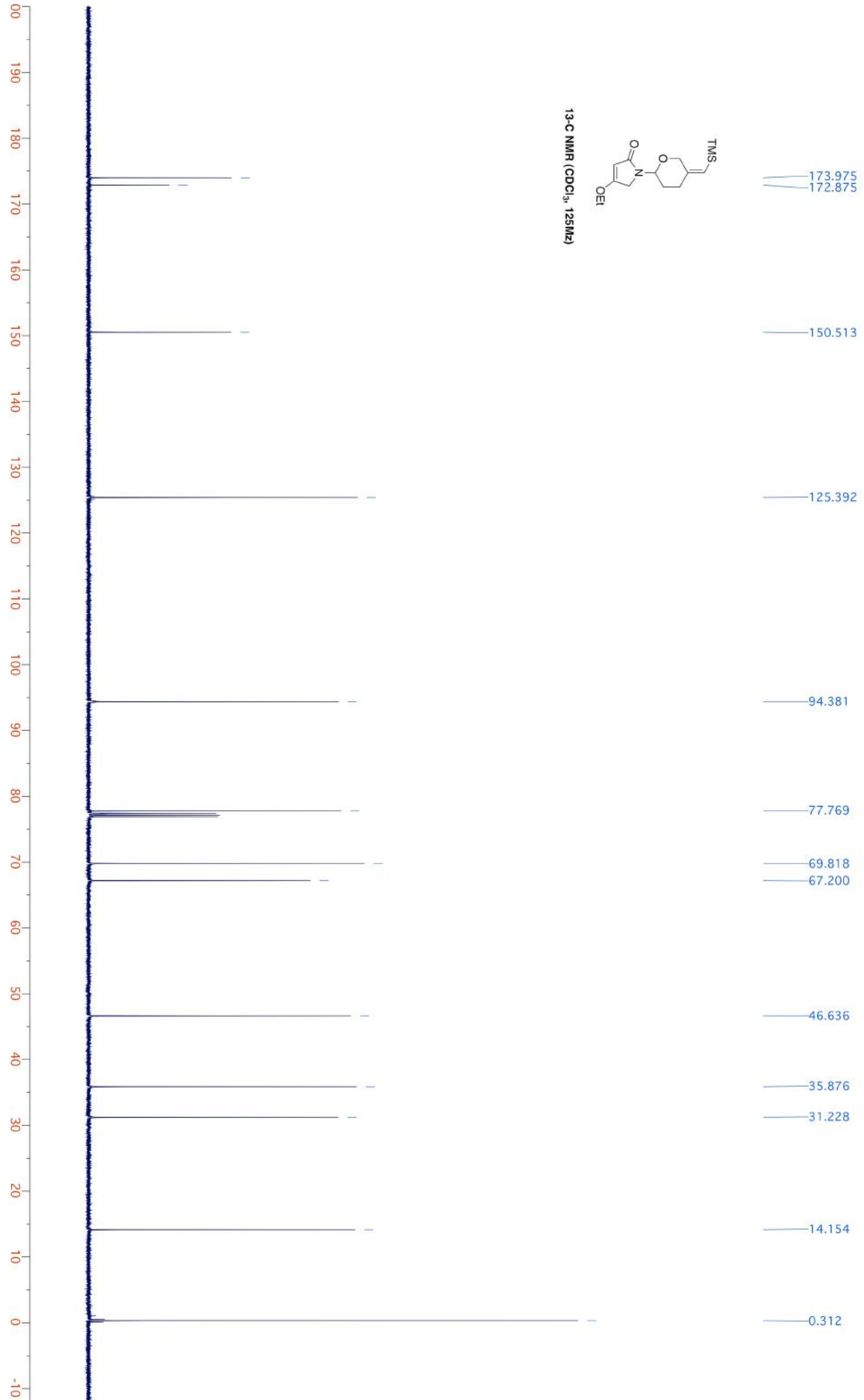
13-C NMR (CDCl$_3$, 125Mz)
TMS
O
N
OEt
173.975
172.875
150.513
125.392
94.381
77.769
69.818
67.200
46.636
35.876
31.228
14.154
0.312
200
190
180
170
160
150
140
130
120
110
100
90
80
70
60
50
40
30
20
10
0
-10

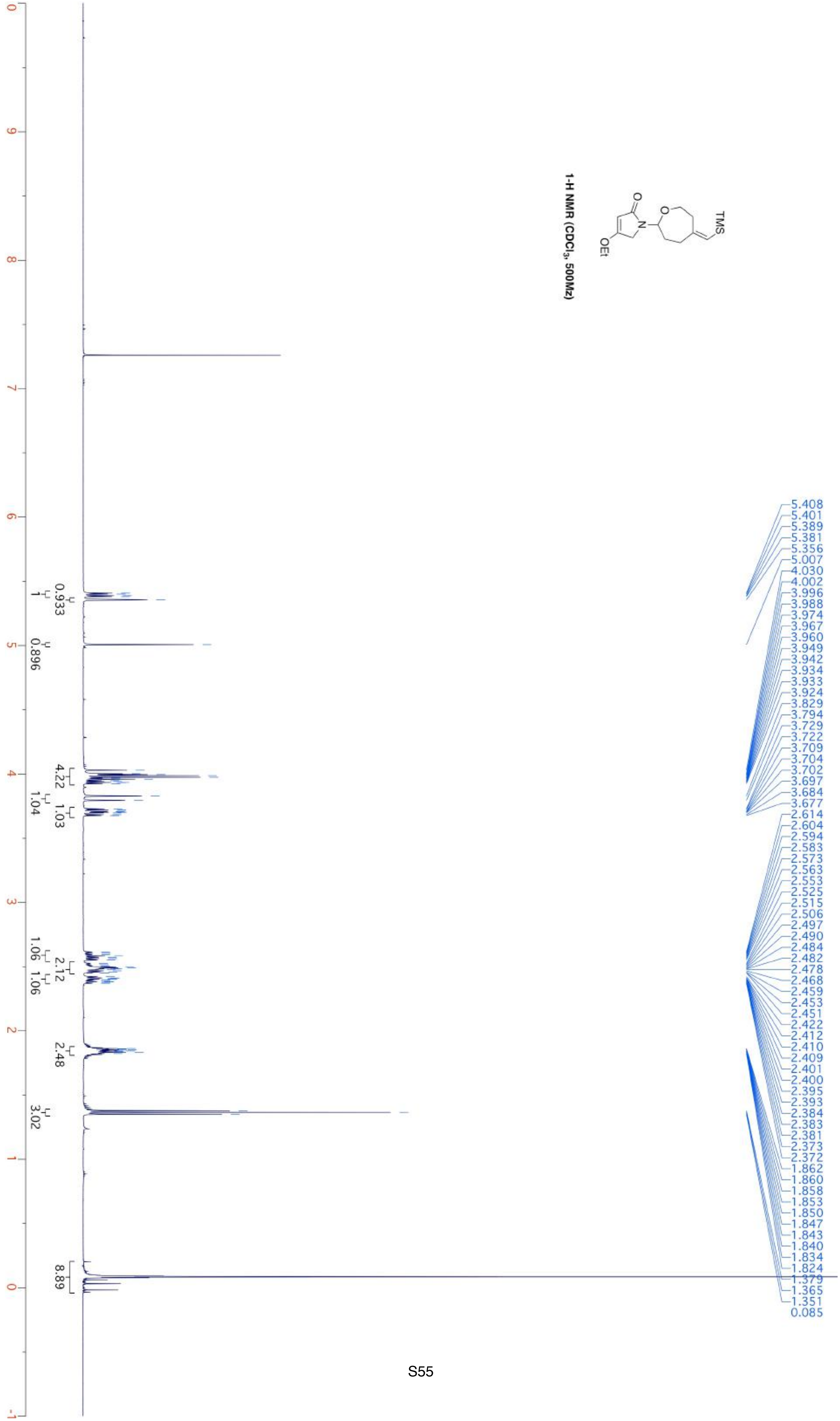

1-H NMR ($CDCl_3$, 500Mz)

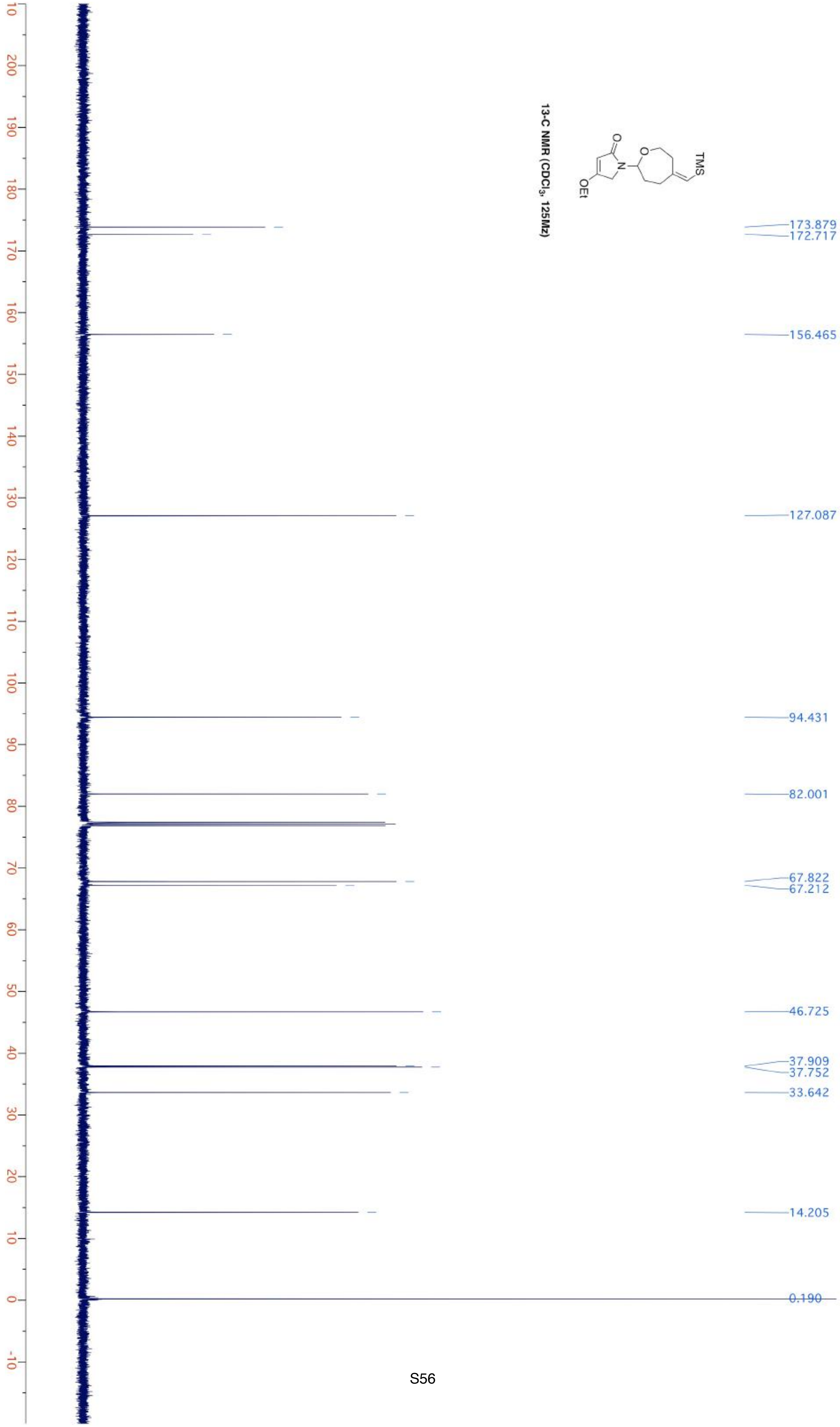
13-C NMR (CDCl$_3$, 125Mz)
TMS
O
N
OEt
173.879
172.717
156.465
127.087
94.431
82.001
67.822
67.212
46.725
37.909
37.752
33.642
14.205
0.190

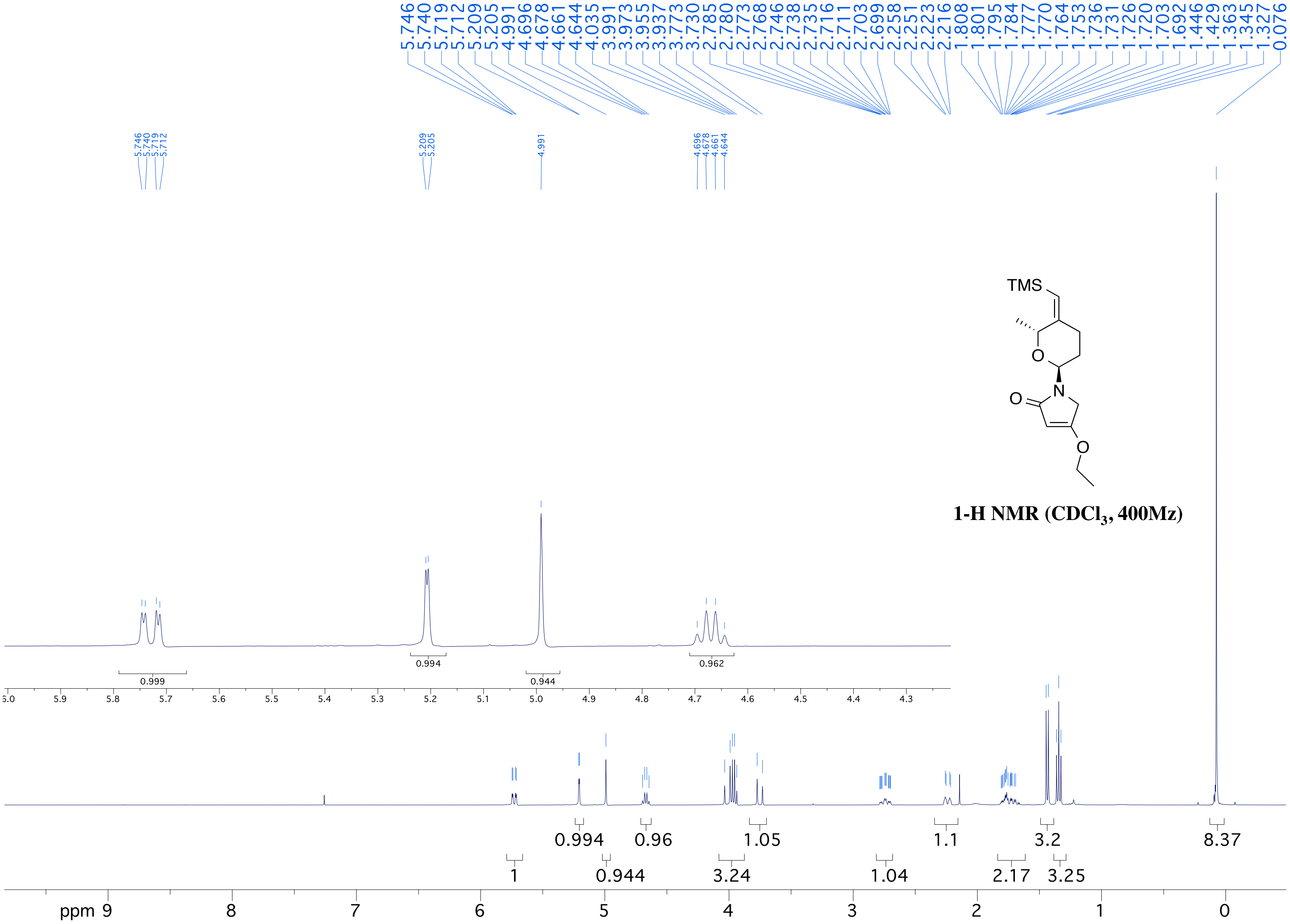

1-H NMR ($CDCl_3$, 400Mz)

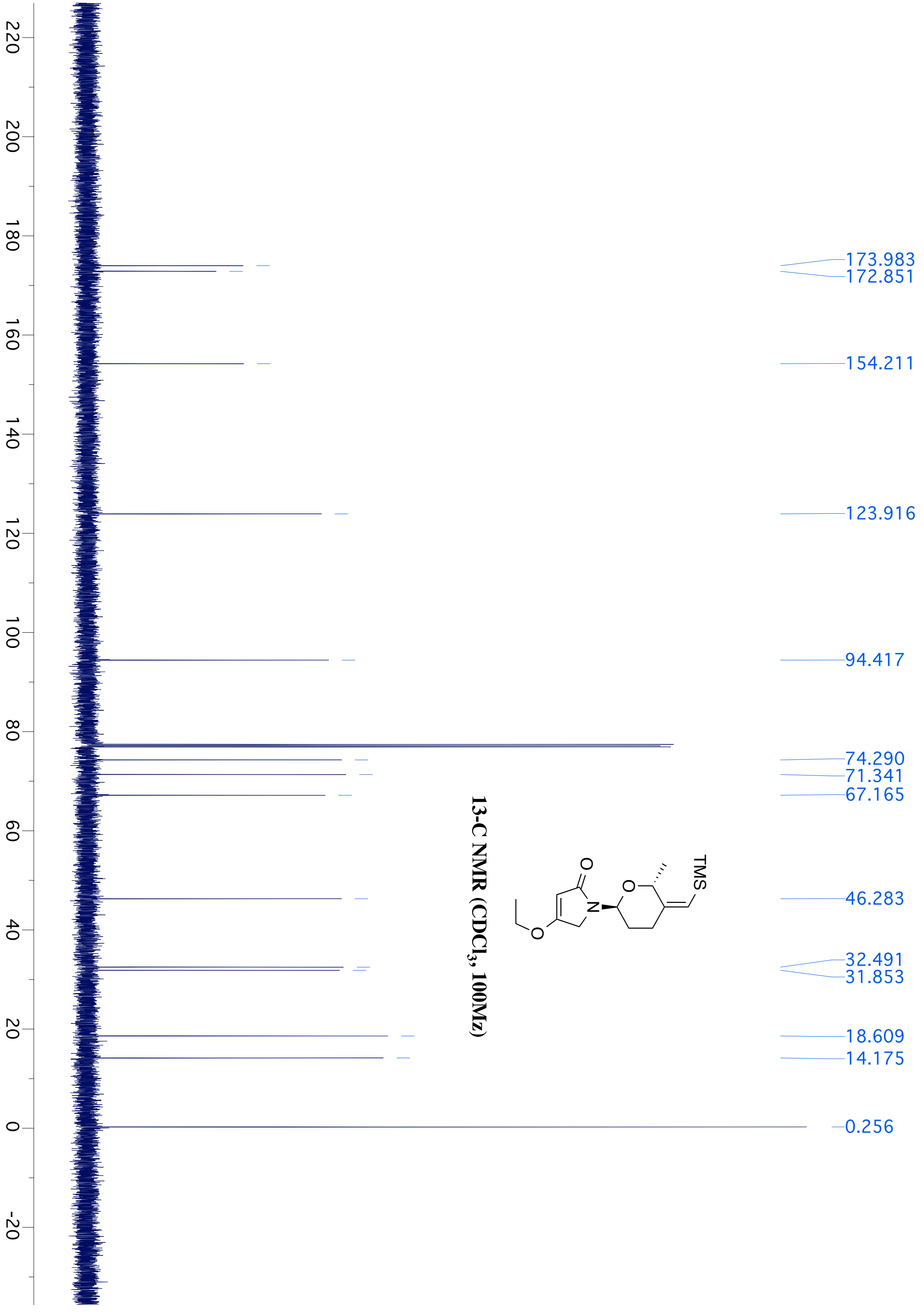
13-C NMR (CDCl$_3$, 100Mz)
173.983
172.851
154.211
123.916
94.417
74.290
71.341
67.165
46.283
32.491
31.853
18.609
14.175
0.256
TMS
O
N
O
O
220
200
180
160
140
120
100
80
60
40
20
0
-20

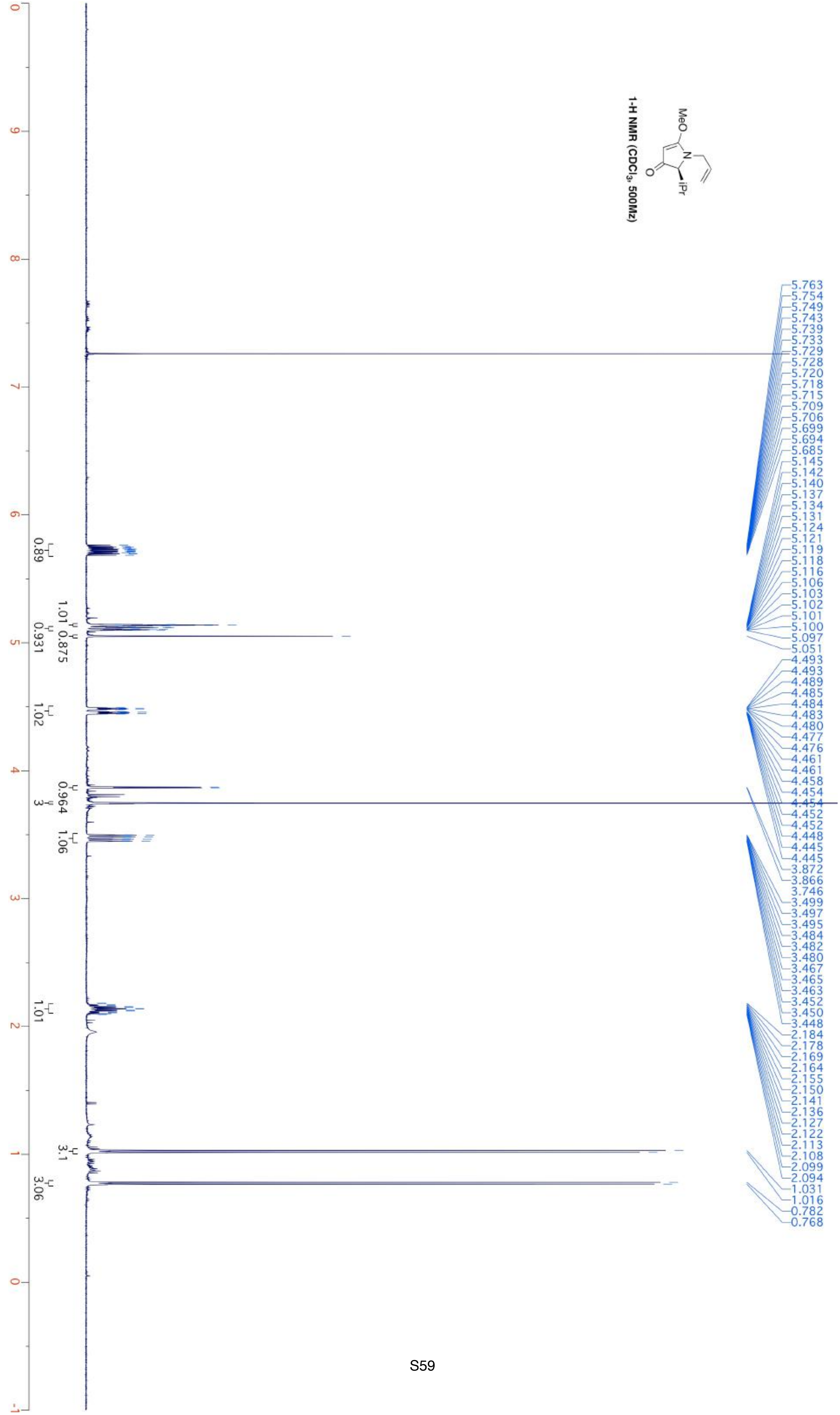
MeO
N
iPr
O
1-H NMR (CDCl3, 500Mz)
5.763
5.754
5.749
5.743
5.739
5.733
5.729
5.728
5.720
5.718
5.715
5.709
5.706
5.699
5.694
5.685
5.145
5.142
5.140
5.137
5.134
5.131
5.124
5.121
5.119
5.118
5.116
5.106
5.103
5.102
5.101
5.100
5.097
5.051
4.493
4.493
4.489
4.485
4.484
4.483
4.480
4.477
4.476
4.461
4.461
4.458
4.454
4.454
4.452
4.452
4.448
4.445
4.445
3.872
3.866
3.746
3.499
3.497
3.495
3.484
3.482
3.480
3.467
3.465
3.463
3.452
3.450
3.448
2.184
2.178
2.169
2.164
2.155
2.150
2.141
2.136
2.127
2.122
2.113
2.108
2.099
2.094
1.031
1.016
0.782
0.768
0.89
1.01
0.931
0.875
1.02
0.964
3
1.06
1.01
3.1
3.06
10
9
8
7
6
5
4
3
2
1
0
-1

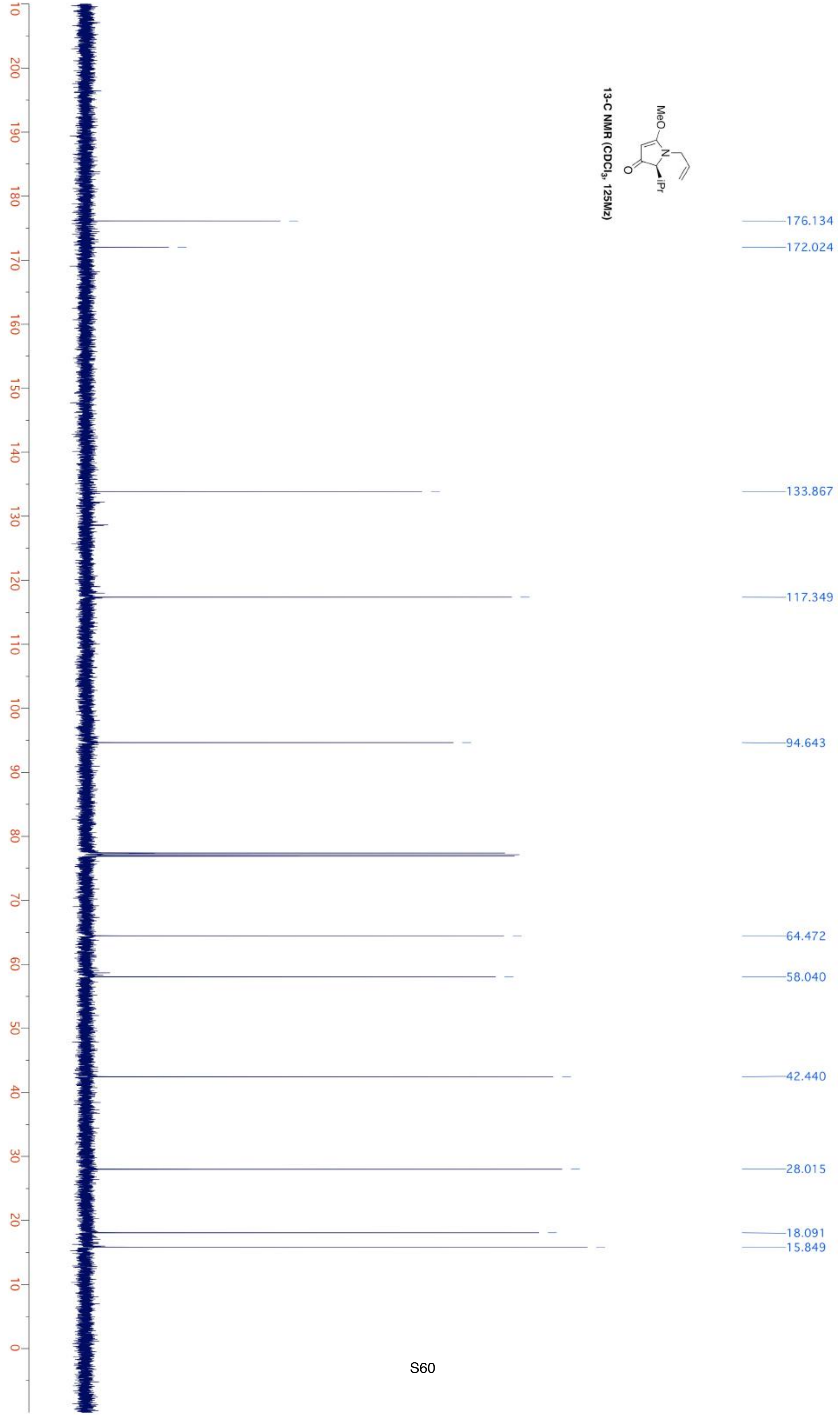

13-C NMR (CDCl$_3$, 125Mz)
MeO
N
iPr
O
176.134
172.024
133.867
117.349
94.643
64.472
58.040
42.440
28.015
18.091
15.849

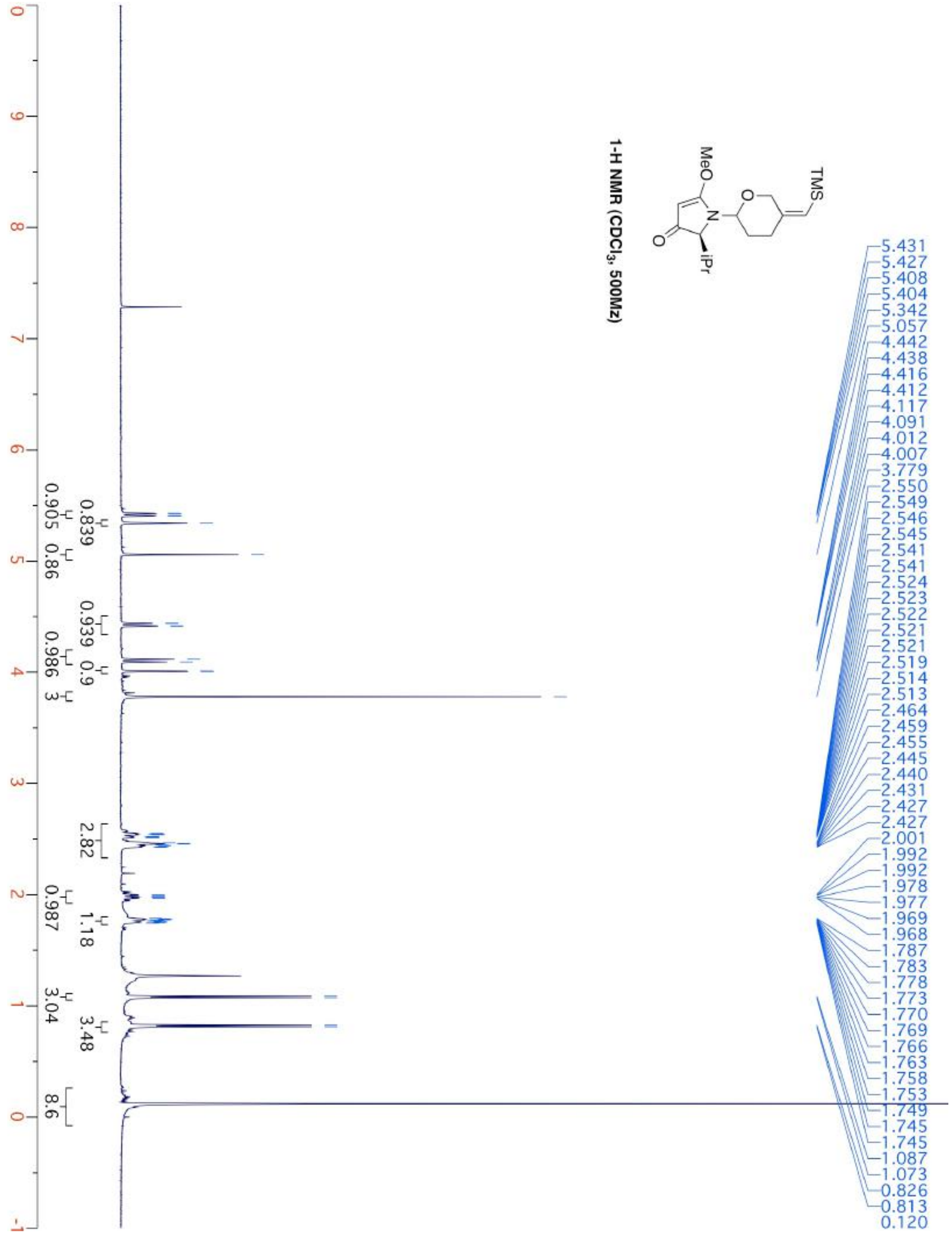
TMS
MeO
N
O
iPr
1-H NMR (CDCl3, 500Mz)
5.431
5.427
5.408
5.404
5.342
5.057
4.442
4.438
4.416
4.412
4.117
4.091
4.012
4.007
3.779
2.550
2.549
2.546
2.545
2.541
2.541
2.524
2.523
2.522
2.521
2.521
2.519
2.514
2.513
2.464
2.459
2.455
2.445
2.440
2.431
2.427
2.427
2.001
1.992
1.992
1.978
1.977
1.969
1.968
1.787
1.783
1.778
1.773
1.770
1.769
1.766
1.763
1.758
1.753
1.749
1.745
1.745
1.087
1.073
0.826
0.813
0.120
0.839
0.905
0.86
0.939
0.9
0.986
3
2.82
0.987
1.18
3.04
3.48
8.6

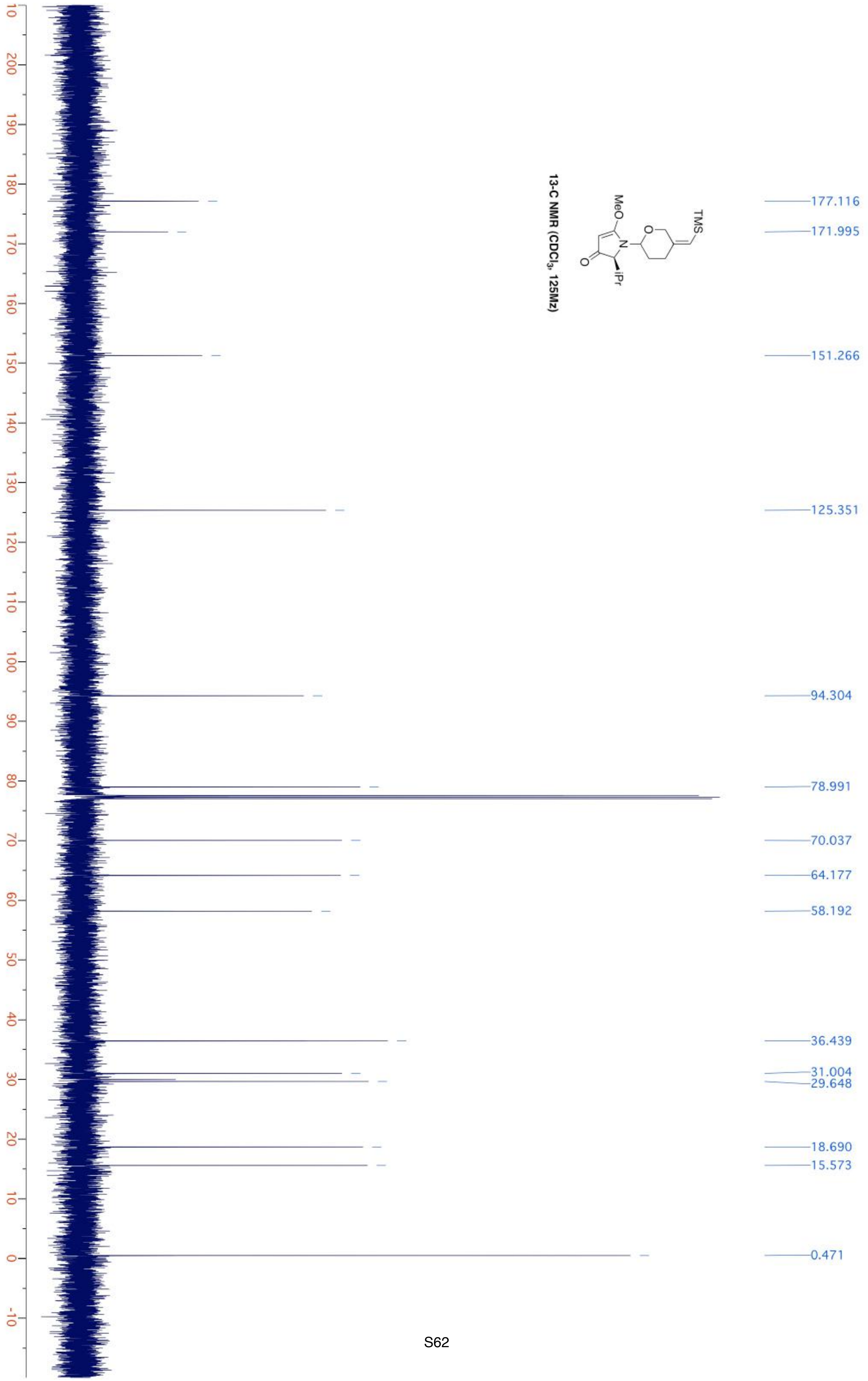
13-C NMR (CDCl3, 125Mz)
TMS
MeO
N
O
iPr
177.116
171.995
151.266
125.351
94.304
78.991
70.037
64.177
58.192
36.439
31.004
29.648
18.690
15.573
0.471

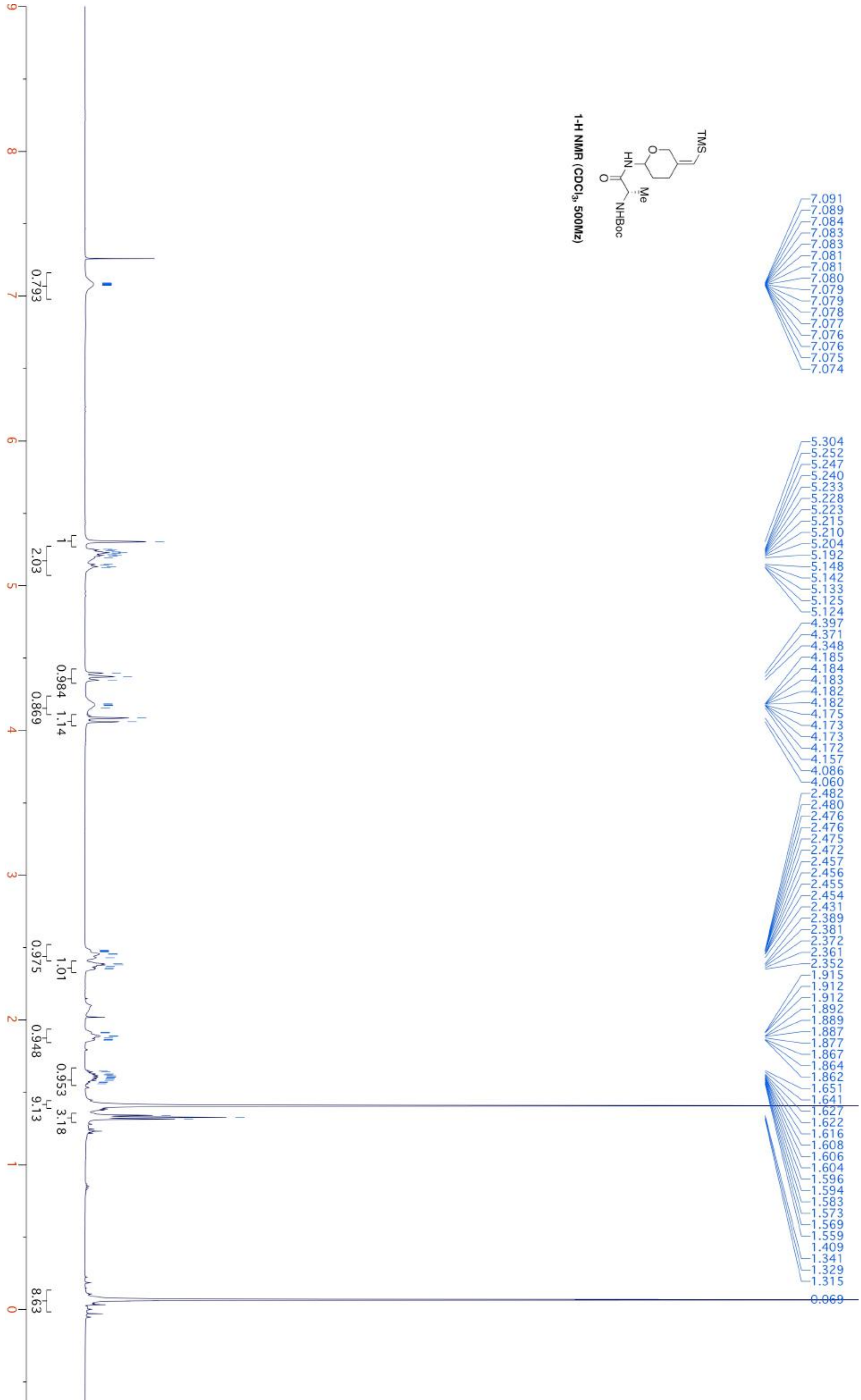
1-H NMR (CDCl3, 500Mz)
TMS
O
HN
O
Me
NHBoc
7.091 7.089 7.084 7.083 7.083 7.081 7.081 7.080 7.079 7.079 7.078 7.077 7.076 7.076 7.075 7.074
5.304 5.252 5.247 5.240 5.233 5.228 5.223 5.215 5.210 5.204 5.192 5.148 5.142 5.133 5.125 5.124
4.397 4.371 4.348 4.185 4.184 4.183 4.182 4.182 4.175 4.173 4.173 4.172 4.157 4.086 4.060
2.482 2.480 2.476 2.476 2.475 2.472 2.457 2.456 2.455 2.454 2.431 2.389 2.381 2.372 2.361 2.352
1.915 1.912 1.912 1.892 1.889 1.887 1.877 1.867 1.864 1.862 1.651 1.641 1.627 1.622 1.616 1.608 1.606 1.604 1.596 1.594 1.583 1.573 1.569 1.559 1.409 1.341 1.329 1.315
0.069
0.793
1
2.03
0.984
0.869
1.14
0.975
1.01
0.948
0.953
9.13
3.18
8.63
9 8 7 6 5 4 3 2 1 0 -1

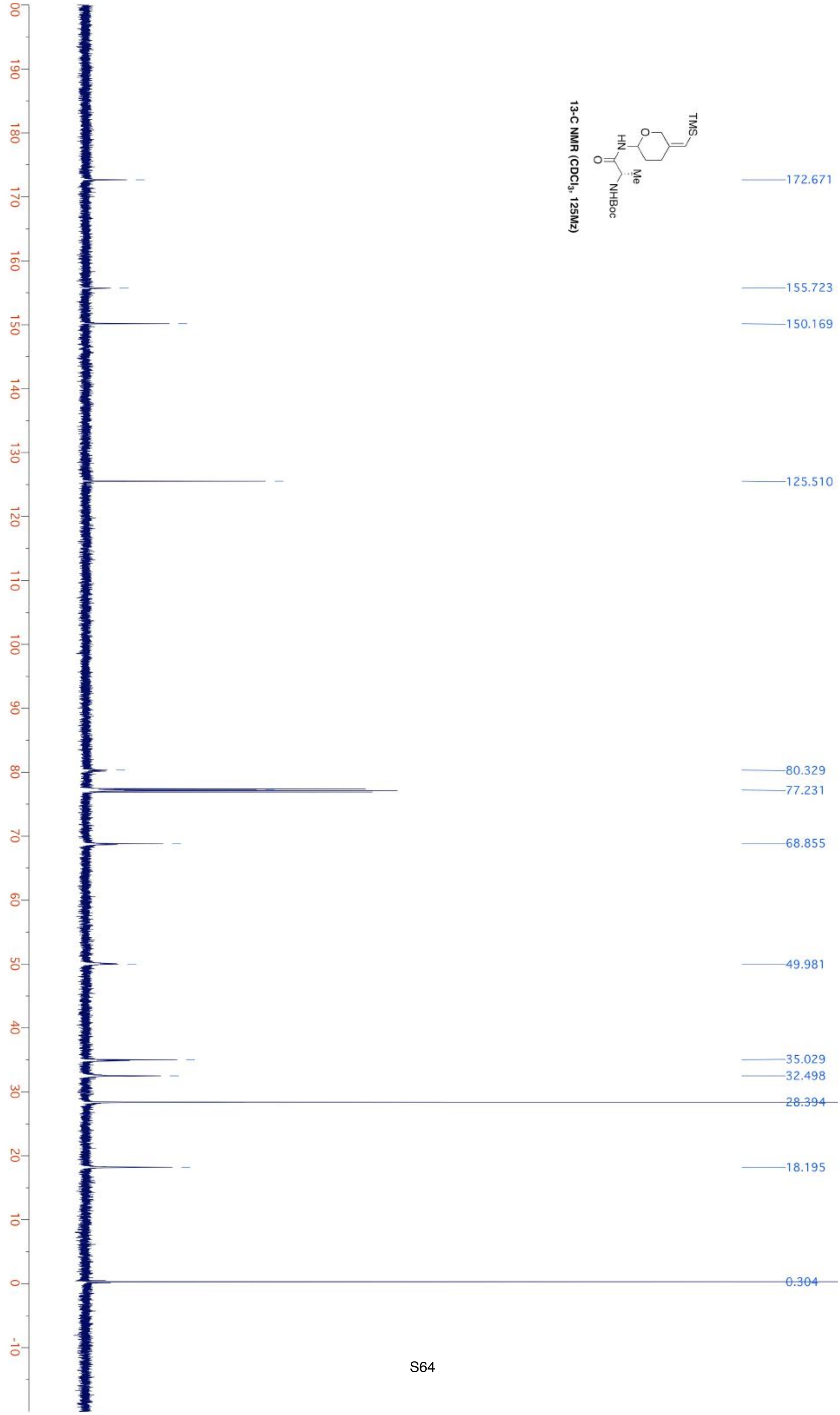
13-C NMR (CDCl3, 125Mz)
TMS
O
HN
O
Me
NHBoc
172.671
155.723
150.169
125.510
80.329
77.231
68.855
49.981
35.029
32.498
28.394
18.195
0.304
200
190
180
170
160
150
140
130
120
110
100
90
80
70
60
50
40
30
20
10
0
-10

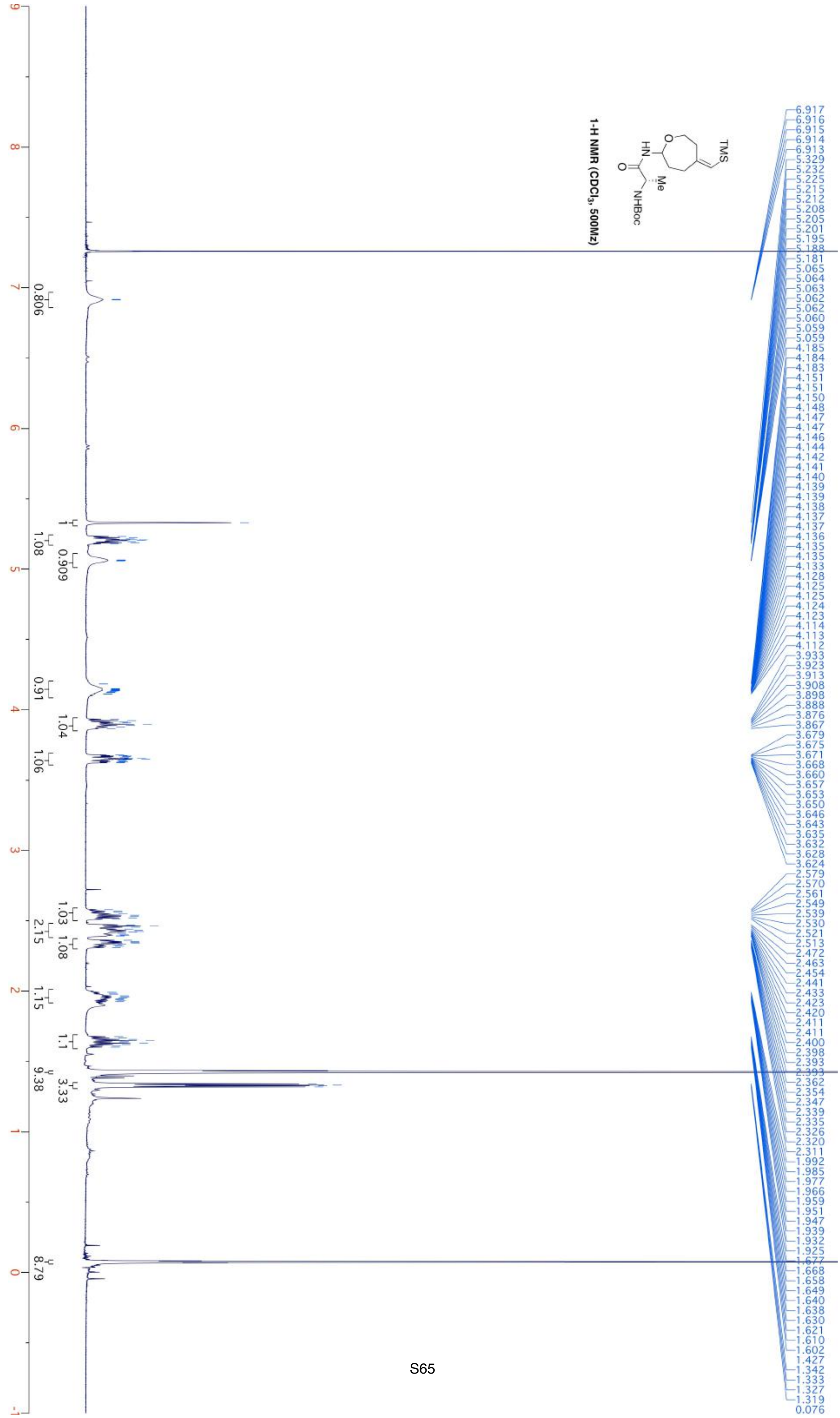
1-H NMR (CDCl3, 500Mz)
TMS
O
HN
Me
O
NHBoc
6.917
6.916
6.915
6.914
6.913
5.329
5.232
5.225
5.215
5.212
5.208
5.205
5.201
5.195
5.188
5.181
5.065
5.064
5.063
5.062
5.062
5.060
5.059
5.059
4.185
4.184
4.183
4.151
4.151
4.150
4.148
4.147
4.147
4.146
4.144
4.142
4.141
4.140
4.139
4.139
4.138
4.137
4.137
4.136
4.135
4.135
4.133
4.128
4.125
4.125
4.124
4.123
4.114
4.113
4.112
3.933
3.923
3.913
3.908
3.898
3.888
3.876
3.867
3.679
3.675
3.671
3.668
3.660
3.657
3.653
3.650
3.646
3.643
3.635
3.632
3.628
3.624
2.579
2.570
2.561
2.549
2.539
2.530
2.521
2.513
2.472
2.463
2.454
2.441
2.433
2.423
2.420
2.411
2.411
2.400
2.398
2.393
2.393
2.362
2.354
2.347
2.339
2.335
2.326
2.320
2.311
1.992
1.985
1.977
1.966
1.959
1.951
1.947
1.939
1.932
1.925
1.677
1.668
1.658
1.649
1.640
1.638
1.630
1.621
1.610
1.602
1.427
1.342
1.333
1.327
1.319
0.076
0.806
1
1.08
0.909
0.91
1.04
1.06
1.03
2.15
1.08
1.15
1.1
9.38
3.33
8.79
9
8
7
6
5
4
3
2
1
0
-1

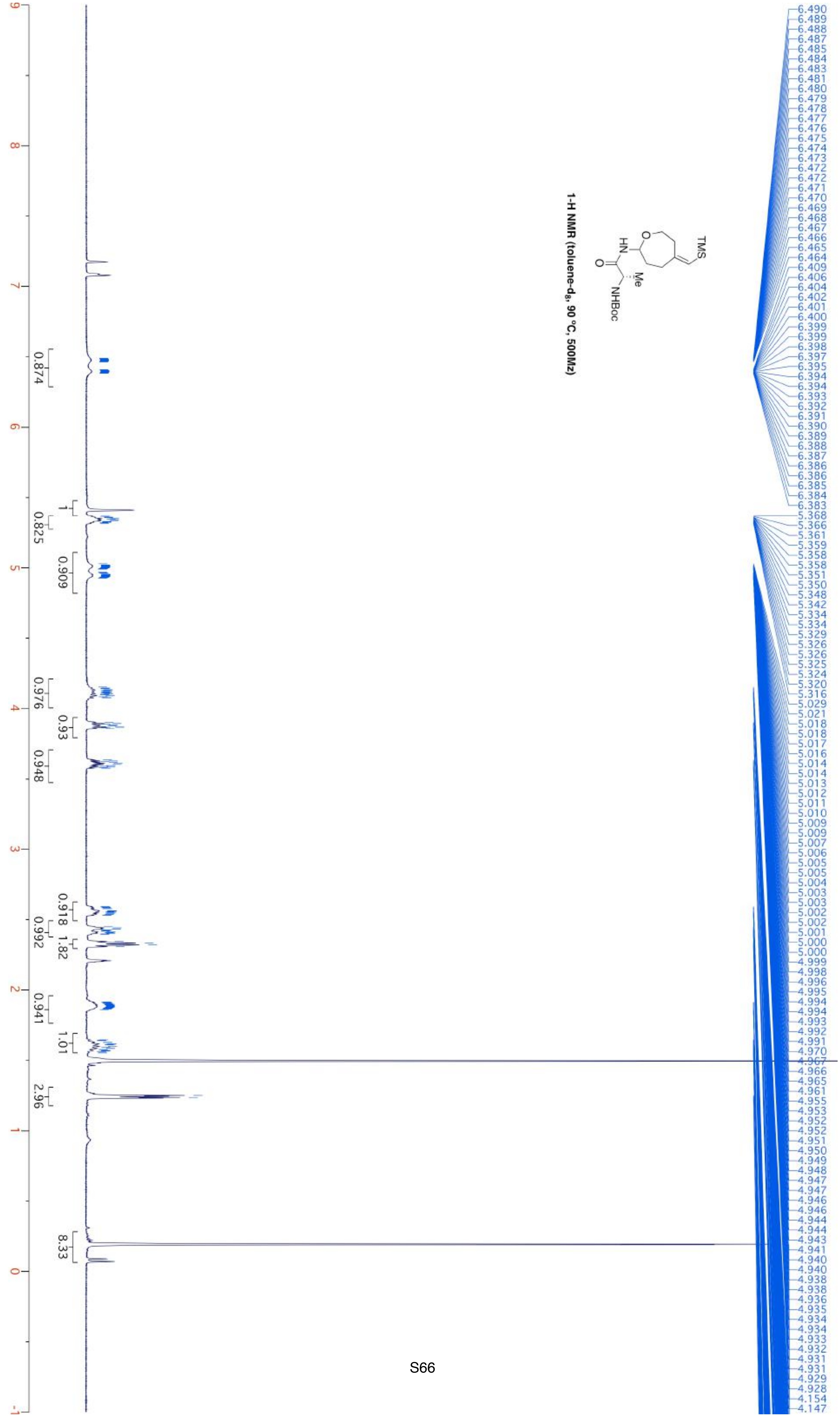
1-H NMR (toluene-d8, 90 °C, 500Mz)
TMS
O
HN
O
Me
NHBoc
6.490 6.489 6.488 6.487 6.485 6.484 6.483 6.481 6.480 6.479 6.478 6.477 6.476 6.475 6.474 6.473 6.472 6.472 6.471 6.470 6.469 6.468 6.467 6.466 6.465 6.464 6.409 6.406 6.404 6.402 6.401 6.400 6.399 6.399 6.398 6.397 6.395 6.394 6.394 6.393 6.392 6.391 6.390 6.389 6.388 6.387 6.386 6.386 6.385 6.384 6.383 5.368 5.366 5.361 5.359 5.358 5.358 5.351 5.350 5.348 5.342 5.334 5.334 5.329 5.326 5.326 5.325 5.324 5.320 5.316 5.029 5.021 5.018 5.018 5.017 5.016 5.014 5.014 5.013 5.012 5.011 5.010 5.009 5.009 5.007 5.006 5.005 5.005 5.004 5.003 5.003 5.002 5.002 5.001 5.000 5.000 4.999 4.998 4.996 4.995 4.994 4.994 4.993 4.992 4.991 4.970 4.967 4.966 4.965 4.961 4.955 4.953 4.952 4.952 4.951 4.950 4.949 4.948 4.947 4.947 4.946 4.946 4.944 4.944 4.943 4.941 4.940 4.940 4.938 4.938 4.936 4.935 4.934 4.934 4.933 4.932 4.931 4.931 4.929 4.928 4.154 4.147
0.874
1
0.825
0.909
0.976
0.93
0.948
0.918
0.992
1.82
0.941
1.01
2.96
8.33
9 8 7 6 5 4 3 2 1 0 -1

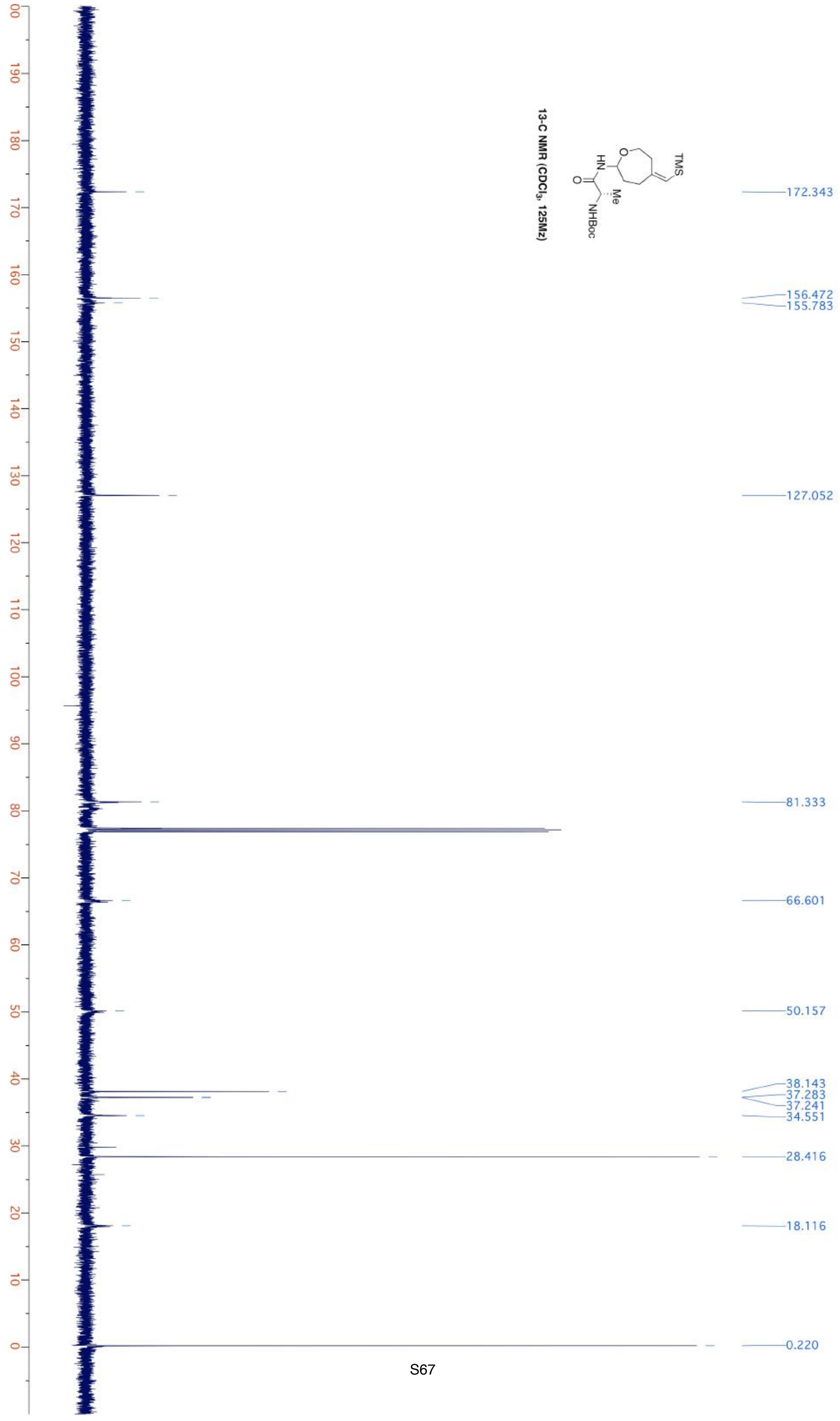
13-C NMR (CDCl3, 125Mz)
TMS
O
HN
O
Me
NHBoc
172.343
156.472
155.783
127.052
81.333
66.601
50.157
38.143
37.283
37.241
34.551
28.416
18.116
0.220
200
190
180
170
160
150
140
130
120
110
100
90
80
70
60
50
40
30
20
10
0

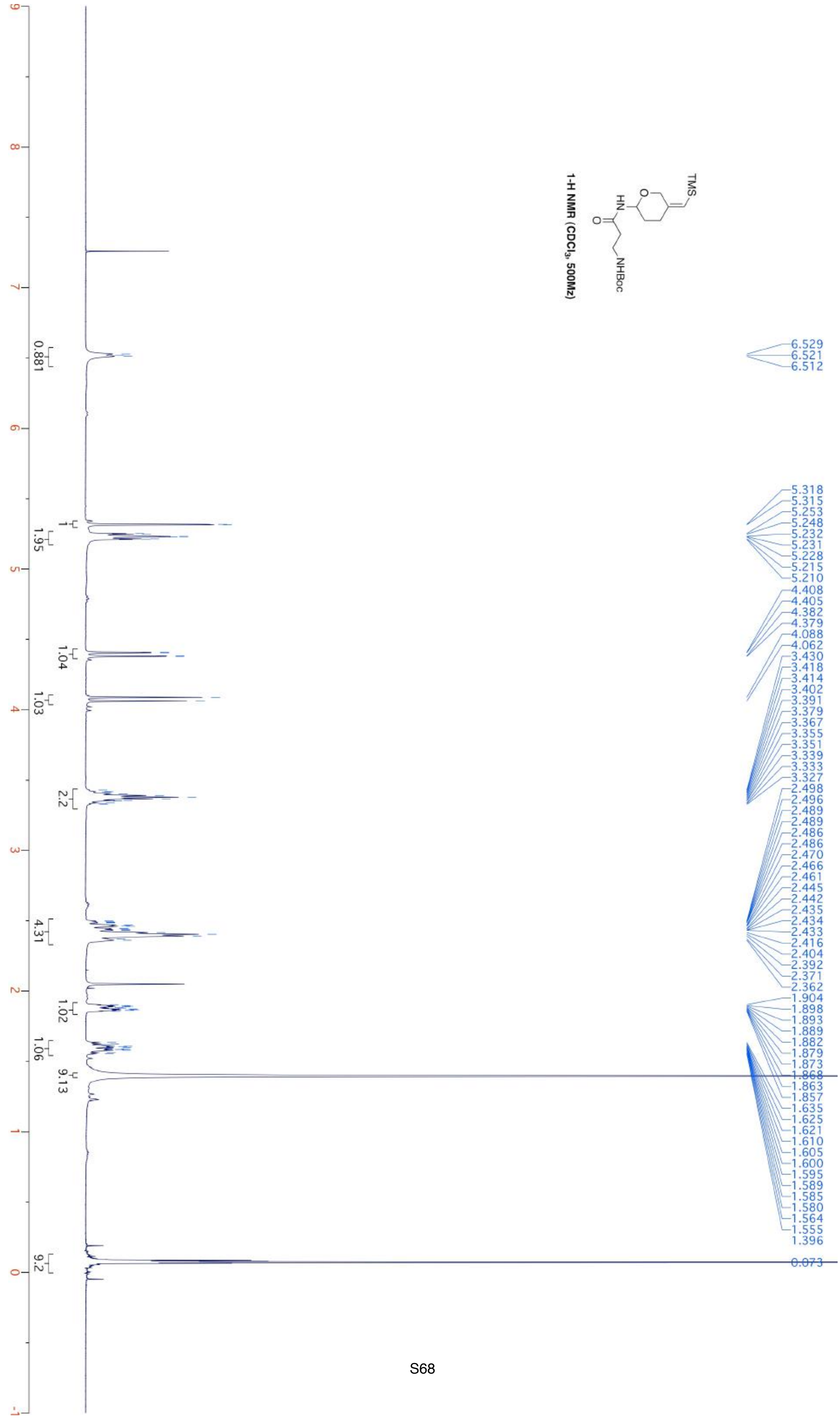
TMS
O
HN
O
NHBoc
1-H NMR (CDCl3, 500Mz)
6.529
6.521
6.512
5.318
5.315
5.253
5.248
5.232
5.231
5.228
5.215
5.210
4.408
4.405
4.382
4.379
4.088
4.062
3.430
3.418
3.414
3.402
3.391
3.379
3.367
3.355
3.351
3.339
3.333
3.327
2.498
2.496
2.489
2.489
2.486
2.486
2.470
2.466
2.461
2.445
2.442
2.435
2.434
2.433
2.416
2.404
2.392
2.371
2.362
1.904
1.898
1.893
1.889
1.882
1.879
1.873
1.868
1.863
1.857
1.635
1.625
1.621
1.610
1.605
1.600
1.595
1.589
1.585
1.580
1.564
1.555
1.396
0.073
0.881
1
1.95
1.04
1.03
2.2
4.31
1.02
1.06
9.13
9.2
9
8
7
6
5
4
3
2
1
0
-1

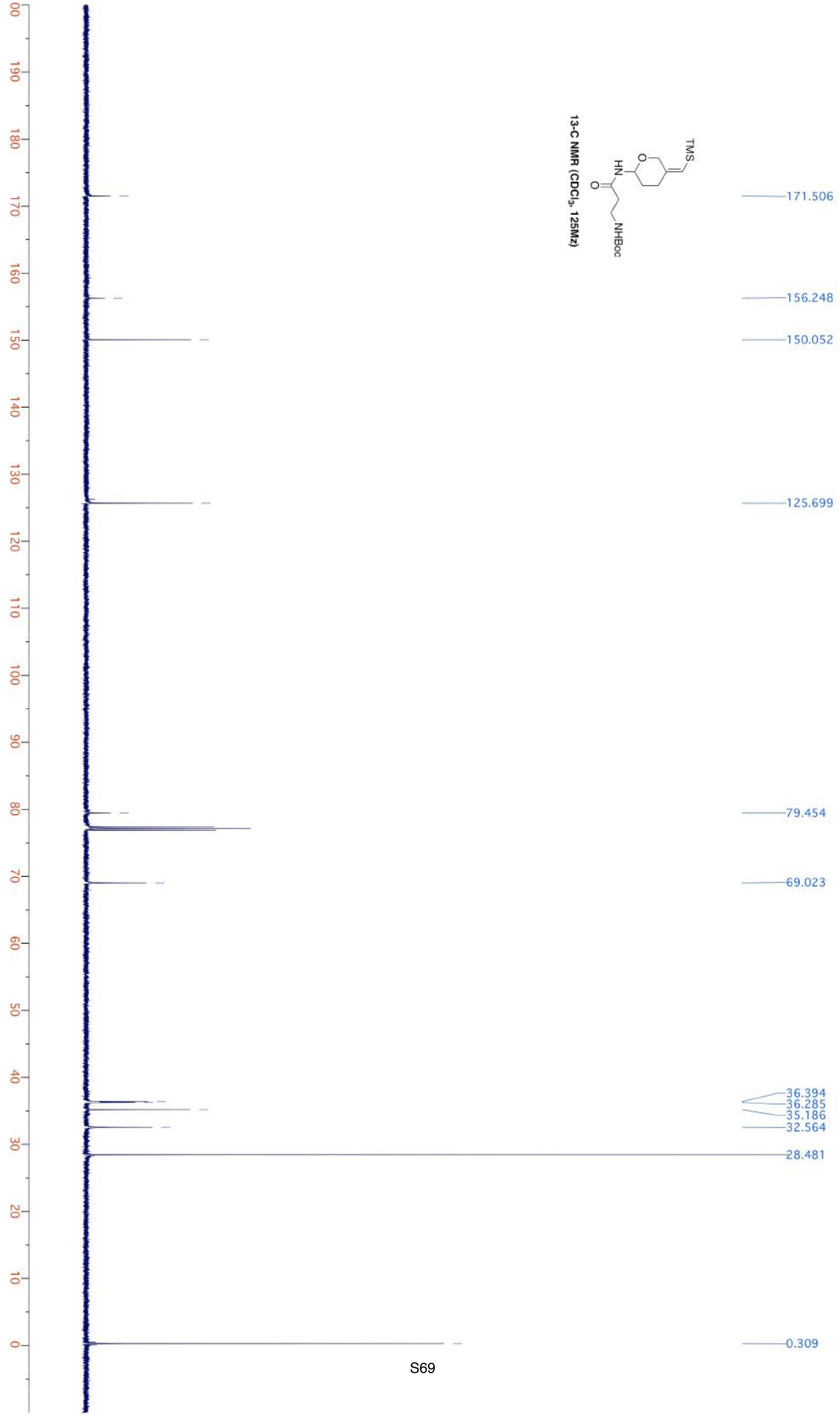
TMS
O
HN
O
NHBoc
13-C NMR (CDCl3, 125Mz)
171.506
156.248
150.052
125.699
79.454
69.023
36.394
36.285
35.186
32.564
28.481
0.309
190
180
170
160
150
140
130
120
110
100
90
80
70
60
50
40
30
20
10
0

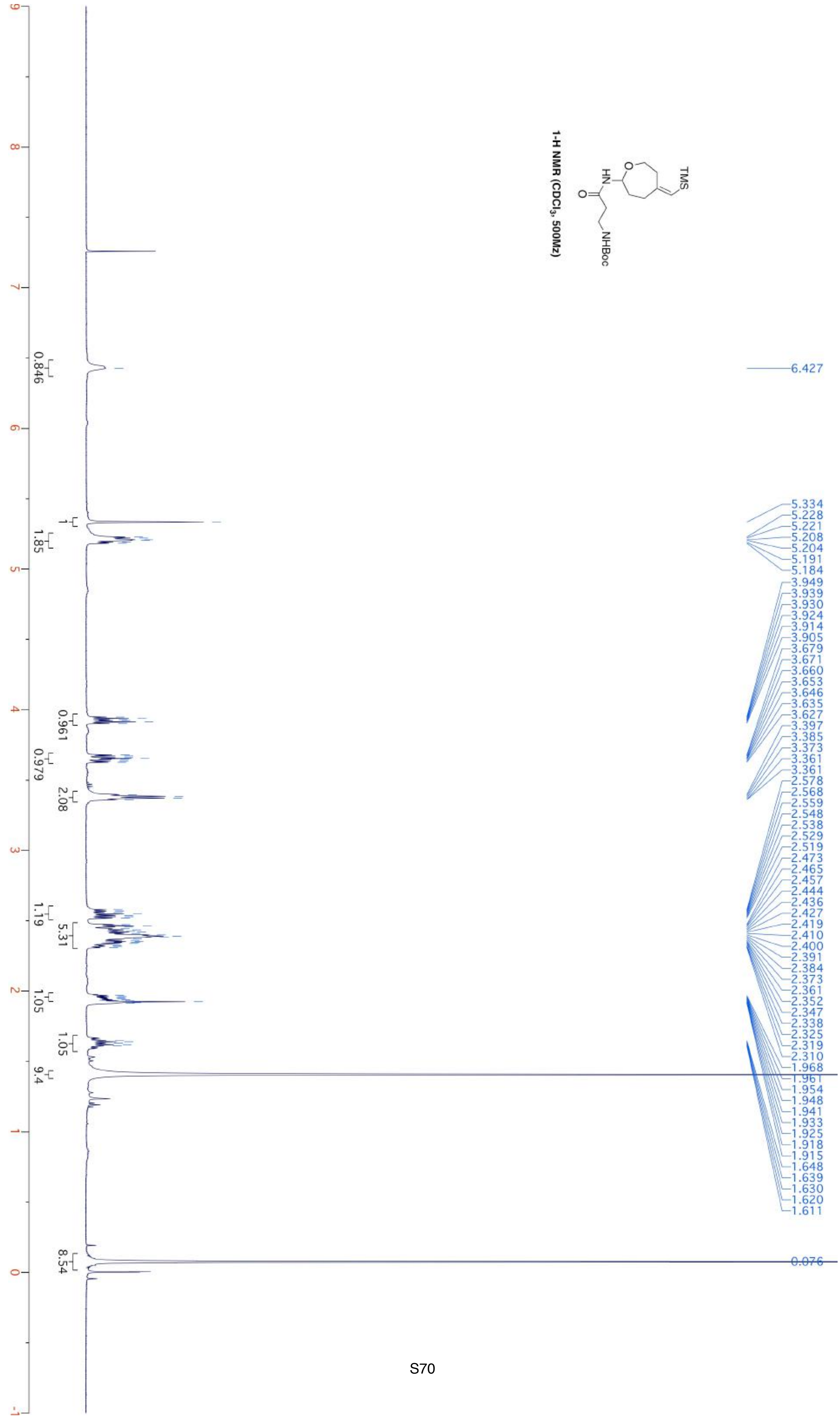
1-H NMR (CDCl$_3$, 500Mz)
TMS
O
HN
O
NHBoc
6.427
5.334
5.228
5.221
5.208
5.204
5.191
5.184
3.949
3.939
3.930
3.924
3.914
3.905
3.679
3.671
3.660
3.653
3.646
3.635
3.627
3.397
3.385
3.373
3.361
3.361
2.578
2.568
2.559
2.548
2.538
2.529
2.519
2.473
2.465
2.457
2.444
2.436
2.427
2.419
2.410
2.400
2.391
2.384
2.373
2.361
2.352
2.347
2.338
2.325
2.319
2.310
1.968
1.961
1.954
1.948
1.941
1.933
1.925
1.918
1.915
1.648
1.639
1.630
1.620
1.611
0.076
0.846
1
1.85
0.961
0.979
2.08
1.19
5.31
1.05
1.05
9.4
8.54
9
8
7
6
5
4
3
2
1
0
-1

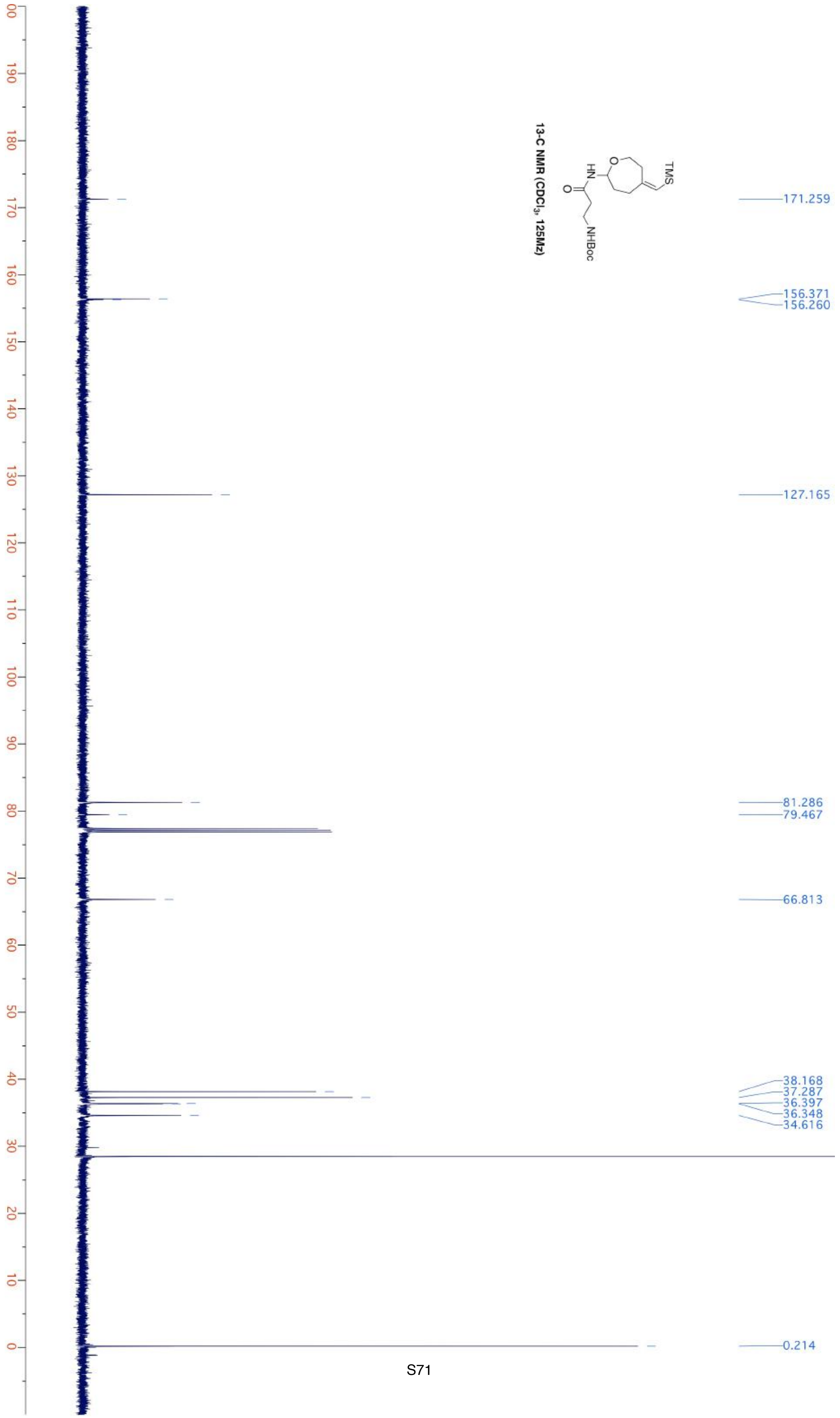
13-C NMR (CDCl3, 125Mz)
TMS
O
HN
O
NHBoc
171.259
156.371
156.260
127.165
81.286
79.467
66.813
38.168
37.287
36.397
36.348
34.616
0.214

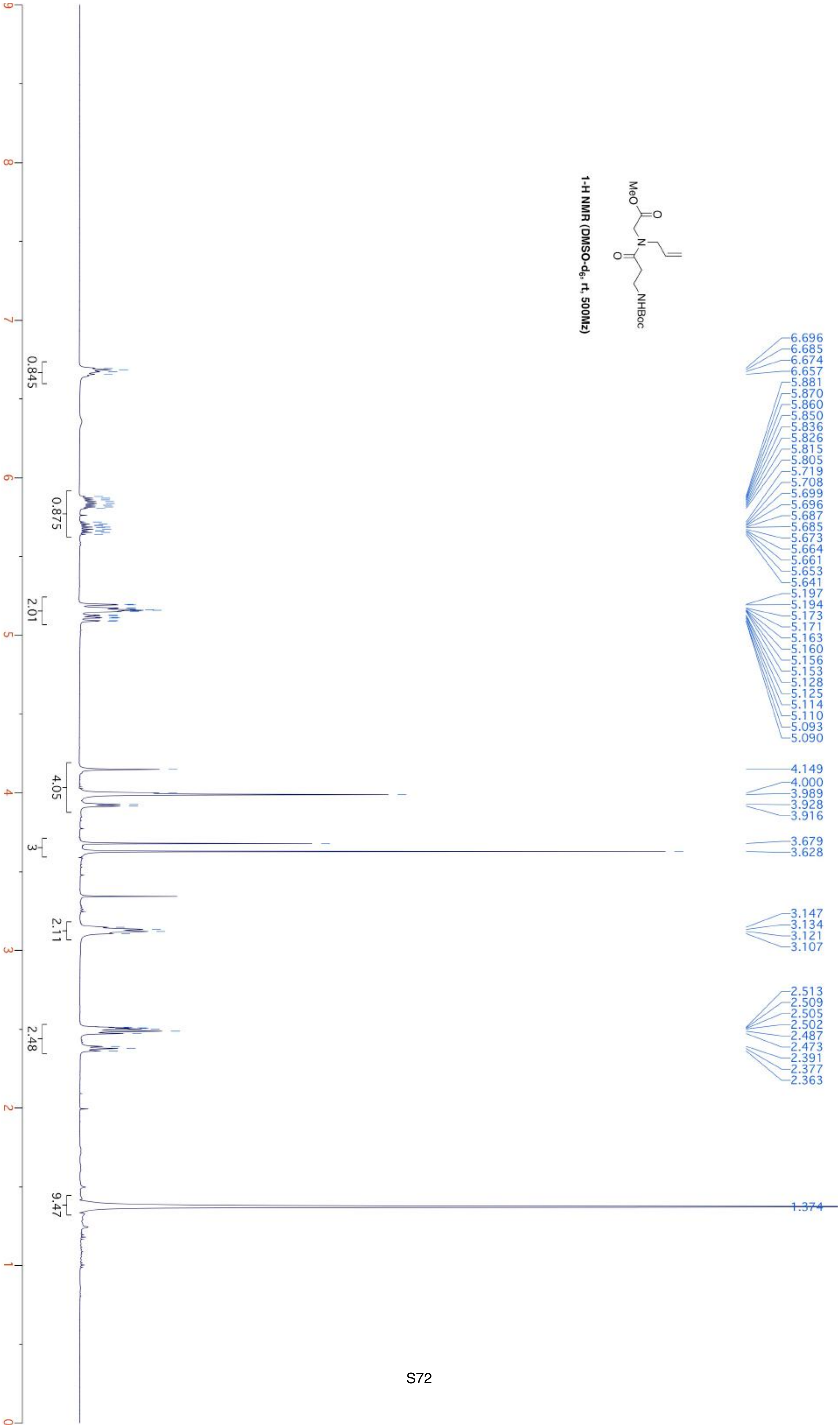
1-H NMR (DMSO-d6, rt, 500Mz)
MeO
O
N
O
NHBoc
6.696
6.685
6.674
6.657
5.881
5.870
5.860
5.850
5.836
5.826
5.815
5.805
5.719
5.708
5.699
5.696
5.687
5.685
5.673
5.664
5.661
5.653
5.641
5.197
5.194
5.173
5.171
5.163
5.160
5.156
5.153
5.128
5.125
5.114
5.110
5.093
5.090
4.149
4.000
3.989
3.928
3.916
3.679
3.628
3.147
3.134
3.121
3.107
2.513
2.509
2.505
2.502
2.487
2.473
2.391
2.377
2.363
1.374
0.845
0.875
2.01
4.05
3
2.11
2.48
9.47

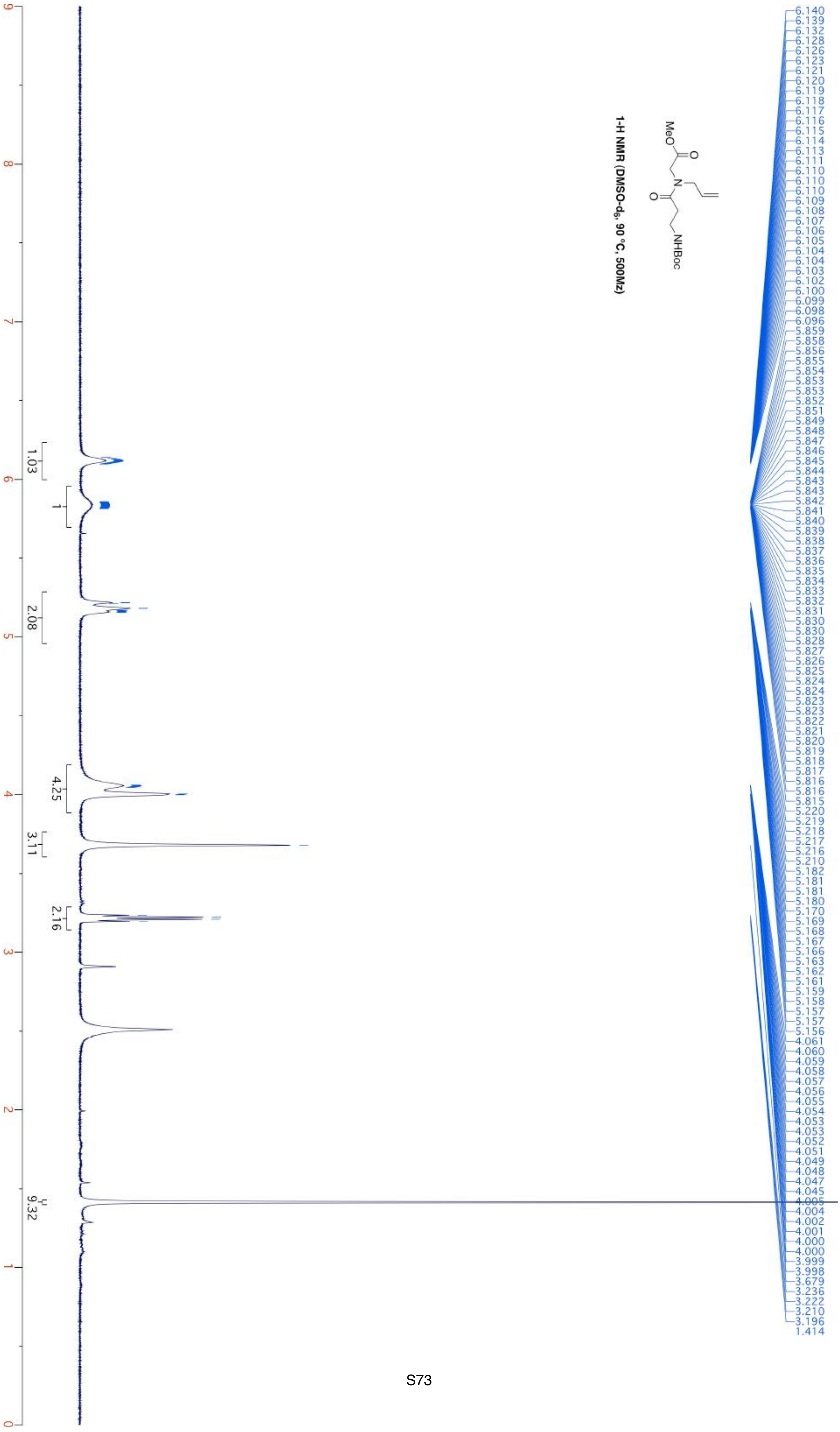
1-H NMR (DMSO-d6, 90 °C, 500Mz)
MeO
O
N
O
NHBoc

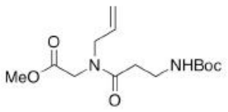

13-C NMR (DMSO-d_6, 90 °C, 125Mz)

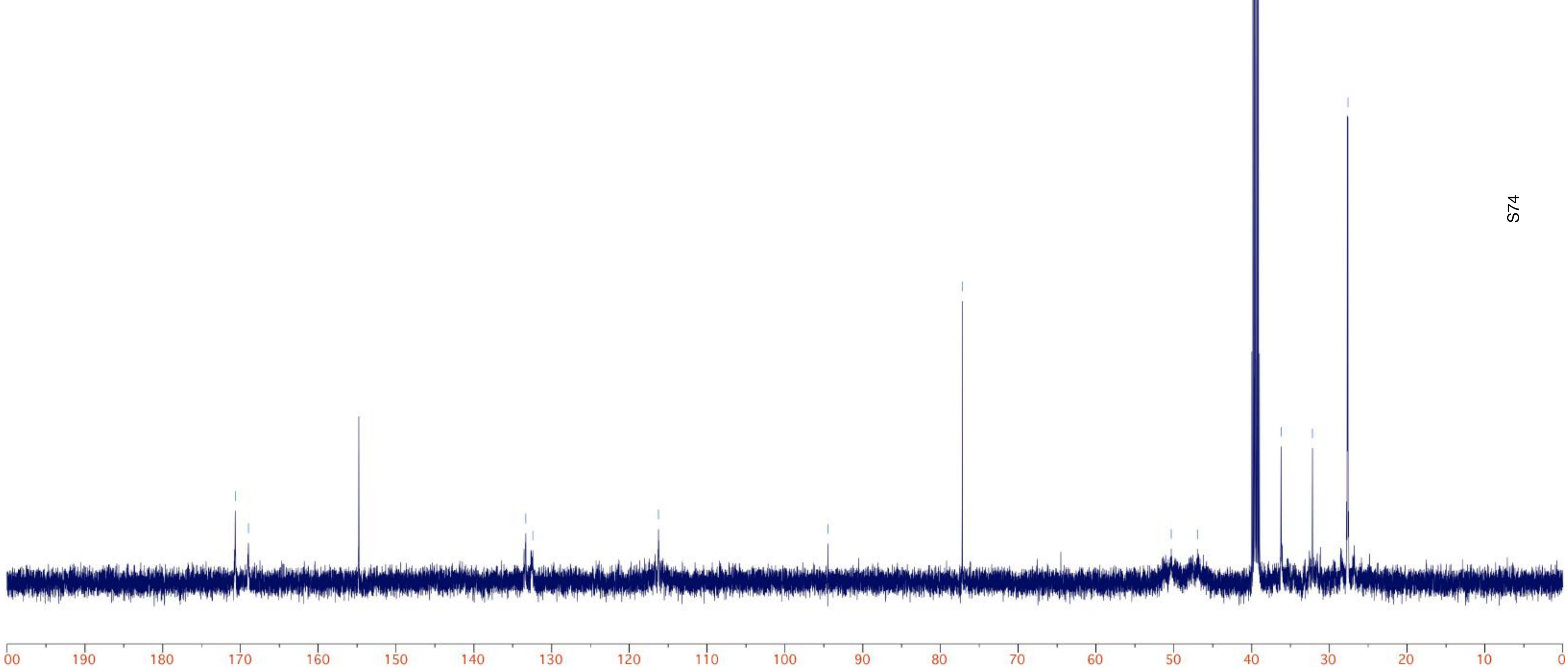

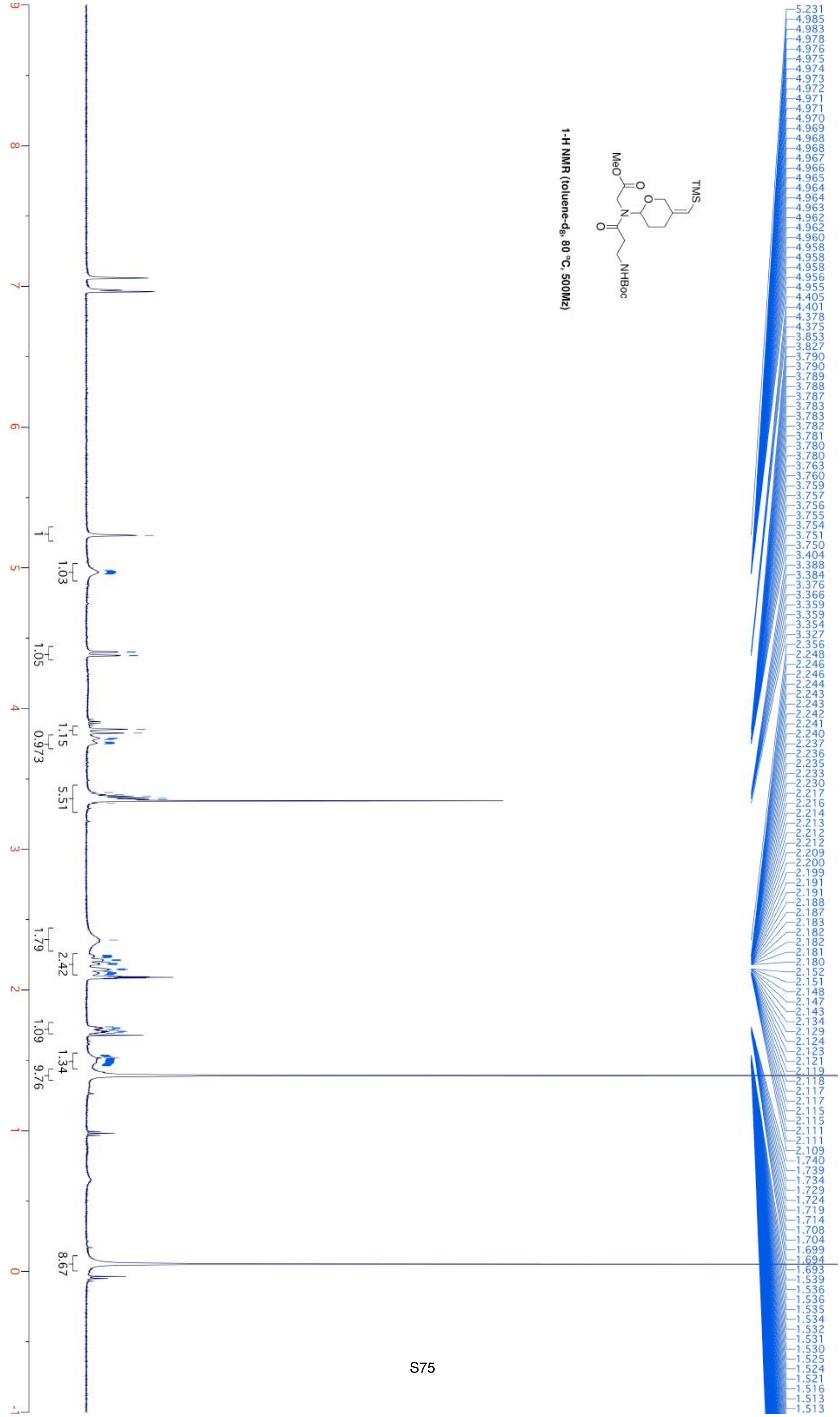
1-H NMR (toluene-d8, 80 °C, 500Mz)
TMS
MeO
NHBoc
N
O
5.231
4.985
4.983
4.978
4.976
4.975
4.974
4.973
4.972
4.971
4.971
4.970
4.969
4.968
4.968
4.967
4.966
4.965
4.964
4.964
4.963
4.962
4.962
4.960
4.958
4.958
4.958
4.956
4.955
4.405
4.401
4.378
4.375
3.853
3.827
3.790
3.790
3.789
3.788
3.787
3.783
3.783
3.782
3.781
3.780
3.780
3.763
3.760
3.759
3.757
3.756
3.755
3.754
3.751
3.750
3.404
3.388
3.384
3.376
3.366
3.359
3.359
3.354
3.327
2.356
2.248
2.246
2.246
2.244
2.243
2.243
2.242
2.241
2.240
2.237
2.236
2.235
2.233
2.230
2.217
2.216
2.214
2.213
2.212
2.212
2.209
2.200
2.199
2.191
2.191
2.188
2.187
2.183
2.182
2.182
2.181
2.180
2.152
2.151
2.148
2.147
2.143
2.134
2.129
2.124
2.123
2.121
2.119
2.118
2.117
2.117
2.115
2.115
2.111
2.111
2.109
1.740
1.739
1.734
1.729
1.724
1.719
1.714
1.708
1.704
1.699
1.694
1.693
1.539
1.536
1.536
1.535
1.534
1.532
1.531
1.530
1.525
1.524
1.521
1.516
1.513
1.513
1
1.03
1.05
1.15
0.973
5.51
1.79
2.42
1.09
1.34
9.76
8.67
9
8
7
6
5
4
3
2
1
0
-1

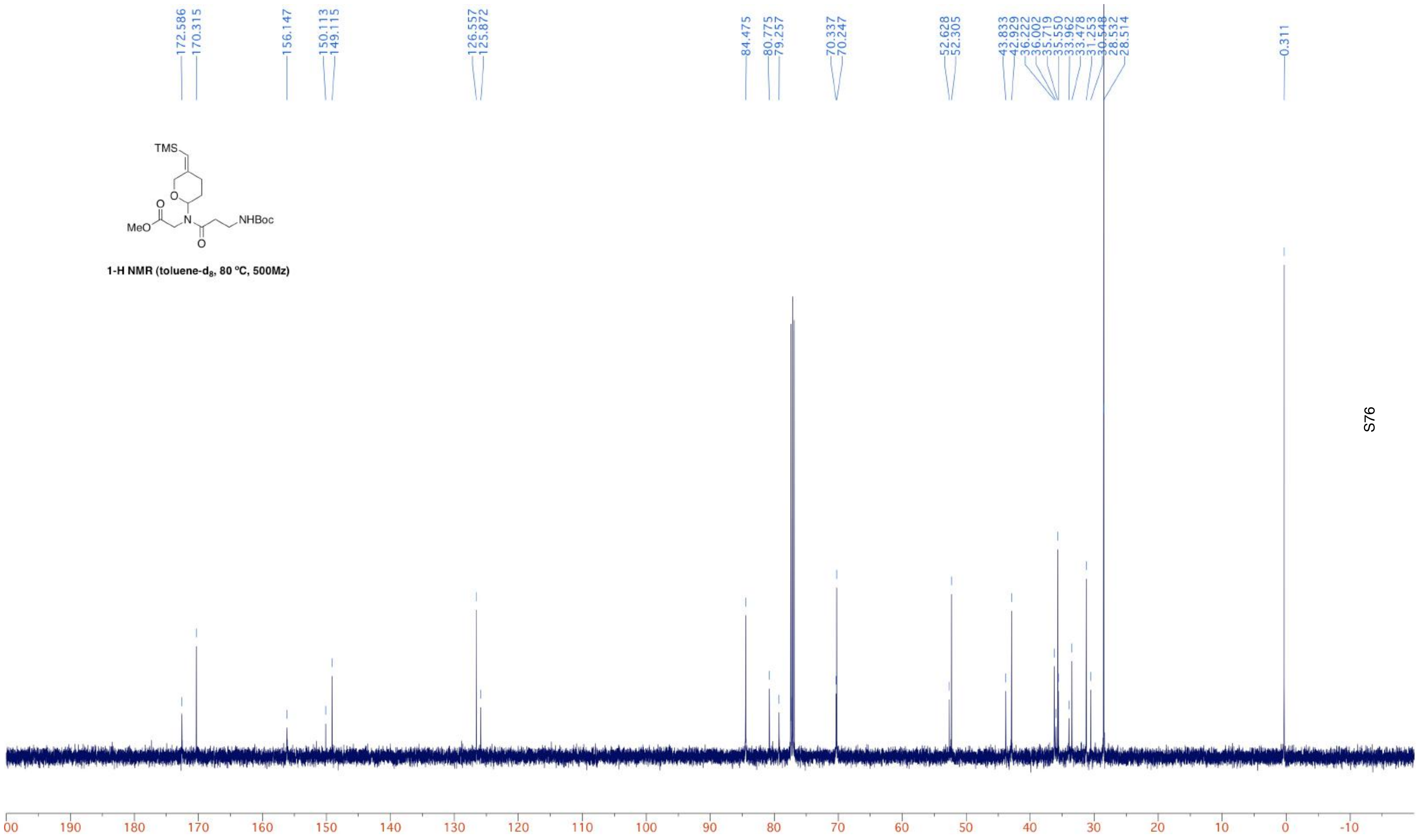
TMS
MeO
O
N
NHBoc
1-H NMR (toluene-d_8, 80 °C, 500Mz)
172.586
170.315
156.147
150.113
149.115
126.557
125.872
84.475
80.775
79.257
70.337
70.247
52.628
52.305
43.833
42.929
36.222
36.002
35.719
35.550
33.962
33.478
31.253
30.548
28.532
28.514
0.311
190
180
170
160
150
140
130
120
110
100
90
80
70
60
50
40
30
20
10
0
-10

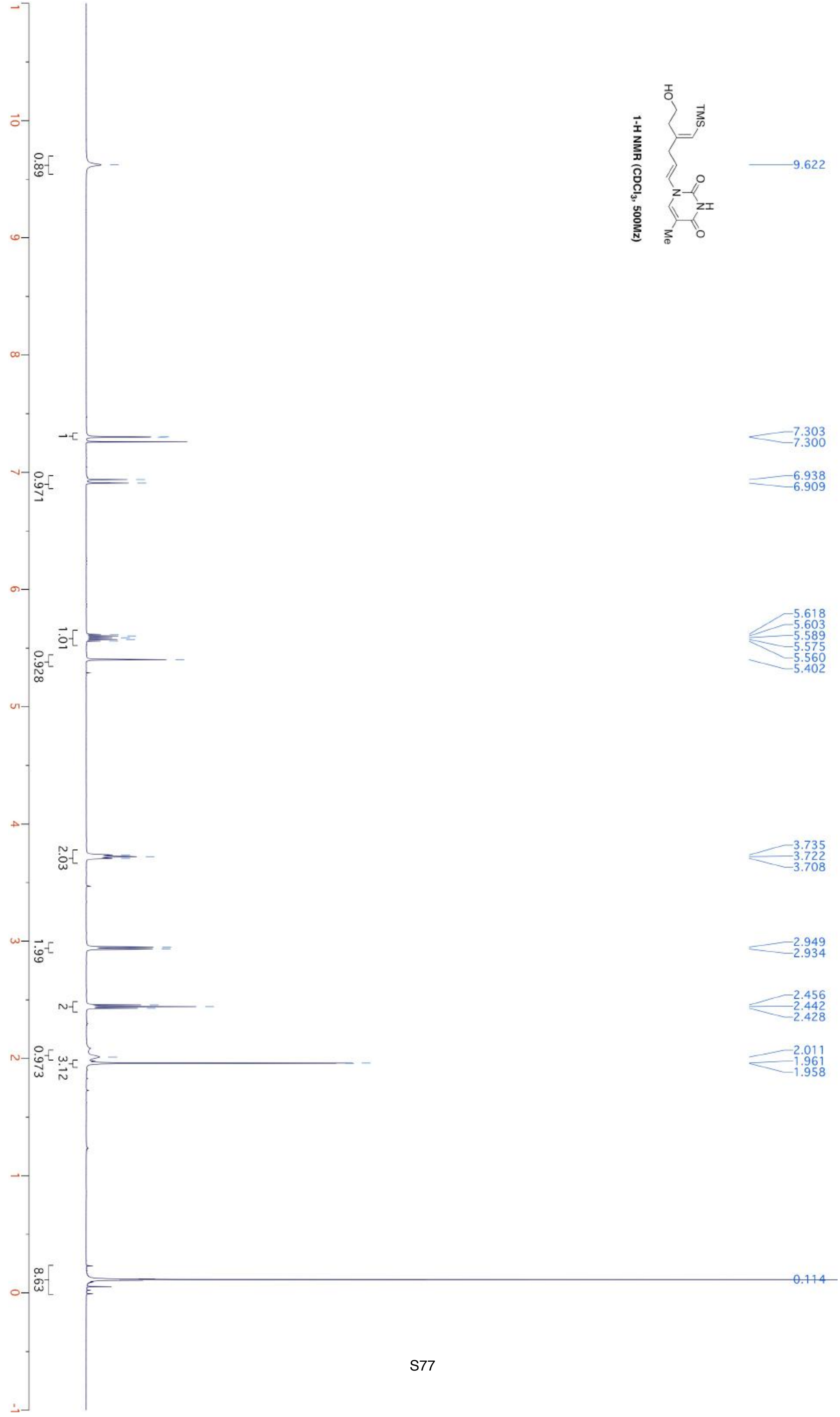
1-H NMR (CDCl3, 500Mz)
TMS
HO
Me
9.622
7.303
7.300
6.938
6.909
5.618
5.603
5.589
5.575
5.560
5.402
3.735
3.722
3.708
2.949
2.934
2.456
2.442
2.428
2.011
1.961
1.958
0.114
0.89
1
0.971
1.01
0.928
2.03
1.99
2
0.973
3.12
8.63

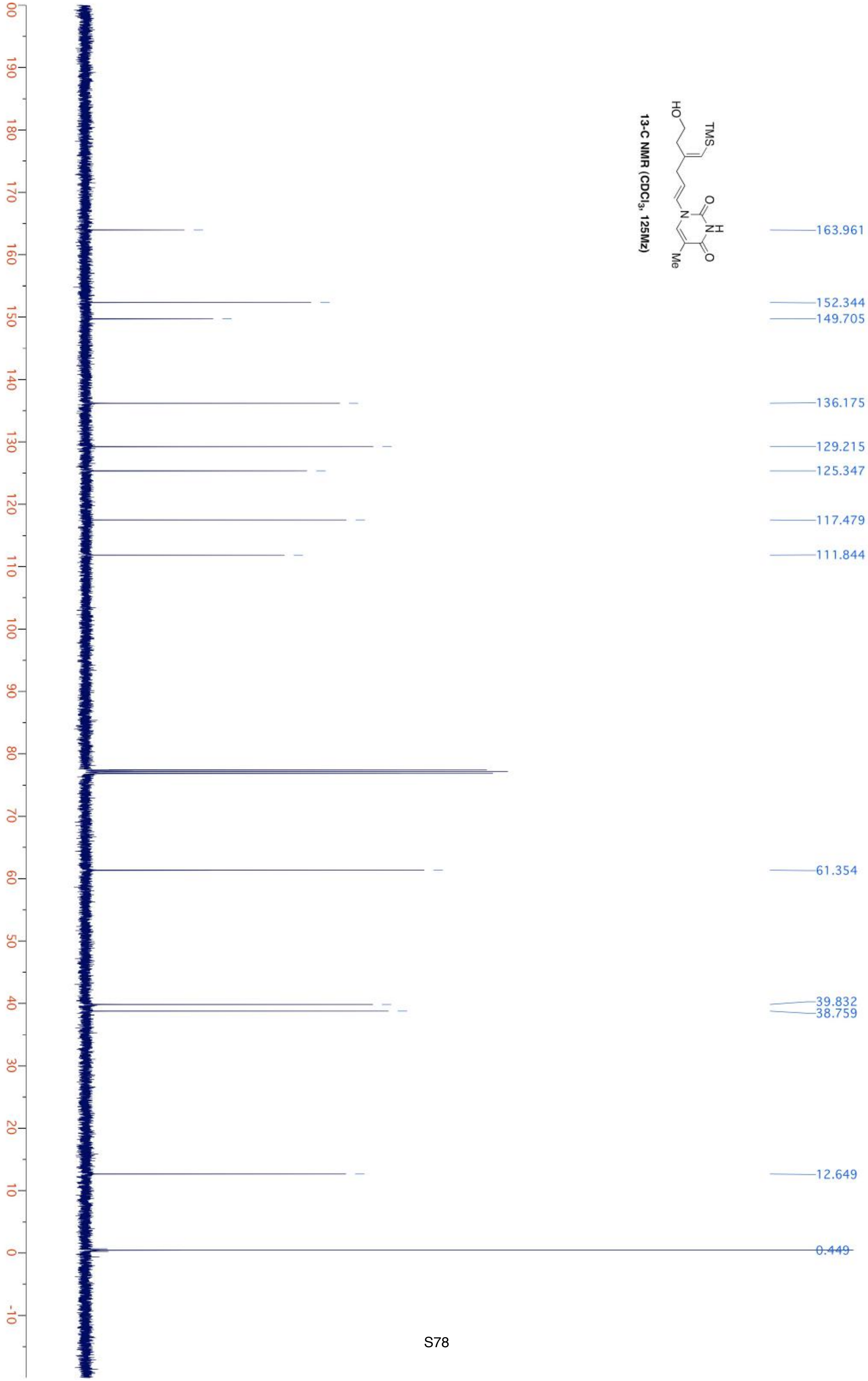
TMS
HO
N
H
O
O
N
Me
13-C NMR (CDCl3, 125Mz)
163.961
152.344
149.705
136.175
129.215
125.347
117.479
111.844
61.354
39.832
38.759
12.649
0.449
190
180
170
160
150
140
130
120
110
100
90
80
70
60
50
40
30
20
10
0
-10

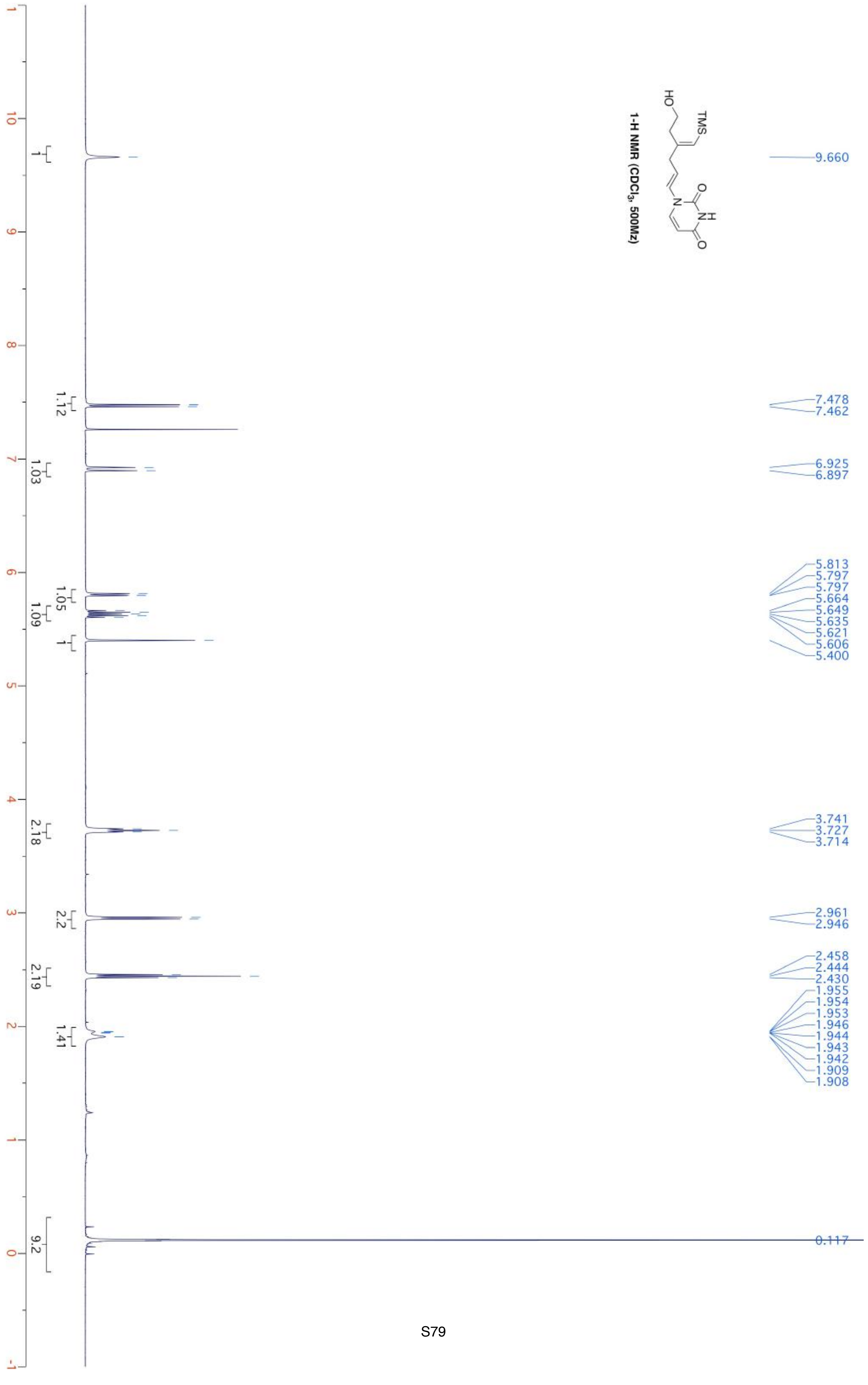
1-H NMR (CDCl$_3$, 500Mz)
TMS
HO
O
H
N
O
N
9.660
7.478
7.462
6.925
6.897
5.813
5.797
5.797
5.664
5.649
5.635
5.621
5.606
5.400
3.741
3.727
3.714
2.961
2.946
2.458
2.444
2.430
1.955
1.954
1.953
1.946
1.944
1.943
1.942
1.909
1.908
0.117
1
1.12
1.03
1.05
1.09
1
2.18
2.2
2.19
1.41
9.2
11
10
9
8
7
6
5
4
3
2
1
0
-1

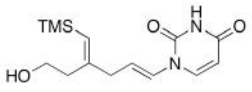

13-C NMR ($CDCl_3$, 125Mz)

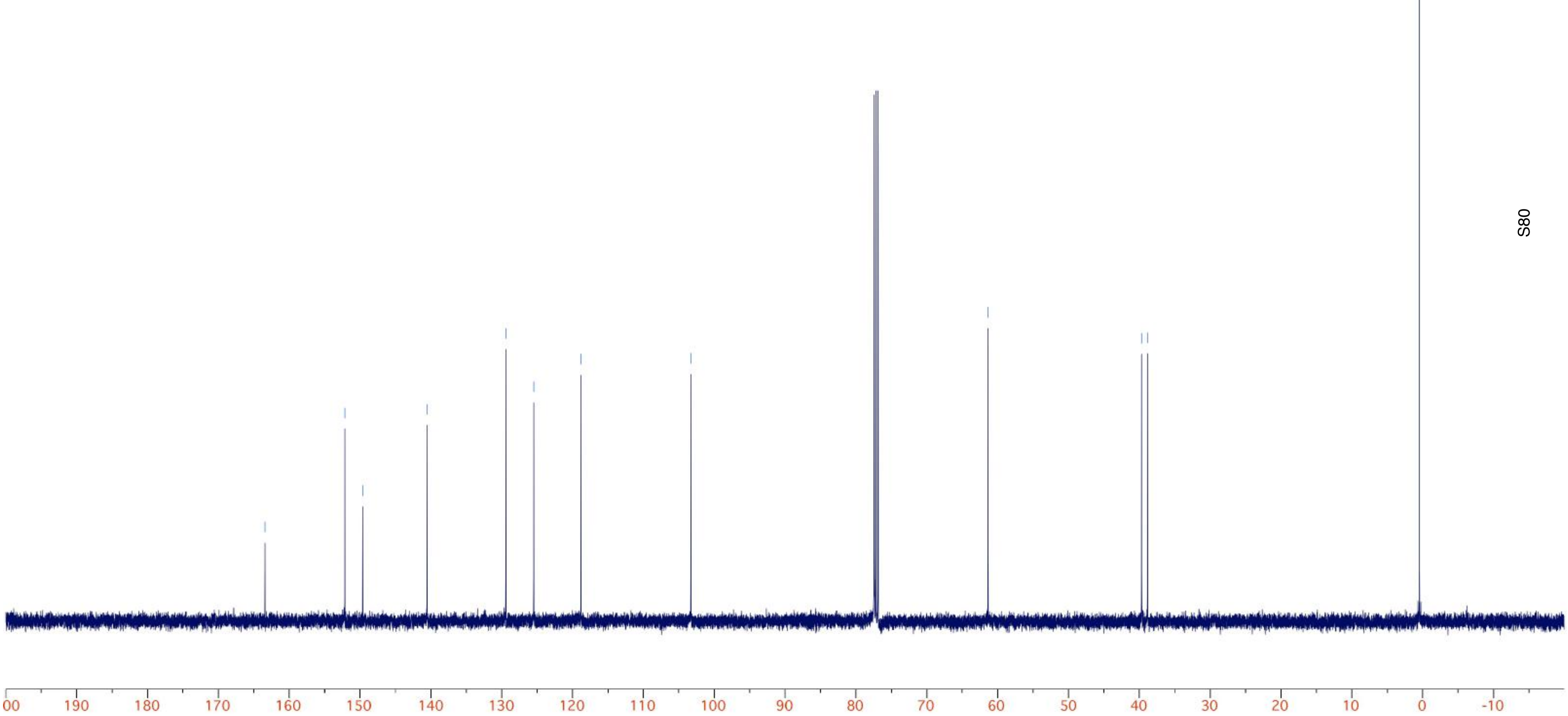

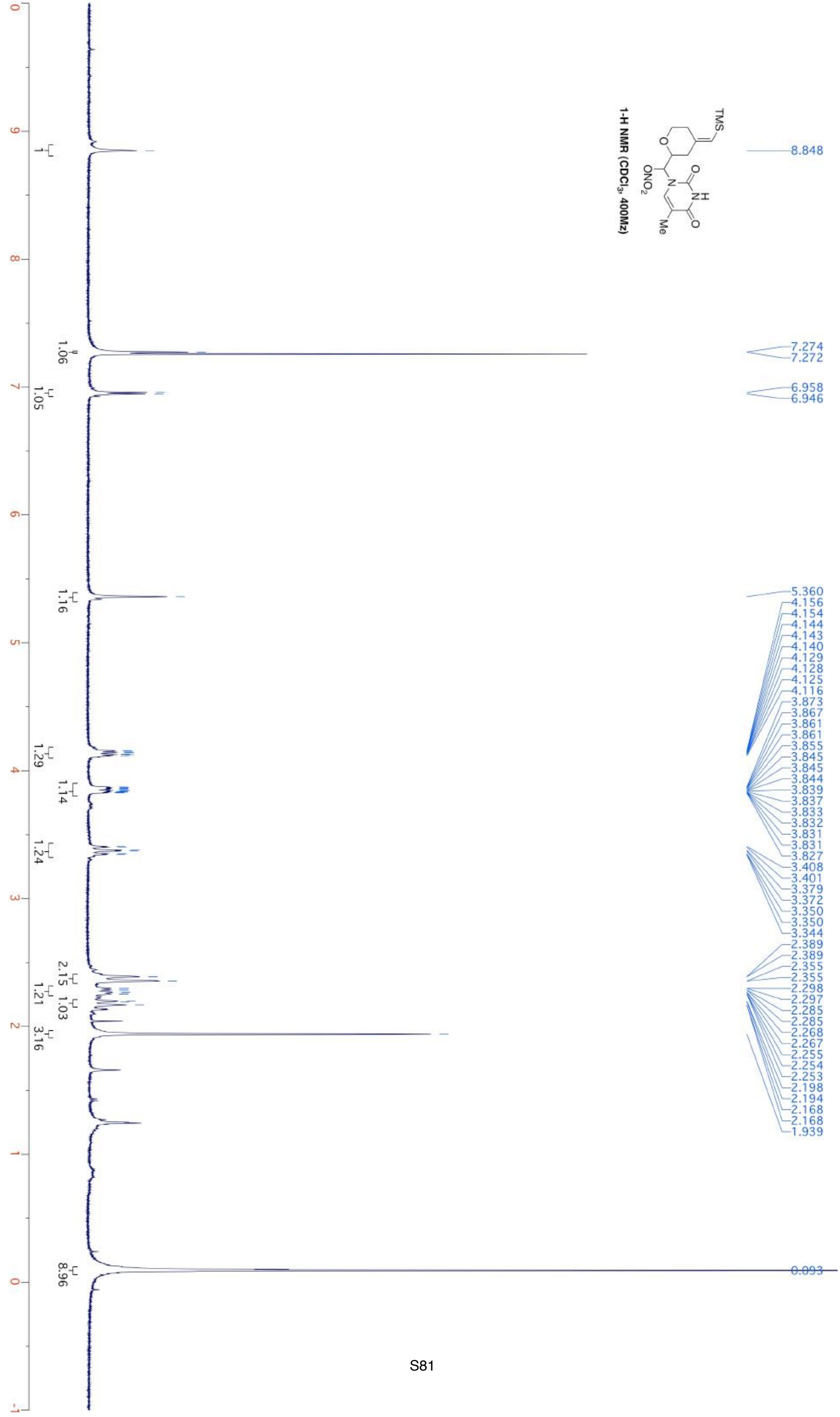

TMS
O
O
H
N
O
N
Me
ONO2
1-H NMR (CDCl3, 400Mz)
8.848
7.274
7.272
6.958
6.946
5.360
4.156
4.154
4.144
4.143
4.140
4.129
4.128
4.125
4.116
3.873
3.867
3.861
3.861
3.855
3.845
3.845
3.844
3.839
3.837
3.833
3.832
3.831
3.831
3.827
3.408
3.401
3.379
3.372
3.350
3.350
3.344
2.389
2.389
2.355
2.355
2.298
2.297
2.285
2.285
2.268
2.267
2.255
2.254
2.253
2.198
2.194
2.168
2.168
1.939
0.093
1
1.06
1.05
1.16
1.29
1.14
1.24
2.15
1.21
1.03
3.16
8.96
10
9
8
7
6
5
4
3
2
1
0
-1

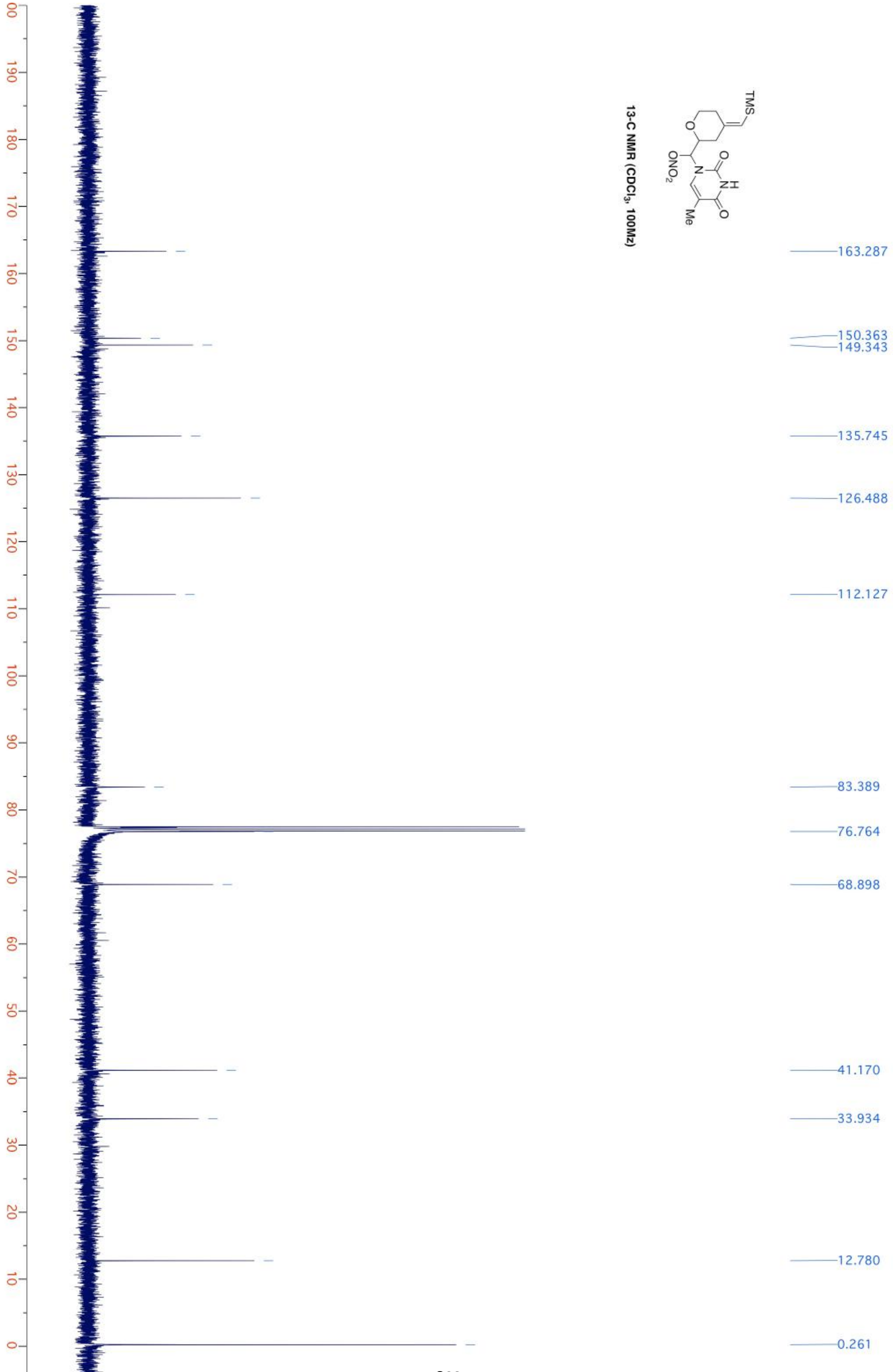
TMS
O
N
H
Me
ONO2
13-C NMR (CDCl3, 100Mz)
163.287
150.363
149.343
135.745
126.488
112.127
83.389
76.764
68.898
41.170
33.934
12.780
0.261
200
190
180
170
160
150
140
130
120
110
100
90
80
70
60
50
40
30
20
10
0

STANDARD PROTON PARAMETERS

Pulse Sequence: gCOSY
Solvent: CDCl3
Temp. 25.0 C / 298.1 K
User: 1-15-87
INOVA-600 "nmr4"

Relax. delay 1.500 sec
Acq. time 0.164 sec
Width 6255.4 Hz
2D Width 6255.4 Hz
2 repetitions
200 increments
OBSERVE H1, 599.7972910 MHz
DATA PROCESSING
Sq. sine bell 0.082 sec
F1 DATA PROCESSING
Sq. sine bell 0.014 sec
FT size 2048 x 2048
Total time 11 min, 43 sec

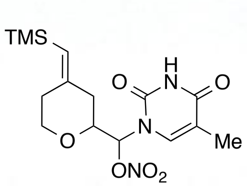

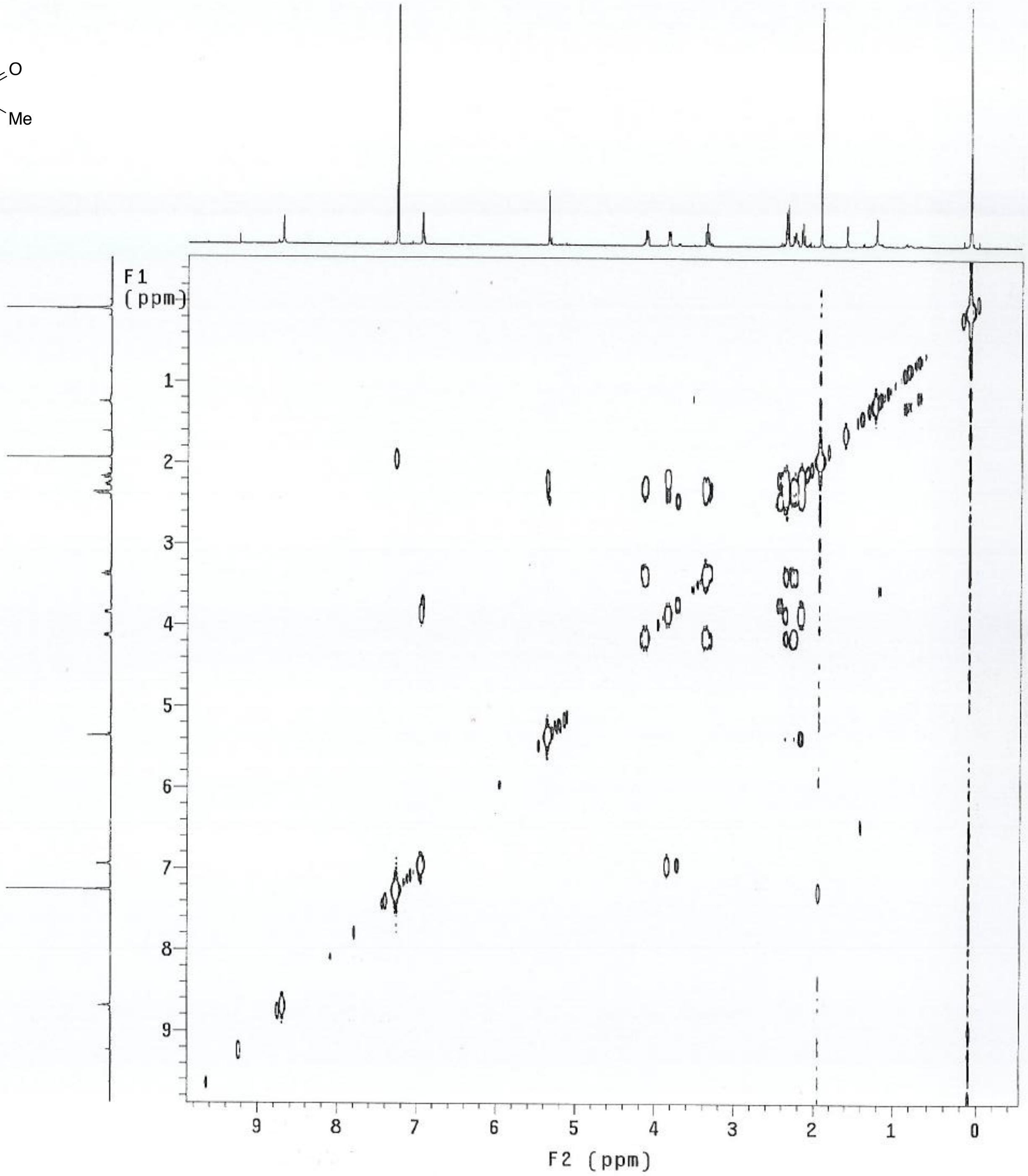

STANDARD PROTON PARAMETERS

Pulse Sequence: gCOSY

Solvent: CDCl3
Temp. 25.0 C / 298.1 K
User: 1-15-87
INOVA-600 "nmr4"

Relax. delay 1.500 sec
Acq. time 0.164 sec
Width 6255.4 Hz
2D Width 6255.4 Hz
2 repetitions
200 increments
OBSERVE H1, 599.7972910 MHz
DATA PROCESSING
Sq. sine bell 0.082 sec
F1 DATA PROCESSING
Sq. sine bell 0.014 sec
FT size 2048 x 2048
Total time 11 min, 43 sec

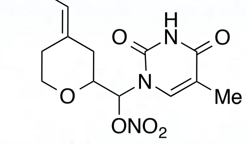

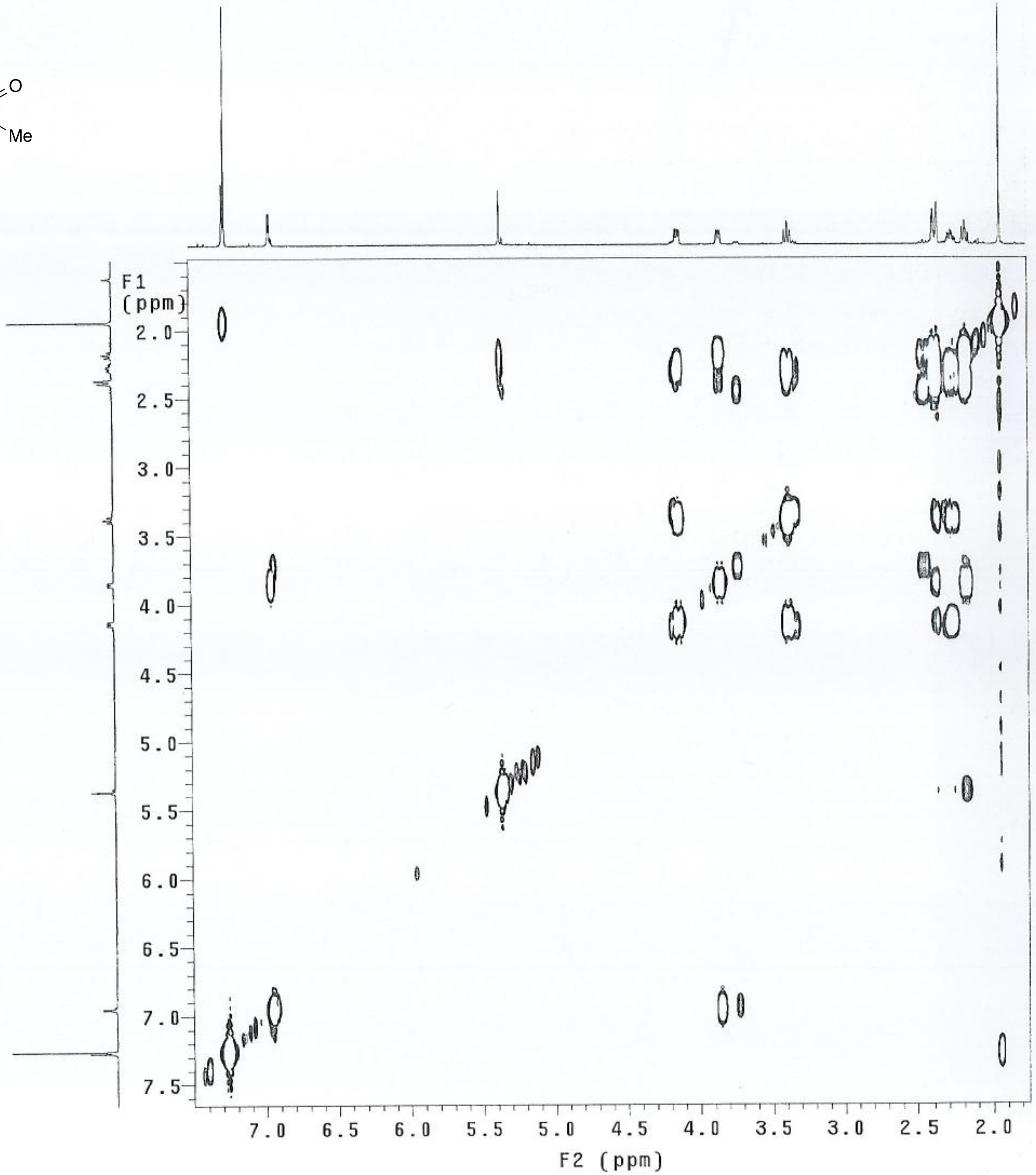

new experiment

Pulse Sequence: gHMBC
Solvent: CDCl3
Temp. 25.0 C / 298.1 K
User: 1-15-87
INOVA-600 "nmr4"

Relax. delay 2.000 sec
Acq. time 0.165 sec
Width 6218.9 Hz
2D Width 18235.7 Hz
16 repetitions
140 increments
OBSERVE H1, 599.7972910 MHz
DATA PROCESSING
Sq. sine bell 0.082 sec
F1 DATA PROCESSING
Sq. sine bell 0.001 sec
FT size 2048 x 2048
Total time 1 hr, 24 min, 23 sec

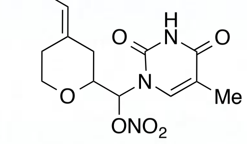

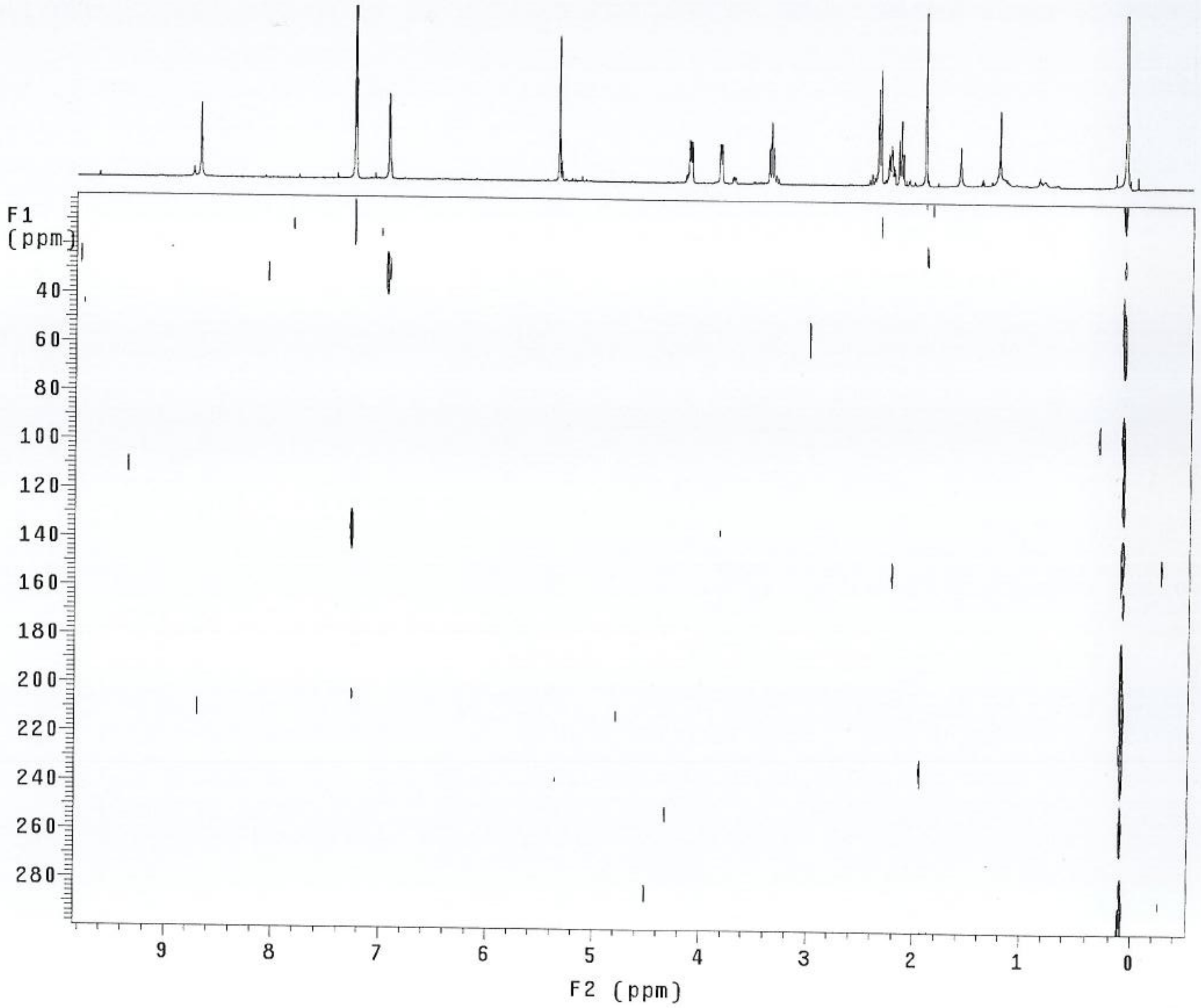

STANDARD PROTON PARAMETERS

Pulse Sequence: gHMBC

Solvent: CDCl3
Temp. 25.0 C / 298.1 K
User: 1-15-87
INOVA-600 "nmr4"

Relax. delay 1.700 sec
Acq. time 0.164 sec
Width 6255.4 Hz
2D Width 36199.1 Hz
8 repetitions
200 increments
OBSERVE H1, 599.7972910 MHz
DATA PROCESSING
Sq. sine bell 0.082 sec
F1 DATA PROCESSING
Sq. sine bell 0.003 sec
FT size 2048 x 2048
Total time 52 min, 59 sec

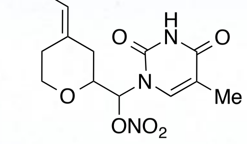

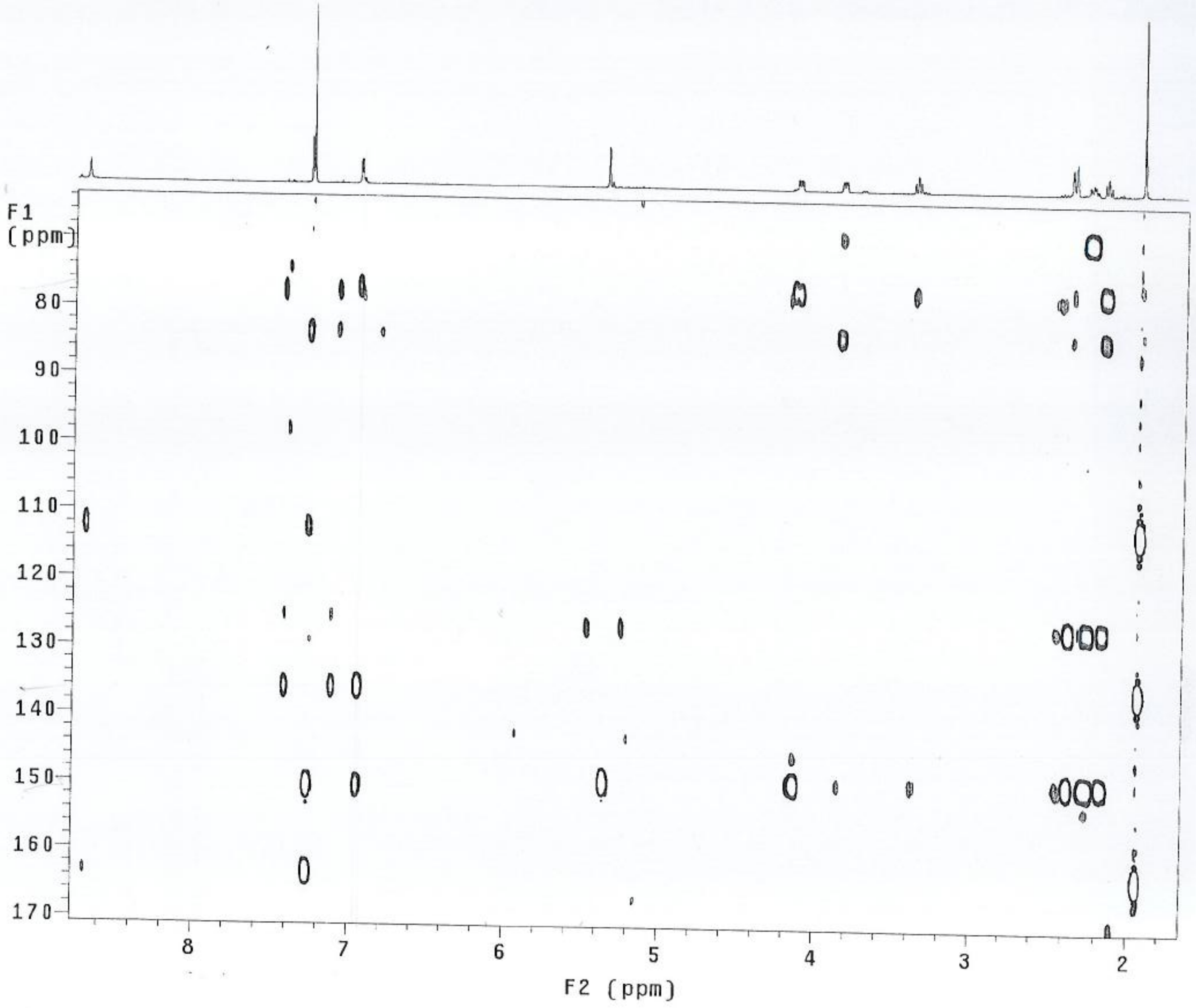

STANDARD PROTON PARAMETERS

Pulse Sequence: gHMBC

Solvent: CDCl3
Temp. 25.0 C / 298.1 K
User: 1-15-87
INOVA-600 "nmr4"

Relax. delay 2.000 sec
Acq. time 0.165 sec
Width 6218.9 Hz
2D Width 18235.7 Hz
32 repetitions
140 increments
OBSERVE H1, 599.7972910 MHz
DATA PROCESSING
Sq. sine bell 0.082 sec
F1 DATA PROCESSING
Sq. sine bell 0.002 sec
FT size 2048 x 2048
Total time 2 hr, 48 min, 28 sec

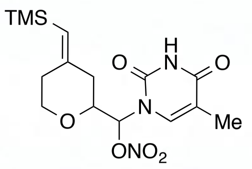

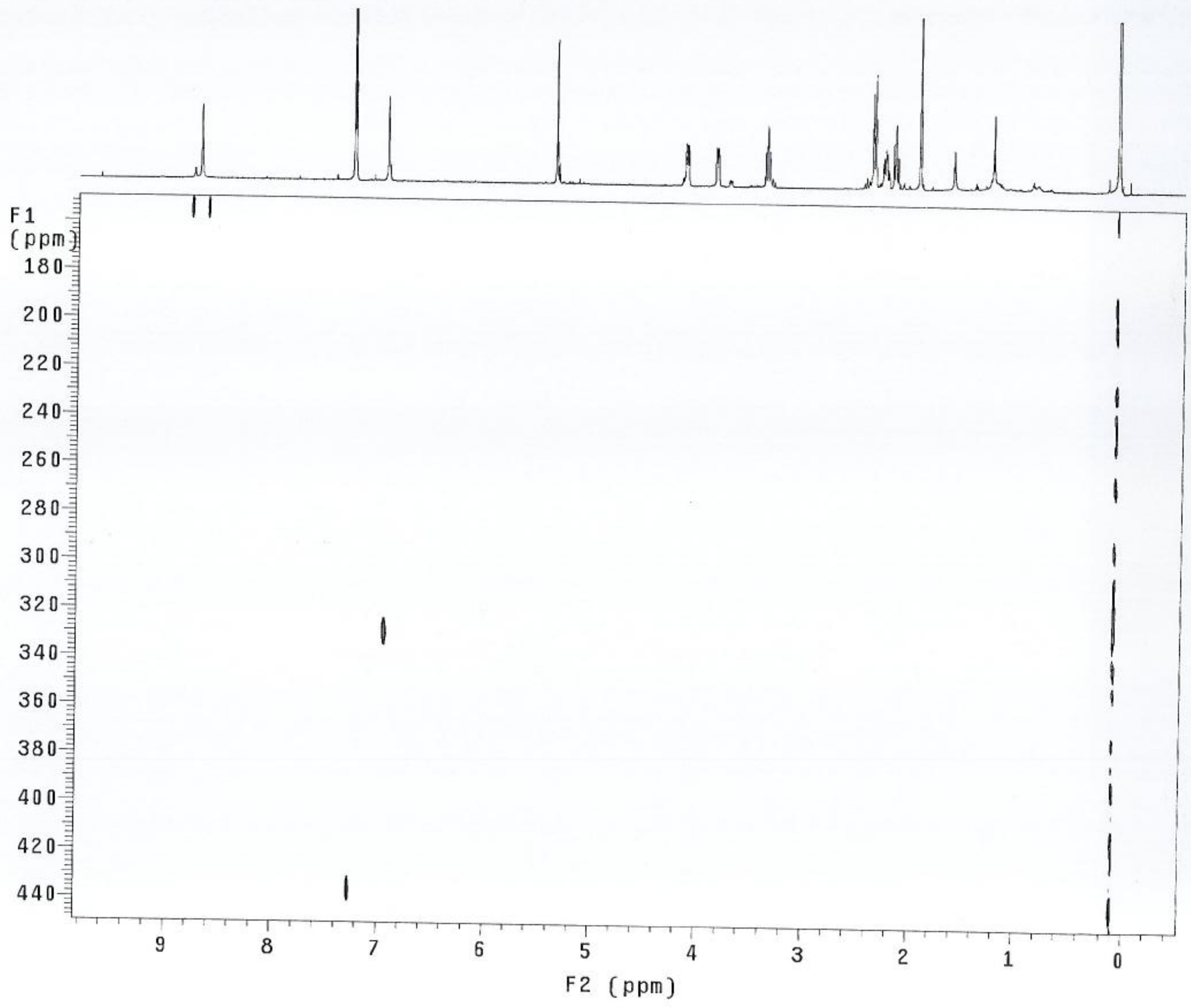

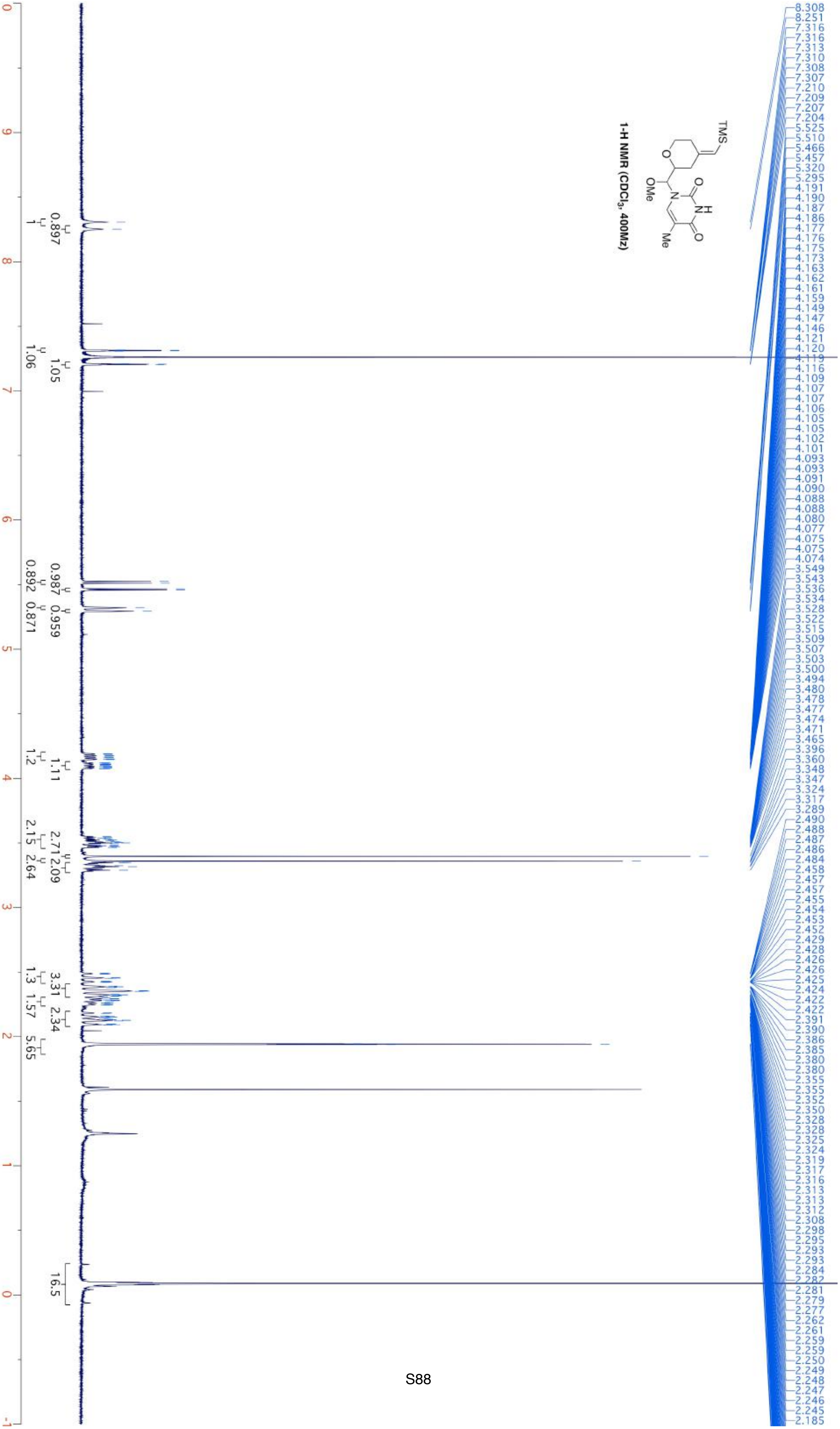
1-H NMR (CDCl$_3$, 400Mz)
TMS
OMe
Me
8.308
8.251
7.316
7.316
7.313
7.310
7.308
7.307
7.210
7.209
7.207
7.204
5.525
5.510
5.466
5.457
5.320
5.295
4.191
4.190
4.187
4.186
4.177
4.176
4.175
4.173
4.163
4.162
4.161
4.159
4.149
4.147
4.146
4.121
4.120
4.119
4.116
4.109
4.107
4.107
4.106
4.105
4.105
4.102
4.101
4.093
4.093
4.091
4.090
4.088
4.088
4.080
4.077
4.075
4.075
4.074
3.549
3.543
3.536
3.534
3.528
3.522
3.515
3.509
3.507
3.503
3.500
3.494
3.480
3.478
3.477
3.474
3.471
3.465
3.396
3.360
3.348
3.347
3.324
3.317
3.289
2.490
2.488
2.487
2.486
2.484
2.458
2.457
2.457
2.455
2.454
2.453
2.452
2.429
2.428
2.426
2.426
2.425
2.424
2.422
2.422
2.391
2.390
2.386
2.385
2.380
2.380
2.355
2.355
2.352
2.350
2.328
2.328
2.325
2.324
2.319
2.317
2.316
2.313
2.313
2.312
2.308
2.298
2.295
2.293
2.293
2.284
2.282
2.281
2.279
2.277
2.262
2.261
2.259
2.259
2.250
2.249
2.248
2.247
2.246
2.245
2.185

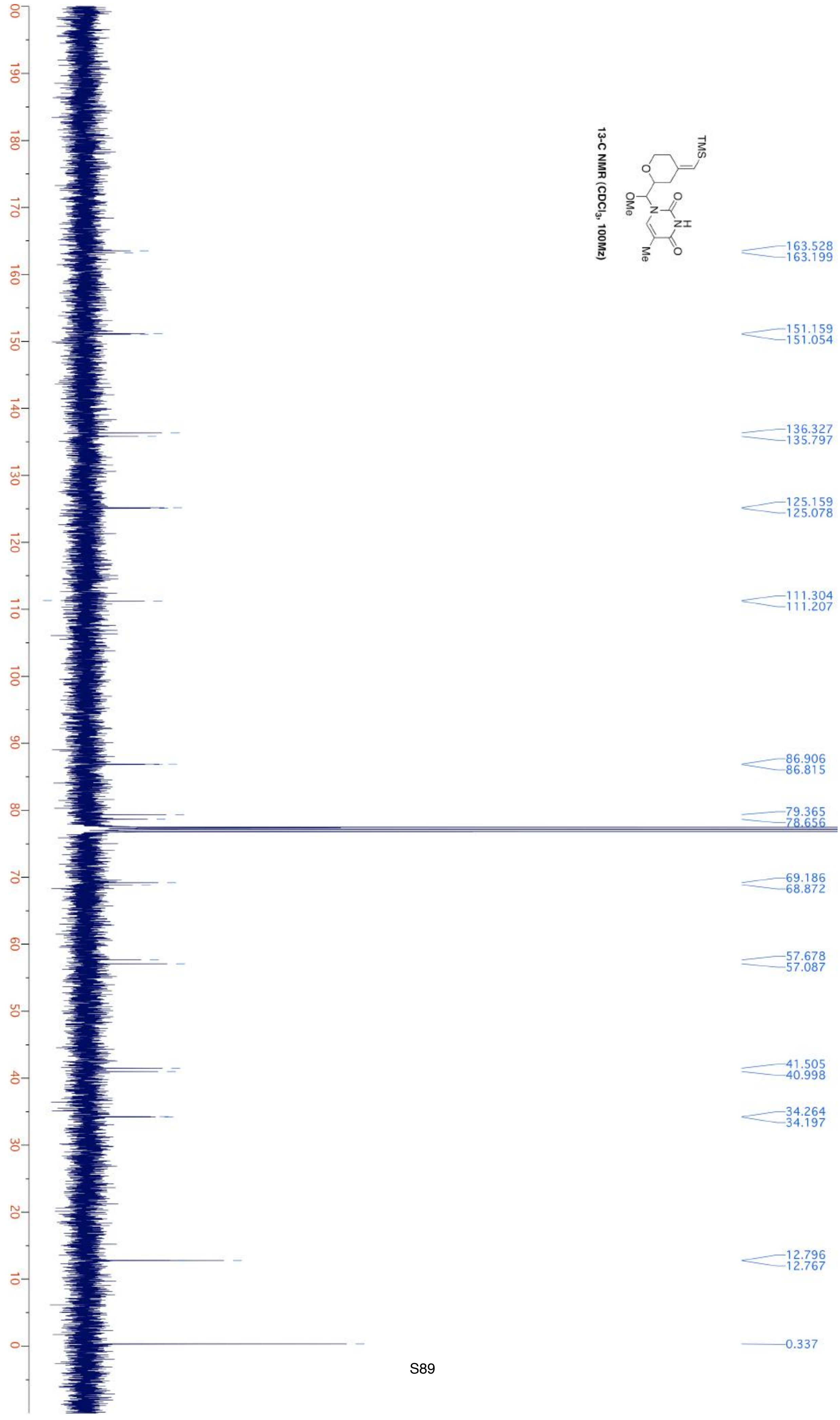
13-C NMR (CDCl3, 100Mz)
TMS
O
N
H
OMe
Me
163.528
163.199
151.159
151.054
136.327
135.797
125.159
125.078
111.304
111.207
86.906
86.815
79.365
78.656
69.186
68.872
57.678
57.087
41.505
40.998
34.264
34.197
12.796
12.767
0.337

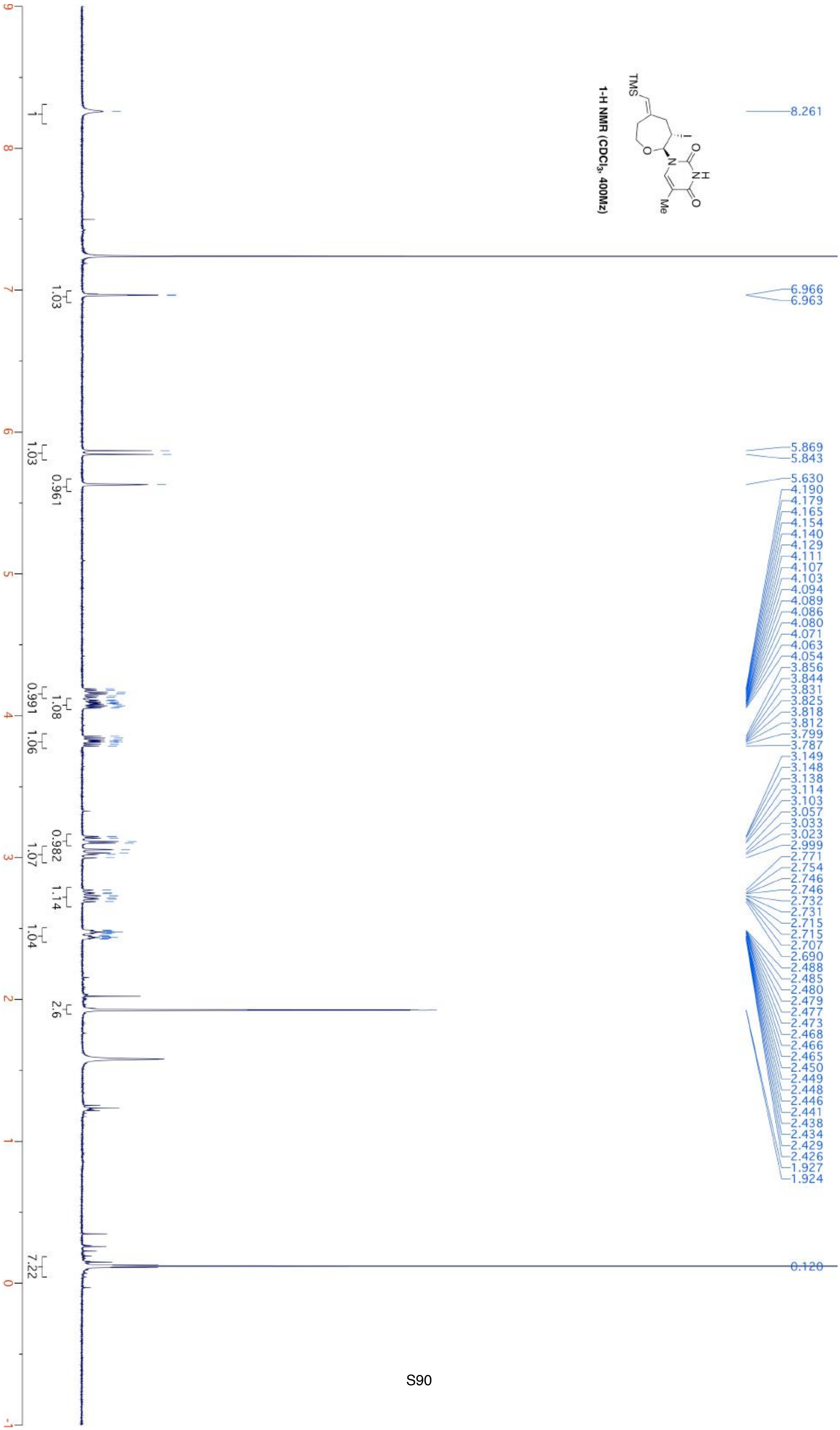
1-H NMR (CDCl$_3$, 400Mz)
TMS
Me
8.261
6.966
6.963
5.869
5.843
5.630
4.190
4.179
4.165
4.154
4.140
4.129
4.111
4.107
4.103
4.094
4.089
4.086
4.080
4.071
4.063
4.054
3.856
3.844
3.831
3.825
3.818
3.812
3.799
3.787
3.149
3.148
3.138
3.114
3.103
3.057
3.033
3.023
2.999
2.771
2.754
2.746
2.746
2.732
2.731
2.715
2.715
2.707
2.690
2.488
2.485
2.480
2.479
2.477
2.473
2.468
2.466
2.465
2.450
2.449
2.448
2.446
2.441
2.438
2.434
2.429
2.426
1.927
1.924
0.120
1
1.03
1.03
0.961
0.991
1.08
1.06
0.982
1.07
1.14
1.04
2.6
7.22

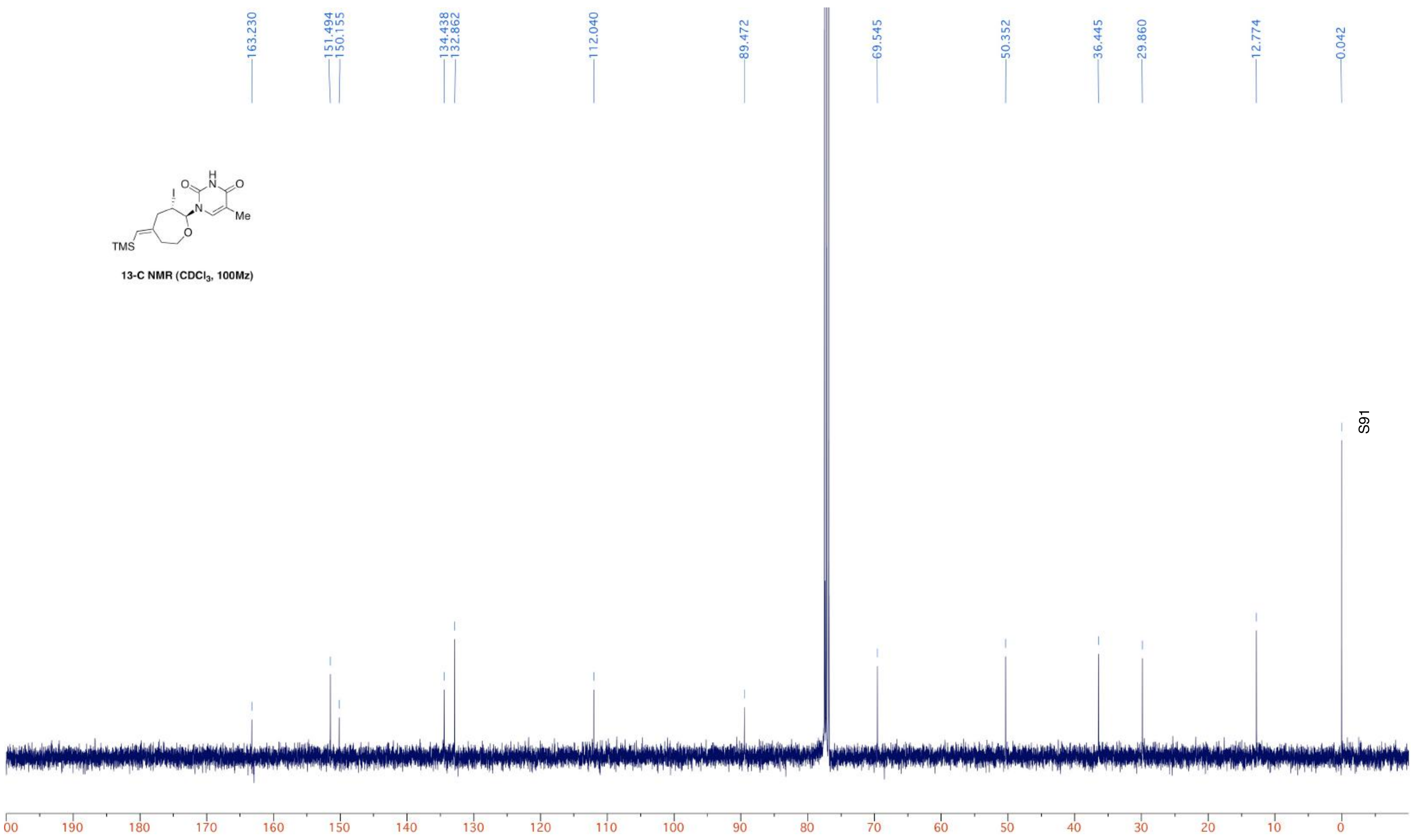
13-C NMR (CDCl$_3$, 100Mz)
TMS
I
O
H
N
Me
163.230
151.494
150.155
134.438
132.862
112.040
89.472
69.545
50.352
36.445
29.860
12.774
0.042
00
190
180
170
160
150
140
130
120
110
100
90
80
70
60
50
40
30
20
10
0

STANDARD PROTON PARAMETERS

Pulse Sequence: gCOSY

Solvent: CDCl3
Temp. 25.0 C / 298.1 K
User: 1-15-87
INOVA-600 "nmr4"

Relax. delay 1.500 sec
Acq. time 0.180 sec
Width 5683.4 Hz
2D Width 5683.4 Hz
2 repetitions
200 increments
OBSERVE H1, 599.7972910 MHz
DATA PROCESSING
Sq. sine bell 0.090 sec
F1 DATA PROCESSING
Sq. sine bell 0.012 sec
FT size 2048 x 2048
Total time 11 min, 51 sec

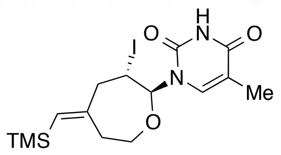

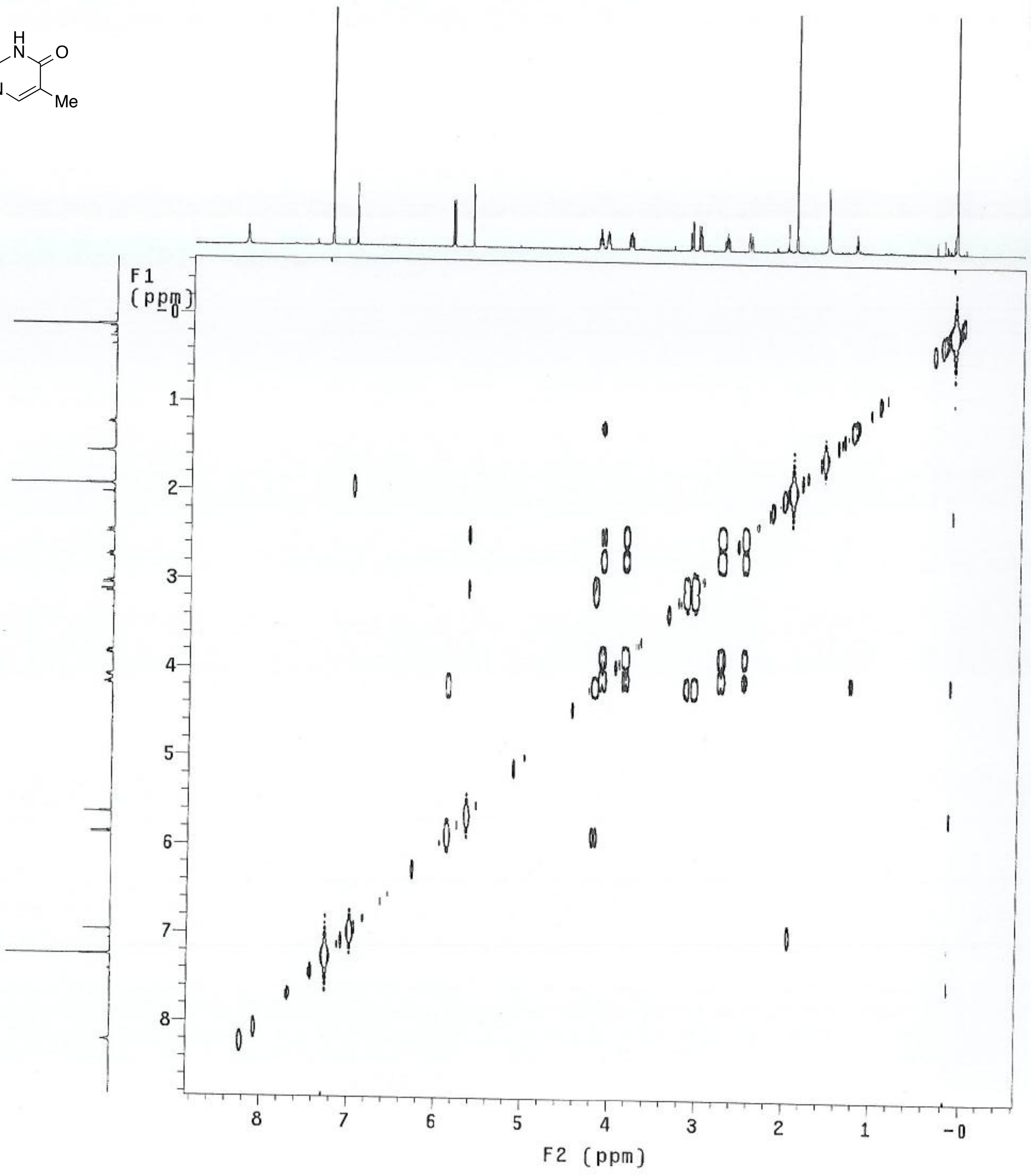

STANDARD PROTON PARAMETERS

Pulse Sequence: gHMBC

Solvent: CDCl3
Temp. 25.0 C / 298.1 K
User: 1-15-87
INOVA-600 "nmr4"

Relax. delay 1.500 sec
Acq. time 0.180 sec
Width 5683.4 Hz
2D Width 36199.1 Hz
8 repetitions
200 increments
OBSERVE H1, 599.7972910 MHz
DATA PROCESSING
Sq. sine bell 0.090 sec
F1 DATA PROCESSING
Sq. sine bell 0.002 sec
FT size 2048 x 2048
Total time 47 min, 59 sec

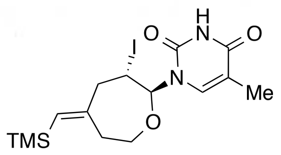

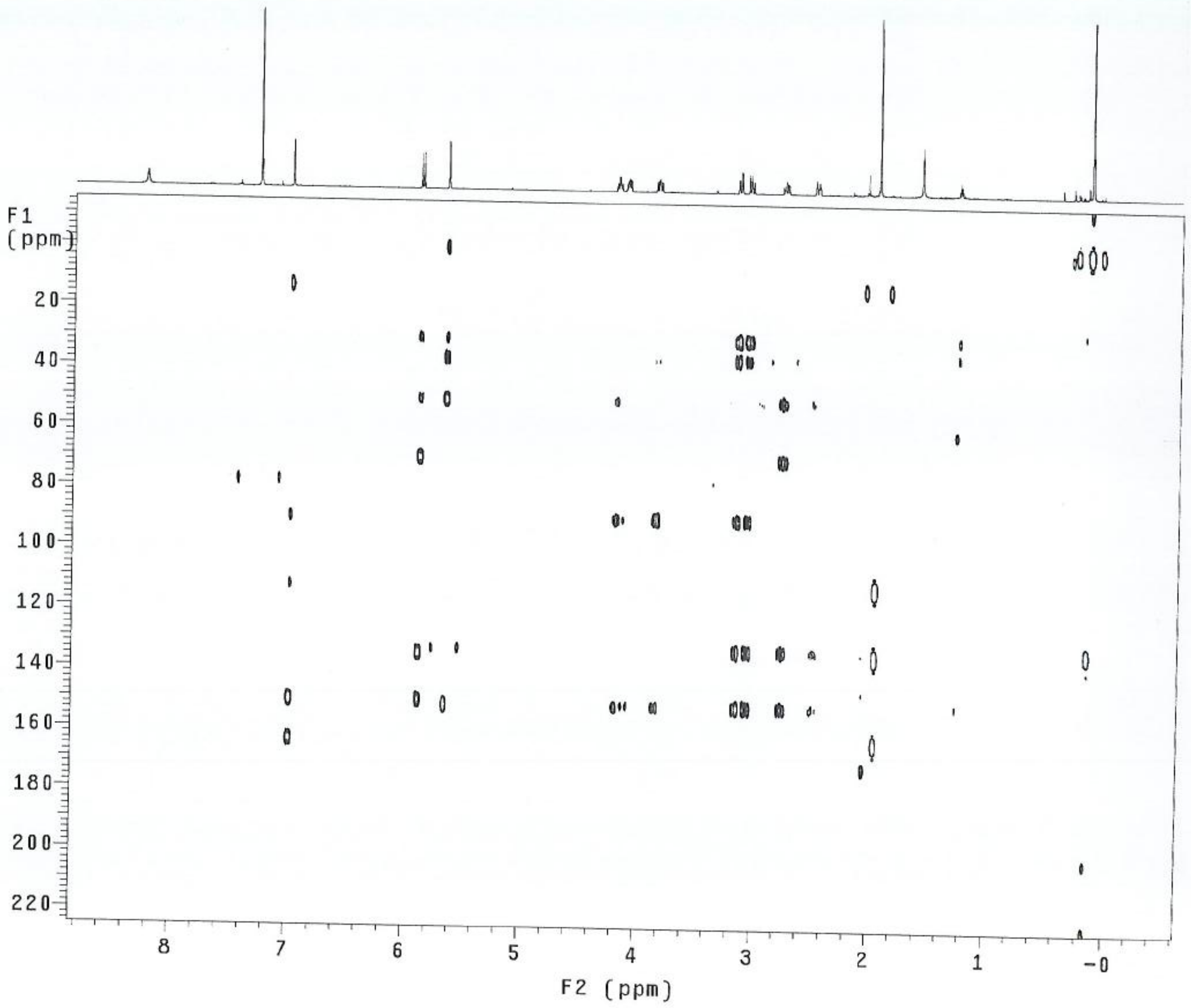

STANDARD PROTON PARAMETERS

Pulse Sequence: gHSQC

Solvent: CDCl3
Temp. 25.0 C / 298.1 K
User: 1-15-87
INOVA-600 "nmr4"

Relax. delay 1.500 sec
Acq. time 0.180 sec
Width 5683.4 Hz
2D Width 25641.0 Hz
2 repetitions
2 x 100 increments
OBSERVE H1, 599.7972910 MHz
DECOUPLE C13, 150.8304242 MHz
Power 48 dB
on during acquisition
off during delay
wurst180 modulated
DATA PROCESSING
Sq. sine bell 0.180 sec
Shifted by -0.180 sec
F1 DATA PROCESSING
Sq. sine bell 0.002 sec
Shifted by -0.002 sec
FT size 2048 x 2048
Total time 12 min, 36 sec

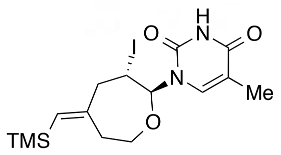

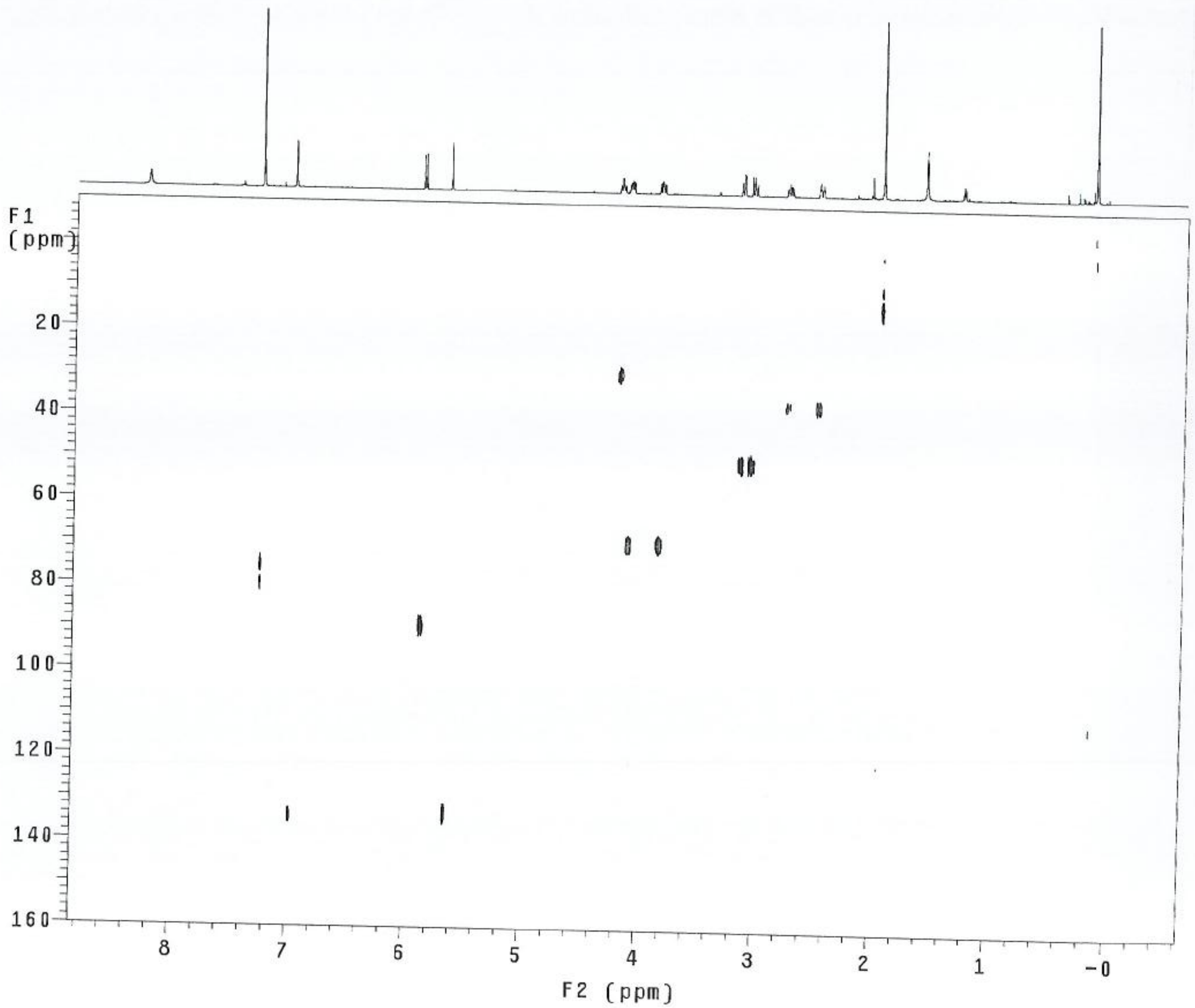

STANDARD PROTON PARAMETERS

Pulse Sequence: gHMBC

Solvent: CDCl3
Temp. 25.0 C / 298.1 K
User: 1-15-87
INOVA-600 "nmr4"

Relax. delay 1.500 sec
Acq. time 0.180 sec
Width 5683.4 Hz
2D Width 36199.1 Hz
8 repetitions
200 increments
OBSERVE H1, 599.7972910 MHz
DATA PROCESSING
Sq. sine bell 0.090 sec
F1 DATA PROCESSING
Sq. sine bell 0.002 sec
FT size 2048 x 2048
Total time 47 min, 59 sec

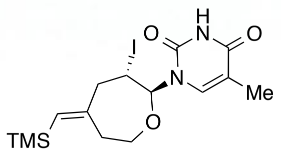

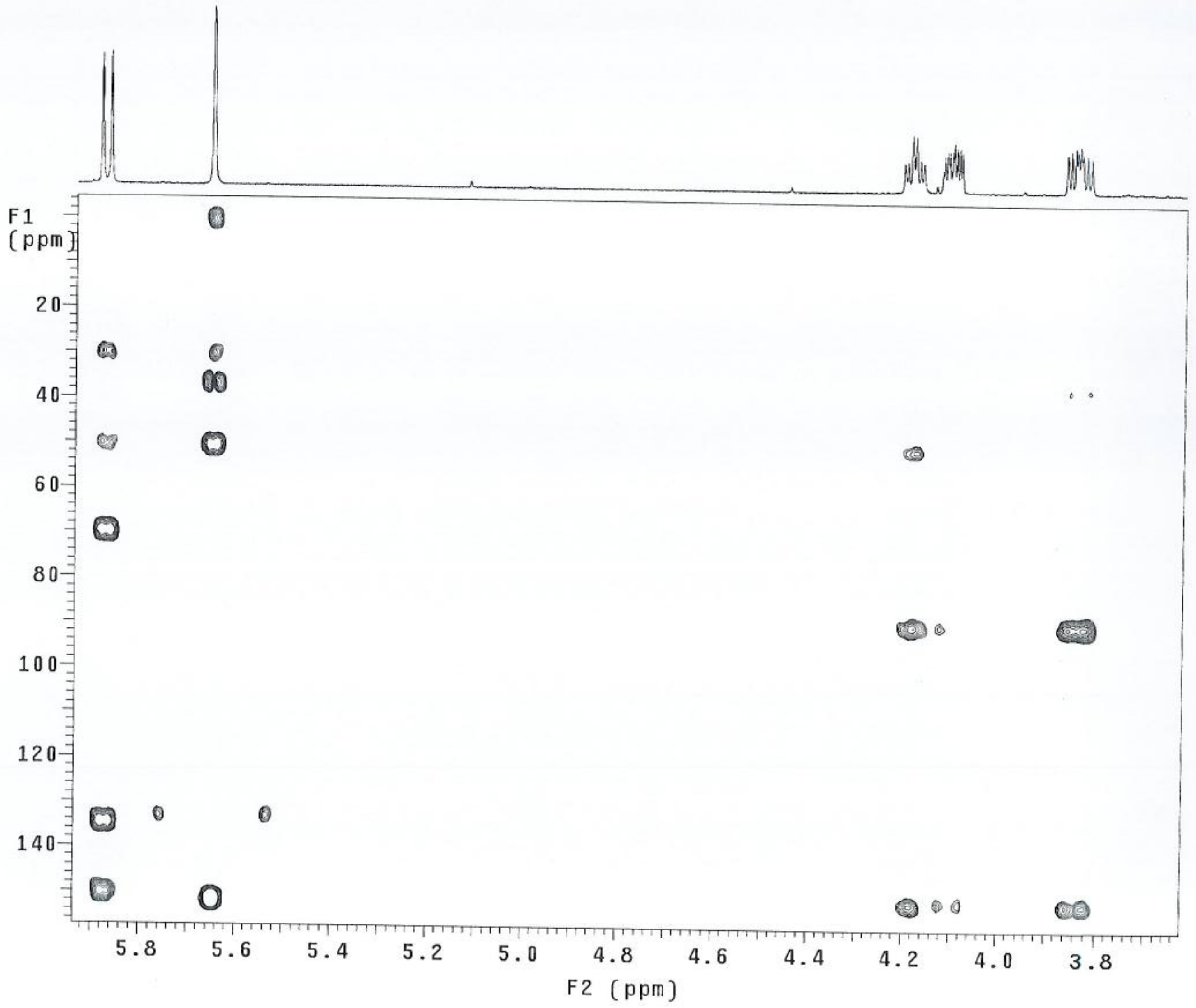

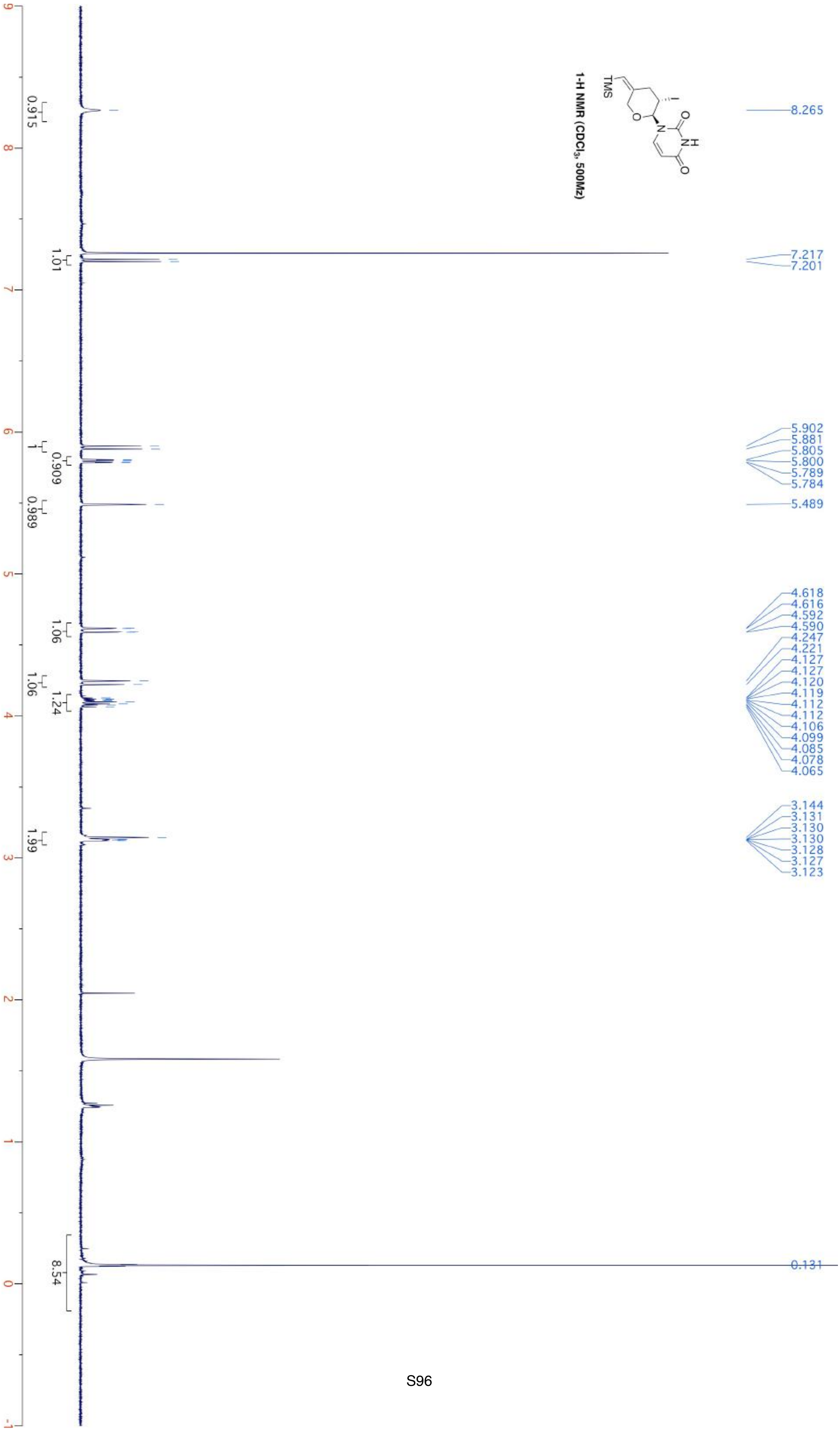
1-H NMR (CDCl3, 500Mz)
TMS
8.265
7.217
7.201
5.902
5.881
5.805
5.800
5.789
5.784
5.489
4.618
4.616
4.592
4.590
4.247
4.221
4.127
4.127
4.120
4.119
4.112
4.112
4.106
4.099
4.085
4.078
4.065
3.144
3.131
3.130
3.130
3.128
3.127
3.123
0.131
0.915
1.01
1
0.909
0.989
1.06
1.06
1.24
1.99
8.54

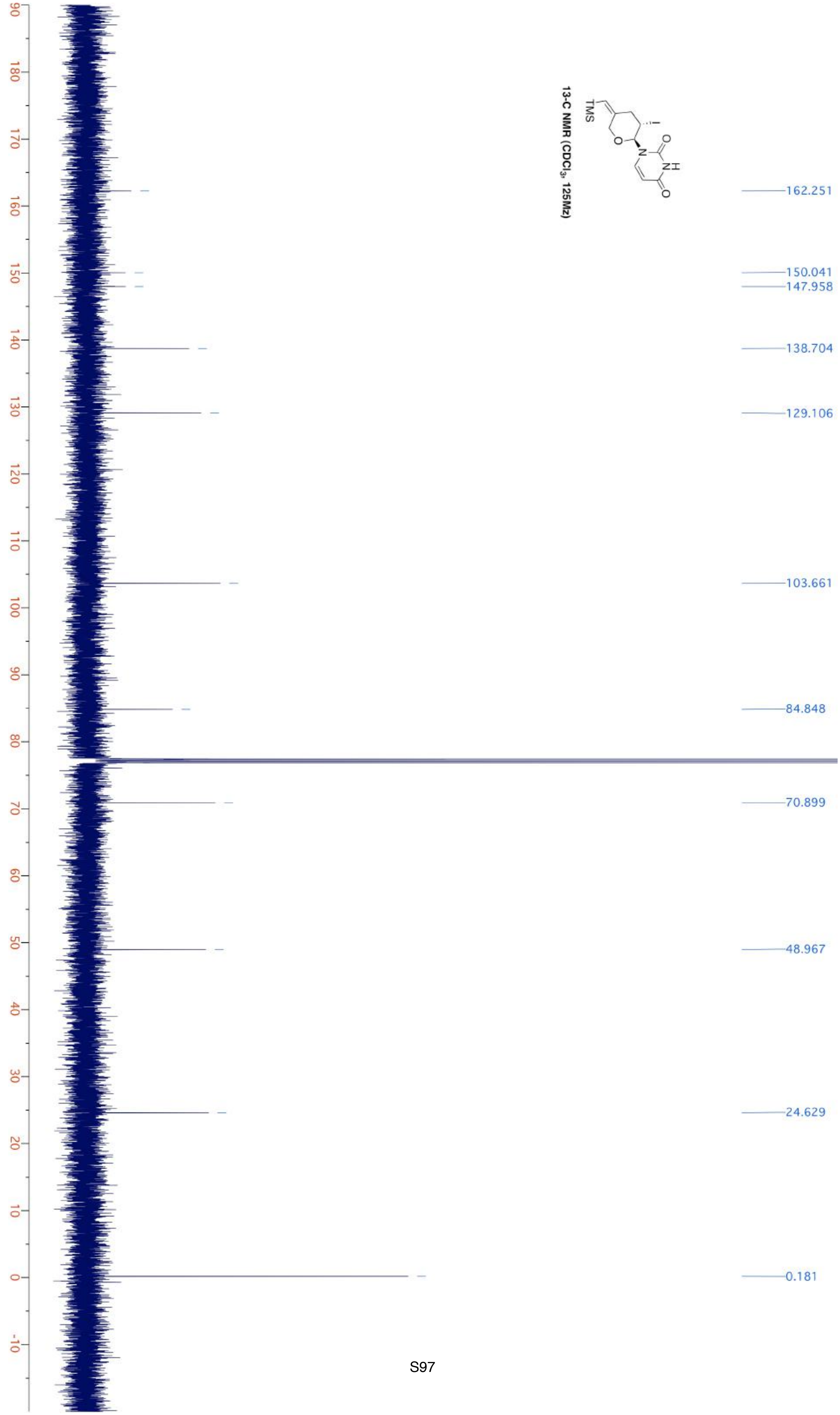
13-C NMR (CDCl3, 125Mz)
TMS
162.251
150.041
147.958
138.704
129.106
103.661
84.848
70.899
48.967
24.629
0.181
190
180
170
160
150
140
130
120
110
100
90
80
70
60
50
40
30
20
10
0
-10

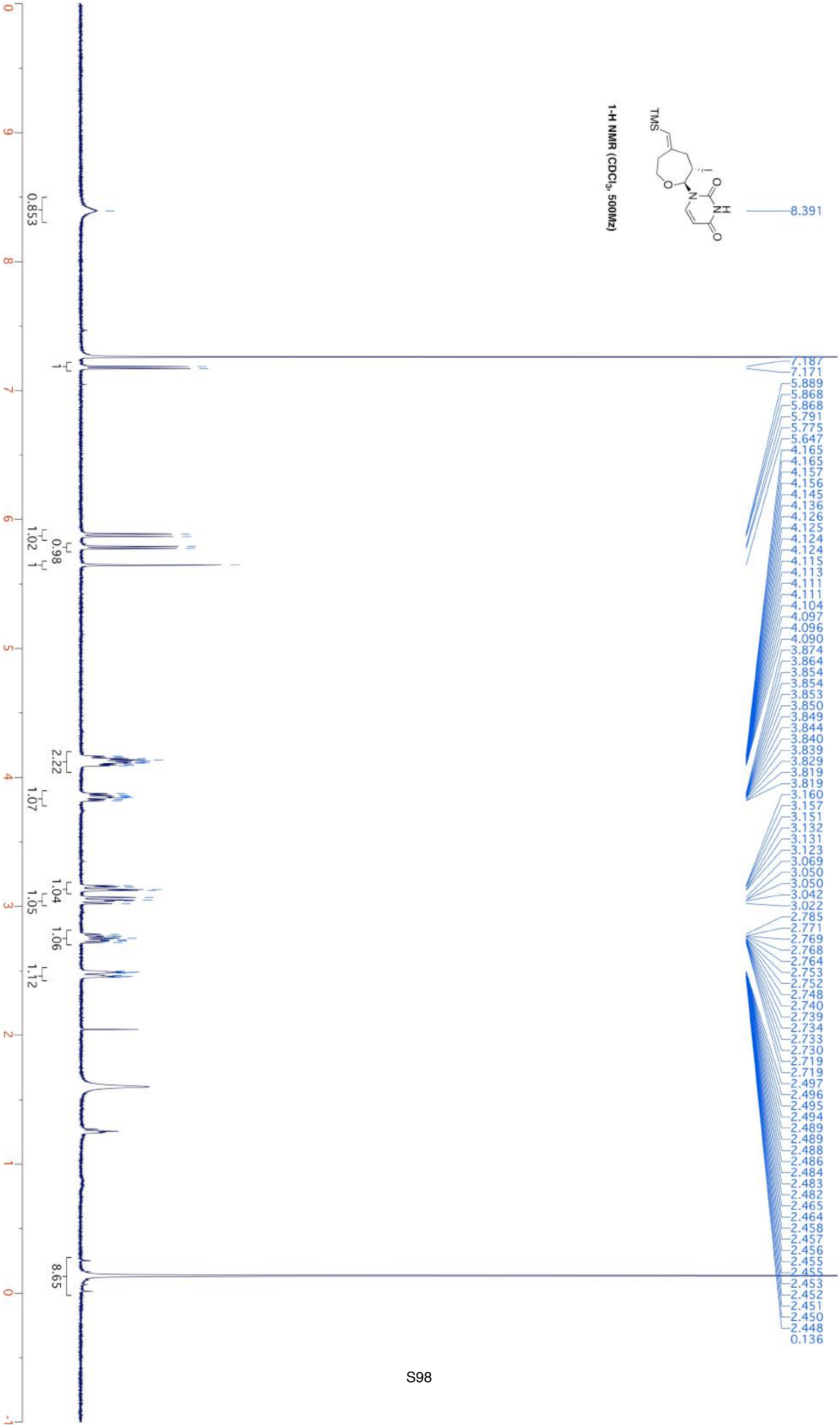
1-H NMR (CDCl3, 500Mz)
TMS
O
N
H
8.391
7.187
7.171
5.889
5.868
5.868
5.791
5.775
5.647
4.165
4.165
4.157
4.156
4.145
4.136
4.126
4.125
4.124
4.124
4.115
4.113
4.111
4.111
4.104
4.097
4.096
4.090
3.874
3.864
3.854
3.854
3.853
3.850
3.849
3.844
3.840
3.839
3.829
3.819
3.819
3.160
3.157
3.151
3.132
3.131
3.123
3.069
3.050
3.050
3.042
3.022
2.785
2.771
2.769
2.768
2.764
2.753
2.752
2.748
2.740
2.739
2.734
2.733
2.730
2.719
2.719
2.497
2.496
2.495
2.494
2.489
2.489
2.488
2.486
2.484
2.483
2.482
2.465
2.464
2.458
2.457
2.456
2.455
2.455
2.453
2.452
2.451
2.450
2.448
0.136
0.853
1
1.02
0.98
1
2.22
1.07
1.04
1.05
1.06
1.12
8.65
9
8
7
6
5
4
3
2
1
0
-1

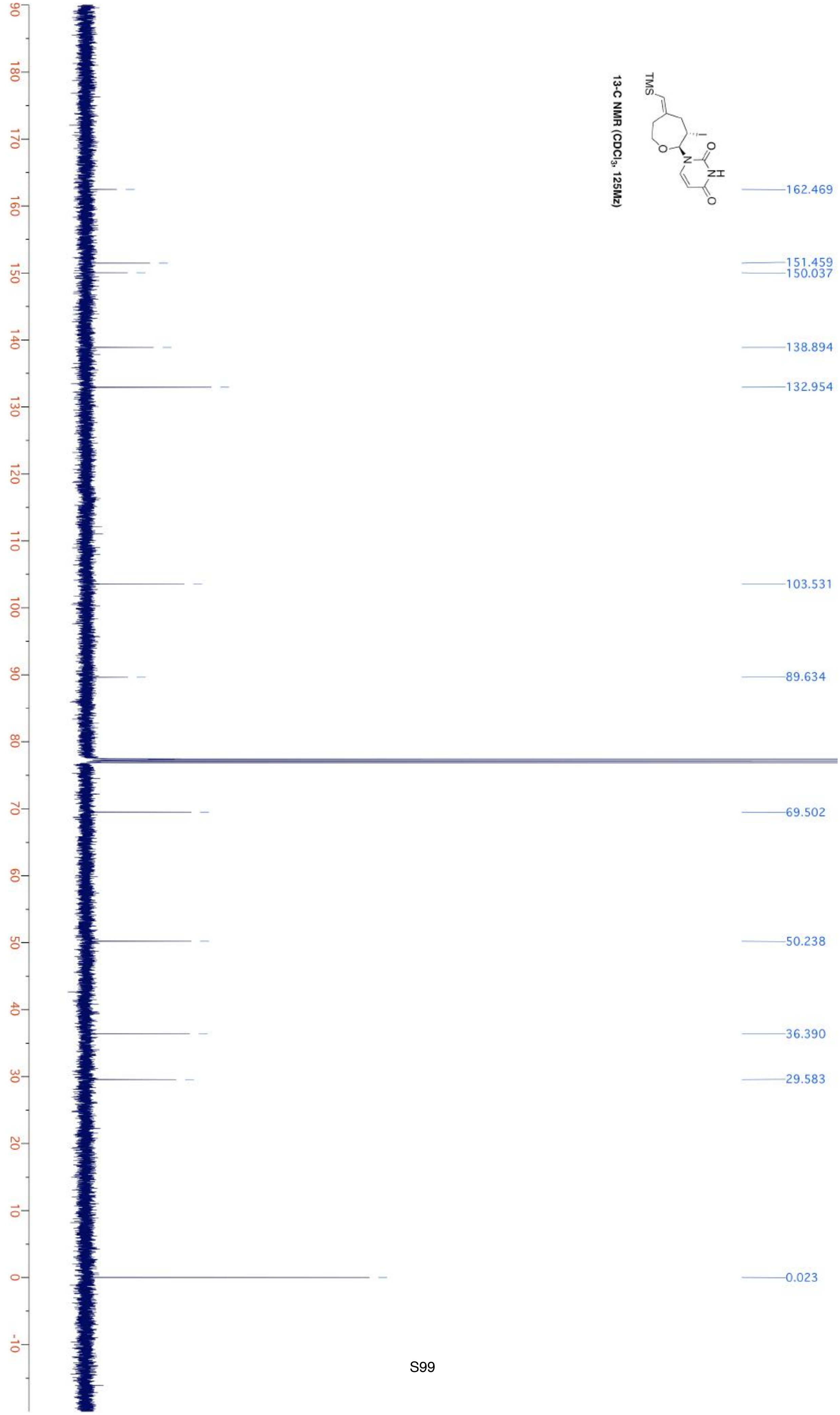
13-C NMR (CDCl3, 125Mz)
TMS
162.469
151.459
150.037
138.894
132.954
103.531
89.634
69.502
50.238
36.390
29.583
0.023
190
180
170
160
150
140
130
120
110
100
90
80
70
60
50
40
30
20
10
0
-10

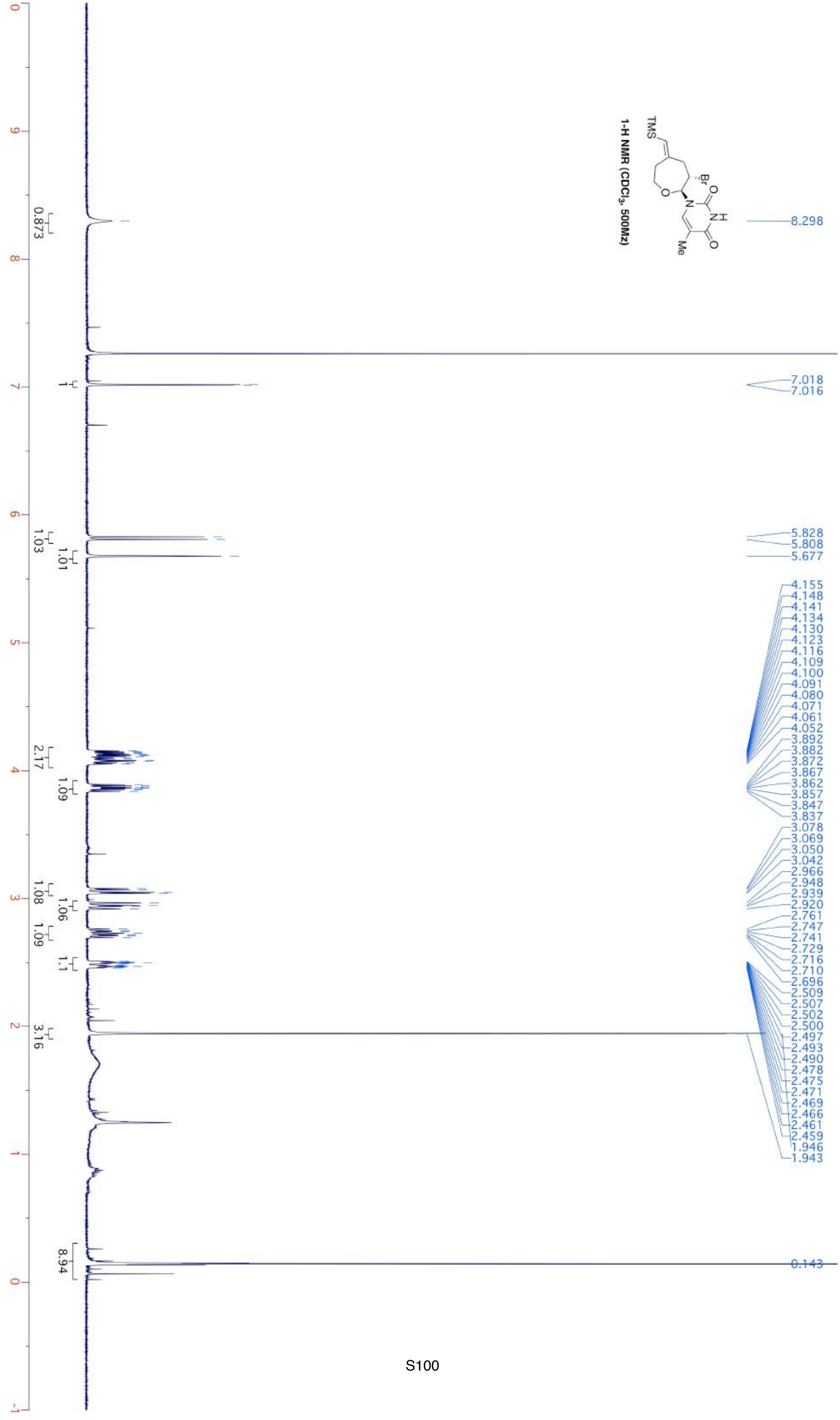
1-H NMR (CDCl3, 500Mz)
TMS
Br
O
N
H
Me
8.298
7.018
7.016
5.828
5.808
5.677
4.155
4.148
4.141
4.134
4.130
4.123
4.116
4.109
4.100
4.091
4.080
4.071
4.061
4.052
3.892
3.882
3.872
3.867
3.862
3.857
3.847
3.837
3.078
3.069
3.050
3.042
2.966
2.948
2.939
2.920
2.761
2.747
2.741
2.729
2.716
2.710
2.696
2.509
2.507
2.502
2.500
2.497
2.493
2.490
2.478
2.475
2.471
2.469
2.466
2.461
2.459
1.946
1.943
0.143
0.873
1
1.03
1.01
2.17
1.09
1.08
1.06
1.09
1.1
3.16
8.94

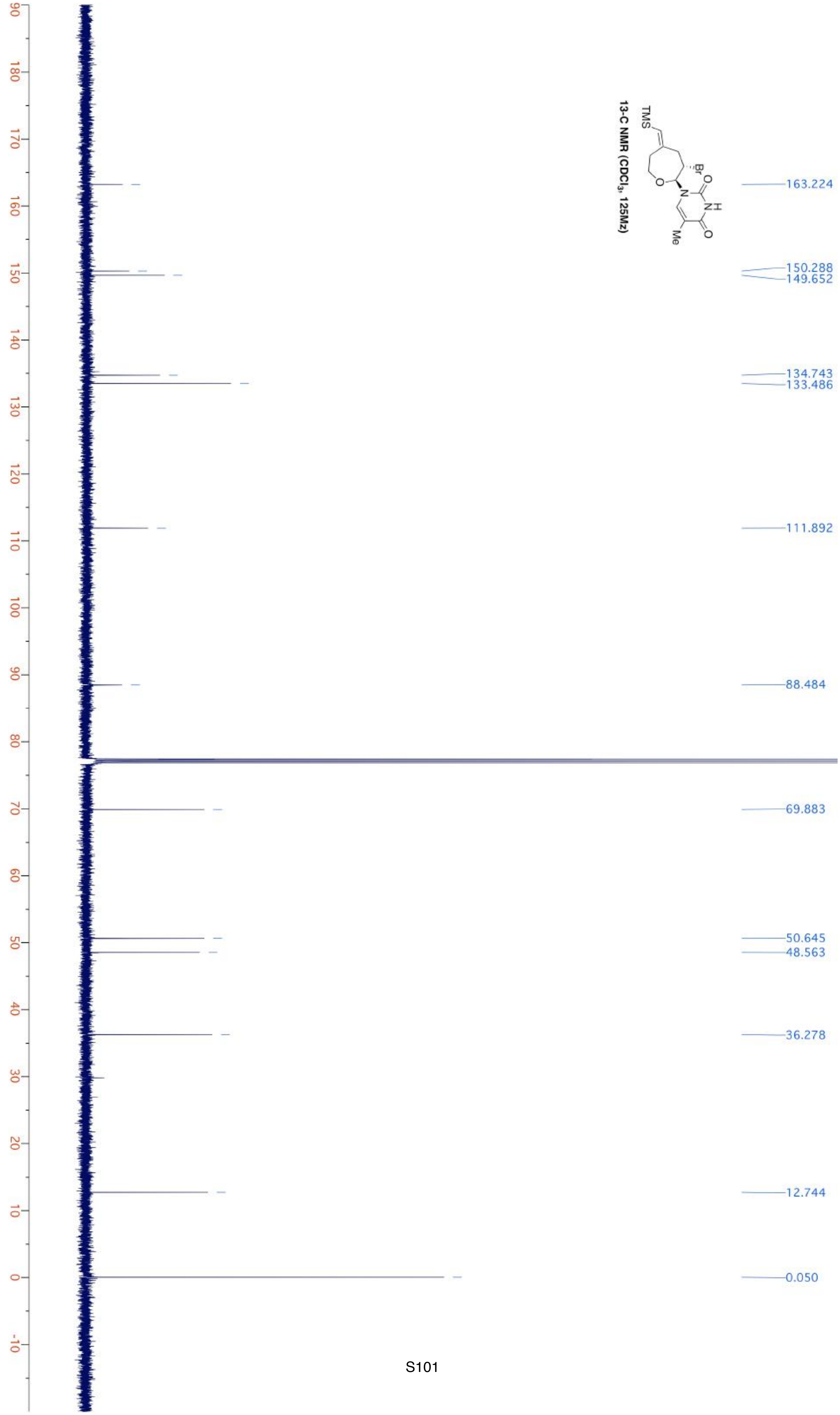
13-C NMR (CDCl3, 125Mz)
TMS
Br
O
N
H
Me
163.224
150.288
149.652
134.743
133.486
111.892
88.484
69.883
50.645
48.563
36.278
12.744
0.050
190
180
170
160
150
140
130
120
110
100
90
80
70
60
50
40
30
20
10
0
-10

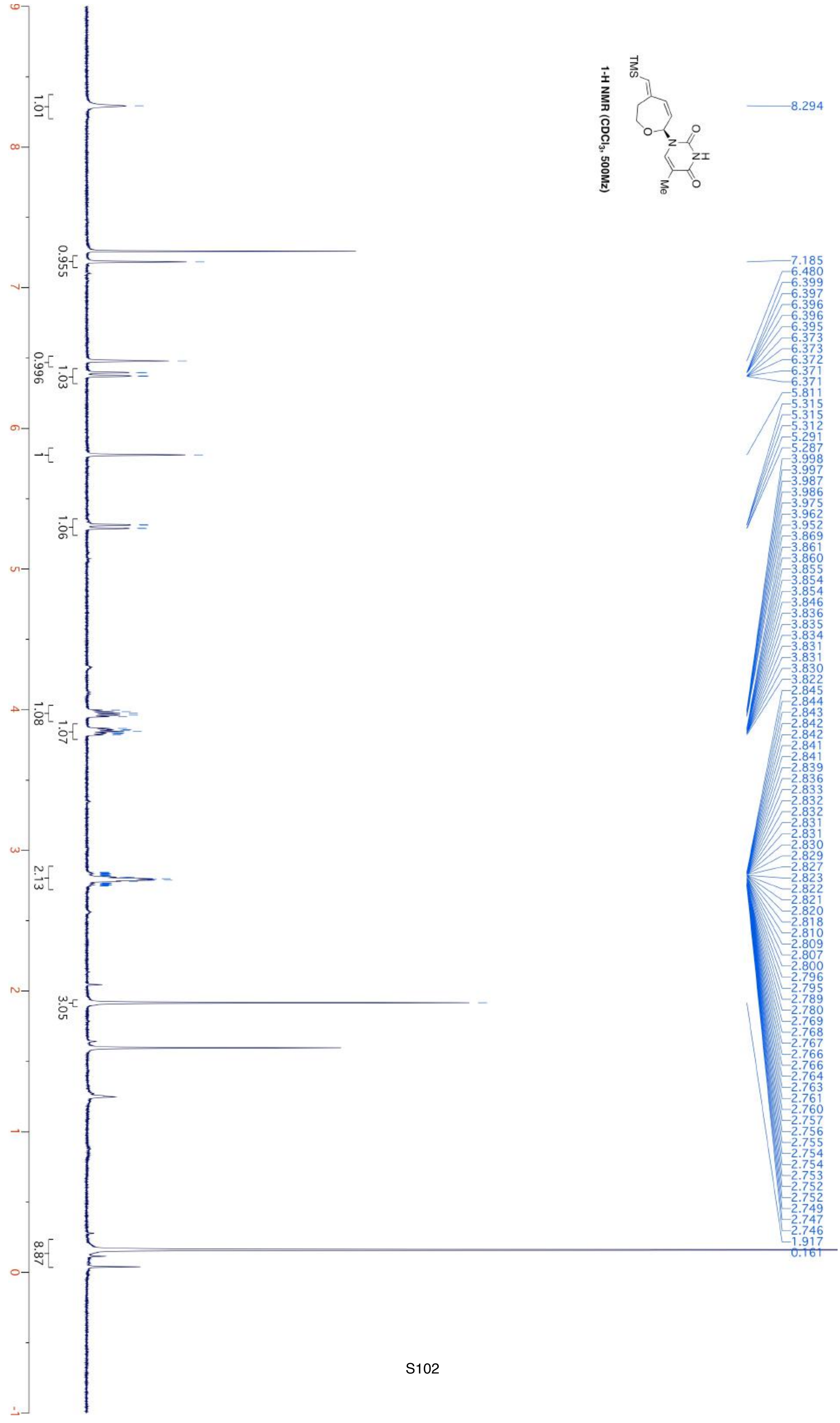
1-H NMR (CDCl3, 500Mz)

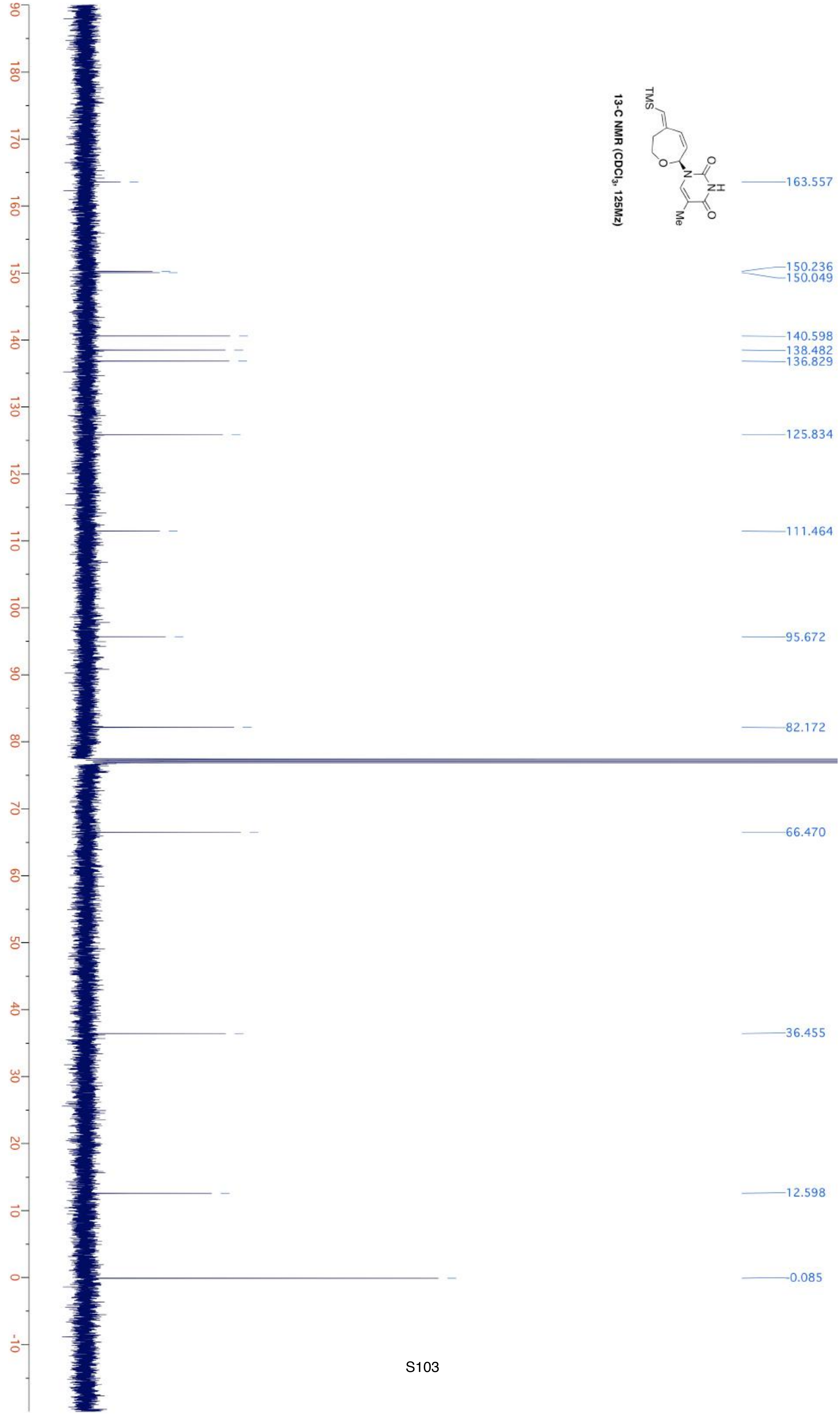

TMS
O
N
O
NH
O
Me
13-C NMR (CDCl3, 125Mz)
163.557
150.236
150.049
140.598
138.482
136.829
125.834
111.464
95.672
82.172
66.470
36.455
12.598
0.085
